# Healthy lifestyle behaviors are major predictors of mental wellbeing during COVID-19 pandemic confinement: A study on adult Arabs in higher educational institutions

**DOI:** 10.1371/journal.pone.0243524

**Published:** 2020-12-14

**Authors:** Hashem A. Kilani, Mo’ath F. Bataineh, Ali Al-Nawayseh, Khaled Atiyat, Omar Obeid, Maher M. Abu-Hilal, Taiysir Mansi, Maher Al-Kilani, Mahfoodha Al-Kitani, Majed El-Saleh, Ruba M. Jaber, Ahmad Sweidan, Mawaheb Himsi, Iyad Yousef, Faten Alzeer, Monther Nasrallah, Ayesha S. Al Dhaheri, Abdulsalam Al-Za’abi, Osama Allala, Laila Al-Kilani, Asma M. Alhasan, Mohamed Ghieda, Yasir Najah, Saad Alsheekhly, Ahmad Alhaifi, Raghda Shukri, Jamal Al Adwani, Mostafa Waly, Laila Kilani, Leen H. Kilani, Ahmad S. al Shareef, Areej Kilani

**Affiliations:** 1 Kinesiology and Training Department, School of Sport Sciences, University of Jordan, Amman, Jordan; 2 Department of Sport Rehabilitation, College of Physical Education and Sport Science, Hashemite University, Zarqa, Jordan; 3 Department of Nutrition and Food Sciences, American University of Beirut, Beirut, Lebanon; 4 Psychology Department, College of Education, Sultan Qaboos University, Muscat, Oman; 5 Physical Education & Sports Sciences, College of Education, Sultan Qaboos University, Muscat, Oman; 6 College of Education, Humanities and Social Sciences, Al-Ain University, Al-Ain, United Arab Emirates; 7 School of Medicine, Family Medicine, University of Jordan, Amman, Jordan; 8 Ministry of Education, Amman, Jordan; 9 Wellness Department, Actness Academy, Amman, Jordan; 10 Physical Education Department, Beir Zeit University, Ramallah, Palestine; 11 Physical Education Department, Khadouri University, Ramallah, Palestine; 12 Military Physical Training Department, Al Istiqlal University, Jericho, Palestine; 13 Food, Nutrition and Health Department, United Arab Emirates University, Al-Ain, United Arab Emirates; 14 Health and Physical Education Department, College of Education, UAEU, Al-Ain, Abu Dhabi, United Arab Emirates; 15 Sports Health Specialist, Abu Dhabi Sports Council, Abu Dhabi, United Arab Emirates; 16 Department of Physical & Sport Sciences, Princess Nourah bint Abdulrahman University, Riyadh, Saudi Arabia; 17 Sport Kinesiology Department, Mansoura University, Mansoura, Egypt; 18 College of Physical Education and Sports Science, University of Baghdad, Baghdad, Iraq; 19 College of Political Science, University of Baghdad, Baghdad, Iraq; 20 College of Health Sciences, The Public Authority for Applied Education and Training, Adailiya, Kuwait; 21 Nursing Department, Head of Nursing Department, Tokyo Human Health Sciences University Vietnam, Ho Chi Minh City, Vietnam; 22 Ministry of Awqaf and Islamic Affairs, Kuwait City, Kuwait; 23 Food Science and Nutrition Department, College of Agricultural and Marine Sciences, Sultan Qaboos University, Muscat, Oman; 24 Clinical Pharmacist, University of Jordan, Amman, Jordan; 25 College of Engineering, University of Jordan, Amman, Jordan; 26 Drassa Academy, Dubai, United Arab Emirates; 27 Internal Medicine Department, Jordan University Hospital, Amman, Jordan; University Sains Malaysia, MALAYSIA

## Abstract

**Background:**

In the past infectious diseases affected the quality of lifestyle during home confinement. The study conducted examines the influence of home confinement during the COVID-19 pandemic outbreak on lifestyle, mental wellbeing, nutritional status, and sleeping pattern.

**Method:**

An online multicategorical questionnaire was distributed to collect demographic information combined with the following tools: Food Frequency Questionnaire (FFQ), International Physical Activity Questionnaire (IPAQ), WHO-5 wellbeing score, and Pittsburgh Sleep Quality Index (PSQI). A snowball non-discriminate sampling procedure was conducted to collect data from people attending or working at higher institutions from March 1, 2020 to April 24, 2020. A total of 1723 completed responses (917 males, 37.4 ±13.4 years old and 806 females 32.2 ± 11.5 years old) were collected.

**Results:**

The female participants had significantly lower mental health scores than males (53.9% vs. 46.1%). The mental wellbeing scores were higher among participants with medium and high physical activity (PA) levels (p < 0.00). Additionally, the mental wellbeing scores were significantly improved by dietary quality and it’s sleeping score (p < 0.001). However, PA was by far the major determinant of the mental health scores.

**Conclusion:**

Factors such as PA, diet, and sleeping patterns were associated with mental wellbeing during the COVID-19 confinement among Arab participants.

## Introduction

Over the past few centuries, Infectious diseases such as cholera, plague, and yellow fever have resurfaced [1] as a result of the major epidemics that has occurred; in which has affected the health status of the people who were affected by the presence of these chronic and infectious diseases [1]. In the past few decades, new infectious diseases such as Severe Acute Respiratory Syndrome (SARS), Middle East Respiratory Syndrome (MERS), Zika, and now the novel coronavirus known as Coronavirus Disease 2019 (COVID-19) have emerged [2, 3].

In December 2019, Wuhan city, the capital of Hubei province in China, became the center of an outbreak of pneumonia disease for unknown causes [4]. Since then, the disease has spread quickly around the globe and the World Health Organization (WHO) has declared it as a global pandemic [5]. By the end of May 2020, about 6 million people were reported to be infected globally, with an increasingly accelerating death rate of about 370,000, the number of cases are increasing exponentially on a daily basis [5].

As a result, COVID-19 has accelerated the number of daily deaths, has led to economic collapses, increased unemployment levels, caused financial losses of major international companies. It has also restricted travelling, and forced daily home confinement. This has become a concern for many people worldwide and has impacted their mental wellbeing dramatically. Although governments have planned and estimated their capacities in containing the spread of the disease and providing patients with treatment, they have neglected the negative impacts on the mental wellbeing across the globe. For example, suicide rates have increased in many countries, in which reflected a poor understanding of the mental wellbeing effects associated with the COVID-19 pandemic [6, 7]. Moreover, an urgent need for timely attention and awareness of mental health care, social support/work programs, and optimal treatment interventions for mental disorders; has emerged. For example, anxiety is the biggest mental challenge people face today, followed by their inability to work remotely and feelings isolation from their social life. It is also has become imperative to pay attention to other lifestyle factors that can enhance mental wellbeing such as sleep quality, dietary behaviors, physical activity, sitting time and sedentary life [8, 9].

The recommended procedures to prevent the spread of the disease so far have focused on social distancing (minimum of 1.5-meters) and home confinement [5, 8]. These were imposed by several governments and were reported to be “very effective” for people who choose to stay indoors [8]. However, home confinement, and curfews are a cause of concern as they have impacted physical activity (PA), eating behaviors, and mental health of the people globally [9].

COVID-19 has impacted our social lives as we know it. Most Arab countries have adopted extreme measures to prevent the spread of the disease and protect their citizens, following the Chinese approach of aggressive isolation measures, which led to a progressive reduction of cases [10, 11]. Thus, academic institutions were closed as early as March 13^th^ and shifted to online activities. Curfew was enforced, few cities were isolated, and access to workplaces, gyms, and other social facilities were prohibited. Grocery stores (convenience stores), bakeries, and medical facilities were the only available services for people to access, as they could walk to or -visit during limited hours of the day especially old people over 60 years of age. These measures favor a sedentary lifestyle due to low mobility and may impact cardiovascular and mental health integrities.

The American Heart Association [12] indicated that “Prevention is the key to limiting the spread of coronavirus and as more people work remotely or reduce their public exposure, it is important to maintain healthy habits at home.” PA includes all forms of skeletal muscle-driven movements that encompass activities of differing levels of intensity. PA include leisure time activities such as walking, hiking, gardening, cycling, and dancing. Although competitive sports have been prohibited, indoor games, cleaning the house, and carrying heavy shopping bags can be counted as a compensatory PA. Also, short breaks from extended periods spent sitting by performing physical movements for 3–5 minutes every one hour have a significant impact on health [13]. Walking or stretching exercises could help relieve muscle fatigue, mental tension, and improve blood circulation and overall physical wellness [13].

Prior to this pandemic and the associated confinement, individuals’ health was already compromised due to a sedentary lifestyle that triggers hypokinetic diseases. The resultant lifestyle modifications may become a severe threat that could affect a number of daily activities for people of all ages. The benefits of physical activities in this emerging condition are expected to exceed their known benefits on improvements to mental status and could help people cope with the new stay-at-home status quo and social-life withdrawal. It has been reported that moderate to regular PA can enhance the immune response [14]. Reports also indicate that moderate to regular PA is inversely related to upper respiratory tract infection (URTI) occurrences, which are usually caused by viral agents [15].

Non-Exercise Activity Thermogenesis (NEAT) and thus total energy expenditure, which may have long term implication on body weight, life expectancy and to manage lifestyle [16]. PA-related energy expenditure is comprised almost entirely of NEAT. Therefore, NEAT represents the main variable component of daily total energy. Moreover, home confinement may alter eating behaviors resulting from boredom and access to food. Thus, balanced nutrition becomes vital to support the immune system and improve the energy balance to reduce the risk of developing chronic and infectious diseases [17]. Furthermore, disturbed sleep patterns due to the change in working hours and daily working patterns may reduce light-based signals for wakefulness and sleep, which is necessary for our daily rhythm (circadian). It has affected and disturbed sleeping patterns which may result in developing insomnia. As a result, falling asleep delayed seven to eight hours every night and waking up on time became more difficult. In turn, this leads to drowsiness, irritation, and lack of focus during the day [18, 19]. Sleep is important for physical health and the effective functioning of the immune system. It promotes emotional wellness and mental health and helps to overcome stress, depression, and anxiety. Millions of people suffered from insomnia before the coronavirus, but the pandemic has resulted in many new challenges, affecting people who did not had trouble sleeping before. Excessive exposure to a screen, especially in the late evening, can have a detrimental effect on sleep. This is probably because of the blue light projected from the screens in which affects the natural production of melatonin, a hormone the body produces to help us sleep [20–22].

The WHO defines health as being not only disease-free, but rather as a state of physical, mental, spiritual, and social integration. Therefore, the importance of mental wellbeing, PA, healthy sleep, and nutrition during a pandemic and its consequences on these variables should be emphasized and explored This study aims to determine the extent to which lifestyle behaviors such as PA, sleep, and diet contributed to mental wellbeing during the COVID-19 pandemic confinement. Consequently, we hypothesized that adequate PA, good diet quality, and good sleep would be linked to better mental wellbeing.

## Materials and methods

### Study design and participant recruitment

A cross-sectional comparable design using a snowball nondiscriminatory sampling procedure was used during the study period from 17^th^–24^th^, April 2020. An online Arabic questionnaire was sent to universities in the Middle Eastern and North Africa (MENA) region. Consenting Arab adults aged 18 to 65 years who complying with government guidelines of home confinement and isolation were recruited electronically. The questionnaire was designed using an online Google form to collect information about demographic, dietary, physical activity, sleep, and mental wellbeing variables. A link to the questionnaire was circulated via email, WhatsApp, Facebook, Twitter, and LinkedIn. Participants were informed about the study objectives, and only participants who provided informed consent form (online) have completed the questionnaire (which lasted 10 min on average) and submitted it online. The questionnaire did not seek personal information (name, email, date of birth) that could be used to identify the participant; therefore, their identity remained anonymous. No compensation was offered to the participants who completed the questionnaire. Ethical approval conforming to the Declaration of Helsinki was obtained from the Human Research Ethics Committees of the University of Jordan and the Hashemite University. All consented participants filled the online questionnaire. The study questionnaire was tested on 63 pilot participants who completed the survey on two occasions separated by a period ranging from one to two weeks to test for internal and external reliability and the clarity of the questions. The data from the pilot group was not included in the final analysis. The study questionnaire showed an adequate internal reliability (Cronbach α > 0.70) and an external reliability (Intraclass Correlation Coefficient > 0.70) for total and all the individual scales.

Overall, self-reported information from 1807 participants was obtained. Records with incomplete and no rational information (data outside the 95% confidence interval) were excluded. Therefore, data was obtained from 1723 participants for the present study. The responses included 1063 (67%) from the Levant region (Jordan, Lebanon, Palestine, and Syria), 442 (25.7%) from the Arab Gulf region (Bahrain, Iraq, Kuwait, Oman, Qatar, Saudi Arabia, and the United Arab Emirates), 119 (6.9%) from the North Africa region (Algeria, Egypt, Libya, Morocco, and Tunisia), and 99 (5.7%) from countries outside these regions (Yemen and Sudan). Participants were mainly students, staff, and faculty members of varied universities in the Middle East and North Africa (MENA) region.

### Demographics, mental wellbeing, dietary behavior, physical activity, and sleep data

Participants completed an online questionnaire composed of multiple scales validated and adopted to be used for the Arab population. The questionnaire sub-scales included the Demographic and Cultural Information (DCI), the World Health Organization-Five Well-Being Index (WHO-5) [22], Food Frequency Questionnaire (FFQ) [23], Pittsburg Sleep Quality Index (PSQI) [24], and Short-Form International Physical Activity Questionnaire (IPAQ) [25]. Data on demographics (age, gender, weight, height, education level, marital status, health status, smoking status, country, housing, occupation, and presence of chronic diseases) were gathered through a self-completed DCI. Body Mass Index (BMI) was calculated from the self-reported weight (kg) and height (cm). The BMI values were used to classify participants into underweight, normal, overweight, or obese categories [26]. Mental wellbeing was assessed through the WHO-5. The WHO-5 consists of five items, which were scored as previously described [22]. Scores from the items were summed up to generate a total score with a maximum of 25 points. Participants with a WHO-5 total score of >13 were recognized as having good mental wellbeing [22].

Dietary behavior was assessed using a qualitative FFQ. The FFQ includes 11 questions, which provided information on the frequency of consumption of healthy and unhealthy dietary components in the last week. Healthy dietary behavior was assessed by the following meals and food items; the consumption of breakfast, vegetables, fruits, dairy, herbs, and nuts. additionally, unhealthy dietary behavior was assessed by the following items; the consumption of preserved foods, sweetened beverages, fried foods, sweets, and energy drinks. Healthy dietary behavior items were rated on a scale of 0 to 4. The higher the score, the more frequent the healthy dietary behavior item has occurred, in the previous week. Conversely, unhealthy dietary behavior items were reverse-scored. The higher the score, the less frequent that unhealthy dietary behavior item has occurred in the previous week. Scores from the individual items were summed to generate a total dietary score of a maximum of 44 points. The total dietary score was categorized into two groups: low and high. The low and high groups were established according to the median split of the total dietary score (mean ± standard deviation = 28.7 ± 5.6; median = 29.0).

The quality of sleep was assessed through the Pittsburg Sleep Quality Index (PSQI). The PSQI scale includes 19 questions. Data from the 19 questions were used to generate seven components. The components were scored individually on a scale of 0 to 3 as described elsewhere [24]. The seven components’ scores were summed to generate a total score with a maximum of 21 points, with higher scores indicating poor sleep quality. Participants with a total PSQI score of <5 were identified as having good sleep quality, as described elsewhere [24].

The PA level for each participant was assessed using data obtained from the completed short form IPAQ. The short form IPAQ consists of seven items that provide information about walking, moderate PA, and vigorous PA categorized as per metabolic equivalents (MET) minutes per week. In addition, the instrument provides information about sitting time. The MET minutes per week were used to categorize participants’ PA into low, moderate, or high PA, as described elsewhere [25].

### Statistical analysis

All analyses were conducted using SPSS Statistics version 23 (IBM, Chicago, IL, USA). Data from continuous variables are presented as means (standard deviation). Data from categorical variables are presented as percentages. Significant differences were elucidated with the use of independent samples t-test, one-way ANOVA (followed by Tukey’s post hoc test) for the continuous variables, and the Chi-Square test was used for the categorical variables. A two-stage hierarchical multiple regression was performed to assess the association of mental wellbeing (dependent variable) with dietary quality, sleep quality, and PA after controlling for the influence of age, gender, BMI, and health status. Statistical significance was set as a p-value<0.05.

## Results and discussion

The demographic characteristics of the 1723 participants (806 female, 917 male) who participated in this study questionnaire are presented in Table 1. Overall, 17.5% (n = 301) of participants were obese, and 83.3% of study participants were living in urban areas. In addition, female participants were significantly younger than male participants (P<0.0001). The prevalence of overweight and obesity, regular smoking and chronic diseases such as hypertension and diabetics were significantly higher in males than in females (both P<0.0001). Also, significantly higher levels of education, physical activity, and mental wellbeing were observed in males than in females (all P<0.0001).

**Table 1:** Participants’ characteristics based on gender.

Variable	Total	Male	Female
	n (%)	n (%)	n (%)
Participants	1723 (100)	917 (53.2)	806 (46.8)
Body Mass Index (kg/m^2^)			
Mean ± SD^a^	25.8 ± 4.5	26.7 ± 4.2	24.7 ± 4.6
Underweight	58 (3.4)	14 (1.5)	44 (5.5)
Normal	778 (45.2)	338 (36.9)	440 (54.6)
Overweight	586 (34.0)	373 (40.7)	213 (26.4)
Obese	301 (17.5)	192 (20.9)	109 (13.5)
Age Group (years)			
Mean ± SD^a^	34.9 ± 12.8	37.4 ± 13.4	32.2 ± 11.5
18-23(25 percentile)	468 (27.2)	187 (20.4)	281 (34.9)
24–33 (26–50 percentile)	397 (23.0)	209 (22.8)	188 (23.3)
34–44 (51–75 percentile)	437 (25.4)	231 (25.2)	206 (25.6)
45 or more (>75)	421 (24.4)	290 (31.6)	131 (16.3)
Education Level			
School	340 (19.7)	156 (17.0)	184 (22.8)
Bachelor/College	941 (54.6)	488 (53.2)	453 (56.2)
Master/Doctorate	442 (25.7)	273 (29.8)	169 (21.0)
Housing			
Urban	1436 (83.3)	758 (82.7)	678 (84.1)
Rural	287 (16.7)	159 (17.3)	128 (15.9)
Smoking			
No	1249 (72.5)	574 (62.6)	675 (83.7)
Yes	474 (27.5)	343 (37.4)	131 (16.3)
Chronic Disease (hypertension and diabetes			
No	1252 (72.7)	598 (65.2)	654 (81.1)
Yes	471 (27.3)	319 (34.8)	152 (18.9)
Health Status			
Poor/Fair	169 (9.8)	85 (9.3)	84 (10.4)
Good	471 (27.3)	255 (27.8)	216 (26.8)
Very Good/excellent	1083 (62.9)	577 (62.9)	506 (62.8)
Marital Status			
Single	711 (41.3)	332 (36.2)	379 (47.0)
Married	947 (55.0)	549 (59.9)	398 (49.4)
Divorced	65 (3.8)	36 (3.9)	29 (3.6)

^a^SD: Standard Deviation

Table 2 reports the association of mental wellbeing with lifestyle variables and with some demographic variables. Overall, 67.4% of participants showed a good level of mental wellbeing. Male participants were more likely to have good mental wellbeing in comparison with female participants (*p*<0.0001). In general, participants with good mental wellbeing were more likely to have a good quality of sleep (*p*<0.0001), significantly higher levels of self-reported health (*p* <0.0001), physical activity (*p*<0.0001), education (*p* = 0.037), and dietary quality score (*p* <0.0001). However, no statistical significant association was found between the level of mental wellbeing with age, body weight, geographical location, marital status and smoking.

**Table 2:** Association of mental wellbeing according to lifestyle variables and selected demographic variables.

Variable	Mental Wellbeing Status		Chi-Square	
	No (<13)	Yes (≥13)	χ2	P-value
	n (%)	n (%)		
Participants	562 (32.6)	1161 (67.4)		
Gender				
Female	303 (53.9)	503 (43.3)	17.058	<0.0001
Male	259 (46.1)	658 (56.7)		
Body Mass Index (kg/m^2^)				
Underweight	23 (4.1)	35 (3.0)	2.812	0.422
Normal	253 (45.0)	525 (45.2)		
Overweight	181 (32.2)	405 (34.9)		
Obese	105 (18.7)	196 (16.9)		
Age Group (years)				
18-23(25 percentile)	166 (29.5)	302 (26.0)	3.391	0.335
24–33 (26–50 percentile)	119 (21.2)	278 (23.9)		
34–44 (51–75 percentile)	137 (24.4)	300 (25.8)		
45 or more (>75)	140 (24.9)	281 (24.2)		
Education Level				
School	112 (19.9)	228 (19.6)	6.577	0.037
Bachelor/College	327 (58.2)	614 (52.9)		
Master/Doctorate	123 (21.9)	319 (27.5)		
Housing				
Urban	477 (84.9)	959 (82.6)	1.411	0.235
Rural	85 (15.1)	202 (17.4)		
Smoking				
No	395 (70.3)	854 (73.6)	2.034	0.154
Yes	167 (29.7)	307 (26.4)		
Health Status				
Poor/Fair	89 (15.8)	80 (6.9)	97.672	<0.0001
Good	211 (37.5)	260 (22.4)		
Very Good/Excellent	262 (46.6)	821 (70.7)		
Marital Status				
Single	230 (40.9)	481 (41.4)	0.074	0.964
Married	310 (55.2)	637 (54.9)		
Divorced	22 (3.9%)	43 (3.7)		
Sleep Status				
Poor > 5	383 (68.1)	353 (30.4)	220.480	<0.0001
Good < 5	179 (31.9)	808 (69.6)		
Physical Activity Status (MET.min.week)				
Low	540 (96.1)	721 (62.1)	226.105	<0.0001
Moderate	22 (3.9)	253 (21.8)		
High	0 (0.0)	187 (16.1)		
Dietary Quality Score				
Low	382 (68.0)	558 (48.1)	60.546	<0.0001
High	180 (32.0)	603 (51.9)		

**Fig 1:**
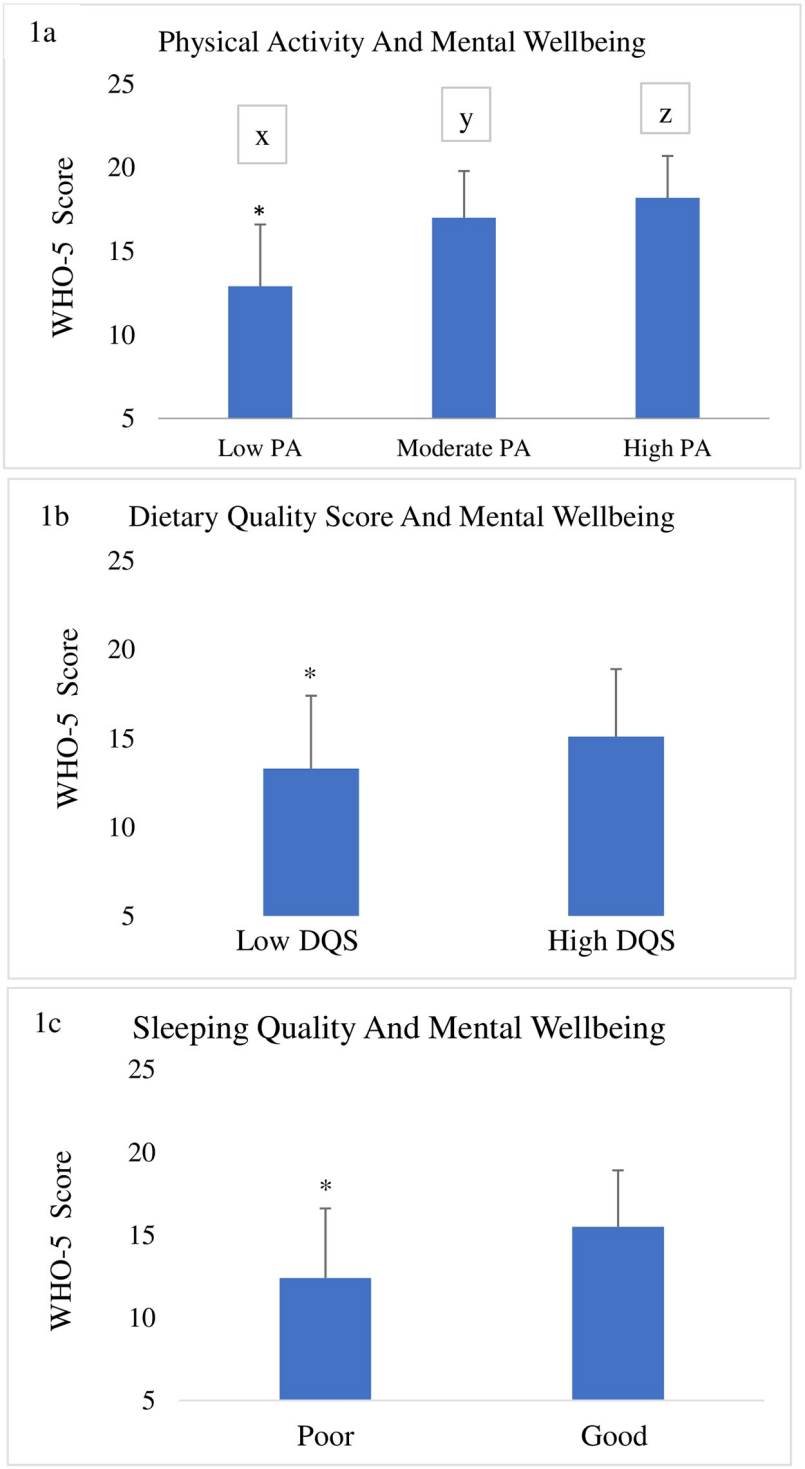
**a.** Mental health score of participants with varied levels of physical activity (low, moderate and high). Bars with different letters are significantly different using one-way analysis of variance (ANOVA). **b.** Mental health score of participants with poor or good dietary quality score p˂0.001 using T-test). **c.** Mental health score of participants with poor or good sleep quality score.

Fig 1a shows the ANOVA results for the mental wellbeing score as a dependent variable by PA level (low, moderate, and high). The ANOVA revealed a significant main effect for PA level (F (2, 1722) = 291.595, p< 0.001), with high PA (18.2 ± 2.5) showing a significantly higher overall mental wellbeing score than moderate PA (17.0 ± 2.8; p < 0.001), and low PA (12.9 ± 3.7; p < 0.001). Further, moderate PA was significantly higher than low PA (p < 0.001).

Fig 1b. shows the results of the t-test comparing the scores of the WHO-5 between low and high dietary quality scores. It is important to point out that more respondents reported low dietary quality (55%) than high dietary quality (45%). Nevertheless, significant differences were observed (t = -9.674, p < 0.001), indicating that a high dietary quality score produced better overall mental wellbeing than low dietary quality score (15.1 ± 3.8 and 13.3 ± 4.1 respectively) (*p*< .001).

In Fig 1c, we compare the means of overall mental wellbeing across different levels of sleep quality (poor and good). The t-test analysis revealed a significant difference in mental wellbeing score based on sleep quality (t = -16.413, p < 0.001). It is noteworthy that a significant number of respondents (43%) reported poor sleep quality. Those with good sleep quality showed significantly better mental wellbeing (15.5 ± 3.4) in comparison with those with poor sleep quality (12.4 ± 4.2) (*p* < .001).

A two-stage stepwise multiple regression analysis was conducted (Table 3) to assess the association of lifestyle behaviors with mental wellbeing after controlling for the influence of age, BMI, gender, and health status. In the first model (step 1), four variables including age, BMI, gender, and health status were assessed. All had well predictions of mental wellbeing (p<0.05) except for BMI (*p* = 0.153). The four variables in step 1 explained 9% (F (4, 1717) = 44.480, *p* < 0.001) of the variance in the mental wellbeing score with health status being the best predictor of mental wellbeing (beta = 0.276, p < .001). In step 2, three additional variables were added as predictors of wellbeing: dietary score, sleep score, and PA. The added variables explained an additional 18% of the variance in the mental wellbeing score (*p* < 0.001) over and above what the first four variables explained. Overall, the seven variables explained about 27% (F (7, 1714) = 93.645, *p* < 0.001) of the variance in the mental wellbeing score. However, when all of the seven variables were in the final model, two variables failed to predict mental wellbeing score: age (p = 0.132) and BMI (p = 0.723). Physical activity (beta = 0.348, p < .001, r = 0.427) was the best predictor of mental wellbeing score, followed by health status (beta = 0.194, *p <* .001, r = 0.277). That is, individuals with higher levels of PA and better health status had higher mental wellbeing scores. Similarly, those who reported higher diet scores also reported a higher mental wellbeing score (beta = 0.165, p < .001, r = 0.281). Furthermore, higher sleep scores were also associated with higher mental wellbeing scores (beta = -0.129, p < .001, r = -0.167). Gender was also a good predictor of mental wellbeing as males reported higher mental wellbeing scores (beta = 0.044, p = 0.047, r = 0.128) than females did. These results provide support to the descriptive results in Fig 1.

**Table 3:** Step wise multiple regression to assess predictors of mental health.

Model		Unstandardized		Standardized	T	Sig.	95.0% C.I. for B		
		B	S.E.	Beta			Lower	Upper	R
1	(Constant)	37.765	2.754		13.714	.000	32.364	43.166	
	Age (Years)	.066	.033	.052	2.003	.045	.001	.131	.031
	BMI (kg/m^2^)	-.134	.093	-.037	-1.431	.153	-.317	.050	-.014
	Gender	3.898	.770	.120	5.061	.000	2.387	5.409	.128
	Health Status	4.800	.403	.276	11.905	.000	4.010	5.591	.277
2	(Constant)	2.601	3.163		.822	.411	-3.603	8.805	
	Age (Years)	.047	.031	.037	1.508	.132	-.014	.109	.031
	BMI (kg/m2)	.030	.084	.008	.354	.723	-.135	.195	-.014
	Gender	1.416	.714	.044	1.983	.047	.016	2.817	.128
	Health Status	3.380	.367	.194	9.208	.000	2.660	4.100	.277
	Dietary Score	.478	.066	.165	7.259	.000	.349	.607	.281
	Sleep score	-.330	.053	-.129	-6.233	.000	-.434	-.226	-.167
	Physical Activity	9.568	.623	.348	15.351	.000	8.346	10.791	.427

## Discussion

This study investigated the influence of home confinement during the COVID-19 pandemic on different dimensions of mental wellbeing and lifestyle behavior. The responses obtained from a cluster of the sample included 1063 (67%) from the Levant, 442 (25.7%) from the Arab Gulf, 119 (6.9%) from North Africa, and the other 99 (5.7%) showed virtuous results during the confinement.

Home confinement and curfew forced most people to work or study at home, a practice that is believed to compromise routine PA by increasing the sitting time. Indeed, many people are expected to spend extended periods of time in front of a screen, either checking news on the phone, joining online studies or meetings, or watching livestream movies, which could result in a more of a sedentary lifestyle. In addition to the alteration in daily practices, confinement was reported to impact mental health. People and medical workers quarantined during the MERS outbreak in 2005 reported experiencing anxiety symptoms and anger issues up to four to six months after the end of the quarantine, and in some cases, a need for psychological help was reported [27]. Due to the COVID-19 pandemic and its associated confinement, increased levels of psychological problems such as anxiety, depression, and poor sleep quality were found in quarantined Chinese sample populations [20–22]. Furthermore, a recent review concluded that quarantine deteriorates peoples’ mental health, causing negative psychological effects, including post-traumatic stress symptoms (PTSS) [28, 29].

The impact of stress on mental health was reported to be affected by gender [30] which is expected, in part, to be related to cultural differences. Gender determines the amount of power and control men acquire over women in some societies and the socio-economic factors affecting their mental health and lives, such as their social status, the way they are perceived and treated in their society, and their vulnerability to specific mental health risks. The conditions during a pandemic, have forced men to go out on foot for home shopping while the women remained at home to cook and take care of children, this has also been a cause of some of their mental health challenges [30, 31]. It can be suggested that such cultural practices influenced the observed differences in mental wellbeing between male and female participants. This can be explained by the fact that most of the responses came from urban areas, and many participants live in apartments that do not allow freedom of movement. For example, they cannot perform gardening activities such as weeding, mowing, and cleaning. The gender difference was reported to be a significant predictor of PTSS [30].

To further improve the understanding of the home confinement’s effect on people’s health, we investigated the association between PA levels, dietary behavior, sleep quality and the magnitude of the COVID-19 pandemic confinement’s effect on mental wellbeing. Table 3 showed that PA was the best predictor of mental health, followed by health status. That is, higher levels of PA and better health status, higher mental wellbeing score were reported in the current study. Those who reported a better diet also reported better mental health. Furthermore, good sleep scores were associated with better mental health. Gender was also a significant factor in mental health as males reported better mental health than females. These results provide evidence that supports our descriptive results in Fig 1a–1c. In the present study, a positive association between PA and mental wellbeing was determined. It may suggest specifically that the lack of mobility and the shutdown have affected physical activity and the mental wellbeing of the subjects [32]. In line with other studies [33–36], mental health was found to be improved with PA.

It is well-known that active individuals always have a better mood which is influenced by the release of certain hormones (endorphins and serotonin). The secretion of endorphins during physical exercise leads to a sense of euphoria, modifies appetite, and boosts the response of the immune system. During the time of involvement into high levels of PA, the body releases endorphins which interact with the receptors in the brain resulting in the reduction of the perception of pain, thus inducing relaxation and reducing stress [37]. Serotonin which is produced in the intestine promotes healthy digestion and helps with sleep, it also helps with mood regulation in the brain [38, 39]. Furthermore, it is known that the benefits of regular PA go beyond supporting the secretion of serotonin, in relieving depression and dealing with stress.

Our findings are consistent with the results of previous research conducted during the SARS epidemic and recent research in Europe [19, 40]. Not getting enough exposure to sunlight is one theory behind why people experience depression during the short, dark days of autumn and winter [41]. A new study has found that continuous exposure to ultraviolet (UV) radiation causes the release of endorphins and vitamin D3, which is essential for bone and musculoskeletal health [42–44]. A deficiency of such hormones was associated with depression. In this study, the group with low PA may feel guilty for exposing their health to sedentary lifestyle-associated risks. The overall mental wellbeing score (WHO-5) was positively influenced by PA level. This result concurs with recent research that reported a negative psycho-emotional effect of COVID-19’s home confinement on lifestyle behaviors, particularly on physically inactive people [28–30, 44].

Moreover, our results indicated that the overall mental wellbeing score (WHO-5) was higher in those who adopted a healthy diet. These findings encourage the use of healthy eating behaviors to control the psycho-emotional effect of home confinement. Bad nutritional behavior may also be stimulated by emotional eating due to confinement-induced anxiety, stress, and long sitting hours [35, 45].

Additionally, results indicate that sleep quality was strongly associated with mental wellbeing (P < .001). Having adequate hours of sleep and good quality of sleep is essential in regulating human biology and the circadian rhythm, which affects hormonal secretion and metabolism. Mood alterations and neurological factors that enhance psychological status are expected to function well with better sleep quality. Earlier studies have reported a negative impact of home confinement on sleep quality [19], which is linked to mental wellbeing, supporting our findings. In addition, the diet score of our study indicate that those who possess a balanced diet, and consume good quality food have an enhanced mental wellbeing. This is in agreement with literature stating that having a good balance of "friendly" bacteria in your intestines is linked to adequate serotonin levels [43, 46].

In brief, mental health among our participants was affected by several factors (PA, diet, and sleep), and thus a further analysis was performed to identify the magnitude of the contribution of each factor. The impact of PA (moderate and above) on mental health was by far the highest. This finding is importance on a public health level in addressing the detrimental impact of confinement on mental health. Therefore, certain interventions are needed to develop programs that focus on improving PA during confinement and other circumstances to attenuate the risk of poor mental health during confinement.

Despite being one of the few studies examining the link between PA, diet, sleep, and mental wellbeing among Arab educational institutions during the COVID-19 pandemic, this study has some limitations. Given the high number of responses examined, the detailed variables are not explained which limits our ability to compare the pre and during confinement effects of each variable. Moreover, given the nature of the comparable cross-sectional design using a snowball nondiscriminatory sampling procedure in this study, causality cannot be inferred from this analysis. Added to that, due to the nature of the surveys, the effects of bias in remembering and social culture cannot be avoided. Inclusion and exclusion criteria were not defined; however, this has been explained in the Method section.

## Conclusions

As a result of confinement due to COVID-19, individuals who were involved in more PA and who have perceived had better health and adequate sleep in terms of time and quality, and have mentally adapted better to the stress of confinement. In brief, the state of mental health during confinement was reported to be better among participants who adopted a healthy lifestyle in terms of PA, dietary, and sleep behaviors. PA was the best predictor of mental health, followed by the health status. That is, the more PA and better health individuals reported, the better mental health they expressed. Also, those who had better diets reported better mental health. Furthermore, participants with good sleep scores were possessed with better mental health. Gender was also a strong factor as males reported better mental health than females. Males and those who followed a good diet regime were better in adapting mentally to confinement. Finally, the impact of social distancing measures on physical activity, was an important determinant of health, especially if prolonged social distancing is required.

## Supporting information

S1 File (PDF)
