# Correction: Healthy lifestyle behaviors are major predictors of mental wellbeing during COVID-19 pandemic confinement: A study on adult Arabs in higher educational institutions

**DOI:** 10.1371/journal.pone.0273276

**Published:** 2022-08-15

**Authors:** Hashem A. KilaniI, Mo’ath F. Bataineh, Ali Al-Nawayseh, Khaled Atiyat, Omar Obeid, Maher M. Abu-Hilal, Taiysir Mansi, Maher Al-Kilani, Mahfoodha Al-Kitani, Majed El-Saleh, Ruba M. Jaber, Ahmad Sweidan, Mawaheb Himsi, Iyad Yousef, Faten Alzeer, Monther Nasrallah, Ayesha S. Al Dhaheri, Abdulsalam Al-Za’abi, Osama Allala, Laila Al-Kilani, Asma M. Alhasan, Mohamed Ghieda, Yasir Najah, Saad Alsheekhly, Ahmad Alhaifi, Raghda Shukri, Jamal Al Adwani, Mostafa Waly, Laila Kilani, Leen H. Kilani, Ahmad S. al Shareef, Areej Kilani

The fourteenth author’s name is spelled incorrectly. The correct name is: Iyad A. Yousef. There is also an error in affiliation 10 for author Iyad A. Yousef. The correct affiliation 10 is: Department of Physical Education, College of Education, Birzeit University, Ramallah, Palestine.
