# Correction: Healthy lifestyle behaviors are major predictors of mental wellbeing during COVID-19 pandemic confinement: A study on adult Arabs in higher educational institutions

**DOI:** 10.1371/journal.pone.0316869

**Published:** 2024-12-30

**Authors:** Hashem A. Kilani, Mo’ath F. Bataineh, Ali Al-Nawayseh, Khaled Atiyat, Omar Obeid, Maher M. Abu-Hilal, Taiysir Mansi, Maher Al-Kilani, Mahfoodha Al-Kitani, Majed El-Saleh, Ruba M. Jaber, Ahmad Sweidan, Mawaheb Himsi, Iyad Yousef, Faten Alzeer, Monther Nasrallah, Ayesha S. Al Dhaheri, Abdulsalam Al-Za’abi, Osama Allala, Laila Al-Kilani, Asma M. Alhasan, Mohamed Ghieda, Yasir Najah, Saad Alsheekhly, Ahmad Alhaifi, Raghda Shukri, Jamal Al Adwani, Mostafa Waly, Laila Kilani, Leen H. Kilani, Ahmad S. al Shareef, Areej Kilani

The Funding statement for this paper is incorrect. The correct Funding statement is as follows: This research was funded by the Deanship of Scientific Research at Princess Nourah bint Abdulrahman University through the Fast-track Research Funding Program.

There is an error in affiliation 16 for author Laila Al-Kilani. The correct affiliation 16 is: Department of Physical & Sports Sciences, College of Education, Princess Nourah bint Abdulrahman University, Riyadh, Saudi Arabia.
