# Supplementary material for: Healthy lifestyle behaviors are major predictors of mental wellbeing during COVID-19 pandemic confinement: A study on adult Arabs in higher educational institutions
Source: PLoS One. 2020 Dec 14;15(12):e0243524. doi: 10.1371/journal.pone.0243524 (PMC7735567; doi:10.1371/journal.pone.0243524)
Supplement: S1 File — (PDF) [file pone.0243524.s001.pdf]

Subject	Age	Weight	Height	BMI	BMI_C	Residence	Education	Marital_Status	Smoking	Chronic_Dise	Gender	Health	Sitting_Time	PA_Min_Day	IPAQ_C	Dietary_Score	Dietary_C	PSQI_Total	Sleep_C	WHO5_Total	WHO5_Total100	WHO5_Class
**1.00**	19.00	65.00	173.00	21.72	2.00	0.00	1.00	1.00	1.00	1.00	1.00	2.00	1.50	112.86	3.00	24.00	0.00	4.00	1.00	17.00	68.00	0.00
**2.00**	23.00	59.00	173.00	19.71	2.00	1.00	2.00	1.00	0.00	0.00	1.00	3.00	1.50	185.71	3.00	35.00	1.00	5.00	1.00	17.00	68.00	0.00
**3.00**	19.00	78.00	174.00	25.76	3.00	0.00	2.00	1.00	1.00	0.00	1.00	3.00	3.00	74.29	3.00	31.00	1.00	5.00	1.00	18.00	72.00	0.00
**4.00**	21.00	84.00	184.00	24.81	2.00	1.00	1.00	1.00	1.00	1.00	1.00	2.00	7.00	83.57	3.00	35.00	1.00	5.00	1.00	17.00	68.00	0.00
**5.00**	21.00	64.00	170.00	22.15	2.00	1.00	2.00	1.00	0.00	0.00	0.00	3.00	7.00	81.43	3.00	35.00	1.00	10.00	0.00	13.00	52.00	0.00
**6.00**	19.00	81.00	185.00	23.67	2.00	1.00	2.00	1.00	0.00	0.00	1.00	3.00	6.00	111.43	3.00	31.00	1.00	4.00	1.00	16.00	64.00	0.00
**7.00**	21.00	78.00	178.00	24.62	2.00	1.00	2.00	1.00	0.00	0.00	1.00	3.00	8.00	171.43	3.00	36.00	1.00	4.00	1.00	17.00	68.00	0.00
**8.00**	23.00	85.00	193.00	22.82	2.00	1.00	2.00	1.00	1.00	1.00	1.00	2.00	7.00	154.29	3.00	19.00	0.00	6.00	0.00	19.00	76.00	0.00
**9.00**	23.00	65.00	172.00	21.97	2.00	0.00	2.00	1.00	0.00	0.00	1.00	3.00	4.50	97.14	3.00	26.00	0.00	4.00	1.00	16.00	64.00	0.00
**10.00**	22.00	43.00	155.00	17.90	1.00	1.00	1.00	1.00	0.00	1.00	0.00	3.00	6.00	81.43	3.00	31.00	1.00	12.00	0.00	18.00	72.00	0.00
**11.00**	22.00	45.00	156.00	18.49	1.00	1.00	2.00	1.00	0.00	0.00	0.00	3.00	8.00	60.00	3.00	24.00	0.00	4.00	1.00	15.00	60.00	0.00
**12.00**	23.00	66.00	161.00	25.46	3.00	1.00	2.00	1.00	0.00	0.00	0.00	3.00	4.50	120.00	3.00	19.00	0.00	8.00	0.00	13.00	52.00	0.00
**13.00**	22.00	48.00	161.00	18.52	2.00	1.00	2.00	1.00	0.00	0.00	1.00	3.00	3.00	128.57	3.00	24.00	0.00	3.00	1.00	17.00	68.00	0.00
**14.00**	21.00	71.00	180.00	21.91	2.00	0.00	2.00	1.00	0.00	0.00	1.00	3.00	4.50	115.71	3.00	32.00	1.00	5.00	1.00	17.00	68.00	0.00
**15.00**	22.00	85.00	188.00	24.05	2.00	1.00	2.00	1.00	0.00	0.00	1.00	3.00	4.00	81.43	3.00	29.00	0.00	4.00	1.00	17.00	68.00	0.00
**16.00**	21.00	50.00	159.00	19.78	2.00	1.00	1.00	1.00	1.00	0.00	0.00	1.00	7.00	80.00	3.00	21.00	0.00	8.00	0.00	16.00	64.00	0.00
**17.00**	20.00	78.00	178.00	24.62	2.00	0.00	1.00	1.00	0.00	0.00	1.00	3.00	7.00	120.00	3.00	17.00	0.00	3.00	1.00	18.00	72.00	0.00
**18.00**	18.00	76.00	180.00	23.46	2.00	1.00	1.00	1.00	0.00	0.00	1.00	3.00	2.50	154.29	3.00	36.00	1.00	3.00	1.00	19.00	76.00	0.00
**19.00**	20.00	70.00	175.00	22.86	2.00	1.00	2.00	1.00	1.00	1.00	1.00	3.00	6.50	101.43	3.00	30.00	1.00	5.00	1.00	17.00	68.00	0.00
**20.00**	21.00	79.00	183.00	23.59	2.00	0.00	2.00	1.00	1.00	0.00	1.00	3.00	6.50	128.57	3.00	33.00	1.00	2.00	1.00	17.00	68.00	0.00
**21.00**	23.00	83.00	184.00	24.52	2.00	1.00	2.00	1.00	1.00	0.00	1.00	3.00	3.50	330.00	3.00	32.00	1.00	2.00	1.00	18.00	72.00	0.00
**22.00**	20.00	60.00	152.00	25.97	3.00	1.00	1.00	1.00	0.00	0.00	0.00	3.00	6.00	122.14	3.00	35.00	1.00	1.00	1.00	18.00	72.00	0.00
**23.00**	21.00	72.00	167.00	25.82	3.00	1.00	1.00	1.00	1.00	0.00	0.00	1.00	6.00	111.43	3.00	31.00	1.00	4.00	1.00	14.00	56.00	0.00
**24.00**	18.00	65.00	150.00	28.89	3.00	1.00	1.00	1.00	0.00	0.00	0.00	3.00	6.00	132.86	3.00	38.00	1.00	5.00	1.00	19.00	76.00	0.00
**25.00**	21.00	79.00	170.00	27.34	3.00	1.00	1.00	1.00	0.00	0.00	1.00	3.00	6.00	154.29	3.00	34.00	1.00	5.00	1.00	18.00	72.00	0.00
**26.00**	20.00	92.00	180.00	28.40	3.00	1.00	1.00	1.00	0.00	0.00	1.00	3.00	6.00	115.71	3.00	32.00	1.00	4.00	1.00	22.00	88.00	0.00
**27.00**	19.00	65.00	160.00	25.39	3.00	0.00	2.00	1.00	0.00	0.00	0.00	3.00	6.50	94.00	3.00	24.00	0.00	6.00	0.00	17.00	68.00	0.00
**28.00**	21.00	75.00	160.00	29.30	3.00	1.00	2.00	1.00	0.00	0.00	0.00	3.00	6.50	225.00	3.00	31.00	1.00	3.00	1.00	22.00	88.00	0.00
**29.00**	20.00	79.00	175.00	25.80	3.00	1.00	2.00	1.00	0.00	0.00	1.00	3.00	4.50	218.57	3.00	25.00	0.00	5.00	1.00	21.00	84.00	0.00
**30.00**	19.00	83.00	178.00	26.20	3.00	1.00	2.00	1.00	0.00	1.00	1.00	2.00	9.00	88.57	3.00	33.00	1.00	6.00	0.00	20.00	80.00	0.00
**31.00**	22.00	58.00	157.00	23.53	2.00	1.00	1.00	2.00	0.00	0.00	0.00	1.00	4.00	84.29	3.00	27.00	0.00	13.00	0.00	16.00	64.00	0.00
**32.00**	21.00	53.00	158.00	21.23	2.00	0.00	1.00	1.00	0.00	0.00	0.00	1.00	5.00	156.43	3.00	30.00	1.00	4.00	1.00	18.00	72.00	0.00
**33.00**	20.00	61.00	170.00	21.11	2.00	1.00	1.00	1.00	0.00	0.00	0.00	2.00	5.50	77.14	3.00	32.00	1.00	6.00	0.00	16.00	64.00	0.00
**34.00**	23.00	55.00	164.00	20.45	2.00	1.00	1.00	1.00	0.00	0.00	0.00	2.00	8.00	85.71	3.00	26.00	0.00	8.00	0.00	20.00	80.00	0.00
**35.00**	20.00	72.00	182.00	21.74	2.00	1.00	1.00	1.00	0.00	0.00	1.00	3.00	10.00	173.57	3.00	20.00	0.00	10.00	0.00	16.00	64.00	0.00
**36.00**	18.00	68.00	169.00	23.81	2.00	0.00	1.00	1.00	0.00	0.00	1.00	3.00	8.00	162.86	3.00	28.00	0.00	6.00	0.00	21.00	84.00	0.00
**37.00**	19.00	70.00	178.00	22.09	2.00	1.00	1.00	1.00	0.00	0.00	1.00	3.00	7.00	128.57	3.00	28.00	0.00	3.00	1.00	17.00	68.00	0.00
**38.00**	20.00	75.00	180.00	23.15	2.00	0.00	1.00	1.00	0.00	0.00	1.00	3.00	11.00	142.14	3.00	25.00	0.00	7.00	0.00	15.00	60.00	0.00
**39.00**	22.00	78.00	187.00	22.31	2.00	0.00	1.00	1.00	0.00	0.00	1.00	3.00	9.00	177.86	3.00	23.00	0.00	4.00	1.00	20.00	80.00	0.00
**40.00**	20.00	65.00	175.00	21.22	2.00	1.00	1.00	1.00	0.00	0.00	0.00	3.00	3.50	141.43	3.00	32.00	1.00	5.00	1.00	22.00	88.00	0.00
**41.00**	19.00	59.00	160.00	23.05	2.00	1.00	1.00	1.00	0.00	0.00	0.00	3.00	4.50	98.57	3.00	32.00	1.00	11.00	0.00	18.00	72.00	0.00
**42.00**	18.00	54.00	165.00	19.83	2.00	1.00	1.00	1.00	0.00	0.00	0.00	3.00	6.00	122.86	3.00	28.00	0.00	0.00	1.00	17.00	68.00	0.00
**43.00**	20.00	77.00	184.00	22.74	2.00	1.00	1.00	1.00	0.00	0.00	1.00	3.00	10.00	90.00	3.00	21.00	0.00	4.00	1.00	20.00	80.00	0.00
**44.00**	19.00	68.00	170.00	23.53	2.00	1.00	1.00	1.00	0.00	0.00	1.00	3.00	7.00	78.57	3.00	32.00	1.00	2.00	1.00	17.00	68.00	0.00
**45.00**	21.00	63.00	164.00	23.42	2.00	0.00	1.00	1.00	0.00	0.00	1.00	3.00	3.00	142.86	3.00	30.00	1.00	86.21	0.00	17.00	68.00	0.00
**46.00**	20.00	71.00	173.00	23.72	2.00	1.00	1.00	1.00	0.00	0.00	1.00	3.00	3.50	91.86	3.00	28.00	0.00	6.00	0.00	18.00	72.00	0.00
**47.00**	23.00	76.00	180.00	23.46	2.00	1.00	1.00	1.00	0.00	0.00	1.00	3.00	5.00	91.43	3.00	37.00	1.00	4.00	1.00	23.00	92.00	0.00
**48.00**	20.00	73.00	176.00	23.57	2.00	1.00	1.00	1.00	1.00	1.00	1.00	1.00	6.00	128.57	3.00	25.00	0.00	1.00	1.00	21.00	84.00	0.00
**49.00**	19.00	75.00	180.00	23.15	2.00	1.00	1.00	1.00	1.00	1.00	1.00	1.00	7.00	157.86	3.00	37.00	1.00	8.00	0.00	21.00	84.00	0.00
**50.00**	22.00	68.00	168.00	24.09	2.00	1.00	1.00	1.00	0.00	1.00	1.00	2.00	9.00	94.29	3.00	26.00	0.00	7.00	0.00	13.00	52.00	0.00
**51.00**	23.00	73.00	182.00	22.04	2.00	1.00	1.00	1.00	0.00	1.00	1.00	2.00	8.00	77.14	3.00	29.00	0.00	4.00	1.00	20.00	80.00	0.00
**52.00**	23.00	81.00	180.00	25.00	2.00	0.00	1.00	1.00	0.00	1.00	1.00	3.00	5.00	61.43	3.00	35.00	1.00	7.00	0.00	17.00	68.00	0.00
**53.00**	20.00	50.00	162.00	19.05	2.00	0.00	2.00	1.00	0.00	0.00	0.00	2.00	8.00	185.71	3.00	25.00	0.00	8.00	0.00	17.00	68.00	0.00
**54.00**	20.00	58.00	173.00	19.38	2.00	0.00	2.00	1.00	1.00	0.00	1.00	2.00	7.00	147.14	3.00	36.00	1.00	3.00	1.00	17.00	68.00	0.00
**55.00**	23.00	64.00	167.00	22.95	2.00	1.00	2.00	1.00	0.00	0.00	0.00	2.00	8.00	154.29	3.00	24.00	0.00	6.00	0.00	17.00	68.00	0.00
**56.00**	21.00	68.00	175.00	22.20	2.00	0.00	2.00	1.00	1.00	0.00	1.00	3.00	8.00	87.14	3.00	22.00	0.00	7.00	0.00	13.00	52.00	0.00
**57.00**	20.00	63.00	175.00	20.57	2.00	1.00	2.00	1.00	0.00	0.00	1.00	3.00	3.00	168.43	3.00	26.00	0.00	5.00	1.00	14.00	56.00	0.00
**58.00**	20.00	53.00	158.00	21.23	2.00	0.00	2.00	1.00	0.00	0.00	0.00	3.00	10.00	111.43	3.00	30.00	1.00	1.00	1.00	24.00	96.00	0.00
**59.00**	21.00	65.00	174.00	21.47	2.00	1.00	2.00	1.00	0.00	0.00	0.00	3.00	6.00	109.29	3.00	38.00	1.00	87.21	0.00	21.00	84.00	0.00
**60.00**	18.00	78.00	189.00	21.84	2.00	0.00	2.00	1.00	0.00	0.00	1.00	3.00	6.00	240.00	3.00	37.00	1.00	3.00	1.00	21.00	84.00	0.00
**61.00**	19.00	59.00	158.00	23.63	2.00	0.00	2.00	1.00	0.00	0.00	0.00	3.00	6.00	145.71	3.00	31.00	1.00	3.00	1.00	20.00	80.00	0.00
**62.00**	18.00	70.00	175.00	22.86	2.00	1.00	2.00	1.00	0.00	0.00	1.00	3.00	8.00	141.43	3.00	25.00	0.00	5.00	1.00	17.00	68.00	0.00
**63.00**	22.00	80.00	183.00	23.89	2.00	1.00	2.00	1.00	0.00	0.00	1.00	3.00	6.50	95.71	3.00	26.00	0.00	3.00	1.00	17.00	68.00	0.00
**64.00**	20.00	76.00	176.00	24.54	2.00	1.00	2.00	1.00	0.00	0.00	1.00	3.00	4.50	143.57	3.00	28.00	0.00	5.00	1.00	20.00	80.00	0.00
**65.00**	21.00	55.00	168.00	19.49	2.00	1.00	2.00	1.00	0.00	0.00	0.00	3.00	7.00	111.43	3.00	42.00	1.00	3.00	1.00	23.00	92.00	0.00
**66.00**	22.00	75.00	178.00	23.67	2.00	1.00	2.00	1.00	0.00	0.00	1.00	3.00	3.00	64.29	3.00	29.00	0.00	6.00	0.00	15.00	60.00	0.00

Subject	Age	Weight	Height	BMI	BMI_C	Residence	Education	Marital_Status	Smoking	Chronic_Dise	Gender	Health	Sitting_Time	PA_Min_Day	IPAQ_C	Dietary_Score	Dietary_C	PSQI_Total	Sleep_C	WHO5_Total	WHO5_Total100	WHO5_Class
67.00	20.00	49.00	170.00	16.96	1.00	1.00	1.00	2.00	0.00	0.00	0.00	3.00	8.00	240.00	3.00	31.00	1.00	4.00	1.00	17.00	68.00	0.00
68.00	20.00	60.00	185.00	17.53	1.00	0.00	2.00	1.00	1.00	1.00	1.00	3.00	10.00	298.57	3.00	23.00	0.00	3.00	1.00	17.00	68.00	0.00
69.00	22.00	56.00	176.00	18.08	1.00	1.00	2.00	1.00	1.00	0.00	1.00	3.00	6.50	112.86	3.00	18.00	0.00	3.00	1.00	21.00	84.00	0.00
70.00	23.00	57.00	180.00	17.59	1.00	1.00	2.00	1.00	1.00	0.00	1.00	3.00	4.50	128.57	3.00	23.00	0.00	3.00	1.00	17.00	68.00	0.00
71.00	19.00	71.00	167.00	25.46	3.00	0.00	2.00	1.00	1.00	0.00	0.00	1.00	8.00	160.71	3.00	23.00	0.00	5.00	1.00	17.00	68.00	0.00
72.00	22.00	54.00	160.00	21.09	2.00	1.00	1.00	1.00	0.00	0.00	0.00	2.00	5.50	210.00	3.00	37.00	1.00	0.00	1.00	19.00	76.00	0.00
73.00	18.00	60.00	165.00	22.04	2.00	1.00	2.00	1.00	1.00	1.00	1.00	3.00	5.00	291.43	3.00	25.00	0.00	5.00	1.00	17.00	68.00	0.00
74.00	21.00	55.00	165.00	20.20	2.00	0.00	2.00	1.00	0.00	0.00	0.00	3.00	6.50	102.86	3.00	30.00	1.00	12.00	0.00	13.00	52.00	0.00
75.00	21.00	60.00	162.00	22.86	2.00	1.00	2.00	1.00	0.00	0.00	0.00	3.00	6.00	140.71	3.00	37.00	1.00	2.00	1.00	17.00	68.00	0.00
76.00	20.00	64.00	165.00	23.51	2.00	1.00	2.00	1.00	0.00	0.00	0.00	3.00	4.00	122.86	3.00	42.00	1.00	10.00	0.00	17.00	68.00	0.00
77.00	28.00	73.00	183.00	21.80	2.00	1.00	2.00	1.00	1.00	1.00	1.00	2.00	5.00	170.71	3.00	25.00	0.00	8.00	0.00	17.00	68.00	0.00
78.00	26.00	75.00	182.00	22.64	2.00	1.00	2.00	1.00	0.00	0.00	1.00	3.00	7.00	145.71	3.00	15.00	0.00	9.00	0.00	21.00	84.00	0.00
79.00	29.00	84.00	183.00	25.08	3.00	1.00	1.00	2.00	0.00	0.00	1.00	3.00	4.00	162.86	3.00	29.00	0.00	1.00	1.00	18.00	72.00	0.00
80.00	33.00	75.00	172.00	25.35	3.00	1.00	2.00	1.00	0.00	0.00	0.00	3.00	8.00	102.86	3.00	38.00	1.00	7.00	0.00	15.00	60.00	0.00
81.00	31.00	86.00	175.00	28.08	3.00	1.00	2.00	2.00	1.00	0.00	1.00	2.00	4.00	110.00	3.00	31.00	1.00	5.00	1.00	16.00	64.00	0.00
82.00	24.00	100.00	184.00	29.54	3.00	1.00	2.00	1.00	0.00	0.00	1.00	3.00	7.00	98.57	3.00	21.00	0.00	11.00	0.00	14.00	56.00	0.00
83.00	28.00	84.00	180.00	25.93	3.00	1.00	2.00	1.00	1.00	0.00	1.00	3.00	4.00	111.43	3.00	25.00	0.00	6.00	0.00	16.00	64.00	0.00
84.00	33.00	80.00	179.00	24.97	2.00	1.00	2.00	2.00	1.00	0.00	1.00	2.00	8.00	160.00	3.00	24.00	0.00	7.00	0.00	15.00	60.00	0.00
85.00	24.00	68.00	180.00	20.99	2.00	1.00	2.00	1.00	0.00	0.00	0.00	2.00	4.00	295.71	3.00	33.00	1.00	4.00	1.00	23.00	92.00	0.00
86.00	25.00	57.00	165.00	20.94	2.00	1.00	2.00	1.00	0.00	0.00	0.00	3.00	4.50	190.00	3.00	32.00	1.00	3.00	1.00	17.00	68.00	0.00
87.00	29.00	50.00	157.00	20.28	2.00	1.00	3.00	1.00	0.00	0.00	0.00	3.00	5.00	140.00	3.00	34.00	1.00	9.00	0.00	19.00	76.00	0.00
88.00	30.00	70.00	168.00	24.80	2.00	1.00	3.00	2.00	0.00	0.00	1.00	3.00	9.00	77.14	3.00	34.00	1.00	2.00	1.00	21.00	84.00	0.00
89.00	26.00	75.00	176.00	24.21	2.00	1.00	2.00	1.00	1.00	0.00	1.00	3.00	3.50	139.29	3.00	33.00	1.00	3.00	1.00	20.00	80.00	0.00
90.00	25.00	70.00	172.00	23.66	2.00	1.00	2.00	1.00	0.00	0.00	1.00	3.00	3.50	175.71	3.00	31.00	1.00	3.00	1.00	18.00	72.00	0.00
91.00	30.00	97.00	175.00	31.67	4.00	1.00	2.00	2.00	0.00	0.00	1.00	2.00	5.00	77.14	3.00	24.00	0.00	5.00	1.00	17.00	68.00	0.00
92.00	30.00	72.00	166.00	26.13	3.00	1.00	2.00	1.00	1.00	0.00	1.00	2.00	4.00	570.00	3.00	26.00	0.00	5.00	1.00	17.00	68.00	0.00
93.00	25.00	90.00	177.00	28.73	3.00	0.00	3.00	2.00	1.00	0.00	1.00	3.00	2.50	85.71	3.00	31.00	1.00	0.00	1.00	21.00	84.00	0.00
94.00	27.00	52.00	163.00	19.57	2.00	1.00	3.00	1.00	1.00	1.00	0.00	3.00	4.00	180.00	3.00	38.00	1.00	5.00	1.00	18.00	72.00	0.00
95.00	31.00	102.00	179.00	31.83	4.00	1.00	1.00	2.00	0.00	0.00	1.00	3.00	2.00	180.00	3.00	32.00	1.00	3.00	1.00	24.00	96.00	0.00
96.00	28.00	80.00	175.00	26.12	3.00	1.00	1.00	1.00	0.00	0.00	1.00	3.00	6.00	63.57	3.00	22.00	0.00	3.00	1.00	17.00	68.00	0.00
97.00	33.00	95.00	186.00	27.46	3.00	0.00	3.00	1.00	0.00	0.00	1.00	2.00	6.50	137.14	3.00	29.00	0.00	3.00	1.00	16.00	64.00	0.00
98.00	24.00	74.00	178.00	23.36	2.00	0.00	1.00	1.00	0.00	0.00	1.00	3.00	6.50	102.86	3.00	27.00	0.00	3.00	1.00	22.00	88.00	0.00
99.00	29.00	81.00	181.00	24.72	2.00	1.00	1.00	2.00	0.00	0.00	1.00	3.00	8.00	145.71	3.00	22.00	0.00	7.00	0.00	21.00	84.00	0.00
100.00	24.00	63.00	169.00	22.06	2.00	1.00	2.00	1.00	1.00	0.00	1.00	3.00	4.00	97.14	3.00	33.00	1.00	9.00	0.00	17.00	68.00	0.00
101.00	30.00	90.00	163.00	33.87	4.00	1.00	2.00	2.00	0.00	1.00	0.00	3.00	8.00	190.00	3.00	29.00	0.00	9.00	0.00	17.00	68.00	0.00
102.00	26.00	68.00	170.00	23.53	2.00	0.00	1.00	2.00	0.00	0.00	0.00	3.00	8.00	162.86	3.00	37.00	1.00	13.00	0.00	16.00	64.00	0.00
103.00	26.00	50.00	160.00	19.53	2.00	1.00	1.00	2.00	1.00	1.00	0.00	1.00	6.50	188.57	3.00	26.00	0.00	8.00	0.00	17.00	68.00	0.00
104.00	31.00	73.00	175.00	23.84	2.00	1.00	2.00	2.00	0.00	0.00	1.00	3.00	7.00	150.00	3.00	33.00	1.00	3.00	1.00	16.00	64.00	0.00
105.00	30.00	112.00	179.00	34.96	4.00	1.00	3.00	2.00	0.00	1.00	1.00	3.00	3.00	87.14	3.00	36.00	1.00	7.00	0.00	15.00	60.00	0.00
106.00	33.00	105.00	187.00	30.03	4.00	1.00	3.00	2.00	1.00	0.00	1.00	3.00	10.00	117.14	3.00	42.00	1.00	0.00	1.00	23.00	92.00	0.00
107.00	28.00	88.40	183.00	26.40	3.00	0.00	2.00	1.00	0.00	0.00	1.00	3.00	3.00	130.71	3.00	31.00	1.00	7.00	0.00	15.00	60.00	0.00
108.00	28.00	57.00	170.00	19.72	2.00	1.00	3.00	1.00	0.00	0.00	0.00	3.00	3.50	167.14	3.00	35.00	1.00	0.00	1.00	24.00	96.00	0.00
109.00	31.00	55.00	161.00	21.22	2.00	0.00	3.00	1.00	0.00	0.00	0.00	3.00	2.00	75.71	3.00	35.00	1.00	2.00	1.00	17.00	68.00	0.00
110.00	25.00	101.00	176.00	32.61	4.00	1.00	3.00	1.00	0.00	0.00	1.00	3.00	4.50	90.00	3.00	35.00	1.00	2.00	1.00	19.00	76.00	0.00
111.00	29.00	72.00	168.00	25.51	3.00	1.00	3.00	1.00	0.00	1.00	1.00	3.00	5.00	205.71	3.00	30.00	1.00	9.00	0.00	22.00	88.00	0.00
112.00	27.00	50.00	160.00	19.53	2.00	0.00	1.00	1.00	0.00	0.00	0.00	1.00	8.00	124.29	3.00	34.00	1.00	2.00	1.00	17.00	68.00	0.00
113.00	24.00	60.00	167.00	21.51	2.00	1.00	1.00	1.00	0.00	0.00	1.00	3.00	3.00	334.29	3.00	33.00	1.00	5.00	1.00	18.00	72.00	0.00
114.00	24.00	60.00	167.00	21.51	2.00	1.00	1.00	1.00	0.00	0.00	1.00	3.00	3.00	334.29	3.00	32.00	1.00	8.00	0.00	17.00	68.00	0.00
115.00	25.00	67.00	172.00	22.65	2.00	0.00	1.00	1.00	0.00	1.00	1.00	3.00	7.00	140.00	3.00	35.00	1.00	8.00	0.00	19.00	76.00	0.00
116.00	24.00	56.00	164.00	20.82	2.00	1.00	2.00	1.00	1.00	0.00	1.00	3.00	8.00	77.14	3.00	33.00	1.00	2.00	1.00	20.00	80.00	0.00
117.00	24.00	55.00	166.00	19.96	2.00	1.00	2.00	1.00	0.00	0.00	1.00	3.00	7.00	75.71	3.00	25.00	0.00	4.00	1.00	16.00	64.00	0.00
118.00	24.00	80.00	185.00	23.37	2.00	1.00	2.00	1.00	0.00	0.00	1.00	3.00	2.50	105.71	3.00	34.00	1.00	2.00	1.00	17.00	68.00	0.00
119.00	30.00	82.00	183.00	24.49	2.00	1.00	3.00	2.00	1.00	0.00	1.00	3.00	6.00	72.14	3.00	31.00	1.00	3.00	1.00	21.00	84.00	0.00
120.00	33.00	95.00	170.00	32.87	4.00	0.00	2.00	2.00	0.00	0.00	1.00	2.00	9.00	214.29	3.00	27.00	0.00	9.00	0.00	16.00	64.00	0.00
121.00	28.00	106.00	183.00	31.65	4.00	0.00	2.00	1.00	1.00	0.00	1.00	3.00	3.00	114.29	3.00	36.00	1.00	3.00	1.00	19.00	76.00	0.00
122.00	27.00	90.00	174.00	29.73	3.00	1.00	2.00	2.00	0.00	0.00	1.00	3.00	5.50	200.00	3.00	28.00	0.00	4.00	1.00	16.00	64.00	0.00
123.00	30.00	82.00	169.00	28.71	3.00	1.00	2.00	1.00	1.00	1.00	1.00	2.00	7.00	124.29	3.00	31.00	1.00	0.00	1.00	24.00	96.00	0.00
124.00	32.00	83.00	180.00	25.62	3.00	1.00	3.00	2.00	0.00	0.00	1.00	3.00	3.00	135.00	3.00	33.00	1.00	7.00	0.00	20.00	80.00	0.00
125.00	30.00	60.00	161.00	23.15	2.00	0.00	2.00	1.00	0.00	0.00	0.00	2.00	3.00	100.00	3.00	26.00	0.00	4.00	1.00	19.00	76.00	0.00
126.00	29.00	75.00	177.00	23.94	2.00	1.00	2.00	2.00	0.00	0.00	1.00	3.00	3.50	64.29	3.00	33.00	1.00	3.00	1.00	22.00	88.00	0.00
127.00	33.00	72.00	176.00	23.24	2.00	1.00	2.00	2.00	1.00	0.00	0.00	3.00	3.00	170.00	3.00	36.00	1.00	6.00	0.00	21.00	84.00	0.00
128.00	28.00	67.00	168.00	23.74	2.00	0.00	2.00	1.00	0.00	0.00	1.00	3.00	5.50	87.86	3.00	30.00	1.00	4.00	1.00	22.00	88.00	0.00
129.00	30.00	51.00	155.00	21.23	2.00	0.00	2.00	2.00	0.00	0.00	0.00	3.00	2.50	90.00	3.00	37.00	1.00	2.00	1.00	17.00	68.00	0.00
130.00	28.00	81.00	187.00	23.16	2.00	0.00	2.00	1.00	0.00	0.00	1.00	3.00	5.50	282.86	3.00	23.00	0.00	8.00	0.00	22.00	88.00	0.00
131.00	28.00	55.00	140.00	28.06	3.00	0.00	1.00	1.00	0.00	0.00	0.00	3.00	4.00	565.71	3.00	25.00	0.00	12.00	0.00	14.00	56.00	0.00
132.00	28.00	80.00	174.00	26.42	3.00	1.00	1.00	1.00	1.00	1.00	1.00	1.00	5.50	157.14	3.00	38.00	1.00	1.00	1.00	18.00	72.00	0.00

Subject	Age	Weight	Height	BMI	BMI_C	Residence	Education	Marital_Status	Smoking	Chronic_Dise	Gender	Health	Sitting_Time	PA_Min_Day	IPAQ_C	Dietary_Score	Dietary_C	PSQI_Total	Sleep_C	WHO5_Total	WHO5_Total100	WHO5_Class
133.00	35.00	60.00	167.00	21.51	2.00	1.00	1.00	2.00	0.00	0.00	1.00	3.00	3.00	102.86	3.00	28.00	0.00	4.00	1.00	17.00	68.00	0.00
134.00	44.00	90.00	173.00	30.07	4.00	1.00	3.00	2.00	0.00	0.00	1.00	2.00	4.50	85.71	3.00	20.00	0.00	3.00	1.00	19.00	76.00	0.00
135.00	36.00	87.00	178.00	27.46	3.00	1.00	2.00	2.00	0.00	0.00	1.00	3.00	3.50	214.29	3.00	26.00	0.00	7.00	0.00	18.00	72.00	0.00
136.00	36.00	88.00	183.00	26.28	3.00	1.00	2.00	2.00	1.00	1.00	1.00	3.00	7.00	128.57	3.00	33.00	1.00	5.00	1.00	21.00	84.00	0.00
137.00	39.00	60.00	164.00	22.31	2.00	1.00	3.00	2.00	1.00	0.00	0.00	3.00	7.00	150.00	3.00	32.00	1.00	10.00	0.00	22.00	88.00	0.00
138.00	35.00	95.00	177.00	30.32	4.00	1.00	1.00	2.00	0.00	0.00	1.00	3.00	5.00	75.00	3.00	39.00	1.00	7.00	0.00	17.00	68.00	0.00
139.00	35.00	89.00	177.00	28.41	3.00	1.00	1.00	2.00	0.00	0.00	1.00	3.00	5.00	150.00	3.00	39.00	1.00	2.00	1.00	21.00	84.00	0.00
140.00	36.00	88.00	178.00	27.77	3.00	1.00	2.00	2.00	1.00	0.00	1.00	3.00	4.00	150.00	3.00	35.00	1.00	4.00	1.00	17.00	68.00	0.00
141.00	42.00	56.00	165.00	20.57	2.00	1.00	2.00	2.00	0.00	0.00	0.00	2.00	7.00	92.14	3.00	30.00	1.00	4.00	1.00	16.00	64.00	0.00
142.00	43.00	106.00	172.00	35.83	4.00	1.00	2.00	2.00	1.00	1.00	1.00	1.00	3.00	145.71	3.00	27.00	0.00	13.00	0.00	20.00	80.00	0.00
143.00	36.00	95.00	189.00	26.60	3.00	1.00	2.00	2.00	1.00	1.00	1.00	2.00	9.00	128.57	3.00	34.00	1.00	2.00	1.00	16.00	64.00	0.00
144.00	43.00	90.00	170.00	31.14	4.00	1.00	1.00	2.00	1.00	1.00	1.00	2.00	5.00	154.29	3.00	36.00	1.00	6.00	0.00	18.00	72.00	0.00
145.00	40.00	73.00	176.00	23.57	2.00	1.00	2.00	2.00	0.00	0.00	0.00	3.00	5.50	188.57	3.00	31.00	1.00	8.00	0.00	17.00	68.00	0.00
146.00	39.00	80.00	156.00	32.87	4.00	1.00	2.00	3.00	0.00	1.00	0.00	1.00	5.00	180.00	3.00	25.00	0.00	7.00	0.00	15.00	60.00	0.00
147.00	39.00	60.00	170.00	20.76	2.00	1.00	2.00	2.00	1.00	0.00	0.00	3.00	3.00	141.43	3.00	35.00	1.00	2.00	1.00	17.00	68.00	0.00
148.00	34.00	58.00	165.00	21.30	2.00	1.00	3.00	1.00	0.00	0.00	0.00	3.00	2.50	255.71	3.00	39.00	1.00	2.00	1.00	23.00	92.00	0.00
149.00	38.00	100.00	170.00	34.60	4.00	1.00	1.00	2.00	0.00	0.00	0.00	3.00	5.00	120.00	3.00	24.00	0.00	4.00	1.00	17.00	68.00	0.00
150.00	41.00	76.00	170.00	26.30	3.00	1.00	2.00	2.00	0.00	0.00	1.00	3.00	7.00	85.71	3.00	32.00	1.00	1.00	1.00	18.00	72.00	0.00
151.00	44.00	95.00	182.00	28.68	3.00	1.00	2.00	2.00	0.00	0.00	1.00	3.00	6.00	290.00	3.00	35.00	1.00	4.00	1.00	18.00	72.00	0.00
152.00	37.00	75.00	180.00	23.15	2.00	0.00	1.00	2.00	0.00	0.00	1.00	3.00	1.50	125.00	3.00	31.00	1.00	3.00	1.00	19.00	76.00	0.00
153.00	40.00	92.00	180.00	28.40	3.00	1.00	3.00	2.00	0.00	0.00	1.00	3.00	7.00	103.57	3.00	35.00	1.00	2.00	1.00	17.00	68.00	0.00
154.00	35.00	73.00	165.00	26.81	3.00	1.00	3.00	3.00	0.00	1.00	0.00	1.00	5.00	152.14	3.00	38.00	1.00	7.00	0.00	17.00	68.00	0.00
155.00	42.00	90.00	186.00	26.01	3.00	1.00	3.00	2.00	1.00	1.00	1.00	2.00	4.50	250.00	3.00	38.00	1.00	10.00	0.00	13.00	52.00	0.00
156.00	40.00	90.00	180.00	27.78	3.00	1.00	3.00	2.00	0.00	1.00	1.00	2.00	3.00	244.43	3.00	23.00	0.00	8.00	0.00	17.00	68.00	0.00
157.00	40.00	70.00	175.00	22.86	2.00	1.00	3.00	1.00	0.00	0.00	1.00	3.00	7.00	201.43	3.00	38.00	1.00	1.00	1.00	23.00	92.00	0.00
158.00	37.00	88.00	178.00	27.77	3.00	1.00	3.00	1.00	0.00	0.00	1.00	3.00	3.00	132.14	3.00	29.00	0.00	2.00	1.00	20.00	80.00	0.00
159.00	42.00	90.00	170.00	31.14	4.00	1.00	2.00	2.00	0.00	0.00	1.00	3.00	7.00	180.00	3.00	27.00	0.00	3.00	1.00	14.00	56.00	0.00
160.00	43.00	68.00	159.00	26.90	3.00	1.00	2.00	3.00	0.00	0.00	1.00	3.00	6.00	368.00	3.00	30.00	1.00	6.00	0.00	16.00	64.00	0.00
161.00	34.00	71.00	168.00	25.16	3.00	0.00	2.00	2.00	0.00	0.00	1.00	3.00	4.00	118.57	3.00	38.00	1.00	1.00	1.00	21.00	84.00	0.00
162.00	37.00	78.00	179.00	24.34	2.00	1.00	2.00	2.00	0.00	0.00	1.00	3.00	3.50	101.43	3.00	35.00	1.00	6.00	0.00	20.00	80.00	0.00
163.00	40.00	78.00	178.00	24.62	2.00	0.00	2.00	2.00	0.00	0.00	1.00	3.00	2.00	257.14	3.00	31.00	1.00	11.00	0.00	17.00	68.00	0.00
164.00	39.00	74.00	183.00	22.10	2.00	1.00	3.00	2.00	0.00	0.00	1.00	3.00	4.00	317.14	3.00	34.00	1.00	5.00	1.00	16.00	64.00	0.00
165.00	50.00	95.00	173.00	31.74	4.00	1.00	2.00	2.00	0.00	0.00	1.00	3.00	4.00	77.14	3.00	34.00	1.00	4.00	1.00	17.00	68.00	0.00
166.00	36.00	82.00	174.00	27.08	3.00	1.00	1.00	2.00	0.00	0.00	1.00	3.00	5.00	137.14	3.00	35.00	1.00	6.00	0.00	21.00	84.00	0.00
167.00	58.00	90.00	178.00	28.41	3.00	1.00	1.00	2.00	0.00	0.00	1.00	3.00	8.00	148.57	3.00	36.00	1.00	4.00	1.00	17.00	68.00	0.00
168.00	51.00	70.00	168.00	24.80	2.00	1.00	2.00	2.00	0.00	0.00	0.00	3.00	5.00	160.00	3.00	37.00	1.00	1.00	1.00	21.00	84.00	0.00
169.00	49.00	65.00	170.00	22.49	2.00	1.00	2.00	2.00	0.00	0.00	1.00	3.00	5.00	421.43	3.00	31.00	1.00	0.00	1.00	23.00	92.00	0.00
170.00	47.00	73.00	173.00	24.39	2.00	1.00	2.00	2.00	0.00	0.00	1.00	3.00	6.00	222.86	3.00	42.00	1.00	0.00	1.00	23.00	92.00	0.00
171.00	49.00	97.00	172.00	32.79	4.00	0.00	2.00	2.00	0.00	0.00	1.00	3.00	6.00	244.29	3.00	38.00	1.00	4.00	1.00	21.00	84.00	0.00
172.00	51.00	77.00	174.00	25.43	3.00	1.00	2.00	2.00	0.00	0.00	1.00	3.00	6.00	148.57	3.00	36.00	1.00	6.00	0.00	21.00	84.00	0.00
173.00	48.00	82.00	181.00	25.03	3.00	1.00	2.00	2.00	1.00	1.00	1.00	2.00	2.00	207.86	3.00	24.00	0.00	3.00	1.00	17.00	68.00	0.00
174.00	50.00	110.00	190.00	30.47	4.00	1.00	3.00	2.00	0.00	0.00	1.00	3.00	4.50	130.00	3.00	34.00	1.00	5.00	1.00	18.00	72.00	0.00
175.00	55.00	98.00	183.00	29.26	3.00	1.00	3.00	2.00	0.00	0.00	1.00	3.00	4.00	87.14	3.00	36.00	1.00	11.00	0.00	17.00	68.00	0.00
176.00	53.00	90.00	182.00	27.17	3.00	1.00	3.00	2.00	0.00	0.00	1.00	3.00	6.00	191.43	3.00	29.00	0.00	4.00	1.00	17.00	68.00	0.00
177.00	51.00	67.00	158.00	26.84	3.00	1.00	3.00	2.00	1.00	1.00	1.00	2.00	9.00	111.43	3.00	36.00	1.00	5.00	1.00	19.00	76.00	0.00
178.00	48.00	92.00	184.00	27.17	3.00	1.00	3.00	3.00	1.00	1.00	1.00	2.00	5.50	82.14	3.00	38.00	1.00	7.00	0.00	17.00	68.00	0.00
179.00	46.00	56.00	156.00	23.01	2.00	1.00	3.00	1.00	0.00	0.00	0.00	2.00	8.00	145.71	3.00	32.00	1.00	7.00	0.00	17.00	68.00	0.00
180.00	47.00	62.00	164.00	23.05	2.00	1.00	3.00	2.00	0.00	0.00	0.00	2.00	8.00	156.43	3.00	32.00	1.00	6.00	0.00	17.00	68.00	0.00
181.00	59.00	90.00	190.00	24.93	2.00	1.00	3.00	2.00	0.00	0.00	1.00	3.00	8.00	102.86	3.00	30.00	1.00	5.00	1.00	14.00	56.00	0.00
182.00	64.00	74.00	177.00	23.62	2.00	1.00	3.00	2.00	1.00	1.00	1.00	1.00	10.00	141.43	3.00	39.00	1.00	3.00	1.00	20.00	80.00	0.00
183.00	47.00	88.00	170.00	30.45	4.00	1.00	3.00	2.00	0.00	1.00	1.00	2.00	3.50	107.86	3.00	40.00	1.00	7.00	0.00	17.00	68.00	0.00
184.00	46.00	95.00	185.00	27.76	3.00	1.00	3.00	2.00	0.00	0.00	1.00	3.00	4.00	145.71	3.00	40.00	1.00	0.00	1.00	20.00	80.00	0.00
185.00	55.00	77.00	170.00	26.64	3.00	0.00	2.00	2.00	0.00	0.00	1.00	3.00	4.00	80.00	3.00	31.00	1.00	4.00	1.00	17.00	68.00	0.00
186.00	60.00	79.00	168.00	27.99	3.00	1.00	2.00	2.00	0.00	0.00	1.00	3.00	6.00	135.00	3.00	40.00	1.00	1.00	1.00	23.00	92.00	0.00
187.00	58.00	80.00	170.00	27.68	3.00	1.00	3.00	2.00	0.00	1.00	1.00	3.00	4.00	117.86	3.00	35.00	1.00	6.00	0.00	20.00	80.00	0.00
188.00	22.00	120.00	185.00	35.06	4.00	1.00	1.00	1.00	0.00	1.00	1.00	2.00	7.00	51.43	2.00	31.00	1.00	5.00	1.00	15.00	60.00	0.00
189.00	18.00	75.00	165.00	27.55	3.00	0.00	1.00	1.00	0.00	0.00	0.00	2.00	2.50	81.43	2.00	30.00	1.00	8.00	0.00	17.00	68.00	0.00
190.00	20.00	80.00	178.00	25.25	3.00	1.00	2.00	1.00	1.00	0.00	1.00	3.00	4.00	85.71	2.00	24.00	0.00	3.00	1.00	17.00	68.00	0.00
191.00	23.00	80.00	189.00	22.40	2.00	1.00	1.00	1.00	0.00	0.00	1.00	3.00	8.00	64.29	2.00	20.00	0.00	6.00	0.00	21.00	84.00	0.00
192.00	23.00	82.00	180.00	25.31	3.00	1.00	2.00	1.00	0.00	0.00	1.00	3.00	10.00	102.14	2.00	38.00	1.00	2.00	1.00	16.00	64.00	0.00
193.00	20.00	80.00	178.00	25.25	3.00	1.00	2.00	1.00	1.00	1.00	1.00	2.00	6.00	55.71	2.00	27.00	0.00	10.00	0.00	13.00	52.00	0.00
194.00	21.00	56.00	161.00	21.60	2.00	1.00	2.00	1.00	0.00	0.00	0.00	3.00	5.50	77.86	2.00	35.00	1.00	10.00	0.00	15.00	60.00	0.00
195.00	19.00	84.00	197.00	21.64	2.00	1.00	2.00	1.00	1.00	1.00	1.00	2.00	10.00	85.71	2.00	26.00	0.00	5.00	1.00	17.00	68.00	0.00
196.00	22.00	65.00	173.00	21.72	2.00	1.00	2.00	1.00	1.00	0.00	1.00	3.00	5.00	34.29	2.00	9.00	0.00	9.00	0.00	12.00	48.00	1.00
197.00	18.00	70.00	177.00	22.34	2.00	1.00	2.00	1.00	0.00	0.00	1.00	3.00	11.00	80.00	2.00	30.00	1.00	1.00	1.00	21.00	84.00	0.00
198.00	22.00	49.00	158.00	19.63	2.00	1.00	2.00	1.00	0.00	0.00	0.00	3.00	5.00	94.29	2.00	31.00	1.00	1.00	1.00	20.00	80.00	0.00

Subject	Age	Weight	Height	BMI	BMI_C	Residence	Education	Marital_Status	Smoking	Chronic_Dise	Gender	Health	Sitting_Time	PA_Min_Day	IPAQ_C	Dietary_Score	Dietary_C	PSQI_Total	Sleep_C	WHO5_Total	WHO5_Total100	WHO5_Class
**199.00**	21.00	77.00	176.00	24.86	2.00	0.00	1.00	1.00	0.00	0.00	1.00	3.00	4.00	78.57	2.00	39.00	1.00	2.00	1.00	18.00	72.00	0.00
**200.00**	21.00	75.00	180.00	23.15	2.00	1.00	2.00	1.00	0.00	0.00	1.00	3.00	4.50	85.71	2.00	23.00	0.00	5.00	1.00	14.00	56.00	0.00
**201.00**	19.00	70.00	169.00	24.51	2.00	1.00	2.00	1.00	0.00	0.00	0.00	3.00	8.00	46.00	2.00	30.00	1.00	8.00	0.00	18.00	72.00	0.00
**202.00**	21.00	70.00	162.00	26.67	3.00	0.00	2.00	1.00	1.00	0.00	0.00	3.00	6.50	85.71	2.00	26.00	0.00	5.00	1.00	16.00	64.00	0.00
**203.00**	20.00	52.00	160.00	20.31	2.00	1.00	1.00	1.00	1.00	1.00	1.00	1.00	6.50	50.71	2.00	20.00	0.00	7.00	0.00	12.00	48.00	1.00
**204.00**	19.00	67.70	161.00	26.12	3.00	1.00	2.00	1.00	0.00	0.00	0.00	3.00	7.00	66.43	2.00	33.00	1.00	7.00	0.00	19.00	76.00	0.00
**205.00**	21.00	64.00	166.00	23.23	2.00	1.00	2.00	1.00	1.00	0.00	1.00	3.00	6.50	85.71	2.00	25.00	0.00	3.00	1.00	18.00	72.00	0.00
**206.00**	19.00	94.00	175.00	30.69	4.00	1.00	1.00	1.00	0.00	0.00	0.00	2.00	7.00	47.14	2.00	27.00	0.00	5.00	1.00	16.00	64.00	0.00
**207.00**	21.00	56.00	136.00	30.28	4.00	1.00	1.00	1.00	1.00	1.00	1.00	2.00	10.00	42.86	2.00	18.00	0.00	3.00	1.00	18.00	72.00	0.00
**208.00**	18.00	65.00	161.00	25.08	3.00	1.00	1.00	1.00	0.00	0.00	0.00	3.00	6.00	94.29	2.00	25.00	0.00	4.00	1.00	21.00	84.00	0.00
**209.00**	21.00	65.00	159.00	25.71	3.00	0.00	1.00	1.00	0.00	0.00	0.00	2.00	4.00	80.71	2.00	31.00	1.00	8.00	0.00	16.00	64.00	0.00
**210.00**	22.00	67.00	160.00	26.17	3.00	0.00	1.00	2.00	0.00	0.00	0.00	2.00	4.00	111.43	2.00	32.00	1.00	5.00	1.00	19.00	76.00	0.00
**211.00**	21.00	67.00	158.00	26.84	3.00	1.00	1.00	1.00	0.00	0.00	0.00	3.00	9.00	61.71	2.00	31.00	1.00	4.00	1.00	14.00	56.00	0.00
**212.00**	22.00	70.00	153.00	29.90	3.00	1.00	1.00	1.00	0.00	0.00	0.00	3.00	12.00	60.00	2.00	29.00	0.00	9.00	0.00	15.00	60.00	0.00
**213.00**	23.00	87.00	176.00	28.09	3.00	1.00	1.00	1.00	0.00	0.00	1.00	3.00	11.00	60.00	2.00	30.00	1.00	1.00	1.00	20.00	80.00	0.00
**214.00**	21.00	85.00	176.00	27.44	3.00	1.00	2.00	1.00	0.00	0.00	1.00	2.00	2.00	71.43	2.00	34.00	1.00	7.00	0.00	16.00	64.00	0.00
**215.00**	21.00	65.00	159.00	25.71	3.00	1.00	2.00	2.00	1.00	0.00	0.00	3.00	7.00	103.57	2.00	34.00	1.00	0.00	1.00	23.00	92.00	0.00
**216.00**	21.00	80.00	176.00	25.83	3.00	0.00	2.00	1.00	0.00	0.00	0.00	2.00	8.00	85.71	2.00	24.00	0.00	2.00	1.00	16.00	64.00	0.00
**217.00**	22.00	64.00	158.00	25.64	3.00	1.00	2.00	1.00	0.00	0.00	0.00	3.00	7.00	60.71	2.00	19.00	0.00	7.00	0.00	16.00	64.00	0.00
**218.00**	21.00	67.00	162.00	25.53	3.00	1.00	2.00	1.00	0.00	0.00	0.00	3.00	4.00	72.86	2.00	30.00	1.00	2.00	1.00	16.00	64.00	0.00
**219.00**	22.00	90.00	189.00	25.20	3.00	1.00	2.00	1.00	1.00	0.00	1.00	3.00	6.50	38.57	2.00	29.00	0.00	9.00	0.00	13.00	52.00	0.00
**220.00**	21.00	60.00	153.00	25.63	3.00	0.00	2.00	1.00	0.00	0.00	0.00	3.00	8.00	94.29	2.00	30.00	1.00	4.00	1.00	19.00	76.00	0.00
**221.00**	21.00	82.00	177.00	26.17	3.00	0.00	2.00	1.00	0.00	0.00	0.00	3.00	8.00	57.14	2.00	28.00	0.00	3.00	1.00	18.00	72.00	0.00
**222.00**	20.00	70.00	174.00	23.12	2.00	1.00	1.00	1.00	0.00	0.00	0.00	1.00	5.50	81.43	2.00	31.00	1.00	10.00	0.00	15.00	60.00	0.00
**223.00**	20.00	53.00	158.00	21.23	2.00	1.00	1.00	1.00	1.00	0.00	0.00	2.00	6.00	71.43	2.00	14.00	0.00	2.00	1.00	16.00	64.00	0.00
**224.00**	22.00	57.00	161.00	21.99	2.00	0.00	1.00	1.00	0.00	0.00	0.00	2.00	10.00	51.43	2.00	23.00	0.00	14.00	0.00	12.00	48.00	1.00
**225.00**	21.00	57.00	165.00	20.94	2.00	1.00	1.00	1.00	0.00	0.00	0.00	2.00	5.50	42.86	2.00	31.00	1.00	6.00	0.00	7.00	28.00	1.00
**226.00**	23.00	53.00	160.00	20.70	2.00	0.00	1.00	1.00	0.00	0.00	0.00	2.00	7.00	43.57	2.00	34.00	1.00	4.00	1.00	19.00	76.00	0.00
**227.00**	20.00	48.00	159.00	18.99	2.00	1.00	1.00	1.00	0.00	0.00	0.00	2.00	7.00	43.57	2.00	32.00	1.00	1.00	1.00	17.00	68.00	0.00
**228.00**	20.00	60.00	160.00	23.44	2.00	0.00	1.00	1.00	0.00	0.00	0.00	3.00	7.00	90.00	2.00	21.00	0.00	7.00	0.00	15.00	60.00	0.00
**229.00**	18.00	53.40	159.00	21.12	2.00	1.00	1.00	1.00	0.00	0.00	0.00	3.00	10.00	94.29	2.00	12.00	0.00	10.00	0.00	16.00	64.00	0.00
**230.00**	20.00	67.00	166.00	24.31	2.00	1.00	1.00	1.00	0.00	0.00	0.00	3.00	5.00	72.86	2.00	28.00	0.00	4.00	1.00	18.00	72.00	0.00
**231.00**	22.00	62.00	162.00	23.62	2.00	1.00	1.00	1.00	0.00	0.00	0.00	3.00	3.50	85.71	2.00	27.00	0.00	0.00	1.00	21.00	84.00	0.00
**232.00**	21.00	58.00	167.00	20.80	2.00	0.00	1.00	1.00	0.00	0.00	0.00	3.00	7.00	81.43	2.00	27.00	0.00	3.00	1.00	21.00	84.00	0.00
**233.00**	21.00	57.00	159.00	22.55	2.00	1.00	1.00	1.00	0.00	0.00	0.00	3.00	4.00	38.57	2.00	25.00	0.00	4.00	1.00	17.00	68.00	0.00
**234.00**	21.00	68.00	183.00	20.31	2.00	0.00	1.00	1.00	0.00	0.00	1.00	3.00	5.00	64.29	2.00	32.00	1.00	1.00	1.00	18.00	72.00	0.00
**235.00**	20.00	70.00	175.00	22.86	2.00	1.00	1.00	1.00	1.00	1.00	1.00	1.00	5.00	54.29	2.00	34.00	1.00	3.00	1.00	23.00	92.00	0.00
**236.00**	22.00	70.00	180.00	21.60	2.00	1.00	1.00	1.00	1.00	1.00	1.00	2.00	8.00	81.43	2.00	32.00	1.00	11.00	0.00	11.00	44.00	1.00
**237.00**	20.00	78.00	183.00	23.29	2.00	1.00	1.00	1.00	1.00	1.00	1.00	2.00	5.50	34.29	2.00	27.00	0.00	7.00	0.00	15.00	60.00	0.00
**238.00**	19.00	68.00	174.00	22.46	2.00	1.00	1.00	1.00	1.00	1.00	0.00	2.00	5.00	64.29	2.00	27.00	0.00	5.00	1.00	16.00	64.00	0.00
**239.00**	20.00	73.00	187.00	20.88	2.00	1.00	1.00	1.00	1.00	1.00	1.00	2.00	5.50	96.00	2.00	31.00	1.00	5.00	1.00	19.00	76.00	0.00
**240.00**	21.00	89.00	191.00	24.40	2.00	0.00	1.00	3.00	1.00	1.00	1.00	2.00	5.50	40.00	2.00	26.00	0.00	5.00	1.00	17.00	68.00	0.00
**241.00**	23.00	78.00	196.00	20.30	2.00	0.00	1.00	1.00	1.00	1.00	1.00	2.00	5.50	64.29	2.00	18.00	0.00	6.00	0.00	16.00	64.00	0.00
**242.00**	19.00	51.00	154.00	21.50	2.00	1.00	1.00	1.00	1.00	1.00	0.00	3.00	6.00	92.86	2.00	35.00	1.00	8.00	0.00	18.00	72.00	0.00
**243.00**	19.00	53.00	160.00	20.70	2.00	1.00	2.00	1.00	0.00	0.00	0.00	1.00	6.00	64.29	2.00	32.00	1.00	7.00	0.00	9.00	36.00	1.00
**244.00**	20.00	55.00	170.00	19.03	2.00	1.00	2.00	1.00	0.00	0.00	1.00	2.00	3.50	79.29	2.00	22.00	0.00	4.00	1.00	21.00	84.00	0.00
**245.00**	20.00	59.00	170.00	20.42	2.00	1.00	2.00	1.00	0.00	0.00	1.00	2.00	6.50	34.29	2.00	24.00	0.00	4.00	1.00	17.00	68.00	0.00
**246.00**	19.00	50.00	156.00	20.55	2.00	1.00	2.00	1.00	0.00	0.00	0.00	3.00	7.00	46.43	2.00	24.00	0.00	4.00	1.00	20.00	80.00	0.00
**247.00**	21.00	74.00	180.00	22.84	2.00	1.00	2.00	1.00	1.00	0.00	1.00	2.00	4.50	102.86	2.00	19.00	0.00	9.00	0.00	12.00	48.00	1.00
**248.00**	21.00	65.00	164.00	24.17	2.00	1.00	2.00	2.00	0.00	0.00	0.00	2.00	5.00	34.29	2.00	31.00	1.00	5.00	1.00	19.00	76.00	0.00
**249.00**	20.00	69.00	174.00	22.79	2.00	1.00	2.00	1.00	1.00	0.00	1.00	3.00	8.00	52.86	2.00	24.00	0.00	5.00	1.00	21.00	84.00	0.00
**250.00**	20.00	66.00	168.00	23.38	2.00	0.00	2.00	1.00	1.00	0.00	1.00	3.00	4.00	87.86	2.00	20.00	0.00	7.00	0.00	16.00	64.00	0.00
**251.00**	22.00	70.00	175.00	22.86	2.00	1.00	2.00	1.00	1.00	0.00	1.00	3.00	5.00	66.43	2.00	36.00	1.00	4.00	1.00	17.00	68.00	0.00
**252.00**	19.00	56.00	168.00	19.84	2.00	0.00	2.00	1.00	0.00	0.00	1.00	3.00	6.50	77.14	2.00	22.00	0.00	5.00	1.00	15.00	60.00	0.00
**253.00**	22.00	76.00	186.00	21.97	2.00	0.00	2.00	1.00	0.00	0.00	1.00	3.00	5.00	100.00	2.00	27.00	0.00	4.00	1.00	16.00	64.00	0.00
**254.00**	21.00	47.00	157.00	19.07	2.00	1.00	2.00	1.00	0.00	0.00	0.00	3.00	9.00	38.57	2.00	28.00	0.00	7.00	0.00	11.00	44.00	1.00
**255.00**	22.00	51.00	157.00	20.69	2.00	1.00	2.00	1.00	0.00	0.00	0.00	3.00	9.00	68.57	2.00	29.00	0.00	6.00	0.00	15.00	60.00	0.00
**256.00**	20.00	67.00	175.00	21.88	2.00	1.00	2.00	1.00	1.00	0.00	1.00	3.00	5.00	58.57	2.00	34.00	1.00	4.00	1.00	17.00	68.00	0.00
**257.00**	19.00	67.00	166.00	24.31	2.00	1.00	2.00	1.00	0.00	0.00	0.00	3.00	12.00	68.57	2.00	21.00	0.00	5.00	1.00	14.00	56.00	0.00
**258.00**	20.00	62.00	170.00	21.45	2.00	0.00	2.00	1.00	1.00	0.00	1.00	3.00	11.00	68.57	2.00	16.00	0.00	4.00	1.00	18.00	72.00	0.00
**259.00**	19.00	49.00	155.00	20.40	2.00	1.00	2.00	1.00	0.00	0.00	0.00	3.00	7.00	68.57	2.00	21.00	0.00	4.00	1.00	15.00	60.00	0.00
**260.00**	23.00	49.00	157.00	19.88	2.00	0.00	2.00	2.00	0.00	0.00	0.00	3.00	5.00	111.43	2.00	28.00	0.00	3.00	1.00	19.00	76.00	0.00
**261.00**	23.00	53.00	153.00	22.64	2.00	1.00	2.00	1.00	0.00	0.00	0.00	3.00	3.50	53.57	2.00	32.00	1.00	3.00	1.00	21.00	84.00	0.00
**262.00**	23.00	58.00	155.00	24.14	2.00	1.00	2.00	1.00	0.00	0.00	0.00	3.00	7.00	45.71	2.00	38.00	1.00	1.00	1.00	19.00	76.00	0.00
**263.00**	20.00	50.00	157.00	20.28	2.00	0.00	2.00	1.00	0.00	0.00	0.00	3.00	10.00	40.71	2.00	28.00	0.00	3.00	1.00	19.00	76.00	0.00
**264.00**	20.00	63.00	173.00	21.05	2.00	1.00	2.00	1.00	0.00	0.00	0.00	3.00	6.00	74.29	2.00	30.00	1.00	2.00	1.00	18.00	72.00	0.00

Subject	Age	Weight	Height	BMI	BMI_C	Residence	Education	Marital_Status	Smoking	Chronic_Dise	Gender	Health	Sitting_Time	PA_Min_Day	IPAQ_C	Dietary_Score	Dietary_C	PSQI_Total	Sleep_C	WHO5_Total	WHO5_Total100	WHO5_Class
**265.00**	20.00	64.00	168.00	22.68	2.00	0.00	2.00	2.00	0.00	0.00	0.00	3.00	9.00	55.71	2.00	35.00	1.00	2.00	1.00	19.00	76.00	0.00
**266.00**	20.00	56.00	157.00	22.72	2.00	1.00	2.00	1.00	0.00	1.00	0.00	2.00	9.00	72.86	2.00	32.00	1.00	3.00	1.00	16.00	64.00	0.00
**267.00**	22.00	60.00	164.00	22.31	2.00	1.00	2.00	1.00	0.00	1.00	0.00	3.00	7.00	66.43	2.00	22.00	0.00	7.00	0.00	17.00	68.00	0.00
**268.00**	21.00	71.00	173.00	23.72	2.00	0.00	2.00	1.00	1.00	1.00	1.00	3.00	6.00	53.57	2.00	26.00	0.00	8.00	0.00	15.00	60.00	0.00
**269.00**	23.00	56.00	159.00	22.15	2.00	1.00	3.00	1.00	0.00	0.00	0.00	1.00	6.50	34.29	2.00	31.00	1.00	9.00	0.00	9.00	36.00	1.00
**270.00**	22.00	52.00	168.00	18.42	1.00	1.00	1.00	1.00	0.00	0.00	0.00	1.00	6.00	98.57	2.00	37.00	1.00	3.00	1.00	15.00	60.00	0.00
**271.00**	18.00	56.00	178.00	17.67	1.00	1.00	1.00	1.00	0.00	0.00	1.00	2.00	8.00	115.71	2.00	20.00	0.00	7.00	0.00	17.00	68.00	0.00
**272.00**	18.00	39.00	150.00	17.33	1.00	1.00	1.00	1.00	0.00	0.00	0.00	2.00	1.00	72.86	2.00	34.00	1.00	3.00	1.00	18.00	72.00	0.00
**273.00**	18.00	50.00	170.00	17.30	1.00	0.00	1.00	1.00	0.00	0.00	1.00	3.00	4.50	71.43	2.00	29.00	0.00	3.00	1.00	16.00	64.00	0.00
**274.00**	22.00	88.00	173.00	29.40	3.00	1.00	2.00	1.00	0.00	0.00	0.00	3.00	5.50	94.29	2.00	20.00	0.00	5.00	1.00	17.00	68.00	0.00
**275.00**	23.00	89.00	176.00	28.73	3.00	1.00	2.00	1.00	0.00	1.00	1.00	1.00	4.00	68.57	2.00	27.00	0.00	8.00	0.00	18.00	72.00	0.00
**276.00**	23.00	60.00	162.00	22.86	2.00	0.00	2.00	1.00	0.00	0.00	0.00	3.00	6.50	72.86	2.00	27.00	0.00	4.00	1.00	16.00	64.00	0.00
**277.00**	19.00	60.00	172.00	20.28	2.00	0.00	2.00	1.00	0.00	0.00	1.00	3.00	3.50	101.43	2.00	24.00	0.00	7.00	0.00	9.00	36.00	1.00
**278.00**	23.00	72.00	175.00	23.51	2.00	1.00	2.00	1.00	1.00	1.00	1.00	2.00	5.00	97.00	2.00	34.00	1.00	6.00	0.00	11.00	44.00	1.00
**279.00**	21.00	65.00	176.00	20.98	2.00	0.00	2.00	1.00	1.00	0.00	1.00	2.00	4.50	83.57	2.00	27.00	0.00	2.00	1.00	17.00	68.00	0.00
**280.00**	20.00	59.00	169.00	20.66	2.00	0.00	2.00	1.00	1.00	0.00	0.00	3.00	6.50	71.86	2.00	26.00	0.00	2.00	1.00	19.00	76.00	0.00
**281.00**	23.00	56.00	159.00	22.15	2.00	0.00	2.00	1.00	0.00	0.00	0.00	3.00	6.00	52.86	2.00	33.00	1.00	2.00	1.00	17.00	68.00	0.00
**282.00**	21.00	59.00	165.00	21.67	2.00	1.00	2.00	1.00	0.00	0.00	0.00	3.00	7.00	60.00	2.00	22.00	0.00	5.00	1.00	17.00	68.00	0.00
**283.00**	27.00	56.00	167.00	20.08	2.00	0.00	1.00	1.00	0.00	0.00	0.00	3.00	6.00	64.29	2.00	38.00	1.00	0.00	1.00	16.00	64.00	0.00
**284.00**	27.00	70.00	180.00	21.60	2.00	1.00	1.00	1.00	0.00	1.00	1.00	3.00	4.00	85.71	2.00	25.00	0.00	5.00	1.00	16.00	64.00	0.00
**285.00**	31.00	65.00	175.00	21.22	2.00	1.00	3.00	2.00	0.00	0.00	0.00	3.00	3.00	42.86	2.00	29.00	0.00	1.00	1.00	19.00	76.00	0.00
**286.00**	26.00	112.00	193.00	30.07	4.00	0.00	2.00	2.00	0.00	0.00	1.00	3.00	8.00	51.43	2.00	35.00	1.00	1.00	1.00	18.00	72.00	0.00
**287.00**	24.00	75.00	172.00	25.35	3.00	1.00	2.00	1.00	0.00	0.00	1.00	3.00	6.00	72.86	2.00	26.00	0.00	2.00	1.00	19.00	76.00	0.00
**288.00**	33.00	75.00	172.00	25.35	3.00	1.00	2.00	2.00	1.00	1.00	1.00	2.00	4.00	71.43	2.00	37.00	1.00	5.00	1.00	14.00	56.00	0.00
**289.00**	28.00	87.00	180.00	26.85	3.00	1.00	2.00	2.00	0.00	0.00	1.00	3.00	6.00	61.43	2.00	31.00	1.00	3.00	1.00	19.00	76.00	0.00
**290.00**	26.00	83.00	178.00	26.20	3.00	1.00	2.00	1.00	0.00	0.00	1.00	3.00	7.00	109.29	2.00	30.00	1.00	5.00	1.00	19.00	76.00	0.00
**291.00**	28.00	76.00	169.00	26.61	3.00	0.00	3.00	2.00	1.00	0.00	1.00	3.00	6.00	85.71	2.00	28.00	0.00	5.00	1.00	16.00	64.00	0.00
**292.00**	25.00	48.00	153.00	20.50	2.00	0.00	2.00	1.00	0.00	0.00	0.00	3.00	4.00	45.00	2.00	25.00	0.00	9.00	0.00	9.00	36.00	1.00
**293.00**	24.00	60.00	167.00	21.51	2.00	0.00	2.00	1.00	0.00	0.00	1.00	3.00	3.50	75.71	2.00	34.00	1.00	6.00	0.00	19.00	76.00	0.00
**294.00**	28.00	70.00	170.00	24.22	2.00	1.00	2.00	2.00	0.00	0.00	1.00	3.00	3.50	52.14	2.00	30.00	1.00	3.00	1.00	21.00	84.00	0.00
**295.00**	24.00	68.00	170.00	23.53	2.00	1.00	2.00	1.00	1.00	1.00	1.00	2.00	7.00	81.43	2.00	30.00	1.00	2.00	1.00	16.00	64.00	0.00
**296.00**	25.00	62.00	167.00	22.23	2.00	0.00	3.00	1.00	0.00	0.00	0.00	3.00	7.00	65.71	2.00	37.00	1.00	5.00	1.00	18.00	72.00	0.00
**297.00**	24.00	77.00	172.00	26.03	3.00	1.00	2.00	1.00	1.00	0.00	0.00	1.00	9.00	57.86	2.00	27.00	0.00	5.00	1.00	11.00	44.00	1.00
**298.00**	29.00	102.00	189.00	28.55	3.00	1.00	2.00	2.00	0.00	1.00	1.00	2.00	2.00	75.00	2.00	28.00	0.00	5.00	1.00	16.00	64.00	0.00
**299.00**	26.00	82.00	182.00	24.76	2.00	1.00	2.00	2.00	1.00	0.00	1.00	3.00	3.50	75.00	2.00	36.00	1.00	2.00	1.00	18.00	72.00	0.00
**300.00**	30.00	63.00	165.00	23.14	2.00	1.00	2.00	2.00	0.00	0.00	0.00	3.00	3.50	72.86	2.00	33.00	1.00	4.00	1.00	20.00	80.00	0.00
**301.00**	27.00	65.00	167.00	23.31	2.00	1.00	2.00	1.00	1.00	0.00	0.00	3.00	4.50	42.86	2.00	34.00	1.00	9.00	0.00	16.00	64.00	0.00
**302.00**	26.00	58.00	157.00	23.53	2.00	1.00	2.00	1.00	1.00	0.00	0.00	3.00	4.00	55.71	2.00	25.00	0.00	3.00	1.00	21.00	84.00	0.00
**303.00**	32.00	81.00	184.00	23.92	2.00	1.00	2.00	1.00	0.00	0.00	1.00	3.00	8.00	38.57	2.00	33.00	1.00	1.00	1.00	19.00	76.00	0.00
**304.00**	27.00	53.00	170.00	18.34	1.00	1.00	2.00	2.00	1.00	0.00	0.00	2.00	7.00	68.57	2.00	30.00	1.00	3.00	1.00	16.00	64.00	0.00
**305.00**	32.00	120.00	195.00	31.56	4.00	1.00	2.00	3.00	0.00	0.00	1.00	3.00	5.00	72.86	2.00	26.00	0.00	2.00	1.00	19.00	76.00	0.00
**306.00**	30.00	84.00	179.00	26.22	3.00	1.00	2.00	1.00	0.00	0.00	1.00	3.00	5.00	32.86	2.00	27.00	0.00	2.00	1.00	19.00	76.00	0.00
**307.00**	32.00	60.00	160.00	23.44	2.00	1.00	2.00	2.00	0.00	1.00	0.00	2.00	7.00	51.43	2.00	37.00	1.00	8.00	0.00	16.00	64.00	0.00
**308.00**	27.00	69.00	172.00	23.32	2.00	1.00	1.00	1.00	0.00	0.00	1.00	3.00	3.50	70.71	2.00	36.00	1.00	2.00	1.00	19.00	76.00	0.00
**309.00**	28.00	63.00	175.00	20.57	2.00	1.00	3.00	1.00	0.00	0.00	1.00	3.00	2.50	92.14	2.00	24.00	0.00	2.00	1.00	18.00	72.00	0.00
**310.00**	31.00	90.00	158.00	36.05	4.00	1.00	1.00	1.00	0.00	0.00	0.00	3.00	9.00	68.57	2.00	34.00	1.00	2.00	1.00	18.00	72.00	0.00
**311.00**	24.00	59.00	167.00	21.16	2.00	1.00	2.00	1.00	1.00	1.00	0.00	3.00	6.50	72.86	2.00	28.00	0.00	7.00	0.00	16.00	64.00	0.00
**312.00**	30.00	75.00	168.00	26.57	3.00	0.00	1.00	1.00	0.00	0.00	1.00	3.00	4.50	90.71	2.00	35.00	1.00	2.00	1.00	19.00	76.00	0.00
**313.00**	27.00	77.00	163.00	28.98	3.00	1.00	2.00	1.00	0.00	0.00	0.00	3.00	2.50	62.14	2.00	34.00	1.00	1.00	1.00	19.00	76.00	0.00
**314.00**	32.00	70.00	170.00	24.22	2.00	1.00	2.00	1.00	1.00	0.00	0.00	3.00	5.00	47.14	2.00	22.00	0.00	3.00	1.00	17.00	68.00	0.00
**315.00**	28.00	59.00	160.00	23.05	2.00	1.00	2.00	1.00	0.00	0.00	0.00	1.00	2.00	70.00	2.00	33.00	1.00	4.00	1.00	15.00	60.00	0.00
**316.00**	25.00	51.00	160.00	19.92	2.00	1.00	2.00	1.00	0.00	0.00	0.00	3.00	8.00	38.57	2.00	20.00	0.00	9.00	0.00	18.00	72.00	0.00
**317.00**	30.00	122.00	183.00	36.43	4.00	1.00	2.00	2.00	0.00	0.00	1.00	1.00	6.00	62.14	2.00	24.00	0.00	9.00	0.00	9.00	36.00	1.00
**318.00**	30.00	85.00	180.00	26.23	3.00	1.00	2.00	1.00	0.00	0.00	1.00	3.00	8.00	60.00	2.00	35.00	1.00	8.00	0.00	13.00	52.00	0.00
**319.00**	31.00	75.00	180.00	23.15	2.00	1.00	2.00	1.00	0.00	0.00	1.00	3.00	1.50	103.71	2.00	28.00	0.00	4.00	1.00	18.00	72.00	0.00
**320.00**	31.00	60.00	159.00	23.73	2.00	1.00	2.00	2.00	0.00	0.00	1.00	3.00	5.00	54.00	2.00	29.00	0.00	6.00	0.00	19.00	76.00	0.00
**321.00**	30.00	75.00	178.00	23.67	2.00	1.00	2.00	2.00	0.00	0.00	1.00	3.00	1.50	72.14	2.00	44.00	1.00	4.00	1.00	21.00	84.00	0.00
**322.00**	26.00	52.00	155.00	21.64	2.00	1.00	2.00	1.00	0.00	0.00	0.00	3.00	10.00	57.14	2.00	36.00	1.00	6.00	0.00	16.00	64.00	0.00
**323.00**	31.00	78.00	172.00	26.37	3.00	1.00	3.00	2.00	0.00	0.00	1.00	3.00	6.00	42.86	2.00	31.00	1.00	10.00	0.00	7.00	28.00	1.00
**324.00**	31.00	58.00	160.00	22.66	2.00	1.00	3.00	1.00	1.00	0.00	0.00	3.00	4.00	85.71	2.00	21.00	0.00	13.00	0.00	12.00	48.00	1.00
**325.00**	28.00	56.00	160.00	21.88	2.00	1.00	3.00	2.00	0.00	0.00	0.00	3.00	3.50	45.71	2.00	30.00	1.00	4.00	1.00	15.00	60.00	0.00
**326.00**	24.00	69.00	159.00	27.29	3.00	1.00	1.00	2.00	0.00	0.00	0.00	1.00	6.00	57.86	2.00	29.00	0.00	8.00	0.00	15.00	60.00	0.00
**327.00**	25.00	73.00	170.00	25.26	3.00	1.00	1.00	1.00	0.00	0.00	1.00	3.00	7.00	82.86	2.00	32.00	1.00	5.00	1.00	17.00	68.00	0.00
**328.00**	24.00	90.00	187.00	25.74	3.00	0.00	2.00	1.00	1.00	0.00	1.00	3.00	8.00	68.57	2.00	27.00	0.00	9.00	0.00	19.00	76.00	0.00
**329.00**	24.00	83.00	182.00	25.06	3.00	1.00	2.00	1.00	0.00	0.00	1.00	3.00	6.00	85.71	2.00	35.00	1.00	5.00	1.00	17.00	68.00	0.00
**330.00**	29.00	80.00	180.00	24.69	2.00	1.00	1.00	2.00	0.00	0.00	1.00	3.00	10.00	51.43	2.00	31.00	1.00	2.00	1.00	19.00	76.00	0.00

Subject	Age	Weight	Height	BMI	BMI_C	Residence	Education	Marital_Status	Smoking	Chronic_Dise	Gender	Health	Sitting_Time	PA_Min_Day	IPAQ_C	Dietary_Score	Dietary_C	PSQI_Total	Sleep_C	WHO5_Total	WHO5_Total100	WHO5_Class
331.00	26.00	80.00	180.00	24.69	2.00	1.00	2.00	1.00	0.00	0.00	1.00	3.00	6.00	68.57	2.00	16.00	0.00	6.00	0.00	17.00	68.00	0.00
332.00	24.00	70.00	168.00	24.80	2.00	1.00	2.00	1.00	0.00	0.00	1.00	3.00	7.00	100.00	2.00	41.00	1.00	2.00	1.00	15.00	60.00	0.00
333.00	25.00	60.00	176.00	19.37	2.00	1.00	3.00	1.00	0.00	0.00	1.00	1.00	6.50	48.57	2.00	36.00	1.00	8.00	0.00	14.00	56.00	0.00
334.00	26.00	42.00	151.00	18.42	1.00	1.00	3.00	1.00	0.00	0.00	0.00	3.00	5.00	81.43	2.00	27.00	0.00	7.00	0.00	19.00	76.00	0.00
335.00	28.00	74.00	152.00	32.03	4.00	0.00	2.00	1.00	0.00	0.00	0.00	3.00	2.00	57.14	2.00	37.00	1.00	3.00	1.00	18.00	72.00	0.00
336.00	31.00	80.00	174.00	26.42	3.00	1.00	2.00	2.00	0.00	0.00	1.00	2.00	9.00	45.00	2.00	37.00	1.00	3.00	1.00	19.00	76.00	0.00
337.00	28.00	74.00	169.00	25.91	3.00	1.00	2.00	2.00	1.00	0.00	0.00	3.00	5.00	85.71	2.00	23.00	0.00	3.00	1.00	17.00	68.00	0.00
338.00	25.00	100.00	184.00	29.54	3.00	1.00	2.00	1.00	0.00	0.00	1.00	3.00	10.00	70.71	2.00	38.00	1.00	1.00	1.00	18.00	72.00	0.00
339.00	29.00	86.00	175.00	28.08	3.00	1.00	2.00	1.00	1.00	1.00	1.00	2.00	8.00	108.57	2.00	23.00	0.00	9.00	0.00	21.00	84.00	0.00
340.00	29.00	70.00	171.00	23.94	2.00	1.00	2.00	2.00	0.00	0.00	1.00	3.00	6.50	68.57	2.00	24.00	0.00	5.00	1.00	16.00	64.00	0.00
341.00	26.00	55.00	170.00	19.03	2.00	0.00	2.00	2.00	0.00	0.00	0.00	3.00	5.00	68.57	2.00	28.00	0.00	7.00	0.00	15.00	60.00	0.00
342.00	24.00	58.00	168.00	20.55	2.00	1.00	2.00	1.00	1.00	1.00	1.00	2.00	5.50	62.14	2.00	26.00	0.00	3.00	1.00	16.00	64.00	0.00
343.00	28.00	81.00	184.00	23.92	2.00	0.00	2.00	1.00	0.00	1.00	1.00	3.00	10.00	102.86	2.00	30.00	1.00	6.00	0.00	18.00	72.00	0.00
344.00	28.00	53.00	170.00	18.34	1.00	1.00	2.00	2.00	1.00	0.00	1.00	3.00	6.50	93.57	2.00	32.00	1.00	6.00	0.00	14.00	56.00	0.00
345.00	27.00	70.00	163.00	26.35	3.00	0.00	1.00	2.00	0.00	0.00	0.00	1.00	4.50	51.43	2.00	30.00	1.00	3.00	1.00	13.00	52.00	0.00
346.00	24.00	90.00	176.00	29.05	3.00	1.00	2.00	1.00	1.00	0.00	1.00	3.00	1.00	73.57	2.00	24.00	0.00	11.00	0.00	16.00	64.00	0.00
347.00	32.00	60.00	165.00	22.04	2.00	1.00	3.00	1.00	0.00	0.00	0.00	3.00	6.00	82.14	2.00	33.00	1.00	1.00	1.00	20.00	80.00	0.00
348.00	42.00	77.00	156.00	31.64	4.00	1.00	1.00	2.00	0.00	0.00	0.00	3.00	6.00	64.29	2.00	36.00	1.00	6.00	0.00	19.00	76.00	0.00
349.00	43.00	88.00	170.00	30.45	4.00	1.00	2.00	2.00	0.00	0.00	1.00	3.00	7.00	64.29	2.00	35.00	1.00	2.00	1.00	15.00	60.00	0.00
350.00	41.00	93.00	190.00	25.76	3.00	1.00	2.00	2.00	0.00	0.00	1.00	3.00	8.00	42.86	2.00	28.00	0.00	2.00	1.00	16.00	64.00	0.00
351.00	38.00	85.00	172.00	28.73	3.00	1.00	2.00	1.00	0.00	0.00	1.00	3.00	6.50	51.43	2.00	30.00	1.00	5.00	1.00	19.00	76.00	0.00
352.00	38.00	78.00	172.00	26.37	3.00	1.00	3.00	2.00	1.00	0.00	1.00	3.00	2.50	65.71	2.00	38.00	1.00	2.00	1.00	19.00	76.00	0.00
353.00	40.00	89.00	187.00	25.45	3.00	1.00	3.00	2.00	1.00	1.00	1.00	3.00	8.00	77.14	2.00	32.00	1.00	8.00	0.00	16.00	64.00	0.00
354.00	38.00	60.00	160.00	23.44	2.00	1.00	2.00	3.00	1.00	0.00	0.00	3.00	7.00	44.29	2.00	32.00	1.00	4.00	1.00	20.00	80.00	0.00
355.00	37.00	80.00	185.00	23.37	2.00	1.00	2.00	1.00	0.00	0.00	1.00	3.00	9.00	64.29	2.00	30.00	1.00	10.00	0.00	18.00	72.00	0.00
356.00	34.00	90.00	171.00	30.78	4.00	1.00	2.00	1.00	0.00	0.00	1.00	3.00	4.00	55.71	2.00	33.00	1.00	5.00	1.00	18.00	72.00	0.00
357.00	34.00	113.00	180.00	34.88	4.00	0.00	2.00	2.00	1.00	1.00	1.00	2.00	5.00	75.00	2.00	22.00	0.00	9.00	0.00	19.00	76.00	0.00
358.00	34.00	75.00	170.00	25.95	3.00	0.00	2.00	2.00	0.00	0.00	1.00	3.00	7.00	102.86	2.00	26.00	0.00	0.00	1.00	20.00	80.00	0.00
359.00	40.00	95.00	185.00	27.76	3.00	1.00	2.00	2.00	0.00	1.00	1.00	3.00	9.00	55.71	2.00	26.00	0.00	7.00	0.00	19.00	76.00	0.00
360.00	34.00	70.00	169.00	24.51	2.00	1.00	2.00	1.00	1.00	1.00	1.00	2.00	3.00	75.14	2.00	26.00	0.00	3.00	1.00	16.00	64.00	0.00
361.00	40.00	60.00	160.00	23.44	2.00	1.00	2.00	2.00	1.00	1.00	0.00	2.00	7.00	42.86	2.00	32.00	1.00	3.00	1.00	17.00	68.00	0.00
362.00	36.00	55.00	173.00	18.38	1.00	1.00	2.00	1.00	0.00	0.00	0.00	2.00	9.00	47.14	2.00	40.00	1.00	14.00	0.00	12.00	48.00	1.00
363.00	36.00	81.00	182.00	24.45	2.00	1.00	2.00	2.00	0.00	0.00	1.00	2.00	3.50	94.29	2.00	29.00	0.00	6.00	0.00	17.00	68.00	0.00
364.00	35.00	50.00	155.00	20.81	2.00	1.00	3.00	2.00	0.00	0.00	0.00	3.00	6.50	111.43	2.00	36.00	1.00	3.00	1.00	19.00	76.00	0.00
365.00	36.00	95.00	180.00	29.32	3.00	1.00	1.00	3.00	0.00	0.00	1.00	3.00	8.00	49.29	2.00	31.00	1.00	6.00	0.00	13.00	52.00	0.00
366.00	38.00	57.00	163.00	21.45	2.00	1.00	2.00	1.00	0.00	0.00	0.00	3.00	4.00	56.43	2.00	34.00	1.00	2.00	1.00	22.00	88.00	0.00
367.00	38.00	57.00	164.00	21.19	2.00	1.00	2.00	2.00	0.00	1.00	0.00	3.00	1.50	55.71	2.00	37.00	1.00	4.00	1.00	18.00	72.00	0.00
368.00	34.00	74.50	174.00	24.61	2.00	1.00	3.00	2.00	1.00	0.00	1.00	3.00	8.00	53.57	2.00	30.00	1.00	6.00	0.00	17.00	68.00	0.00
369.00	40.00	80.00	175.00	26.12	3.00	1.00	2.00	2.00	0.00	0.00	1.00	3.00	8.00	30.00	2.00	31.00	1.00	7.00	0.00	12.00	48.00	1.00
370.00	38.00	80.00	170.00	27.68	3.00	1.00	3.00	2.00	1.00	1.00	0.00	3.00	3.50	68.57	2.00	35.00	1.00	8.00	0.00	17.00	68.00	0.00
371.00	44.00	90.00	167.00	32.27	4.00	1.00	2.00	2.00	0.00	1.00	0.00	2.00	3.00	107.14	2.00	36.00	1.00	5.00	1.00	19.00	76.00	0.00
372.00	36.00	108.00	180.00	33.33	4.00	1.00	2.00	1.00	1.00	1.00	1.00	1.00	7.00	77.14	2.00	34.00	1.00	6.00	0.00	16.00	64.00	0.00
373.00	34.00	62.00	155.00	25.81	3.00	1.00	2.00	2.00	0.00	0.00	0.00	2.00	7.00	30.00	2.00	27.00	0.00	1.00	1.00	17.00	68.00	0.00
374.00	38.00	80.00	175.00	26.12	3.00	1.00	2.00	2.00	0.00	0.00	0.00	3.00	6.00	59.29	2.00	41.00	1.00	0.00	1.00	20.00	80.00	0.00
375.00	35.00	87.00	176.00	28.09	3.00	1.00	3.00	2.00	0.00	0.00	1.00	1.00	6.50	89.14	2.00	27.00	0.00	3.00	1.00	16.00	64.00	0.00
376.00	43.00	56.00	160.00	21.88	2.00	1.00	2.00	2.00	0.00	0.00	0.00	2.00	3.00	38.57	2.00	37.00	1.00	6.00	0.00	19.00	76.00	0.00
377.00	34.00	72.00	172.00	24.34	2.00	1.00	2.00	2.00	1.00	1.00	1.00	3.00	8.00	42.86	2.00	24.00	0.00	4.00	1.00	16.00	64.00	0.00
378.00	34.00	63.00	166.00	22.86	2.00	1.00	3.00	2.00	0.00	0.00	1.00	3.00	5.00	107.14	2.00	25.00	0.00	3.00	1.00	18.00	72.00	0.00
379.00	34.00	77.00	160.00	30.08	4.00	1.00	3.00	3.00	1.00	0.00	0.00	3.00	7.00	69.71	2.00	23.00	0.00	6.00	0.00	17.00	68.00	0.00
380.00	37.00	95.00	175.00	31.02	4.00	0.00	3.00	2.00	0.00	0.00	1.00	3.00	6.00	71.43	2.00	26.00	0.00	9.00	0.00	22.00	88.00	0.00
381.00	42.00	94.00	191.00	25.77	3.00	0.00	3.00	2.00	1.00	0.00	1.00	3.00	6.00	94.29	2.00	33.00	1.00	3.00	1.00	17.00	68.00	0.00
382.00	43.00	86.00	170.00	29.76	3.00	1.00	3.00	2.00	1.00	0.00	1.00	3.00	7.00	96.43	2.00	38.00	1.00	2.00	1.00	16.00	64.00	0.00
383.00	35.00	78.00	176.00	25.18	3.00	1.00	3.00	1.00	0.00	0.00	1.00	3.00	8.00	77.14	2.00	29.00	0.00	3.00	1.00	21.00	84.00	0.00
384.00	38.00	87.00	181.00	26.56	3.00	1.00	3.00	2.00	0.00	0.00	1.00	3.00	7.00	55.71	2.00	25.00	0.00	2.00	1.00	17.00	68.00	0.00
385.00	40.00	64.00	172.00	21.63	2.00	1.00	3.00	2.00	1.00	0.00	0.00	2.00	3.50	47.14	2.00	43.00	1.00	1.00	1.00	20.00	80.00	0.00
386.00	41.00	73.00	174.00	24.11	2.00	1.00	3.00	1.00	0.00	0.00	1.00	3.00	6.00	77.14	2.00	22.00	0.00	3.00	1.00	14.00	56.00	0.00
387.00	34.00	65.00	166.00	23.59	2.00	1.00	3.00	1.00	1.00	0.00	0.00	3.00	11.00	38.57	2.00	25.00	0.00	1.00	1.00	17.00	68.00	0.00
388.00	39.00	97.00	187.00	27.74	3.00	1.00	1.00	1.00	0.00	0.00	1.00	3.00	7.00	38.57	2.00	21.00	0.00	4.00	1.00	18.00	72.00	0.00
389.00	35.00	81.00	178.00	25.56	3.00	1.00	3.00	2.00	0.00	0.00	1.00	3.00	8.00	60.00	2.00	32.00	1.00	7.00	0.00	19.00	76.00	0.00
390.00	37.00	67.00	165.00	24.61	2.00	1.00	3.00	2.00	0.00	0.00	1.00	3.00	8.00	92.14	2.00	33.00	1.00	4.00	1.00	17.00	68.00	0.00
391.00	44.00	80.00	163.00	30.11	4.00	1.00	2.00	2.00	0.00	0.00	0.00	3.00	11.00	85.71	2.00	34.00	1.00	1.00	1.00	19.00	76.00	0.00
392.00	34.00	100.00	168.00	35.43	4.00	1.00	2.00	2.00	1.00	1.00	1.00	2.00	7.00	68.57	2.00	25.00	0.00	12.00	0.00	15.00	60.00	0.00
393.00	39.00	69.00	160.00	26.95	3.00	1.00	2.00	2.00	0.00	0.00	0.00	2.00	7.00	49.29	2.00	33.00	1.00	7.00	0.00	16.00	64.00	0.00
394.00	36.00	100.00	183.00	29.86	3.00	1.00	2.00	1.00	0.00	0.00	1.00	3.00	7.00	38.57	2.00	28.00	0.00	4.00	1.00	14.00	56.00	0.00
395.00	37.00	76.00	172.00	25.69	3.00	0.00	2.00	2.00	0.00	0.00	1.00	3.00	4.00	96.43	2.00	33.00	1.00	4.00	1.00	18.00	72.00	0.00
396.00	41.00	93.00	180.00	28.70	3.00	1.00	2.00	2.00	0.00	0.00	1.00	3.00	9.00	64.29	2.00	28.00	0.00	2.00	1.00	19.00	76.00	0.00

Subject	Age	Weight	Height	BMI	BMI_C	Residence	Education	Marital_Status	Smoking	Chronic_Dise	Gender	Health	Sitting_Time	PA_Min_Day	IPAQ_C	Dietary_Score	Dietary_C	PSQI_Total	Sleep_C	WHO5_Total	WHO5_Total100	WHO5_Class
397.00	39.00	85.00	175.00	27.76	3.00	1.00	2.00	2.00	0.00	1.00	1.00	3.00	2.00	85.71	2.00	28.00	0.00	8.00	0.00	17.00	68.00	0.00
398.00	40.00	74.00	168.00	26.22	3.00	0.00	2.00	2.00	0.00	1.00	0.00	3.00	4.00	74.29	2.00	30.00	1.00	0.00	1.00	19.00	76.00	0.00
399.00	35.00	87.00	177.00	27.77	3.00	1.00	3.00	2.00	0.00	0.00	1.00	2.00	6.00	65.71	2.00	26.00	0.00	7.00	0.00	14.00	56.00	0.00
400.00	39.00	75.00	165.00	27.55	3.00	1.00	3.00	2.00	0.00	0.00	1.00	2.00	6.00	65.71	2.00	33.00	1.00	6.00	0.00	19.00	76.00	0.00
401.00	35.00	75.00	167.00	26.89	3.00	1.00	3.00	2.00	0.00	0.00	0.00	3.00	8.00	81.43	2.00	34.00	1.00	4.00	1.00	19.00	76.00	0.00
402.00	39.00	73.00	165.00	26.81	3.00	0.00	3.00	2.00	0.00	0.00	1.00	3.00	7.00	80.00	2.00	27.00	0.00	0.00	1.00	21.00	84.00	0.00
403.00	42.00	80.00	170.00	27.68	3.00	1.00	3.00	2.00	0.00	1.00	1.00	3.00	6.50	57.86	2.00	36.00	1.00	12.00	0.00	15.00	60.00	0.00
404.00	38.00	64.00	166.00	23.23	2.00	1.00	2.00	1.00	0.00	0.00	0.00	3.00	6.50	98.57	2.00	29.00	0.00	6.00	0.00	14.00	56.00	0.00
405.00	40.00	65.00	169.00	22.76	2.00	1.00	2.00	2.00	0.00	0.00	1.00	3.00	2.00	111.43	2.00	37.00	1.00	5.00	1.00	22.00	88.00	0.00
406.00	42.00	69.00	176.00	22.28	2.00	1.00	2.00	2.00	0.00	0.00	0.00	3.00	2.50	90.00	2.00	23.00	0.00	3.00	1.00	19.00	76.00	0.00
407.00	39.00	70.00	170.00	24.22	2.00	1.00	3.00	2.00	0.00	0.00	1.00	3.00	4.50	68.57	2.00	30.00	1.00	6.00	0.00	19.00	76.00	0.00
408.00	38.00	50.00	170.00	17.30	1.00	1.00	2.00	2.00	1.00	0.00	0.00	2.00	3.50	84.29	2.00	28.00	0.00	12.00	0.00	15.00	60.00	0.00
409.00	46.00	67.00	160.00	26.17	3.00	1.00	2.00	3.00	0.00	0.00	0.00	3.00	5.00	68.57	2.00	37.00	1.00	3.00	1.00	18.00	72.00	0.00
410.00	54.00	95.00	185.00	27.76	3.00	0.00	2.00	2.00	0.00	0.00	1.00	3.00	8.00	64.29	2.00	37.00	1.00	2.00	1.00	18.00	72.00	0.00
411.00	67.00	77.00	165.00	28.28	3.00	1.00	2.00	2.00	1.00	1.00	1.00	3.00	4.00	114.00	2.00	31.00	1.00	5.00	1.00	20.00	80.00	0.00
412.00	46.00	88.00	182.00	26.57	3.00	1.00	3.00	2.00	0.00	0.00	1.00	3.00	6.00	84.29	2.00	33.00	1.00	5.00	1.00	16.00	64.00	0.00
413.00	53.00	63.00	153.00	26.91	3.00	1.00	3.00	2.00	0.00	0.00	0.00	3.00	10.00	77.14	2.00	28.00	0.00	5.00	1.00	17.00	68.00	0.00
414.00	63.00	79.00	173.00	26.40	3.00	1.00	3.00	2.00	1.00	0.00	1.00	3.00	11.00	90.00	2.00	42.00	1.00	4.00	1.00	19.00	76.00	0.00
415.00	60.00	80.00	170.00	27.68	3.00	0.00	2.00	2.00	1.00	1.00	1.00	2.00	5.00	34.29	2.00	37.00	1.00	1.00	1.00	20.00	80.00	0.00
416.00	52.00	123.00	185.00	35.94	4.00	0.00	1.00	2.00	1.00	0.00	1.00	2.00	9.00	90.00	2.00	25.00	0.00	5.00	1.00	12.00	48.00	1.00
417.00	52.00	100.00	184.00	29.54	3.00	1.00	1.00	2.00	0.00	0.00	1.00	3.00	2.50	68.57	2.00	34.00	1.00	2.00	1.00	17.00	68.00	0.00
418.00	50.00	60.00	168.00	21.26	2.00	1.00	2.00	2.00	0.00	0.00	0.00	3.00	6.50	72.86	2.00	36.00	1.00	2.00	1.00	16.00	64.00	0.00
419.00	45.00	65.00	163.00	24.46	2.00	1.00	2.00	2.00	1.00	1.00	0.00	2.00	3.00	92.14	2.00	35.00	1.00	9.00	0.00	17.00	68.00	0.00
420.00	60.00	64.00	163.00	24.09	2.00	1.00	2.00	2.00	1.00	1.00	0.00	3.00	7.00	81.43	2.00	35.00	1.00	4.00	1.00	18.00	72.00	0.00
421.00	66.00	86.00	175.00	28.08	3.00	1.00	3.00	2.00	0.00	0.00	1.00	3.00	9.00	81.43	2.00	29.00	0.00	3.00	1.00	18.00	72.00	0.00
422.00	50.00	96.00	168.00	34.01	4.00	1.00	2.00	2.00	1.00	1.00	0.00	3.00	11.00	101.43	2.00	28.00	0.00	12.00	0.00	12.00	48.00	1.00
423.00	48.00	107.00	183.00	31.95	4.00	0.00	2.00	2.00	0.00	0.00	1.00	3.00	10.00	41.43	2.00	27.00	0.00	3.00	1.00	17.00	68.00	0.00
424.00	47.00	110.00	170.00	38.06	4.00	1.00	2.00	1.00	1.00	1.00	1.00	1.00	11.00	77.14	2.00	29.00	0.00	15.00	0.00	7.00	28.00	1.00
425.00	47.00	110.00	170.00	38.06	4.00	1.00	2.00	2.00	0.00	1.00	1.00	2.00	2.00	72.86	2.00	33.00	1.00	4.00	1.00	17.00	68.00	0.00
426.00	58.00	96.00	169.00	33.61	4.00	1.00	2.00	2.00	1.00	1.00	1.00	2.00	5.00	85.71	2.00	41.00	1.00	6.00	0.00	15.00	60.00	0.00
427.00	45.00	110.00	189.00	30.79	4.00	1.00	2.00	2.00	0.00	1.00	1.00	3.00	7.00	58.57	2.00	25.00	0.00	6.00	0.00	19.00	76.00	0.00
428.00	48.00	90.00	175.00	29.39	3.00	0.00	2.00	2.00	1.00	1.00	1.00	1.00	7.00	104.29	2.00	30.00	1.00	7.00	0.00	13.00	52.00	0.00
429.00	55.00	73.00	175.00	23.84	2.00	1.00	2.00	2.00	1.00	0.00	1.00	2.00	4.00	92.14	2.00	25.00	0.00	6.00	0.00	17.00	68.00	0.00
430.00	55.00	48.00	150.00	21.33	2.00	1.00	2.00	2.00	0.00	0.00	0.00	3.00	1.50	56.43	2.00	26.00	0.00	1.00	1.00	21.00	84.00	0.00
431.00	65.00	102.00	181.00	31.13	4.00	1.00	3.00	3.00	0.00	0.00	1.00	3.00	6.00	90.71	2.00	34.00	1.00	86.21	0.00	21.00	84.00	0.00
432.00	54.00	110.00	191.00	30.15	4.00	1.00	3.00	2.00	0.00	0.00	1.00	3.00	10.00	85.71	2.00	27.00	0.00	3.00	1.00	16.00	64.00	0.00
433.00	50.00	101.00	179.00	31.52	4.00	0.00	3.00	2.00	1.00	0.00	1.00	3.00	7.00	62.14	2.00	34.00	1.00	3.00	1.00	16.00	64.00	0.00
434.00	69.00	107.00	180.00	33.02	4.00	1.00	3.00	2.00	1.00	1.00	1.00	2.00	7.00	115.71	2.00	35.00	1.00	11.00	0.00	14.00	56.00	0.00
435.00	61.00	105.00	173.00	35.08	4.00	1.00	3.00	2.00	1.00	1.00	1.00	2.00	5.00	102.86	2.00	32.00	1.00	7.00	0.00	17.00	68.00	0.00
436.00	50.00	99.00	178.00	31.25	4.00	1.00	3.00	2.00	0.00	1.00	1.00	2.00	7.00	82.86	2.00	30.00	1.00	3.00	1.00	18.00	72.00	0.00
437.00	64.00	84.00	162.00	32.01	4.00	1.00	3.00	1.00	0.00	1.00	0.00	3.00	3.50	42.86	2.00	39.00	1.00	3.00	1.00	20.00	80.00	0.00
438.00	50.00	92.00	178.00	29.04	3.00	1.00	3.00	2.00	0.00	0.00	1.00	2.00	8.00	71.43	2.00	33.00	1.00	4.00	1.00	17.00	68.00	0.00
439.00	48.00	94.00	186.00	27.17	3.00	1.00	3.00	2.00	0.00	0.00	1.00	3.00	6.00	111.43	2.00	33.00	1.00	4.00	1.00	20.00	80.00	0.00
440.00	57.00	90.00	175.00	29.39	3.00	1.00	3.00	2.00	0.00	0.00	1.00	3.00	8.00	68.57	2.00	34.00	1.00	1.00	1.00	21.00	84.00	0.00
441.00	63.00	95.00	185.00	27.76	3.00	1.00	3.00	2.00	0.00	0.00	1.00	3.00	8.00	75.00	2.00	28.00	0.00	3.00	1.00	18.00	72.00	0.00
442.00	60.00	87.00	171.00	29.75	3.00	1.00	3.00	2.00	0.00	0.00	1.00	3.00	5.00	64.29	2.00	34.00	1.00	2.00	1.00	19.00	76.00	0.00
443.00	50.00	79.00	165.00	29.02	3.00	1.00	3.00	2.00	0.00	0.00	1.00	3.00	6.00	115.71	2.00	27.00	0.00	5.00	1.00	17.00	68.00	0.00
444.00	46.00	90.00	182.00	27.17	3.00	1.00	3.00	2.00	0.00	0.00	1.00	3.00	5.50	85.71	2.00	28.00	0.00	5.00	1.00	16.00	64.00	0.00
445.00	49.00	96.00	184.00	28.36	3.00	1.00	3.00	2.00	0.00	1.00	1.00	2.00	6.50	57.86	2.00	33.00	1.00	6.00	0.00	17.00	68.00	0.00
446.00	53.00	88.00	172.00	29.75	3.00	1.00	3.00	2.00	1.00	1.00	1.00	2.00	8.00	77.14	2.00	30.00	1.00	10.00	0.00	21.00	84.00	0.00
447.00	50.00	60.00	160.00	23.44	2.00	1.00	3.00	2.00	0.00	0.00	1.00	3.00	2.50	70.71	2.00	28.00	0.00	4.00	1.00	18.00	72.00	0.00
448.00	50.00	70.00	170.00	24.22	2.00	1.00	3.00	2.00	1.00	0.00	0.00	3.00	4.00	55.71	2.00	42.00	1.00	3.00	1.00	18.00	72.00	0.00
449.00	48.00	74.00	177.00	23.62	2.00	1.00	3.00	1.00	1.00	0.00	1.00	3.00	7.00	110.00	2.00	28.00	0.00	1.00	1.00	19.00	76.00	0.00
450.00	50.00	75.00	175.00	24.49	2.00	0.00	3.00	2.00	1.00	1.00	1.00	2.00	6.00	77.14	2.00	34.00	1.00	6.00	0.00	21.00	84.00	0.00
451.00	53.00	70.00	150.00	31.11	4.00	1.00	2.00	2.00	0.00	0.00	0.00	3.00	3.00	68.57	2.00	27.00	0.00	4.00	1.00	17.00	68.00	0.00
452.00	48.00	65.00	156.00	26.71	3.00	1.00	2.00	2.00	0.00	0.00	0.00	3.00	1.50	65.71	2.00	38.00	1.00	4.00	1.00	21.00	84.00	0.00
453.00	59.00	103.00	188.00	29.14	3.00	1.00	2.00	2.00	0.00	0.00	1.00	3.00	6.00	81.43	2.00	27.00	0.00	4.00	1.00	17.00	68.00	0.00
454.00	47.00	88.00	175.00	28.73	3.00	1.00	2.00	2.00	0.00	0.00	1.00	3.00	4.00	81.43	2.00	25.00	0.00	5.00	1.00	16.00	64.00	0.00
455.00	45.00	78.00	162.00	29.72	3.00	1.00	2.00	2.00	0.00	1.00	1.00	2.00	11.00	60.00	2.00	27.00	0.00	9.00	0.00	12.00	48.00	1.00
456.00	50.00	60.00	156.00	24.65	2.00	1.00	2.00	2.00	0.00	0.00	0.00	3.00	9.00	71.43	2.00	25.00	0.00	3.00	1.00	16.00	64.00	0.00
457.00	57.00	64.00	165.00	23.51	2.00	1.00	2.00	2.00	0.00	1.00	0.00	3.00	5.00	122.86	2.00	41.00	1.00	1.00	1.00	22.00	88.00	0.00
458.00	50.00	65.00	185.00	18.99	2.00	1.00	3.00	2.00	0.00	0.00	1.00	3.00	6.00	79.29	2.00	33.00	1.00	5.00	1.00	20.00	80.00	0.00
459.00	55.00	70.00	162.00	26.67	3.00	1.00	1.00	2.00	1.00	0.00	0.00	1.00	4.00	34.29	2.00	38.00	1.00	4.00	1.00	15.00	60.00	0.00
460.00	55.00	70.00	165.00	25.71	3.00	1.00	2.00	3.00	0.00	1.00	0.00	3.00	7.00	51.43	2.00	29.00	0.00	5.00	1.00	18.00	72.00	0.00
461.00	53.00	82.00	174.00	27.08	3.00	1.00	3.00	2.00	1.00	0.00	1.00	3.00	10.00	81.43	2.00	28.00	0.00	7.00	0.00	17.00	68.00	0.00
462.00	66.00	85.00	186.00	24.57	2.00	1.00	2.00	2.00	1.00	1.00	1.00	1.00	6.00	57.86	2.00	33.00	1.00	5.00	1.00	18.00	72.00	0.00

Subject	Age	Weight	Height	BMI	BMI_C	Residence	Education	Marital_Status	Smoking	Chronic_Dise	Gender	Health	Sitting_Time	PA_Min_Day	IPAQ_C	Dietary_Score	Dietary_C	PSQI_Total	Sleep_C	WHO5_Total	WHO5_Total100	WHO5_Class
**463.00**	20.00	53.00	167.00	19.00	2.00	1.00	1.00	1.00	0.00	0.00	0.00	2.00	5.50	12.86	1.00	34.00	1.00	10.00	0.00	2.00	8.00	1.00
**464.00**	18.00	82.00	188.00	23.20	2.00	1.00	1.00	1.00	0.00	0.00	1.00	3.00	6.00	38.57	1.00	27.00	0.00	1.00	1.00	13.00	52.00	0.00
**465.00**	21.00	91.00	188.00	25.75	3.00	1.00	2.00	1.00	0.00	0.00	1.00	3.00	3.50	53.57	1.00	36.00	1.00	6.00	0.00	15.00	60.00	0.00
**466.00**	23.00	60.00	163.00	22.58	2.00	1.00	1.00	1.00	0.00	0.00	0.00	3.00	12.00	2.14	1.00	24.00	0.00	13.00	0.00	6.00	24.00	1.00
**467.00**	21.00	56.00	152.00	24.24	2.00	1.00	1.00	1.00	0.00	0.00	0.00	3.00	3.50	17.14	1.00	34.00	1.00	4.00	1.00	16.00	64.00	0.00
**468.00**	23.00	53.00	153.00	22.64	2.00	1.00	2.00	1.00	0.00	0.00	0.00	2.00	7.00	19.29	1.00	21.00	0.00	3.00	1.00	8.00	32.00	1.00
**469.00**	20.00	55.00	159.00	21.76	2.00	1.00	2.00	1.00	0.00	0.00	0.00	3.00	10.00	21.43	1.00	27.00	0.00	9.00	0.00	10.00	40.00	1.00
**470.00**	23.00	68.00	184.00	20.09	2.00	0.00	2.00	1.00	0.00	0.00	1.00	3.00	12.00	21.43	1.00	26.00	0.00	3.00	1.00	18.00	72.00	0.00
**471.00**	21.00	60.00	163.00	22.58	2.00	1.00	2.00	1.00	0.00	0.00	0.00	3.00	11.00	17.14	1.00	25.00	0.00	10.00	0.00	11.00	44.00	1.00
**472.00**	21.00	54.00	157.00	21.91	2.00	0.00	2.00	1.00	0.00	0.00	0.00	3.00	4.00	42.86	1.00	27.00	0.00	9.00	0.00	8.00	32.00	1.00
**473.00**	19.00	63.00	174.00	20.81	2.00	1.00	2.00	1.00	0.00	0.00	1.00	3.00	9.00	38.57	1.00	22.00	0.00	6.00	0.00	12.00	48.00	1.00
**474.00**	23.00	60.00	168.00	21.26	2.00	1.00	2.00	1.00	0.00	0.00	1.00	3.00	4.50	21.43	1.00	23.00	0.00	11.00	0.00	15.00	60.00	0.00
**475.00**	23.00	49.00	159.00	19.38	2.00	1.00	2.00	1.00	0.00	0.00	0.00	3.00	6.50	42.86	1.00	34.00	1.00	8.00	0.00	14.00	56.00	0.00
**476.00**	22.00	45.00	154.00	18.97	2.00	1.00	2.00	1.00	1.00	0.00	0.00	3.00	11.00	18.57	1.00	14.00	0.00	4.00	1.00	13.00	52.00	0.00
**477.00**	20.00	50.00	163.00	18.82	2.00	0.00	2.00	1.00	1.00	1.00	1.00	1.00	5.50	12.14	1.00	20.00	0.00	7.00	0.00	9.00	36.00	1.00
**478.00**	20.00	73.00	175.00	23.84	2.00	1.00	2.00	1.00	1.00	1.00	1.00	2.00	7.00	10.00	1.00	26.00	0.00	4.00	1.00	15.00	60.00	0.00
**479.00**	21.00	44.00	155.00	18.31	1.00	1.00	2.00	1.00	0.00	0.00	0.00	2.00	8.00	42.86	1.00	30.00	1.00	1.00	1.00	13.00	52.00	0.00
**480.00**	19.00	110.00	188.00	31.12	4.00	1.00	2.00	1.00	0.00	0.00	1.00	3.00	1.00	7.86	1.00	19.00	0.00	5.00	1.00	14.00	56.00	0.00
**481.00**	22.00	63.00	136.00	34.06	4.00	1.00	2.00	1.00	0.00	0.00	0.00	3.00	2.50	25.43	1.00	26.00	0.00	7.00	0.00	16.00	64.00	0.00
**482.00**	20.00	73.00	166.00	26.49	3.00	1.00	2.00	1.00	0.00	0.00	0.00	1.00	4.50	0.00	1.00	20.00	0.00	6.00	0.00	6.00	24.00	1.00
**483.00**	23.00	92.80	178.00	29.29	3.00	1.00	2.00	1.00	1.00	0.00	1.00	3.00	6.50	49.29	1.00	27.00	0.00	5.00	1.00	15.00	60.00	0.00
**484.00**	23.00	74.00	176.00	23.89	2.00	0.00	2.00	1.00	0.00	0.00	1.00	3.00	1.50	0.00	1.00	27.00	0.00	4.00	1.00	18.00	72.00	0.00
**485.00**	23.00	71.00	173.00	23.72	2.00	1.00	2.00	1.00	0.00	0.00	1.00	3.00	8.00	31.43	1.00	27.00	0.00	7.00	0.00	7.00	28.00	1.00
**486.00**	18.00	95.00	177.00	30.32	4.00	1.00	2.00	1.00	0.00	0.00	1.00	2.00	8.00	94.29	1.00	19.00	0.00	7.00	0.00	17.00	68.00	0.00
**487.00**	22.00	70.00	161.00	27.01	3.00	1.00	1.00	1.00	0.00	0.00	0.00	3.00	10.00	0.00	1.00	14.00	0.00	8.00	0.00	12.00	48.00	1.00
**488.00**	23.00	74.00	170.00	25.61	3.00	1.00	2.00	1.00	1.00	0.00	0.00	3.00	11.00	0.00	1.00	35.00	1.00	6.00	0.00	10.00	40.00	1.00
**489.00**	23.00	51.00	159.00	20.17	2.00	0.00	1.00	1.00	0.00	0.00	0.00	3.00	5.50	0.00	1.00	28.00	0.00	4.00	1.00	11.00	44.00	1.00
**490.00**	19.00	78.00	178.00	24.62	2.00	1.00	1.00	1.00	1.00	0.00	1.00	3.00	7.00	55.71	1.00	26.00	0.00	7.00	0.00	13.00	52.00	0.00
**491.00**	23.00	55.00	160.00	21.48	2.00	1.00	1.00	1.00	0.00	0.00	0.00	1.00	11.00	8.57	1.00	27.00	0.00	8.00	0.00	9.00	36.00	1.00
**492.00**	21.00	55.00	155.00	22.89	2.00	0.00	1.00	1.00	0.00	0.00	0.00	3.00	8.00	0.00	1.00	21.00	0.00	13.00	0.00	4.00	16.00	1.00
**493.00**	19.00	50.00	154.00	21.08	2.00	0.00	1.00	1.00	0.00	0.00	0.00	3.00	6.00	6.43	1.00	22.00	0.00	4.00	1.00	9.00	36.00	1.00
**494.00**	20.00	55.00	160.00	21.48	2.00	0.00	1.00	1.00	1.00	1.00	1.00	2.00	1.50	35.71	1.00	19.00	0.00	10.00	0.00	14.00	56.00	0.00
**495.00**	21.00	75.00	179.50	23.28	2.00	1.00	1.00	1.00	1.00	1.00	1.00	3.00	7.00	10.71	1.00	21.00	0.00	8.00	0.00	9.00	36.00	1.00
**496.00**	20.00	69.00	184.00	20.38	2.00	1.00	2.00	1.00	0.00	0.00	1.00	3.00	11.00	4.29	1.00	26.00	0.00	3.00	1.00	14.00	56.00	0.00
**497.00**	21.00	70.00	177.00	22.34	2.00	1.00	2.00	1.00	1.00	1.00	1.00	3.00	8.00	13.86	1.00	18.00	0.00	5.00	1.00	15.00	60.00	0.00
**498.00**	20.00	43.00	158.00	17.22	1.00	1.00	1.00	1.00	0.00	0.00	0.00	3.00	1.00	42.86	1.00	23.00	0.00	2.00	1.00	13.00	52.00	0.00
**499.00**	23.00	65.00	157.00	26.37	3.00	0.00	2.00	1.00	0.00	0.00	0.00	3.00	9.00	38.57	1.00	28.00	0.00	2.00	1.00	14.00	56.00	0.00
**500.00**	21.00	55.00	167.00	19.72	2.00	0.00	2.00	1.00	1.00	1.00	0.00	3.00	11.00	11.43	1.00	22.00	0.00	8.00	0.00	10.00	40.00	1.00
**501.00**	21.00	63.00	170.00	21.80	2.00	1.00	2.00	1.00	1.00	0.00	1.00	2.00	11.00	25.71	1.00	19.00	0.00	4.00	1.00	11.00	44.00	1.00
**502.00**	22.00	66.00	167.00	23.67	2.00	1.00	2.00	1.00	0.00	0.00	0.00	2.00	9.00	0.00	1.00	23.00	0.00	3.00	1.00	14.00	56.00	0.00
**503.00**	22.00	81.00	180.00	25.00	2.00	1.00	2.00	1.00	1.00	0.00	1.00	3.00	11.00	12.86	1.00	14.00	0.00	9.00	0.00	7.00	28.00	1.00
**504.00**	22.00	54.00	157.00	21.91	2.00	1.00	2.00	1.00	0.00	0.00	0.00	3.00	9.00	0.00	1.00	17.00	0.00	9.00	0.00	10.00	40.00	1.00
**505.00**	21.00	72.00	170.00	24.91	2.00	1.00	2.00	1.00	0.00	0.00	0.00	3.00	3.50	17.14	1.00	20.00	0.00	8.00	0.00	13.00	52.00	0.00
**506.00**	20.00	58.00	156.00	23.83	2.00	1.00	2.00	1.00	1.00	0.00	0.00	3.00	10.00	22.86	1.00	27.00	0.00	7.00	0.00	17.00	68.00	0.00
**507.00**	21.00	60.00	166.00	21.77	2.00	1.00	2.00	1.00	0.00	0.00	0.00	3.00	5.00	36.43	1.00	41.00	1.00	2.00	1.00	13.00	52.00	0.00
**508.00**	22.00	68.00	166.00	24.68	2.00	1.00	2.00	1.00	0.00	0.00	0.00	2.00	7.00	19.29	1.00	28.00	0.00	3.00	1.00	11.00	44.00	1.00
**509.00**	21.00	53.00	167.00	19.00	2.00	1.00	2.00	1.00	0.00	0.00	0.00	3.00	9.00	2.14	1.00	30.00	1.00	5.00	1.00	10.00	40.00	1.00
**510.00**	23.00	58.00	160.00	22.66	2.00	1.00	2.00	2.00	0.00	0.00	0.00	3.00	7.00	0.00	1.00	28.00	0.00	7.00	0.00	13.00	52.00	0.00
**511.00**	20.00	50.00	159.00	19.78	2.00	1.00	2.00	1.00	0.00	0.00	0.00	2.00	10.00	51.43	1.00	25.00	0.00	11.00	0.00	11.00	44.00	1.00
**512.00**	21.00	69.00	174.00	22.79	2.00	1.00	2.00	1.00	1.00	0.00	1.00	3.00	5.00	18.57	1.00	17.00	0.00	3.00	1.00	14.00	56.00	0.00
**513.00**	22.00	60.00	165.00	22.04	2.00	0.00	2.00	1.00	0.00	0.00	0.00	2.00	6.50	15.00	1.00	29.00	0.00	6.00	0.00	12.00	48.00	1.00
**514.00**	22.00	60.00	166.00	21.77	2.00	1.00	2.00	1.00	0.00	0.00	0.00	3.00	5.00	40.71	1.00	27.00	0.00	7.00	0.00	5.00	20.00	1.00
**515.00**	23.00	67.00	170.00	23.18	2.00	0.00	2.00	1.00	0.00	0.00	1.00	3.00	6.00	95.71	1.00	27.00	0.00	9.00	0.00	17.00	68.00	0.00
**516.00**	22.00	75.00	166.00	27.22	3.00	0.00	2.00	2.00	0.00	0.00	0.00	3.00	10.00	42.86	1.00	36.00	1.00	0.00	1.00	12.00	48.00	1.00
**517.00**	21.00	61.00	164.00	22.68	2.00	1.00	2.00	1.00	1.00	0.00	0.00	1.00	5.50	8.57	1.00	36.00	1.00	1.00	1.00	14.00	56.00	0.00
**518.00**	20.00	58.00	165.00	21.30	2.00	1.00	2.00	1.00	0.00	0.00	0.00	2.00	9.00	4.29	1.00	38.00	1.00	4.00	1.00	12.00	48.00	1.00
**519.00**	23.00	62.00	162.00	23.62	2.00	1.00	3.00	2.00	0.00	0.00	0.00	2.00	7.00	17.14	1.00	29.00	0.00	4.00	1.00	13.00	52.00	0.00
**520.00**	22.00	124.00	200.00	31.00	4.00	1.00	1.00	1.00	0.00	0.00	1.00	3.00	10.00	25.71	1.00	33.00	1.00	5.00	1.00	17.00	68.00	0.00
**521.00**	22.00	107.00	170.00	37.02	4.00	1.00	1.00	2.00	0.00	0.00	0.00	3.00	7.00	25.71	1.00	28.00	0.00	5.00	1.00	13.00	52.00	0.00
**522.00**	21.00	56.00	136.00	30.28	4.00	1.00	1.00	1.00	0.00	0.00	1.00	3.00	12.00	25.71	1.00	18.00	0.00	2.00	1.00	18.00	72.00	0.00
**523.00**	19.00	105.00	179.00	32.77	4.00	1.00	1.00	1.00	0.00	0.00	1.00	3.00	9.00	20.00	1.00	18.00	0.00	5.00	1.00	16.00	64.00	0.00
**524.00**	20.00	110.00	161.00	42.44	4.00	0.00	2.00	3.00	0.00	0.00	1.00	1.00	1.00	0.00	1.00	20.00	0.00	14.00	0.00	8.00	32.00	1.00
**525.00**	23.00	107.00	176.00	34.54	4.00	0.00	2.00	1.00	0.00	0.00	1.00	3.00	11.00	0.00	1.00	27.00	0.00	5.00	1.00	8.00	32.00	1.00
**526.00**	20.00	90.00	173.00	30.07	4.00	1.00	2.00	1.00	0.00	0.00	1.00	3.00	11.00	0.00	1.00	20.00	0.00	2.00	1.00	16.00	64.00	0.00
**527.00**	20.00	100.00	178.00	31.56	4.00	1.00	2.00	1.00	1.00	0.00	1.00	3.00	1.50	8.57	1.00	20.00	0.00	7.00	0.00	9.00	36.00	1.00
**528.00**	22.00	80.00	162.00	30.48	4.00	0.00	2.00	1.00	1.00	0.00	0.00	3.00	9.00	0.00	1.00	24.00	0.00	4.00	1.00	10.00	40.00	1.00

Subject	Age	Weight	Height	BMI	BMI_C	Residence	Education	Marital_Status	Smoking	Chronic_Dise	Gender	Health	Sitting_Time	PA_Min_Day	IPAQ_C	Dietary_Score	Dietary_C	PSQI_Total	Sleep_C	WHO5_Total	WHO5_Total100	WHO5_Class
529.00	18.00	90.00	170.00	31.14	4.00	0.00	2.00	1.00	0.00	0.00	1.00	3.00	7.00	38.57	1.00	36.00	1.00	1.00	1.00	15.00	60.00	0.00
530.00	20.00	100.00	178.00	31.56	4.00	1.00	2.00	1.00	0.00	0.00	1.00	3.00	8.00	25.71	1.00	22.00	0.00	6.00	0.00	13.00	52.00	0.00
531.00	21.00	116.00	190.00	32.13	4.00	1.00	2.00	1.00	0.00	0.00	1.00	3.00	7.00	11.43	1.00	24.00	0.00	3.00	1.00	17.00	68.00	0.00
532.00	19.00	120.00	185.00	35.06	4.00	0.00	2.00	1.00	0.00	1.00	1.00	2.00	11.00	0.00	1.00	15.00	0.00	9.00	0.00	6.00	24.00	1.00
533.00	22.00	65.00	160.00	25.39	3.00	1.00	1.00	2.00	0.00	0.00	0.00	1.00	6.00	4.29	1.00	27.00	0.00	4.00	1.00	19.00	76.00	0.00
534.00	19.00	70.00	164.00	26.03	3.00	0.00	1.00	1.00	0.00	0.00	0.00	2.00	5.00	49.29	1.00	21.00	0.00	4.00	1.00	2.00	8.00	1.00
535.00	18.00	85.00	175.00	27.76	3.00	1.00	1.00	1.00	0.00	0.00	0.00	2.00	7.00	0.00	1.00	25.00	0.00	11.00	0.00	9.00	36.00	1.00
536.00	22.00	90.00	185.00	26.30	3.00	1.00	1.00	1.00	1.00	0.00	1.00	2.00	9.00	5.71	1.00	33.00	1.00	2.00	1.00	13.00	52.00	0.00
537.00	19.00	68.00	160.00	26.56	3.00	0.00	1.00	1.00	1.00	0.00	0.00	2.00	5.50	1.43	1.00	28.00	0.00	2.00	1.00	14.00	56.00	0.00
538.00	19.00	80.00	170.00	27.68	3.00	1.00	1.00	1.00	0.00	0.00	0.00	2.00	7.00	45.00	1.00	33.00	1.00	4.00	1.00	13.00	52.00	0.00
539.00	21.00	68.00	160.00	26.56	3.00	1.00	1.00	1.00	0.00	0.00	0.00	2.00	5.00	56.86	1.00	31.00	1.00	3.00	1.00	14.00	56.00	0.00
540.00	20.00	75.00	169.00	26.26	3.00	1.00	1.00	1.00	0.00	0.00	1.00	3.00	12.00	25.71	1.00	26.00	0.00	9.00	0.00	5.00	20.00	1.00
541.00	21.00	102.00	186.00	29.48	3.00	1.00	1.00	1.00	0.00	0.00	1.00	3.00	7.00	43.57	1.00	24.00	0.00	6.00	0.00	10.00	40.00	1.00
542.00	20.00	76.00	160.00	29.69	3.00	1.00	1.00	1.00	0.00	0.00	0.00	3.00	5.00	37.14	1.00	19.00	0.00	9.00	0.00	10.00	40.00	1.00
543.00	22.00	61.00	155.00	25.39	3.00	1.00	1.00	1.00	0.00	0.00	1.00	3.00	8.00	25.71	1.00	21.00	0.00	4.00	1.00	8.00	32.00	1.00
544.00	23.00	100.00	183.00	29.86	3.00	0.00	1.00	1.00	0.00	0.00	1.00	3.00	6.00	4.29	1.00	29.00	0.00	12.00	0.00	11.00	44.00	1.00
545.00	23.00	95.00	182.00	28.68	3.00	1.00	1.00	1.00	0.00	0.00	1.00	3.00	7.00	8.57	1.00	34.00	1.00	3.00	1.00	15.00	60.00	0.00
546.00	21.00	83.00	173.00	27.73	3.00	1.00	1.00	1.00	0.00	0.00	1.00	3.00	5.00	20.00	1.00	24.00	0.00	5.00	1.00	14.00	56.00	0.00
547.00	22.00	68.00	160.00	26.56	3.00	1.00	1.00	1.00	0.00	0.00	0.00	3.00	6.50	30.00	1.00	20.00	0.00	4.00	1.00	9.00	36.00	1.00
548.00	23.00	83.00	173.00	27.73	3.00	1.00	1.00	1.00	0.00	0.00	1.00	3.00	8.00	30.00	1.00	23.00	0.00	6.00	0.00	9.00	36.00	1.00
549.00	18.00	65.00	153.00	27.77	3.00	1.00	1.00	1.00	0.00	0.00	0.00	3.00	6.00	14.29	1.00	27.00	0.00	5.00	1.00	15.00	60.00	0.00
550.00	22.00	70.00	159.00	27.69	3.00	0.00	1.00	1.00	0.00	0.00	0.00	3.00	10.00	8.57	1.00	36.00	1.00	2.00	1.00	15.00	60.00	0.00
551.00	22.00	75.00	168.00	26.57	3.00	1.00	1.00	1.00	1.00	1.00	1.00	2.00	6.50	12.86	1.00	26.00	0.00	8.00	0.00	5.00	20.00	1.00
552.00	19.00	66.00	160.00	25.78	3.00	1.00	1.00	1.00	1.00	1.00	1.00	2.00	6.00	0.00	1.00	24.00	0.00	6.00	0.00	12.00	48.00	1.00
553.00	22.00	82.00	167.00	29.40	3.00	0.00	1.00	1.00	1.00	1.00	1.00	2.00	10.00	25.71	1.00	20.00	0.00	13.00	0.00	14.00	56.00	0.00
554.00	21.00	75.00	164.00	27.89	3.00	0.00	1.00	1.00	0.00	1.00	0.00	3.00	8.00	34.29	1.00	33.00	1.00	11.00	0.00	5.00	20.00	1.00
555.00	21.00	59.00	152.00	25.54	3.00	0.00	1.00	1.00	0.00	1.00	0.00	3.00	9.00	50.00	1.00	29.00	0.00	4.00	1.00	16.00	64.00	0.00
556.00	22.00	70.00	160.00	27.34	3.00	0.00	2.00	1.00	0.00	0.00	0.00	1.00	7.00	8.57	1.00	25.00	0.00	7.00	0.00	9.00	36.00	1.00
557.00	23.00	80.00	176.00	25.83	3.00	1.00	2.00	1.00	1.00	0.00	1.00	2.00	8.00	4.29	1.00	25.00	0.00	6.00	0.00	4.00	16.00	1.00
558.00	21.00	68.00	160.00	26.56	3.00	1.00	2.00	2.00	0.00	0.00	0.00	2.00	6.50	60.71	1.00	28.00	0.00	3.00	1.00	15.00	60.00	0.00
559.00	20.00	84.00	170.00	29.07	3.00	1.00	2.00	1.00	1.00	0.00	1.00	2.00	3.00	25.71	1.00	42.00	1.00	4.00	1.00	14.00	56.00	0.00
560.00	21.00	69.00	160.00	26.95	3.00	0.00	2.00	1.00	0.00	0.00	0.00	2.00	2.50	0.00	1.00	24.00	0.00	3.00	1.00	14.00	56.00	0.00
561.00	20.00	83.00	178.00	26.20	3.00	1.00	2.00	1.00	0.00	0.00	1.00	2.00	10.00	25.71	1.00	25.00	0.00	1.00	1.00	14.00	56.00	0.00
562.00	18.00	60.00	152.00	25.97	3.00	1.00	2.00	1.00	0.00	0.00	0.00	3.00	11.00	17.14	1.00	25.00	0.00	7.00	0.00	5.00	20.00	1.00
563.00	21.00	94.00	192.00	25.50	3.00	1.00	2.00	1.00	0.00	0.00	1.00	3.00	1.00	12.86	1.00	26.00	0.00	6.00	0.00	11.00	44.00	1.00
564.00	21.00	71.00	167.00	25.46	3.00	1.00	2.00	1.00	1.00	0.00	0.00	3.00	5.00	17.14	1.00	23.00	0.00	7.00	0.00	9.00	36.00	1.00
565.00	20.00	70.00	165.00	25.71	3.00	0.00	2.00	1.00	0.00	0.00	1.00	3.00	5.00	42.86	1.00	32.00	1.00	6.00	0.00	15.00	60.00	0.00
566.00	21.00	80.00	173.00	26.73	3.00	0.00	2.00	1.00	0.00	0.00	0.00	3.00	11.00	0.00	1.00	29.00	0.00	3.00	1.00	17.00	68.00	0.00
567.00	19.00	78.00	173.00	26.06	3.00	1.00	2.00	1.00	0.00	0.00	1.00	3.00	6.50	25.71	1.00	22.00	0.00	4.00	1.00	15.00	60.00	0.00
568.00	20.00	68.00	157.00	27.59	3.00	1.00	2.00	1.00	1.00	0.00	0.00	3.00	5.00	0.00	1.00	16.00	0.00	3.00	1.00	11.00	44.00	1.00
569.00	19.00	65.00	158.00	26.04	3.00	1.00	2.00	1.00	0.00	1.00	0.00	2.00	8.00	2.14	1.00	15.00	0.00	7.00	0.00	13.00	52.00	0.00
570.00	20.00	70.00	158.00	28.04	3.00	0.00	2.00	1.00	0.00	1.00	0.00	3.00	11.00	40.71	1.00	32.00	1.00	6.00	0.00	5.00	20.00	1.00
571.00	23.00	79.00	169.00	27.66	3.00	0.00	2.00	1.00	1.00	1.00	1.00	3.00	7.00	45.71	1.00	18.00	0.00	8.00	0.00	7.00	28.00	1.00
572.00	18.00	52.00	150.00	23.11	2.00	1.00	1.00	1.00	0.00	0.00	0.00	2.00	11.00	13.86	1.00	26.00	0.00	4.00	1.00	12.00	48.00	1.00
573.00	18.00	58.00	164.00	21.56	2.00	1.00	1.00	1.00	1.00	0.00	0.00	3.00	7.00	8.57	1.00	17.00	0.00	5.00	1.00	16.00	64.00	0.00
574.00	19.00	57.00	169.00	19.96	2.00	0.00	1.00	1.00	0.00	0.00	1.00	3.00	4.00	32.14	1.00	27.00	0.00	4.00	1.00	14.00	56.00	0.00
575.00	22.00	64.00	168.00	22.68	2.00	1.00	1.00	1.00	0.00	0.00	0.00	1.00	7.00	38.57	1.00	33.00	1.00	4.00	1.00	15.00	60.00	0.00
576.00	23.00	70.00	170.00	24.22	2.00	1.00	1.00	1.00	0.00	0.00	0.00	1.00	7.00	0.00	1.00	25.00	0.00	7.00	0.00	9.00	36.00	1.00
577.00	20.00	60.00	170.00	20.76	2.00	1.00	1.00	1.00	0.00	0.00	0.00	2.00	11.00	0.00	1.00	29.00	0.00	5.00	1.00	3.00	12.00	1.00
578.00	22.00	59.00	168.00	20.90	2.00	1.00	1.00	1.00	0.00	0.00	0.00	2.00	5.00	4.29	1.00	22.00	0.00	13.00	0.00	11.00	44.00	1.00
579.00	22.00	61.00	162.00	23.24	2.00	1.00	1.00	1.00	0.00	0.00	0.00	2.00	7.00	30.00	1.00	32.00	1.00	5.00	1.00	11.00	44.00	1.00
580.00	22.00	52.00	154.00	21.93	2.00	1.00	1.00	2.00	0.00	0.00	0.00	2.00	4.00	21.43	1.00	32.00	1.00	6.00	0.00	11.00	44.00	1.00
581.00	19.00	54.00	165.00	19.83	2.00	1.00	1.00	1.00	0.00	0.00	0.00	2.00	9.00	19.29	1.00	25.00	0.00	6.00	0.00	11.00	44.00	1.00
582.00	20.00	53.00	160.00	20.70	2.00	0.00	1.00	1.00	0.00	0.00	0.00	2.00	7.00	0.00	1.00	17.00	0.00	4.00	1.00	14.00	56.00	0.00
583.00	20.00	59.00	167.00	21.16	2.00	1.00	1.00	1.00	0.00	0.00	0.00	2.00	5.50	8.57	1.00	26.00	0.00	5.00	1.00	12.00	48.00	1.00
584.00	19.00	60.00	180.00	18.52	2.00	1.00	1.00	1.00	0.00	0.00	1.00	2.00	7.00	64.29	1.00	25.00	0.00	4.00	1.00	13.00	52.00	0.00
585.00	20.00	48.00	150.00	21.33	2.00	0.00	1.00	1.00	0.00	0.00	0.00	2.00	6.00	0.00	1.00	23.00	0.00	2.00	1.00	19.00	76.00	0.00
586.00	19.00	54.00	156.00	22.19	2.00	0.00	1.00	2.00	0.00	0.00	0.00	2.00	5.00	23.43	1.00	27.00	0.00	2.00	1.00	19.00	76.00	0.00
587.00	20.00	60.00	162.00	22.86	2.00	0.00	1.00	1.00	0.00	0.00	0.00	2.00	6.00	2.71	1.00	21.00	0.00	1.00	1.00	15.00	60.00	0.00
588.00	20.00	74.00	180.00	22.84	2.00	1.00	1.00	1.00	1.00	0.00	0.00	2.00	5.50	45.00	1.00	27.00	0.00	4.00	1.00	11.00	44.00	1.00
589.00	20.00	52.00	162.00	19.81	2.00	1.00	1.00	1.00	0.00	0.00	0.00	3.00	8.00	51.43	1.00	23.00	0.00	11.00	0.00	8.00	32.00	1.00
590.00	23.00	85.30	187.00	24.39	2.00	1.00	1.00	1.00	0.00	0.00	1.00	3.00	9.00	60.00	1.00	35.00	1.00	8.00	0.00	15.00	60.00	0.00
591.00	23.00	73.00	176.00	23.57	2.00	0.00	1.00	1.00	0.00	0.00	1.00	3.00	10.00	0.00	1.00	23.00	0.00	11.00	0.00	8.00	32.00	1.00
592.00	19.00	53.00	169.00	18.56	2.00	1.00	1.00	1.00	0.00	0.00	0.00	3.00	6.00	17.14	1.00	27.00	0.00	13.00	0.00	8.00	32.00	1.00
593.00	20.00	60.00	164.00	22.31	2.00	1.00	1.00	1.00	0.00	0.00	0.00	3.00	5.00	35.71	1.00	28.00	0.00	7.00	0.00	15.00	60.00	0.00
594.00	23.00	58.00	156.00	23.83	2.00	1.00	1.00	1.00	0.00	0.00	0.00	3.00	6.50	51.43	1.00	32.00	1.00	9.00	0.00	9.00	36.00	1.00

Subject	Age	Weight	Height	BMI	BMI_C	Residence	Education	Marital_Status	Smoking	Chronic_Dise	Gender	Health	Sitting_Time	PA_Min_Day	IPAQ_C	Dietary_Score	Dietary_C	PSQI_Total	Sleep_C	WHO5_Total	WHO5_Total100	WHO5_Class
595.00	20.00	57.00	167.00	20.44	2.00	1.00	1.00	1.00	0.00	0.00	0.00	3.00	11.00	12.86	1.00	32.00	1.00	4.00	1.00	10.00	40.00	1.00
596.00	20.00	71.00	183.00	21.20	2.00	0.00	1.00	1.00	0.00	0.00	1.00	3.00	12.00	28.57	1.00	30.00	1.00	12.00	0.00	15.00	60.00	0.00
597.00	21.00	55.00	164.00	20.45	2.00	1.00	1.00	1.00	0.00	0.00	0.00	3.00	3.00	24.29	1.00	34.00	1.00	11.00	0.00	13.00	52.00	0.00
598.00	23.00	53.00	167.00	19.00	2.00	1.00	1.00	1.00	0.00	0.00	1.00	3.00	8.00	21.43	1.00	19.00	0.00	8.00	0.00	10.00	40.00	1.00
599.00	19.00	60.00	170.00	20.76	2.00	1.00	1.00	1.00	0.00	0.00	1.00	3.00	4.50	10.71	1.00	26.00	0.00	5.00	1.00	9.00	36.00	1.00
600.00	22.00	56.00	169.00	19.61	2.00	0.00	1.00	1.00	0.00	0.00	0.00	3.00	4.00	0.00	1.00	28.00	0.00	5.00	1.00	15.00	60.00	0.00
601.00	18.00	57.00	155.00	23.73	2.00	1.00	1.00	1.00	0.00	0.00	0.00	3.00	7.00	8.57	1.00	32.00	1.00	5.00	1.00	16.00	64.00	0.00
602.00	21.00	60.00	170.00	20.76	2.00	1.00	1.00	1.00	0.00	0.00	1.00	3.00	1.50	31.43	1.00	26.00	0.00	5.00	1.00	15.00	60.00	0.00
603.00	21.00	60.00	170.00	20.76	2.00	1.00	1.00	1.00	0.00	0.00	0.00	3.00	7.00	0.00	1.00	25.00	0.00	7.00	0.00	10.00	40.00	1.00
604.00	19.00	58.00	167.00	20.80	2.00	1.00	1.00	2.00	0.00	0.00	0.00	3.00	6.00	0.00	1.00	24.00	0.00	4.00	1.00	17.00	68.00	0.00
605.00	20.00	56.00	160.00	21.88	2.00	1.00	1.00	1.00	0.00	0.00	0.00	3.00	10.00	4.29	1.00	26.00	0.00	6.00	0.00	10.00	40.00	1.00
606.00	18.00	53.00	159.00	20.96	2.00	1.00	1.00	1.00	0.00	0.00	0.00	3.00	7.00	17.14	1.00	23.00	0.00	5.00	1.00	12.00	48.00	1.00
607.00	19.00	62.00	162.00	23.62	2.00	0.00	1.00	1.00	0.00	0.00	0.00	3.00	3.00	30.00	1.00	32.00	1.00	4.00	1.00	12.00	48.00	1.00
608.00	20.00	61.00	165.00	22.41	2.00	1.00	1.00	1.00	0.00	0.00	0.00	3.00	4.00	8.57	1.00	28.00	0.00	3.00	1.00	15.00	60.00	0.00
609.00	21.00	65.00	175.00	21.22	2.00	1.00	1.00	2.00	0.00	0.00	0.00	3.00	7.00	37.14	1.00	31.00	1.00	1.00	1.00	15.00	60.00	0.00
610.00	21.00	50.00	155.00	20.81	2.00	0.00	1.00	1.00	0.00	0.00	0.00	3.00	4.00	7.86	1.00	38.00	1.00	4.00	1.00	17.00	68.00	0.00
611.00	20.00	65.00	176.00	20.98	2.00	0.00	1.00	1.00	0.00	0.00	1.00	3.00	1.50	4.29	1.00	31.00	1.00	6.00	0.00	17.00	68.00	0.00
612.00	21.00	75.00	176.00	24.21	2.00	1.00	1.00	1.00	0.00	0.00	1.00	3.00	4.50	36.43	1.00	33.00	1.00	8.00	0.00	16.00	64.00	0.00
613.00	23.00	53.00	153.00	22.64	2.00	1.00	1.00	2.00	0.00	0.00	0.00	3.00	6.00	0.00	1.00	34.00	1.00	6.00	0.00	13.00	52.00	0.00
614.00	22.00	53.00	159.00	20.96	2.00	0.00	1.00	1.00	0.00	0.00	0.00	3.00	9.00	32.86	1.00	28.00	0.00	5.00	1.00	11.00	44.00	1.00
615.00	18.00	54.00	164.00	20.08	2.00	1.00	1.00	1.00	0.00	0.00	0.00	3.00	4.00	32.86	1.00	31.00	1.00	4.00	1.00	16.00	64.00	0.00
616.00	22.00	55.00	160.00	21.48	2.00	1.00	1.00	1.00	0.00	0.00	0.00	3.00	8.00	25.71	1.00	27.00	0.00	3.00	1.00	16.00	64.00	0.00
617.00	22.00	50.00	150.00	22.22	2.00	1.00	1.00	2.00	0.00	0.00	0.00	3.00	5.50	7.14	1.00	24.00	0.00	4.00	1.00	13.00	52.00	0.00
618.00	23.00	70.00	172.00	23.66	2.00	0.00	1.00	1.00	0.00	0.00	1.00	3.00	10.00	25.71	1.00	28.00	0.00	0.00	1.00	17.00	68.00	0.00
619.00	18.00	52.00	157.00	21.10	2.00	1.00	1.00	1.00	0.00	0.00	0.00	3.00	3.50	0.00	1.00	32.00	1.00	3.00	1.00	17.00	68.00	0.00
620.00	22.00	48.00	158.00	19.23	2.00	1.00	1.00	1.00	0.00	1.00	0.00	1.00	9.00	15.00	1.00	8.00	0.00	6.00	0.00	10.00	40.00	1.00
621.00	19.00	76.00	175.00	24.82	2.00	0.00	1.00	1.00	1.00	1.00	1.00	1.00	4.50	8.57	1.00	31.00	1.00	1.00	1.00	16.00	64.00	0.00
622.00	21.00	56.00	162.00	21.34	2.00	1.00	1.00	1.00	0.00	1.00	0.00	2.00	9.00	38.57	1.00	27.00	0.00	6.00	0.00	6.00	24.00	1.00
623.00	21.00	62.00	167.00	22.23	2.00	0.00	1.00	1.00	1.00	1.00	1.00	2.00	10.00	0.00	1.00	23.00	0.00	7.00	0.00	9.00	36.00	1.00
624.00	20.00	49.00	155.00	20.40	2.00	1.00	1.00	1.00	1.00	1.00	0.00	2.00	10.00	12.86	1.00	23.00	0.00	10.00	0.00	9.00	36.00	1.00
625.00	23.00	65.00	163.00	24.46	2.00	1.00	1.00	1.00	0.00	1.00	0.00	2.00	11.00	9.00	1.00	24.00	0.00	3.00	1.00	10.00	40.00	1.00
626.00	19.00	58.00	175.00	18.94	2.00	1.00	1.00	1.00	1.00	1.00	1.00	2.00	5.50	0.00	1.00	19.00	0.00	5.00	1.00	9.00	36.00	1.00
627.00	23.00	70.00	169.00	24.51	2.00	1.00	1.00	1.00	1.00	1.00	1.00	2.00	8.00	41.43	1.00	26.00	0.00	8.00	0.00	11.00	44.00	1.00
628.00	22.00	60.00	173.00	20.05	2.00	1.00	1.00	1.00	1.00	1.00	1.00	2.00	8.00	47.14	1.00	33.00	1.00	7.00	0.00	12.00	48.00	1.00
629.00	20.00	60.00	172.00	20.28	2.00	1.00	1.00	1.00	1.00	1.00	1.00	2.00	11.00	32.14	1.00	24.00	0.00	3.00	1.00	14.00	56.00	0.00
630.00	20.00	54.00	158.00	21.63	2.00	0.00	1.00	1.00	1.00	1.00	0.00	2.00	6.50	17.14	1.00	16.00	0.00	8.00	0.00	16.00	64.00	0.00
631.00	22.00	75.00	182.00	22.64	2.00	1.00	1.00	1.00	1.00	1.00	1.00	2.00	6.00	38.57	1.00	34.00	1.00	1.00	1.00	15.00	60.00	0.00
632.00	21.00	65.00	164.00	24.17	2.00	1.00	1.00	1.00	0.00	1.00	0.00	3.00	11.00	25.71	1.00	18.00	0.00	6.00	0.00	10.00	40.00	1.00
633.00	20.00	46.00	157.00	18.66	2.00	0.00	1.00	1.00	0.00	1.00	0.00	3.00	6.00	10.71	1.00	25.00	0.00	13.00	0.00	12.00	48.00	1.00
634.00	21.00	64.00	168.00	22.68	2.00	0.00	1.00	1.00	0.00	1.00	0.00	3.00	6.00	34.29	1.00	28.00	0.00	5.00	1.00	17.00	68.00	0.00
635.00	19.00	61.00	163.00	22.96	2.00	1.00	1.00	1.00	1.00	1.00	0.00	3.00	5.00	8.57	1.00	31.00	1.00	2.00	1.00	16.00	64.00	0.00
636.00	23.00	64.00	160.00	25.00	2.00	1.00	2.00	1.00	1.00	0.00	1.00	1.00	3.00	34.29	1.00	21.00	0.00	4.00	1.00	10.00	40.00	1.00
637.00	18.00	58.00	158.00	23.23	2.00	1.00	2.00	1.00	1.00	0.00	0.00	1.00	11.00	14.29	1.00	25.00	0.00	5.00	1.00	19.00	76.00	0.00
638.00	21.00	60.00	165.00	22.04	2.00	1.00	2.00	2.00	0.00	0.00	0.00	2.00	7.00	25.71	1.00	20.00	0.00	6.00	0.00	5.00	20.00	1.00
639.00	20.00	55.00	160.00	21.48	2.00	1.00	2.00	1.00	0.00	0.00	0.00	2.00	9.00	28.57	1.00	21.00	0.00	5.00	1.00	12.00	48.00	1.00
640.00	18.00	59.00	156.00	24.24	2.00	1.00	2.00	1.00	0.00	0.00	1.00	3.00	9.00	2.86	1.00	21.00	0.00	4.00	1.00	12.00	48.00	1.00
641.00	21.00	55.00	162.50	20.83	2.00	1.00	2.00	1.00	0.00	0.00	0.00	3.00	8.00	32.14	1.00	33.00	1.00	7.00	0.00	13.00	52.00	0.00
642.00	19.00	62.00	165.00	22.77	2.00	0.00	2.00	1.00	0.00	0.00	0.00	3.00	10.00	14.29	1.00	35.00	1.00	6.00	0.00	13.00	52.00	0.00
643.00	23.00	55.00	170.00	19.03	2.00	1.00	2.00	1.00	0.00	0.00	0.00	3.00	7.00	4.29	1.00	27.00	0.00	2.00	1.00	14.00	56.00	0.00
644.00	19.00	47.50	160.00	18.55	2.00	1.00	2.00	2.00	0.00	0.00	0.00	3.00	10.00	14.29	1.00	30.00	1.00	4.00	1.00	19.00	76.00	0.00
645.00	20.00	66.00	170.00	22.84	2.00	1.00	2.00	1.00	0.00	0.00	0.00	3.00	1.00	8.57	1.00	23.00	0.00	1.00	1.00	13.00	52.00	0.00
646.00	19.00	51.00	164.00	18.96	2.00	0.00	2.00	1.00	0.00	1.00	0.00	1.00	6.50	22.86	1.00	26.00	0.00	7.00	0.00	5.00	20.00	1.00
647.00	20.00	50.00	161.00	19.29	2.00	0.00	2.00	1.00	0.00	0.00	0.00	1.00	11.00	2.14	1.00	20.00	0.00	9.00	0.00	6.00	24.00	1.00
648.00	22.00	84.00	190.00	23.27	2.00	1.00	2.00	1.00	0.00	0.00	1.00	1.00	6.00	17.14	1.00	28.00	0.00	4.00	1.00	8.00	32.00	1.00
649.00	19.00	48.00	156.00	19.72	2.00	1.00	2.00	1.00	0.00	0.00	0.00	1.00	6.50	0.00	1.00	20.00	0.00	1.00	1.00	11.00	44.00	1.00
650.00	21.00	72.00	176.00	23.24	2.00	1.00	2.00	1.00	1.00	0.00	1.00	2.00	11.00	17.14	1.00	29.00	0.00	12.00	0.00	6.00	24.00	1.00
651.00	22.00	60.00	162.00	22.86	2.00	1.00	2.00	1.00	0.00	0.00	0.00	2.00	10.00	17.14	1.00	19.00	0.00	12.00	0.00	5.00	20.00	1.00
652.00	19.00	53.00	153.00	22.64	2.00	1.00	2.00	1.00	0.00	0.00	0.00	2.00	11.00	0.00	1.00	17.00	0.00	6.00	0.00	6.00	24.00	1.00
653.00	22.00	60.00	156.00	24.65	2.00	0.00	2.00	2.00	0.00	0.00	0.00	2.00	11.00	4.29	1.00	24.00	0.00	6.00	0.00	7.00	28.00	1.00
654.00	20.00	50.00	157.00	20.28	2.00	0.00	2.00	1.00	0.00	0.00	0.00	2.00	10.00	35.71	1.00	28.00	0.00	4.00	1.00	7.00	28.00	1.00
655.00	21.00	57.00	162.00	21.72	2.00	1.00	2.00	1.00	0.00	0.00	0.00	2.00	11.00	30.00	1.00	26.00	0.00	5.00	1.00	8.00	32.00	1.00
656.00	20.00	60.00	162.00	22.86	2.00	1.00	2.00	1.00	0.00	0.00	0.00	2.00	10.00	49.71	1.00	29.00	0.00	9.00	0.00	12.00	48.00	1.00
657.00	20.00	47.00	153.00	20.08	2.00	1.00	2.00	1.00	0.00	0.00	0.00	2.00	6.00	12.86	1.00	27.00	0.00	5.00	1.00	12.00	48.00	1.00
658.00	23.00	72.00	173.00	24.06	2.00	1.00	2.00	1.00	1.00	0.00	1.00	2.00	5.00	34.29	1.00	28.00	0.00	1.00	1.00	19.00	76.00	0.00
659.00	20.00	48.00	156.00	19.72	2.00	1.00	2.00	1.00	0.00	0.00	0.00	2.00	8.00	30.00	1.00	25.00	0.00	3.00	1.00	18.00	72.00	0.00
660.00	19.00	54.00	164.00	20.08	2.00	1.00	2.00	1.00	1.00	0.00	0.00	3.00	9.00	35.00	1.00	20.00	0.00	10.00	0.00	6.00	24.00	1.00

Subject	Age	Weight	Height	BMI	BMI_C	Residence	Education	Marital_Status	Smoking	Chronic_Dise	Gender	Health	Sitting_Time	PA_Min_Day	IPAQ_C	Dietary_Score	Dietary_C	PSQI_Total	Sleep_C	WHO5_Total	WHO5_Total100	WHO5_Class
661.00	21.00	55.00	160.00	21.48	2.00	0.00	2.00	1.00	0.00	0.00	0.00	3.00	6.50	8.57	1.00	31.00	1.00	6.00	0.00	6.00	24.00	1.00
662.00	21.00	52.00	160.00	20.31	2.00	0.00	2.00	1.00	1.00	0.00	0.00	3.00	11.00	2.14	1.00	30.00	1.00	6.00	0.00	5.00	20.00	1.00
663.00	20.00	65.00	165.00	23.88	2.00	1.00	2.00	1.00	0.00	0.00	0.00	3.00	9.00	1.43	1.00	31.00	1.00	6.00	0.00	3.00	12.00	1.00
664.00	20.00	76.00	175.00	24.82	2.00	1.00	2.00	1.00	0.00	0.00	1.00	3.00	6.50	12.86	1.00	23.00	0.00	7.00	0.00	6.00	24.00	1.00
665.00	22.00	60.00	162.00	22.86	2.00	1.00	2.00	2.00	0.00	0.00	0.00	3.00	8.00	2.14	1.00	21.00	0.00	4.00	1.00	4.00	16.00	1.00
666.00	22.00	60.00	170.00	20.76	2.00	1.00	2.00	1.00	0.00	0.00	0.00	3.00	8.00	45.71	1.00	29.00	0.00	10.00	0.00	5.00	20.00	1.00
667.00	22.00	69.00	168.00	24.45	2.00	1.00	2.00	1.00	0.00	0.00	0.00	3.00	6.00	36.43	1.00	24.00	0.00	7.00	0.00	10.00	40.00	1.00
668.00	21.00	50.00	150.00	22.22	2.00	0.00	2.00	1.00	0.00	0.00	0.00	3.00	10.00	21.43	1.00	28.00	0.00	11.00	0.00	4.00	16.00	1.00
669.00	22.00	60.00	160.00	23.44	2.00	1.00	2.00	1.00	0.00	0.00	0.00	3.00	9.00	15.71	1.00	21.00	0.00	9.00	0.00	10.00	40.00	1.00
670.00	22.00	57.00	166.00	20.69	2.00	1.00	2.00	1.00	1.00	0.00	0.00	3.00	8.00	25.71	1.00	34.00	1.00	12.00	0.00	9.00	36.00	1.00
671.00	20.00	60.00	164.00	22.31	2.00	1.00	2.00	1.00	0.00	0.00	0.00	3.00	4.00	8.57	1.00	21.00	0.00	7.00	0.00	9.00	36.00	1.00
672.00	22.00	43.00	150.00	19.11	2.00	0.00	2.00	1.00	0.00	0.00	0.00	3.00	9.00	0.00	1.00	20.00	0.00	4.00	1.00	15.00	60.00	0.00
673.00	22.00	56.00	162.00	21.34	2.00	1.00	2.00	1.00	0.00	0.00	0.00	3.00	10.00	0.00	1.00	29.00	0.00	7.00	0.00	12.00	48.00	1.00
674.00	19.00	51.00	162.00	19.43	2.00	1.00	2.00	1.00	1.00	0.00	0.00	3.00	9.00	28.57	1.00	19.00	0.00	6.00	0.00	9.00	36.00	1.00
675.00	20.00	72.00	176.00	23.24	2.00	0.00	2.00	1.00	1.00	0.00	1.00	3.00	6.00	0.00	1.00	27.00	0.00	6.00	0.00	14.00	56.00	0.00
676.00	22.00	55.00	157.00	22.31	2.00	1.00	2.00	1.00	1.00	0.00	0.00	3.00	10.00	48.57	1.00	34.00	1.00	4.00	1.00	12.00	48.00	1.00
677.00	20.00	71.00	174.00	23.45	2.00	1.00	2.00	1.00	0.00	0.00	1.00	3.00	11.00	10.71	1.00	23.00	0.00	6.00	0.00	15.00	60.00	0.00
678.00	18.00	54.00	165.00	19.83	2.00	1.00	2.00	1.00	0.00	0.00	0.00	3.00	8.00	0.00	1.00	24.00	0.00	4.00	1.00	13.00	52.00	0.00
679.00	22.00	65.00	172.00	21.97	2.00	1.00	2.00	1.00	0.00	0.00	1.00	3.00	4.50	51.43	1.00	24.00	0.00	2.00	1.00	17.00	68.00	0.00
680.00	20.00	70.00	170.00	24.22	2.00	1.00	2.00	1.00	0.00	0.00	1.00	3.00	4.00	10.71	1.00	27.00	0.00	4.00	1.00	17.00	68.00	0.00
681.00	20.00	68.00	177.00	21.71	2.00	1.00	2.00	1.00	1.00	0.00	1.00	3.00	7.00	51.43	1.00	23.00	0.00	5.00	1.00	17.00	68.00	0.00
682.00	18.00	54.00	158.00	21.63	2.00	1.00	2.00	1.00	0.00	0.00	0.00	3.00	11.00	34.29	1.00	16.00	0.00	4.00	1.00	16.00	64.00	0.00
683.00	19.00	54.00	155.00	22.48	2.00	1.00	2.00	1.00	0.00	0.00	0.00	3.00	6.00	22.86	1.00	25.00	0.00	0.00	1.00	17.00	68.00	0.00
684.00	20.00	57.00	172.00	19.27	2.00	1.00	2.00	1.00	1.00	0.00	1.00	3.00	6.50	24.29	1.00	21.00	0.00	4.00	1.00	13.00	52.00	0.00
685.00	21.00	58.00	160.00	22.66	2.00	1.00	2.00	1.00	0.00	0.00	1.00	3.00	5.00	21.43	1.00	28.00	0.00	3.00	1.00	17.00	68.00	0.00
686.00	23.00	55.00	156.00	22.60	2.00	1.00	2.00	1.00	0.00	0.00	0.00	3.00	8.00	20.71	1.00	24.00	0.00	3.00	1.00	15.00	60.00	0.00
687.00	20.00	60.00	165.00	22.04	2.00	1.00	2.00	1.00	0.00	0.00	0.00	3.00	4.00	32.14	1.00	29.00	0.00	6.00	0.00	12.00	48.00	1.00
688.00	22.00	60.00	165.00	22.04	2.00	1.00	2.00	2.00	0.00	0.00	0.00	3.00	5.00	4.29	1.00	27.00	0.00	5.00	1.00	16.00	64.00	0.00
689.00	21.00	53.00	158.00	21.23	2.00	1.00	2.00	1.00	0.00	0.00	0.00	3.00	6.00	0.00	1.00	24.00	0.00	0.00	1.00	16.00	64.00	0.00
690.00	21.00	55.00	168.00	19.49	2.00	1.00	2.00	1.00	0.00	0.00	0.00	3.00	6.00	17.14	1.00	23.00	0.00	4.00	1.00	16.00	64.00	0.00
691.00	21.00	49.00	157.00	19.88	2.00	1.00	2.00	1.00	0.00	0.00	0.00	3.00	4.00	25.71	1.00	26.00	0.00	3.00	1.00	16.00	64.00	0.00
692.00	23.00	64.00	176.00	20.66	2.00	1.00	2.00	1.00	0.00	0.00	1.00	3.00	7.00	0.00	1.00	24.00	0.00	3.00	1.00	11.00	44.00	1.00
693.00	19.00	79.00	178.00	24.93	2.00	1.00	2.00	1.00	1.00	0.00	1.00	3.00	6.00	25.71	1.00	31.00	1.00	3.00	1.00	13.00	52.00	0.00
694.00	21.00	70.00	173.00	23.39	2.00	1.00	2.00	1.00	1.00	1.00	1.00	3.00	8.00	28.57	1.00	22.00	0.00	10.00	0.00	10.00	40.00	1.00
695.00	19.00	77.00	187.00	22.02	2.00	1.00	2.00	1.00	1.00	1.00	1.00	3.00	6.00	42.86	1.00	27.00	0.00	0.00	1.00	14.00	56.00	0.00
696.00	22.00	62.00	165.00	22.77	2.00	1.00	3.00	1.00	0.00	0.00	0.00	3.00	8.00	60.00	1.00	34.00	1.00	4.00	1.00	9.00	36.00	1.00
697.00	23.00	57.00	161.00	21.99	2.00	1.00	3.00	1.00	0.00	0.00	0.00	3.00	5.00	17.14	1.00	28.00	0.00	7.00	0.00	10.00	40.00	1.00
698.00	22.00	45.00	162.00	17.15	1.00	0.00	1.00	1.00	0.00	1.00	0.00	2.00	3.50	51.43	1.00	20.00	0.00	14.00	0.00	4.00	16.00	1.00
699.00	22.00	46.00	160.00	17.97	1.00	1.00	1.00	1.00	0.00	0.00	0.00	1.00	10.00	21.43	1.00	25.00	0.00	5.00	1.00	12.00	48.00	1.00
700.00	18.00	52.00	170.00	17.99	1.00	1.00	1.00	1.00	0.00	0.00	0.00	2.00	11.00	2.14	1.00	18.00	0.00	7.00	0.00	9.00	36.00	1.00
701.00	22.00	49.00	163.00	18.44	1.00	0.00	1.00	1.00	0.00	0.00	0.00	2.00	2.50	0.00	1.00	25.00	0.00	4.00	1.00	11.00	44.00	1.00
702.00	21.00	49.00	167.00	17.57	1.00	1.00	1.00	1.00	1.00	0.00	0.00	2.00	8.00	0.00	1.00	23.00	0.00	3.00	1.00	13.00	52.00	0.00
703.00	20.00	44.50	165.00	16.35	1.00	0.00	1.00	1.00	0.00	0.00	0.00	2.00	10.00	24.29	1.00	24.00	0.00	3.00	1.00	18.00	72.00	0.00
704.00	20.00	51.00	170.00	17.65	1.00	1.00	1.00	1.00	0.00	0.00	0.00	2.00	3.00	30.00	1.00	23.00	0.00	4.00	1.00	15.00	60.00	0.00
705.00	21.00	50.00	165.00	18.37	1.00	0.00	1.00	1.00	0.00	0.00	0.00	3.00	11.00	9.29	1.00	23.00	0.00	7.00	0.00	12.00	48.00	1.00
706.00	21.00	46.00	163.00	17.31	1.00	1.00	1.00	1.00	0.00	0.00	0.00	3.00	10.00	44.29	1.00	24.00	0.00	6.00	0.00	10.00	40.00	1.00
707.00	22.00	58.00	178.00	18.31	1.00	0.00	1.00	1.00	0.00	0.00	1.00	3.00	7.00	11.43	1.00	28.00	0.00	2.00	1.00	17.00	68.00	0.00
708.00	19.00	47.00	172.00	15.89	1.00	1.00	1.00	1.00	0.00	1.00	0.00	1.00	8.00	0.00	1.00	28.00	0.00	8.00	0.00	10.00	40.00	1.00
709.00	22.00	55.00	174.00	18.17	1.00	1.00	1.00	1.00	1.00	1.00	1.00	1.00	10.00	0.71	1.00	27.00	0.00	4.00	1.00	15.00	60.00	0.00
710.00	22.00	43.00	155.00	17.90	1.00	0.00	2.00	1.00	0.00	0.00	0.00	1.00	11.00	0.00	1.00	15.00	0.00	7.00	0.00	3.00	12.00	1.00
711.00	21.00	46.00	169.00	16.11	1.00	1.00	2.00	1.00	1.00	0.00	0.00	1.00	8.00	8.57	1.00	31.00	1.00	12.00	0.00	8.00	32.00	1.00
712.00	21.00	46.00	165.00	16.90	1.00	1.00	2.00	1.00	0.00	0.00	0.00	2.00	2.50	2.14	1.00	29.00	0.00	12.00	0.00	3.00	12.00	1.00
713.00	20.00	47.00	169.00	16.46	1.00	1.00	2.00	1.00	0.00	0.00	0.00	2.00	4.50	43.57	1.00	32.00	1.00	3.00	1.00	15.00	60.00	0.00
714.00	20.00	50.00	165.00	18.37	1.00	1.00	2.00	1.00	1.00	0.00	0.00	2.00	11.00	4.29	1.00	25.00	0.00	4.00	1.00	14.00	56.00	0.00
715.00	22.00	48.00	165.00	17.63	1.00	1.00	2.00	1.00	1.00	0.00	0.00	2.00	10.00	8.57	1.00	29.00	0.00	5.00	1.00	12.00	48.00	1.00
716.00	23.00	45.00	160.00	17.58	1.00	1.00	2.00	1.00	0.00	0.00	1.00	2.00	6.00	21.43	1.00	29.00	0.00	7.00	0.00	12.00	48.00	1.00
717.00	21.00	44.00	165.00	16.16	1.00	1.00	2.00	1.00	0.00	0.00	0.00	3.00	7.00	25.71	1.00	38.00	1.00	90.21	0.00	6.00	24.00	1.00
718.00	22.00	49.00	168.00	17.36	1.00	1.00	2.00	1.00	0.00	0.00	0.00	3.00	5.00	44.29	1.00	30.00	1.00	7.00	0.00	14.00	56.00	0.00
719.00	19.00	40.00	148.00	18.26	1.00	1.00	2.00	1.00	0.00	0.00	0.00	3.00	9.00	34.29	1.00	25.00	0.00	8.00	0.00	9.00	36.00	1.00
720.00	20.00	51.00	171.00	17.44	1.00	1.00	2.00	1.00	0.00	0.00	0.00	3.00	6.00	25.71	1.00	29.00	0.00	8.00	0.00	18.00	72.00	0.00
721.00	20.00	50.00	177.00	15.96	1.00	1.00	2.00	1.00	0.00	0.00	0.00	3.00	2.50	0.00	1.00	27.00	0.00	6.00	0.00	16.00	64.00	0.00
722.00	21.00	48.00	162.00	18.29	1.00	1.00	2.00	1.00	0.00	1.00	0.00	1.00	10.00	0.00	1.00	20.00	0.00	8.00	0.00	6.00	24.00	1.00
723.00	22.00	50.00	172.00	16.90	1.00	1.00	3.00	1.00	0.00	0.00	0.00	2.00	8.00	0.00	1.00	26.00	0.00	8.00	0.00	11.00	44.00	1.00
724.00	21.00	80.00	162.00	30.48	4.00	1.00	2.00	1.00	0.00	0.00	0.00	3.00	8.00	63.57	1.00	36.00	1.00	1.00	1.00	12.00	48.00	1.00
725.00	23.00	64.00	157.00	25.96	3.00	1.00	2.00	1.00	0.00	0.00	0.00	3.00	6.00	8.57	1.00	23.00	0.00	5.00	1.00	15.00	60.00	0.00
726.00	22.00	49.00	160.00	19.14	2.00	0.00	2.00	1.00	0.00	0.00	0.00	2.00	6.50	2.14	1.00	31.00	1.00	5.00	1.00	15.00	60.00	0.00

Subject	Age	Weight	Height	BMI	BMI_C	Residence	Education	Marital_Status	Smoking	Chronic_Dise	Gender	Health	Sitting_Time	PA_Min_Day	IPAQ_C	Dietary_Score	Dietary_C	PSQI_Total	Sleep_C	WHO5_Total	WHO5_Total100	WHO5_Class
**727.00**	20.00	67.00	176.00	21.63	2.00	0.00	2.00	1.00	0.00	0.00	1.00	2.00	6.00	34.29	1.00	22.00	0.00	9.00	0.00	12.00	48.00	1.00
**728.00**	23.00	50.00	157.00	20.28	2.00	1.00	2.00	1.00	0.00	0.00	0.00	2.00	5.00	17.14	1.00	29.00	0.00	6.00	0.00	12.00	48.00	1.00
**729.00**	20.00	70.00	180.00	21.60	2.00	1.00	2.00	1.00	0.00	0.00	1.00	3.00	10.00	2.86	1.00	32.00	1.00	10.00	0.00	6.00	24.00	1.00
**730.00**	21.00	68.00	166.00	24.68	2.00	1.00	2.00	1.00	0.00	0.00	0.00	3.00	6.00	51.43	1.00	18.00	0.00	7.00	0.00	9.00	36.00	1.00
**731.00**	20.00	68.00	170.00	23.53	2.00	1.00	2.00	1.00	0.00	0.00	0.00	3.00	7.00	0.00	1.00	21.00	0.00	5.00	1.00	9.00	36.00	1.00
**732.00**	23.00	58.00	163.00	21.83	2.00	1.00	2.00	1.00	0.00	0.00	0.00	3.00	3.00	11.43	1.00	31.00	1.00	4.00	1.00	13.00	52.00	0.00
**733.00**	20.00	80.00	183.00	23.89	2.00	1.00	2.00	1.00	1.00	0.00	1.00	3.00	2.00	38.57	1.00	35.00	1.00	4.00	1.00	16.00	64.00	0.00
**734.00**	23.00	55.00	157.00	22.31	2.00	1.00	2.00	1.00	0.00	0.00	0.00	3.00	6.50	2.86	1.00	16.00	0.00	5.00	1.00	16.00	64.00	0.00
**735.00**	20.00	50.00	158.00	20.03	2.00	1.00	2.00	1.00	0.00	1.00	0.00	3.00	6.50	40.00	1.00	30.00	1.00	3.00	1.00	17.00	68.00	0.00
**736.00**	23.00	88.00	165.00	32.32	4.00	0.00	2.00	1.00	0.00	0.00	0.00	3.00	8.00	10.00	1.00	30.00	1.00	5.00	1.00	6.00	24.00	1.00
**737.00**	20.00	103.00	175.00	33.63	4.00	1.00	2.00	1.00	0.00	0.00	1.00	3.00	1.00	34.86	1.00	20.00	0.00	5.00	1.00	15.00	60.00	0.00
**738.00**	18.00	65.00	156.00	26.71	3.00	1.00	1.00	1.00	0.00	0.00	0.00	3.00	7.00	1.71	1.00	27.00	0.00	10.00	0.00	18.00	72.00	0.00
**739.00**	22.00	70.00	155.00	29.14	3.00	0.00	2.00	1.00	1.00	0.00	0.00	1.00	11.00	8.57	1.00	19.00	0.00	7.00	0.00	9.00	36.00	1.00
**740.00**	19.00	74.00	170.00	25.61	3.00	1.00	2.00	1.00	1.00	0.00	1.00	2.00	6.00	12.86	1.00	22.00	0.00	4.00	1.00	13.00	52.00	0.00
**741.00**	22.00	83.00	180.00	25.62	3.00	0.00	3.00	1.00	0.00	0.00	1.00	3.00	11.00	51.43	1.00	23.00	0.00	5.00	1.00	14.00	56.00	0.00
**742.00**	19.00	50.00	156.00	20.55	2.00	1.00	1.00	1.00	0.00	0.00	0.00	1.00	7.00	21.43	1.00	25.00	0.00	7.00	0.00	9.00	36.00	1.00
**743.00**	20.00	59.00	167.00	21.16	2.00	0.00	1.00	1.00	0.00	0.00	1.00	3.00	4.50	25.71	1.00	19.00	0.00	4.00	1.00	16.00	64.00	0.00
**744.00**	19.00	60.00	165.00	22.04	2.00	1.00	1.00	2.00	0.00	0.00	0.00	1.00	8.00	8.86	1.00	23.00	0.00	9.00	0.00	7.00	28.00	1.00
**745.00**	20.00	54.00	160.00	21.09	2.00	1.00	1.00	2.00	0.00	0.00	0.00	3.00	10.00	12.57	1.00	20.00	0.00	11.00	0.00	7.00	28.00	1.00
**746.00**	22.00	56.00	154.00	23.61	2.00	0.00	1.00	1.00	0.00	1.00	0.00	1.00	10.00	0.00	1.00	23.00	0.00	4.00	1.00	10.00	40.00	1.00
**747.00**	18.00	64.00	170.00	22.15	2.00	1.00	1.00	1.00	0.00	1.00	0.00	2.00	11.00	32.14	1.00	14.00	0.00	9.00	0.00	9.00	36.00	1.00
**748.00**	21.00	68.00	180.00	20.99	2.00	1.00	1.00	1.00	1.00	1.00	1.00	2.00	6.00	10.00	1.00	20.00	0.00	11.00	0.00	11.00	44.00	1.00
**749.00**	22.00	56.00	156.00	23.01	2.00	1.00	2.00	1.00	1.00	0.00	0.00	2.00	11.00	51.43	1.00	15.00	0.00	6.00	0.00	4.00	16.00	1.00
**750.00**	22.00	47.00	159.00	18.59	2.00	0.00	2.00	1.00	0.00	0.00	0.00	2.00	7.00	3.14	1.00	22.00	0.00	5.00	1.00	12.00	48.00	1.00
**751.00**	21.00	56.00	167.00	20.08	2.00	0.00	2.00	1.00	1.00	0.00	0.00	3.00	9.00	7.14	1.00	17.00	0.00	8.00	0.00	15.00	60.00	0.00
**752.00**	21.00	47.00	155.00	19.56	2.00	1.00	2.00	1.00	0.00	0.00	0.00	3.00	8.00	21.43	1.00	32.00	1.00	7.00	0.00	9.00	36.00	1.00
**753.00**	23.00	69.00	170.00	23.88	2.00	0.00	2.00	1.00	0.00	0.00	1.00	3.00	10.00	25.71	1.00	24.00	0.00	6.00	0.00	15.00	60.00	0.00
**754.00**	20.00	64.00	165.00	23.51	2.00	1.00	2.00	1.00	0.00	0.00	0.00	3.00	5.00	27.86	1.00	22.00	0.00	6.00	0.00	10.00	40.00	1.00
**755.00**	20.00	68.00	175.00	22.20	2.00	0.00	2.00	1.00	1.00	0.00	1.00	3.00	4.00	57.86	1.00	24.00	0.00	4.00	1.00	21.00	84.00	0.00
**756.00**	21.00	50.00	155.00	20.81	2.00	1.00	2.00	1.00	0.00	0.00	0.00	3.00	12.00	1.43	1.00	14.00	0.00	4.00	1.00	13.00	52.00	0.00
**757.00**	23.00	70.00	176.00	22.60	2.00	1.00	2.00	1.00	0.00	1.00	1.00	2.00	1.00	17.14	1.00	36.00	1.00	5.00	1.00	12.00	48.00	1.00
**758.00**	21.00	55.00	176.00	17.76	1.00	1.00	1.00	1.00	1.00	1.00	1.00	2.00	6.00	31.43	1.00	23.00	0.00	3.00	1.00	13.00	52.00	0.00
**759.00**	20.00	50.00	165.00	18.37	1.00	1.00	2.00	1.00	1.00	0.00	0.00	3.00	10.00	10.00	1.00	21.00	0.00	2.00	1.00	17.00	68.00	0.00
**760.00**	28.00	105.00	169.00	36.76	4.00	1.00	2.00	1.00	1.00	0.00	1.00	1.00	7.00	17.14	1.00	35.00	1.00	11.00	0.00	14.00	56.00	0.00
**761.00**	25.00	87.00	180.00	26.85	3.00	0.00	2.00	1.00	0.00	0.00	1.00	2.00	11.00	0.00	1.00	20.00	0.00	9.00	0.00	7.00	28.00	1.00
**762.00**	27.00	100.00	185.00	29.22	3.00	1.00	2.00	1.00	0.00	0.00	1.00	3.00	8.00	25.71	1.00	29.00	0.00	9.00	0.00	7.00	28.00	1.00
**763.00**	24.00	76.00	170.00	26.30	3.00	1.00	2.00	1.00	0.00	0.00	1.00	3.00	4.00	22.86	1.00	32.00	1.00	5.00	1.00	11.00	44.00	1.00
**764.00**	25.00	90.00	180.00	27.78	3.00	1.00	2.00	2.00	0.00	0.00	1.00	3.00	5.00	17.14	1.00	33.00	1.00	3.00	1.00	12.00	48.00	1.00
**765.00**	26.00	78.00	173.00	26.06	3.00	0.00	2.00	1.00	1.00	1.00	1.00	2.00	10.00	20.00	1.00	33.00	1.00	6.00	0.00	12.00	48.00	1.00
**766.00**	27.00	84.00	178.00	26.51	3.00	0.00	2.00	1.00	1.00	1.00	1.00	2.00	3.00	30.00	1.00	22.00	0.00	4.00	1.00	15.00	60.00	0.00
**767.00**	25.00	70.00	155.00	29.14	3.00	1.00	2.00	1.00	1.00	1.00	0.00	3.00	8.00	8.57	1.00	21.00	0.00	5.00	1.00	4.00	16.00	1.00
**768.00**	24.00	65.00	163.00	24.46	2.00	1.00	2.00	2.00	0.00	0.00	0.00	3.00	7.00	10.00	1.00	33.00	1.00	4.00	1.00	13.00	52.00	0.00
**769.00**	28.00	75.00	176.00	24.21	2.00	1.00	2.00	1.00	1.00	1.00	1.00	2.00	5.00	13.57	1.00	25.00	0.00	8.00	0.00	12.00	48.00	1.00
**770.00**	25.00	70.00	169.00	24.51	2.00	1.00	2.00	1.00	0.00	0.00	1.00	2.00	5.00	15.71	1.00	22.00	0.00	6.00	0.00	16.00	64.00	0.00
**771.00**	28.00	60.00	165.00	22.04	2.00	1.00	2.00	2.00	1.00	0.00	0.00	3.00	8.00	31.43	1.00	34.00	1.00	5.00	1.00	5.00	20.00	1.00
**772.00**	26.00	58.00	170.00	20.07	2.00	1.00	2.00	3.00	0.00	0.00	0.00	3.00	8.00	17.14	1.00	30.00	1.00	8.00	0.00	8.00	32.00	1.00
**773.00**	27.00	71.00	180.00	21.91	2.00	1.00	2.00	1.00	0.00	0.00	1.00	3.00	3.50	30.00	1.00	31.00	1.00	9.00	0.00	6.00	24.00	1.00
**774.00**	28.00	54.00	166.00	19.60	2.00	1.00	2.00	2.00	0.00	0.00	0.00	3.00	8.00	27.86	1.00	28.00	0.00	6.00	0.00	14.00	56.00	0.00
**775.00**	30.00	50.00	153.00	21.36	2.00	1.00	2.00	2.00	0.00	0.00	0.00	3.00	9.00	25.71	1.00	22.00	0.00	6.00	0.00	9.00	36.00	1.00
**776.00**	26.00	65.00	165.00	23.88	2.00	1.00	2.00	1.00	0.00	0.00	0.00	3.00	4.00	8.57	1.00	27.00	0.00	1.00	1.00	14.00	56.00	0.00
**777.00**	29.00	72.00	179.00	22.47	2.00	1.00	2.00	2.00	0.00	0.00	1.00	3.00	2.50	42.86	1.00	26.00	0.00	3.00	1.00	16.00	64.00	0.00
**778.00**	24.00	70.00	173.00	23.39	2.00	1.00	2.00	1.00	1.00	1.00	1.00	2.00	7.00	0.00	1.00	23.00	0.00	6.00	0.00	6.00	24.00	1.00
**779.00**	25.00	107.00	185.00	31.26	4.00	1.00	2.00	1.00	1.00	1.00	1.00	1.00	6.50	30.00	1.00	15.00	0.00	12.00	0.00	9.00	36.00	1.00
**780.00**	29.00	115.00	182.00	34.72	4.00	1.00	2.00	2.00	1.00	0.00	1.00	1.00	9.00	0.00	1.00	14.00	0.00	9.00	0.00	3.00	12.00	1.00
**781.00**	31.00	90.00	166.00	32.66	4.00	1.00	2.00	3.00	1.00	0.00	0.00	1.00	8.00	8.57	1.00	22.00	0.00	11.00	0.00	9.00	36.00	1.00
**782.00**	33.00	120.00	190.00	33.24	4.00	1.00	2.00	1.00	1.00	0.00	1.00	3.00	8.00	8.57	1.00	22.00	0.00	9.00	0.00	7.00	28.00	1.00
**783.00**	30.00	82.00	163.00	30.86	4.00	1.00	2.00	1.00	0.00	0.00	0.00	3.00	4.00	50.00	1.00	31.00	1.00	4.00	1.00	15.00	60.00	0.00
**784.00**	33.00	130.00	170.00	44.98	4.00	1.00	2.00	2.00	0.00	1.00	1.00	1.00	8.00	0.00	1.00	23.00	0.00	9.00	0.00	11.00	44.00	1.00
**785.00**	32.00	97.00	185.00	28.34	3.00	1.00	1.00	1.00	0.00	0.00	1.00	3.00	8.00	15.00	1.00	32.00	1.00	6.00	0.00	16.00	64.00	0.00
**786.00**	28.00	70.00	160.00	27.34	3.00	1.00	1.00	2.00	1.00	0.00	1.00	3.00	3.00	6.43	1.00	32.00	1.00	3.00	1.00	15.00	60.00	0.00
**787.00**	24.00	70.00	167.00	25.10	3.00	1.00	2.00	1.00	1.00	0.00	0.00	2.00	10.00	18.57	1.00	30.00	1.00	10.00	0.00	13.00	52.00	0.00
**788.00**	28.00	80.00	177.00	25.54	3.00	1.00	2.00	2.00	0.00	0.00	1.00	3.00	7.00	40.00	1.00	31.00	1.00	5.00	1.00	12.00	48.00	1.00
**789.00**	24.00	70.00	157.00	28.40	3.00	1.00	2.00	1.00	0.00	0.00	0.00	3.00	6.00	17.14	1.00	20.00	0.00	2.00	1.00	18.00	72.00	0.00
**790.00**	32.00	74.00	176.00	23.89	2.00	1.00	1.00	2.00	0.00	0.00	1.00	3.00	7.00	25.71	1.00	28.00	0.00	8.00	0.00	9.00	36.00	1.00
**791.00**	30.00	72.00	170.00	24.91	2.00	1.00	2.00	1.00	0.00	0.00	1.00	2.00	8.00	3.57	1.00	19.00	0.00	7.00	0.00	6.00	24.00	1.00
**792.00**	33.00	76.00	189.00	21.28	2.00	1.00	2.00	1.00	1.00	1.00	1.00	2.00	5.00	28.57	1.00	40.00	1.00	2.00	1.00	12.00	48.00	1.00

Subject	Age	Weight	Height	BMI	BMI_C	Residence	Education	Marital_Status	Smoking	Chronic_Dise	Gender	Health	Sitting_Time	PA_Min_Day	IPAQ_C	Dietary_Score	Dietary_C	PSQI_Total	Sleep_C	WHO5_Total	WHO5_Total100	WHO5_Class
**793.00**	28.00	73.00	176.00	23.57	2.00	1.00	2.00	2.00	1.00	0.00	1.00	2.00	5.00	0.00	1.00	25.00	0.00	6.00	0.00	14.00	56.00	0.00
**794.00**	32.00	63.00	160.00	24.61	2.00	1.00	2.00	2.00	0.00	0.00	0.00	2.00	5.00	0.00	1.00	27.00	0.00	3.00	1.00	15.00	60.00	0.00
**795.00**	28.00	76.00	176.00	24.54	2.00	1.00	2.00	1.00	0.00	0.00	1.00	3.00	5.50	14.29	1.00	18.00	0.00	8.00	0.00	5.00	20.00	1.00
**796.00**	30.00	70.00	174.00	23.12	2.00	1.00	2.00	1.00	1.00	0.00	1.00	3.00	6.00	12.86	1.00	30.00	1.00	4.00	1.00	19.00	76.00	0.00
**797.00**	25.00	70.00	175.00	22.86	2.00	1.00	2.00	2.00	0.00	0.00	1.00	3.00	7.00	42.86	1.00	24.00	0.00	4.00	1.00	19.00	76.00	0.00
**798.00**	30.00	65.00	170.00	22.49	2.00	1.00	2.00	3.00	1.00	0.00	1.00	3.00	5.50	22.86	1.00	23.00	0.00	5.00	1.00	12.00	48.00	1.00
**799.00**	29.00	58.00	170.00	20.07	2.00	1.00	2.00	2.00	0.00	1.00	1.00	2.00	6.00	0.00	1.00	28.00	0.00	4.00	1.00	17.00	68.00	0.00
**800.00**	28.00	79.00	179.00	24.66	2.00	0.00	3.00	1.00	1.00	0.00	1.00	3.00	4.00	42.86	1.00	34.00	1.00	6.00	0.00	13.00	52.00	0.00
**801.00**	30.00	78.00	178.00	24.62	2.00	1.00	3.00	2.00	0.00	1.00	0.00	2.00	6.00	2.14	1.00	23.00	0.00	13.00	0.00	4.00	16.00	1.00
**802.00**	26.00	98.00	175.00	32.00	4.00	1.00	2.00	2.00	0.00	0.00	1.00	3.00	10.00	0.00	1.00	23.00	0.00	5.00	1.00	15.00	60.00	0.00
**803.00**	27.00	95.00	180.00	29.32	3.00	0.00	2.00	2.00	0.00	0.00	1.00	3.00	5.00	0.00	1.00	28.00	0.00	8.00	0.00	15.00	60.00	0.00
**804.00**	25.00	80.00	177.00	25.54	3.00	1.00	2.00	1.00	0.00	1.00	1.00	2.00	11.00	32.14	1.00	29.00	0.00	4.00	1.00	12.00	48.00	1.00
**805.00**	31.00	84.00	182.00	25.36	3.00	1.00	2.00	2.00	0.00	1.00	1.00	2.00	8.00	8.57	1.00	25.00	0.00	3.00	1.00	16.00	64.00	0.00
**806.00**	31.00	61.00	167.00	21.87	2.00	1.00	2.00	2.00	0.00	0.00	0.00	3.00	8.00	45.71	1.00	33.00	1.00	1.00	1.00	13.00	52.00	0.00
**807.00**	27.00	58.00	159.00	22.94	2.00	1.00	2.00	2.00	0.00	0.00	0.00	3.00	6.50	21.43	1.00	42.00	1.00	1.00	1.00	17.00	68.00	0.00
**808.00**	25.00	64.00	163.00	24.09	2.00	1.00	3.00	1.00	0.00	1.00	0.00	3.00	11.00	51.43	1.00	28.00	0.00	1.00	1.00	13.00	52.00	0.00
**809.00**	24.00	81.00	163.00	30.49	4.00	1.00	2.00	2.00	1.00	1.00	1.00	2.00	11.00	0.00	1.00	30.00	1.00	8.00	0.00	12.00	48.00	1.00
**810.00**	25.00	110.00	170.00	38.06	4.00	0.00	3.00	1.00	1.00	0.00	1.00	3.00	1.00	25.71	1.00	24.00	0.00	4.00	1.00	16.00	64.00	0.00
**811.00**	27.00	88.00	187.00	25.17	3.00	1.00	1.00	1.00	1.00	1.00	1.00	2.00	6.00	38.57	1.00	24.00	0.00	4.00	1.00	11.00	44.00	1.00
**812.00**	24.00	76.00	170.00	26.30	3.00	1.00	1.00	1.00	0.00	0.00	1.00	3.00	4.00	11.43	1.00	31.00	1.00	3.00	1.00	18.00	72.00	0.00
**813.00**	26.00	70.00	165.00	25.71	3.00	1.00	2.00	2.00	0.00	0.00	0.00	2.00	3.00	38.57	1.00	29.00	0.00	8.00	0.00	12.00	48.00	1.00
**814.00**	30.00	67.00	170.00	23.18	2.00	1.00	1.00	2.00	0.00	0.00	1.00	3.00	7.00	17.14	1.00	30.00	1.00	4.00	1.00	13.00	52.00	0.00
**815.00**	29.00	81.00	183.00	24.19	2.00	1.00	1.00	1.00	0.00	0.00	1.00	3.00	6.00	25.71	1.00	24.00	0.00	4.00	1.00	19.00	76.00	0.00
**816.00**	32.00	65.00	168.00	23.03	2.00	1.00	1.00	1.00	0.00	0.00	1.00	3.00	10.00	17.14	1.00	30.00	1.00	3.00	1.00	17.00	68.00	0.00
**817.00**	27.00	85.00	190.00	23.55	2.00	0.00	1.00	1.00	1.00	1.00	1.00	1.00	3.00	24.29	1.00	25.00	0.00	10.00	0.00	9.00	36.00	1.00
**818.00**	27.00	70.00	180.00	21.60	2.00	1.00	1.00	1.00	1.00	1.00	1.00	2.00	6.00	0.00	1.00	32.00	1.00	4.00	1.00	15.00	60.00	0.00
**819.00**	27.00	77.00	188.00	21.79	2.00	1.00	1.00	2.00	1.00	1.00	1.00	2.00	5.50	22.86	1.00	32.00	1.00	4.00	1.00	14.00	56.00	0.00
**820.00**	27.00	54.00	167.00	19.36	2.00	0.00	1.00	2.00	0.00	0.00	1.00	3.00	7.00	60.00	1.00	32.00	1.00	2.00	1.00	17.00	68.00	0.00
**821.00**	30.00	60.00	156.00	24.65	2.00	0.00	2.00	2.00	1.00	0.00	0.00	3.00	1.50	0.00	1.00	39.00	1.00	6.00	0.00	17.00	68.00	0.00
**822.00**	24.00	68.00	173.00	22.72	2.00	1.00	2.00	1.00	1.00	1.00	0.00	3.00	6.00	64.29	1.00	28.00	0.00	4.00	1.00	16.00	64.00	0.00
**823.00**	31.00	70.00	180.00	21.60	2.00	1.00	3.00	1.00	0.00	0.00	1.00	2.00	1.00	2.14	1.00	23.00	0.00	5.00	1.00	9.00	36.00	1.00
**824.00**	30.00	77.00	173.00	25.73	3.00	1.00	2.00	2.00	0.00	0.00	1.00	3.00	6.00	62.14	1.00	25.00	0.00	6.00	0.00	15.00	60.00	0.00
**825.00**	29.00	70.00	157.00	28.40	3.00	1.00	2.00	1.00	1.00	1.00	0.00	3.00	4.50	0.00	1.00	16.00	0.00	5.00	1.00	10.00	40.00	1.00
**826.00**	31.00	56.00	154.00	23.61	2.00	1.00	2.00	2.00	0.00	0.00	0.00	1.00	3.50	0.00	1.00	33.00	1.00	6.00	0.00	8.00	32.00	1.00
**827.00**	25.00	68.00	165.00	24.98	2.00	1.00	2.00	2.00	0.00	0.00	0.00	2.00	7.00	8.57	1.00	26.00	0.00	4.00	1.00	12.00	48.00	1.00
**828.00**	32.00	68.00	168.00	24.09	2.00	1.00	2.00	1.00	0.00	0.00	0.00	3.00	8.00	17.14	1.00	30.00	1.00	6.00	0.00	23.00	92.00	0.00
**829.00**	28.00	55.00	159.00	21.76	2.00	1.00	2.00	2.00	0.00	1.00	0.00	2.00	10.00	0.00	1.00	32.00	1.00	6.00	0.00	12.00	48.00	1.00
**830.00**	28.00	58.00	163.00	21.83	2.00	1.00	3.00	1.00	0.00	0.00	0.00	3.00	6.50	40.29	1.00	30.00	1.00	4.00	1.00	21.00	84.00	0.00
**831.00**	27.00	49.00	163.00	18.44	1.00	1.00	2.00	2.00	0.00	0.00	0.00	3.00	7.00	0.00	1.00	27.00	0.00	6.00	0.00	12.00	48.00	1.00
**832.00**	26.00	45.00	157.00	18.26	1.00	1.00	2.00	1.00	0.00	0.00	0.00	3.00	6.50	21.43	1.00	35.00	1.00	1.00	1.00	16.00	64.00	0.00
**833.00**	29.00	82.00	161.00	31.63	4.00	1.00	1.00	2.00	0.00	0.00	0.00	3.00	5.00	0.00	1.00	24.00	0.00	6.00	0.00	17.00	68.00	0.00
**834.00**	32.00	99.00	161.00	38.19	4.00	1.00	2.00	2.00	1.00	1.00	0.00	1.00	5.00	24.29	1.00	32.00	1.00	9.00	0.00	13.00	52.00	0.00
**835.00**	30.00	63.00	158.00	25.24	3.00	1.00	1.00	2.00	0.00	0.00	0.00	3.00	5.00	51.43	1.00	39.00	1.00	3.00	1.00	17.00	68.00	0.00
**836.00**	29.00	70.00	158.00	28.04	3.00	1.00	2.00	1.00	0.00	0.00	0.00	2.00	6.00	0.00	1.00	29.00	0.00	8.00	0.00	7.00	28.00	1.00
**837.00**	30.00	75.00	169.00	26.26	3.00	1.00	2.00	2.00	0.00	1.00	0.00	2.00	7.00	0.00	1.00	27.00	0.00	2.00	1.00	12.00	48.00	1.00
**838.00**	33.00	80.00	170.00	27.68	3.00	1.00	2.00	2.00	0.00	0.00	0.00	2.00	3.00	0.00	1.00	28.00	0.00	88.34	0.00	17.00	68.00	0.00
**839.00**	30.00	70.00	163.00	26.35	3.00	1.00	2.00	2.00	0.00	0.00	0.00	2.00	4.00	0.00	1.00	25.00	0.00	6.00	0.00	12.00	48.00	1.00
**840.00**	32.00	72.00	169.00	25.21	3.00	1.00	2.00	2.00	0.00	0.00	0.00	3.00	1.50	5.71	1.00	32.00	1.00	9.00	0.00	19.00	76.00	0.00
**841.00**	32.00	74.00	171.00	25.31	3.00	1.00	2.00	2.00	1.00	0.00	0.00	3.00	7.00	4.29	1.00	25.00	0.00	4.00	1.00	14.00	56.00	0.00
**842.00**	29.00	67.00	160.00	26.17	3.00	1.00	2.00	2.00	1.00	0.00	0.00	3.00	5.00	17.14	1.00	34.00	1.00	2.00	1.00	17.00	68.00	0.00
**843.00**	29.00	80.00	165.00	29.38	3.00	1.00	2.00	2.00	0.00	1.00	0.00	3.00	6.00	2.14	1.00	30.00	1.00	5.00	1.00	11.00	44.00	1.00
**844.00**	26.00	65.00	164.00	24.17	2.00	1.00	1.00	2.00	0.00	0.00	0.00	2.00	5.00	8.57	1.00	21.00	0.00	7.00	0.00	9.00	36.00	1.00
**845.00**	25.00	62.00	159.00	24.52	2.00	0.00	1.00	1.00	1.00	0.00	0.00	2.00	2.50	0.00	1.00	27.00	0.00	12.00	0.00	9.00	36.00	1.00
**846.00**	30.00	63.00	159.00	24.92	2.00	1.00	1.00	2.00	0.00	0.00	0.00	2.00	7.00	2.14	1.00	26.00	0.00	5.00	1.00	15.00	60.00	0.00
**847.00**	30.00	60.00	165.00	22.04	2.00	1.00	1.00	2.00	1.00	0.00	0.00	3.00	5.00	0.00	1.00	23.00	0.00	6.00	0.00	11.00	44.00	1.00
**848.00**	28.00	54.00	158.00	21.63	2.00	1.00	1.00	2.00	0.00	1.00	0.00	1.00	11.00	0.00	1.00	30.00	1.00	8.00	0.00	13.00	52.00	0.00
**849.00**	31.00	59.40	159.00	23.50	2.00	1.00	1.00	2.00	0.00	1.00	0.00	3.00	9.00	25.71	1.00	31.00	1.00	7.00	0.00	10.00	40.00	1.00
**850.00**	31.00	57.00	155.00	23.73	2.00	1.00	2.00	2.00	0.00	0.00	0.00	3.00	4.00	4.29	1.00	37.00	1.00	4.00	1.00	15.00	60.00	0.00
**851.00**	30.00	59.00	170.00	20.42	2.00	1.00	2.00	1.00	1.00	1.00	0.00	3.00	7.00	30.00	1.00	29.00	0.00	9.00	0.00	12.00	48.00	1.00
**852.00**	31.00	64.00	165.00	23.51	2.00	1.00	2.00	2.00	0.00	1.00	0.00	3.00	2.00	8.57	1.00	38.00	1.00	9.00	0.00	13.00	52.00	0.00
**853.00**	26.00	65.00	165.00	23.88	2.00	1.00	2.00	2.00	0.00	0.00	0.00	2.00	2.50	4.29	1.00	28.00	0.00	7.00	0.00	13.00	52.00	0.00
**854.00**	31.00	68.00	165.00	24.98	2.00	1.00	2.00	2.00	1.00	0.00	0.00	3.00	5.00	0.00	1.00	25.00	0.00	7.00	0.00	9.00	36.00	1.00
**855.00**	33.00	68.00	172.00	22.99	2.00	0.00	2.00	2.00	1.00	0.00	0.00	3.00	4.00	21.43	1.00	23.00	0.00	89.21	0.00	14.00	56.00	0.00
**856.00**	30.00	57.00	162.00	21.72	2.00	1.00	2.00	2.00	1.00	1.00	0.00	3.00	1.50	0.00	1.00	26.00	0.00	2.00	1.00	19.00	76.00	0.00
**857.00**	32.00	97.00	169.00	33.96	4.00	1.00	2.00	2.00	0.00	0.00	0.00	3.00	8.00	10.71	1.00	28.00	0.00	4.00	1.00	11.00	44.00	1.00
**858.00**	28.00	82.00	172.00	27.72	3.00	1.00	2.00	2.00	1.00	0.00	1.00	1.00	8.00	8.57	1.00	21.00	0.00	3.00	1.00	8.00	32.00	1.00

Subject	Age	Weight	Height	BMI	BMI_C	Residence	Education	Marital_Status	Smoking	Chronic_Dise	Gender	Health	Sitting_Time	PA_Min_Day	IPAQ_C	Dietary_Score	Dietary_C	PSQI_Total	Sleep_C	WHO5_Total	WHO5_Total100	WHO5_Class
**859.00**	30.00	95.00	179.00	29.65	3.00	0.00	2.00	2.00	0.00	0.00	1.00	3.00	10.00	0.00	1.00	27.00	0.00	3.00	1.00	14.00	56.00	0.00
**860.00**	30.00	75.00	160.00	29.30	3.00	1.00	2.00	2.00	0.00	0.00	0.00	3.00	8.00	0.00	1.00	25.00	0.00	4.00	1.00	13.00	52.00	0.00
**861.00**	26.00	87.00	190.00	24.10	2.00	1.00	2.00	1.00	0.00	0.00	0.00	3.00	8.00	25.71	1.00	18.00	0.00	6.00	0.00	9.00	36.00	1.00
**862.00**	33.00	65.00	163.00	24.46	2.00	1.00	2.00	2.00	1.00	0.00	0.00	3.00	4.00	38.57	1.00	23.00	0.00	5.00	1.00	16.00	64.00	0.00
**863.00**	28.00	92.00	175.00	30.04	4.00	1.00	2.00	3.00	0.00	0.00	1.00	3.00	7.00	25.71	1.00	24.00	0.00	1.00	1.00	13.00	52.00	0.00
**864.00**	27.00	90.00	167.00	32.27	4.00	1.00	3.00	1.00	0.00	0.00	0.00	2.00	8.00	0.00	1.00	29.00	0.00	4.00	1.00	12.00	48.00	1.00
**865.00**	24.00	70.00	160.00	27.34	3.00	1.00	2.00	1.00	1.00	0.00	0.00	3.00	6.00	38.57	1.00	23.00	0.00	9.00	0.00	9.00	36.00	1.00
**866.00**	29.00	80.00	176.00	25.83	3.00	1.00	2.00	2.00	0.00	0.00	1.00	3.00	8.00	12.86	1.00	29.00	0.00	4.00	1.00	15.00	60.00	0.00
**867.00**	29.00	85.00	175.00	27.76	3.00	1.00	2.00	2.00	0.00	0.00	1.00	3.00	12.00	14.86	1.00	39.00	1.00	4.00	1.00	20.00	80.00	0.00
**868.00**	31.00	84.00	171.00	28.73	3.00	1.00	2.00	2.00	0.00	0.00	0.00	3.00	4.00	34.29	1.00	32.00	1.00	3.00	1.00	21.00	84.00	0.00
**869.00**	31.00	85.00	170.00	29.41	3.00	1.00	2.00	1.00	1.00	1.00	1.00	2.00	8.00	0.00	1.00	21.00	0.00	10.00	0.00	15.00	60.00	0.00
**870.00**	30.00	65.00	159.00	25.71	3.00	1.00	3.00	2.00	0.00	0.00	0.00	3.00	4.00	0.00	1.00	31.00	1.00	2.00	1.00	13.00	52.00	0.00
**871.00**	29.00	66.00	160.00	25.78	3.00	1.00	3.00	2.00	0.00	0.00	0.00	3.00	2.00	5.71	1.00	28.00	0.00	1.00	1.00	18.00	72.00	0.00
**872.00**	28.00	60.00	158.00	24.03	2.00	1.00	2.00	2.00	0.00	0.00	0.00	3.00	4.00	7.14	1.00	39.00	1.00	10.00	0.00	12.00	48.00	1.00
**873.00**	29.00	76.00	177.00	24.26	2.00	1.00	2.00	1.00	0.00	0.00	1.00	3.00	4.00	15.71	1.00	20.00	0.00	5.00	1.00	15.00	60.00	0.00
**874.00**	29.00	52.00	167.00	18.65	2.00	1.00	2.00	2.00	0.00	0.00	0.00	3.00	3.50	0.00	1.00	27.00	0.00	5.00	1.00	12.00	48.00	1.00
**875.00**	27.00	89.00	191.00	24.40	2.00	1.00	2.00	1.00	0.00	0.00	1.00	3.00	9.00	21.43	1.00	26.00	0.00	5.00	1.00	17.00	68.00	0.00
**876.00**	27.00	56.00	156.00	23.01	2.00	1.00	2.00	2.00	0.00	0.00	0.00	3.00	2.50	10.71	1.00	26.00	0.00	3.00	1.00	16.00	64.00	0.00
**877.00**	30.00	80.00	179.00	24.97	2.00	1.00	2.00	3.00	1.00	1.00	1.00	1.00	10.00	17.14	1.00	27.00	0.00	9.00	0.00	17.00	68.00	0.00
**878.00**	29.00	60.00	163.00	22.58	2.00	1.00	3.00	1.00	0.00	0.00	0.00	2.00	6.50	37.14	1.00	22.00	0.00	9.00	0.00	12.00	48.00	1.00
**879.00**	27.00	79.00	178.00	24.93	2.00	1.00	3.00	1.00	0.00	0.00	1.00	3.00	3.00	5.71	1.00	33.00	1.00	4.00	1.00	17.00	68.00	0.00
**880.00**	31.00	60.00	160.00	23.44	2.00	1.00	3.00	1.00	1.00	0.00	1.00	3.00	6.00	51.43	1.00	28.00	0.00	4.00	1.00	13.00	52.00	0.00
**881.00**	32.00	100.00	178.00	31.56	4.00	1.00	2.00	1.00	1.00	0.00	1.00	3.00	11.00	15.00	1.00	26.00	0.00	5.00	1.00	15.00	60.00	0.00
**882.00**	33.00	88.00	178.00	27.77	3.00	1.00	1.00	2.00	0.00	0.00	1.00	3.00	11.00	0.00	1.00	29.00	0.00	3.00	1.00	15.00	60.00	0.00
**883.00**	32.00	85.00	178.00	26.83	3.00	1.00	2.00	1.00	1.00	1.00	1.00	2.00	5.00	8.57	1.00	19.00	0.00	10.00	0.00	7.00	28.00	1.00
**884.00**	28.00	66.00	158.00	26.44	3.00	1.00	2.00	1.00	0.00	0.00	0.00	2.00	8.00	17.14	1.00	29.00	0.00	6.00	0.00	12.00	48.00	1.00
**885.00**	28.00	75.00	170.00	25.95	3.00	0.00	2.00	2.00	0.00	0.00	1.00	3.00	2.50	15.00	1.00	21.00	0.00	3.00	1.00	9.00	36.00	1.00
**886.00**	27.00	86.00	170.00	29.76	3.00	1.00	2.00	1.00	0.00	0.00	1.00	3.00	5.50	25.71	1.00	31.00	1.00	5.00	1.00	9.00	36.00	1.00
**887.00**	24.00	83.00	176.00	26.79	3.00	0.00	2.00	1.00	0.00	0.00	1.00	3.00	4.00	51.43	1.00	30.00	1.00	7.00	0.00	14.00	56.00	0.00
**888.00**	32.00	66.00	160.00	25.78	3.00	1.00	2.00	2.00	0.00	0.00	0.00	3.00	6.00	2.14	1.00	37.00	1.00	8.00	0.00	13.00	52.00	0.00
**889.00**	33.00	90.00	180.00	27.78	3.00	1.00	2.00	2.00	1.00	1.00	1.00	1.00	7.00	2.14	1.00	18.00	0.00	9.00	0.00	6.00	24.00	1.00
**890.00**	25.00	84.00	171.00	28.73	3.00	1.00	2.00	2.00	1.00	1.00	1.00	2.00	7.00	34.29	1.00	32.00	1.00	3.00	1.00	13.00	52.00	0.00
**891.00**	27.00	53.00	168.00	18.78	2.00	1.00	1.00	1.00	0.00	0.00	0.00	1.00	5.50	2.14	1.00	19.00	0.00	5.00	1.00	10.00	40.00	1.00
**892.00**	32.00	70.00	168.00	24.80	2.00	0.00	1.00	2.00	1.00	1.00	1.00	3.00	6.00	15.71	1.00	30.00	1.00	11.00	0.00	10.00	40.00	1.00
**893.00**	31.00	63.00	173.00	21.05	2.00	0.00	2.00	1.00	0.00	0.00	1.00	2.00	7.00	0.00	1.00	22.00	0.00	3.00	1.00	9.00	36.00	1.00
**894.00**	28.00	69.00	169.00	24.16	2.00	1.00	2.00	2.00	1.00	0.00	1.00	3.00	8.00	0.00	1.00	20.00	0.00	9.00	0.00	13.00	52.00	0.00
**895.00**	31.00	65.00	166.00	23.59	2.00	1.00	2.00	2.00	1.00	0.00	0.00	3.00	5.00	0.00	1.00	24.00	0.00	1.00	1.00	17.00	68.00	0.00
**896.00**	33.00	58.00	159.00	22.94	2.00	1.00	2.00	2.00	0.00	0.00	0.00	2.00	10.00	0.00	1.00	24.00	0.00	9.00	0.00	15.00	60.00	0.00
**897.00**	31.00	63.00	162.00	24.01	2.00	1.00	2.00	2.00	1.00	0.00	0.00	2.00	6.00	55.71	1.00	34.00	1.00	1.00	1.00	12.00	48.00	1.00
**898.00**	31.00	75.00	176.00	24.21	2.00	1.00	2.00	2.00	0.00	0.00	0.00	3.00	5.50	55.71	1.00	25.00	0.00	8.00	0.00	10.00	40.00	1.00
**899.00**	27.00	68.00	175.00	22.20	2.00	1.00	2.00	3.00	0.00	0.00	1.00	3.00	1.50	17.14	1.00	25.00	0.00	5.00	1.00	8.00	32.00	1.00
**900.00**	25.00	75.00	175.00	24.49	2.00	1.00	2.00	1.00	0.00	0.00	1.00	3.00	9.00	24.29	1.00	33.00	1.00	6.00	0.00	15.00	60.00	0.00
**901.00**	28.00	52.00	164.00	19.33	2.00	0.00	2.00	1.00	1.00	0.00	1.00	3.00	2.50	35.71	1.00	19.00	0.00	3.00	1.00	14.00	56.00	0.00
**902.00**	33.00	53.00	153.00	22.64	2.00	1.00	2.00	2.00	0.00	0.00	0.00	3.00	3.50	0.00	1.00	21.00	0.00	6.00	0.00	16.00	64.00	0.00
**903.00**	33.00	68.00	169.00	23.81	2.00	1.00	2.00	1.00	1.00	1.00	0.00	2.00	6.50	0.00	1.00	22.00	0.00	6.00	0.00	12.00	48.00	1.00
**904.00**	29.00	60.00	170.00	20.76	2.00	1.00	2.00	1.00	1.00	1.00	1.00	2.00	8.00	7.14	1.00	30.00	1.00	2.00	1.00	15.00	60.00	0.00
**905.00**	33.00	58.00	155.00	24.14	2.00	1.00	3.00	2.00	0.00	0.00	0.00	3.00	8.00	10.00	1.00	33.00	1.00	3.00	1.00	14.00	56.00	0.00
**906.00**	33.00	59.00	156.00	24.24	2.00	0.00	3.00	1.00	0.00	1.00	0.00	2.00	4.00	7.86	1.00	30.00	1.00	7.00	0.00	15.00	60.00	0.00
**907.00**	24.00	55.00	175.00	17.96	1.00	0.00	2.00	1.00	1.00	0.00	1.00	3.00	4.50	0.00	1.00	24.00	0.00	2.00	1.00	16.00	64.00	0.00
**908.00**	27.00	51.00	163.00	19.20	2.00	1.00	2.00	1.00	0.00	0.00	0.00	2.00	3.50	25.71	1.00	32.00	1.00	1.00	1.00	17.00	68.00	0.00
**909.00**	25.00	53.00	159.00	20.96	2.00	1.00	2.00	1.00	1.00	0.00	0.00	2.00	6.00	8.57	1.00	27.00	0.00	3.00	1.00	16.00	64.00	0.00
**910.00**	25.00	57.00	158.00	22.83	2.00	1.00	2.00	2.00	0.00	0.00	0.00	3.00	10.00	4.29	1.00	24.00	0.00	3.00	1.00	10.00	40.00	1.00
**911.00**	30.00	60.00	165.00	22.04	2.00	1.00	2.00	2.00	0.00	0.00	0.00	3.00	5.00	68.57	1.00	29.00	0.00	7.00	0.00	12.00	48.00	1.00
**912.00**	26.00	54.00	163.00	20.32	2.00	1.00	3.00	1.00	0.00	0.00	0.00	3.00	3.50	47.14	1.00	34.00	1.00	10.00	0.00	15.00	60.00	0.00
**913.00**	33.00	92.00	173.00	30.74	4.00	1.00	3.00	2.00	0.00	0.00	1.00	3.00	4.00	8.57	1.00	29.00	0.00	10.00	0.00	14.00	56.00	0.00
**914.00**	26.00	95.00	193.00	25.50	3.00	0.00	2.00	1.00	0.00	0.00	1.00	3.00	9.00	0.00	1.00	23.00	0.00	2.00	1.00	20.00	80.00	0.00
**915.00**	32.00	71.00	167.00	25.46	3.00	1.00	3.00	2.00	0.00	0.00	0.00	1.00	3.50	0.00	1.00	30.00	1.00	12.00	0.00	3.00	12.00	1.00
**916.00**	33.00	65.00	160.00	25.39	3.00	1.00	3.00	1.00	0.00	0.00	0.00	3.00	9.00	0.00	1.00	38.00	1.00	4.00	1.00	13.00	52.00	0.00
**917.00**	32.00	80.00	164.00	29.74	3.00	1.00	3.00	2.00	0.00	0.00	0.00	3.00	4.50	0.00	1.00	24.00	0.00	2.00	1.00	18.00	72.00	0.00
**918.00**	33.00	80.00	174.00	26.42	3.00	1.00	3.00	2.00	0.00	0.00	1.00	3.00	9.00	43.57	1.00	37.00	1.00	5.00	1.00	13.00	52.00	0.00
**919.00**	33.00	55.00	161.00	21.22	2.00	1.00	3.00	2.00	0.00	0.00	0.00	1.00	6.00	32.14	1.00	29.00	0.00	1.00	1.00	19.00	76.00	0.00
**920.00**	28.00	58.00	162.00	22.10	2.00	1.00	3.00	2.00	1.00	0.00	0.00	2.00	5.50	60.00	1.00	35.00	1.00	5.00	1.00	12.00	48.00	1.00
**921.00**	25.00	71.00	175.00	23.18	2.00	0.00	3.00	1.00	0.00	0.00	1.00	3.00	8.00	25.71	1.00	28.00	0.00	6.00	0.00	6.00	24.00	1.00
**922.00**	30.00	65.00	165.00	23.88	2.00	1.00	3.00	2.00	0.00	0.00	0.00	3.00	5.00	5.71	1.00	35.00	1.00	6.00	0.00	11.00	44.00	1.00
**923.00**	24.00	80.00	180.00	24.69	2.00	1.00	3.00	1.00	0.00	0.00	1.00	3.00	6.00	50.71	1.00	39.00	1.00	1.00	1.00	12.00	48.00	1.00
**924.00**	33.00	87.00	188.00	24.62	2.00	1.00	3.00	1.00	0.00	0.00	1.00	3.00	8.00	57.43	1.00	30.00	1.00	5.00	1.00	14.00	56.00	0.00

Subject	Age	Weight	Height	BMI	BMI_C	Residence	Education	Marital_Status	Smoking	Chronic_Dise	Gender	Health	Sitting_Time	PA_Min_Day	IPAQ_C	Dietary_Score	Dietary_C	PSQI_Total	Sleep_C	WHO5_Total	WHO5_Total100	WHO5_Class
**925.00**	31.00	55.00	178.00	17.36	1.00	0.00	3.00	2.00	0.00	0.00	0.00	1.00	5.00	4.29	1.00	24.00	0.00	3.00	1.00	9.00	36.00	1.00
**926.00**	27.00	85.00	163.00	31.99	4.00	1.00	1.00	2.00	0.00	0.00	0.00	3.00	6.00	0.00	1.00	23.00	0.00	8.00	0.00	8.00	32.00	1.00
**927.00**	31.00	87.00	169.00	30.46	4.00	1.00	1.00	2.00	1.00	1.00	0.00	1.00	9.00	12.86	1.00	29.00	0.00	9.00	0.00	18.00	72.00	0.00
**928.00**	25.00	65.00	160.00	25.39	3.00	0.00	1.00	1.00	0.00	0.00	0.00	1.00	6.00	17.14	1.00	26.00	0.00	3.00	1.00	10.00	40.00	1.00
**929.00**	30.00	81.00	176.00	26.15	3.00	1.00	1.00	1.00	0.00	0.00	1.00	3.00	10.00	22.86	1.00	13.00	0.00	6.00	0.00	6.00	24.00	1.00
**930.00**	24.00	65.00	160.00	25.39	3.00	1.00	1.00	2.00	0.00	0.00	1.00	3.00	8.00	12.86	1.00	27.00	0.00	7.00	0.00	14.00	56.00	0.00
**931.00**	28.00	72.00	165.00	26.45	3.00	1.00	1.00	1.00	0.00	0.00	1.00	3.00	8.00	35.00	1.00	27.00	0.00	6.00	0.00	14.00	56.00	0.00
**932.00**	26.00	95.00	184.00	28.06	3.00	1.00	1.00	1.00	0.00	0.00	1.00	3.00	7.00	45.00	1.00	39.00	1.00	1.00	1.00	17.00	68.00	0.00
**933.00**	26.00	68.00	164.00	25.28	3.00	0.00	1.00	2.00	0.00	0.00	0.00	3.00	7.00	21.43	1.00	25.00	0.00	5.00	1.00	17.00	68.00	0.00
**934.00**	24.00	80.00	176.00	25.83	3.00	0.00	1.00	2.00	1.00	1.00	1.00	1.00	8.00	8.57	1.00	28.00	0.00	10.00	0.00	13.00	52.00	0.00
**935.00**	29.00	70.00	160.00	27.34	3.00	0.00	2.00	2.00	0.00	0.00	0.00	2.00	5.00	19.29	1.00	25.00	0.00	8.00	0.00	7.00	28.00	1.00
**936.00**	24.00	78.00	176.00	25.18	3.00	0.00	2.00	1.00	1.00	0.00	1.00	3.00	6.00	21.43	1.00	23.00	0.00	9.00	0.00	9.00	36.00	1.00
**937.00**	25.00	66.00	160.00	25.78	3.00	1.00	2.00	1.00	0.00	0.00	0.00	3.00	3.50	22.86	1.00	29.00	0.00	3.00	1.00	17.00	68.00	0.00
**938.00**	28.00	90.00	177.00	28.73	3.00	1.00	3.00	1.00	0.00	0.00	1.00	3.00	6.00	60.00	1.00	23.00	0.00	2.00	1.00	21.00	84.00	0.00
**939.00**	29.00	58.00	159.00	22.94	2.00	1.00	1.00	2.00	0.00	0.00	0.00	1.00	10.00	0.00	1.00	20.00	0.00	6.00	0.00	21.00	84.00	0.00
**940.00**	25.00	52.00	164.00	19.33	2.00	1.00	1.00	1.00	0.00	0.00	0.00	2.00	6.00	4.29	1.00	27.00	0.00	4.00	1.00	14.00	56.00	0.00
**941.00**	31.00	68.00	174.00	22.46	2.00	1.00	1.00	2.00	0.00	0.00	0.00	3.00	2.00	25.71	1.00	31.00	1.00	6.00	0.00	5.00	20.00	1.00
**942.00**	30.00	55.00	160.00	21.48	2.00	1.00	1.00	2.00	0.00	0.00	0.00	3.00	7.00	10.00	1.00	28.00	0.00	8.00	0.00	11.00	44.00	1.00
**943.00**	32.00	53.00	169.00	18.56	2.00	1.00	1.00	2.00	0.00	0.00	0.00	3.00	3.50	15.00	1.00	30.00	1.00	6.00	0.00	9.00	36.00	1.00
**944.00**	27.00	63.00	165.00	23.14	2.00	1.00	1.00	2.00	0.00	0.00	0.00	3.00	1.00	21.43	1.00	38.00	1.00	9.00	0.00	15.00	60.00	0.00
**945.00**	24.00	78.00	181.00	23.81	2.00	1.00	1.00	1.00	0.00	0.00	1.00	3.00	5.00	22.86	1.00	31.00	1.00	9.00	0.00	14.00	56.00	0.00
**946.00**	28.00	75.00	174.00	24.77	2.00	1.00	1.00	1.00	0.00	0.00	1.00	3.00	7.00	18.57	1.00	29.00	0.00	1.00	1.00	21.00	84.00	0.00
**947.00**	24.00	54.00	164.00	20.08	2.00	0.00	1.00	1.00	0.00	0.00	1.00	3.00	3.00	20.00	1.00	27.00	0.00	7.00	0.00	13.00	52.00	0.00
**948.00**	26.00	58.00	170.00	20.07	2.00	1.00	1.00	2.00	0.00	0.00	0.00	3.00	3.00	25.71	1.00	35.00	1.00	3.00	1.00	15.00	60.00	0.00
**949.00**	25.00	70.00	170.00	24.22	2.00	0.00	1.00	1.00	1.00	1.00	1.00	1.00	4.50	35.00	1.00	31.00	1.00	6.00	0.00	13.00	52.00	0.00
**950.00**	25.00	61.00	160.00	23.83	2.00	1.00	1.00	1.00	0.00	1.00	0.00	3.00	6.50	0.00	1.00	29.00	0.00	8.00	0.00	12.00	48.00	1.00
**951.00**	24.00	64.00	164.00	23.80	2.00	1.00	1.00	1.00	0.00	1.00	0.00	3.00	10.00	1.43	1.00	36.00	1.00	5.00	1.00	14.00	56.00	0.00
**952.00**	24.00	50.00	160.00	19.53	2.00	1.00	2.00	1.00	1.00	0.00	0.00	2.00	9.00	35.71	1.00	25.00	0.00	5.00	1.00	4.00	16.00	1.00
**953.00**	24.00	58.00	172.00	19.61	2.00	1.00	2.00	1.00	0.00	0.00	1.00	1.00	11.00	54.29	1.00	20.00	0.00	4.00	1.00	6.00	24.00	1.00
**954.00**	25.00	60.00	168.00	21.26	2.00	0.00	2.00	1.00	0.00	0.00	1.00	1.00	6.50	51.43	1.00	22.00	0.00	7.00	0.00	9.00	36.00	1.00
**955.00**	27.00	70.00	172.00	23.66	2.00	1.00	2.00	1.00	0.00	0.00	1.00	3.00	6.00	21.43	1.00	32.00	1.00	6.00	0.00	5.00	20.00	1.00
**956.00**	24.00	60.00	156.00	24.65	2.00	1.00	2.00	1.00	1.00	0.00	0.00	3.00	8.00	38.57	1.00	26.00	0.00	4.00	1.00	12.00	48.00	1.00
**957.00**	25.00	78.00	178.00	24.62	2.00	1.00	2.00	1.00	1.00	0.00	1.00	3.00	10.00	22.86	1.00	26.00	0.00	5.00	1.00	17.00	68.00	0.00
**958.00**	26.00	52.00	157.00	21.10	2.00	1.00	2.00	1.00	0.00	0.00	0.00	3.00	6.00	25.71	1.00	26.00	0.00	4.00	1.00	13.00	52.00	0.00
**959.00**	26.00	52.00	167.00	18.65	2.00	1.00	3.00	1.00	0.00	0.00	0.00	1.00	10.00	6.43	1.00	22.00	0.00	6.00	0.00	7.00	28.00	1.00
**960.00**	29.00	60.00	160.00	23.44	2.00	0.00	3.00	1.00	0.00	0.00	0.00	2.00	10.00	0.00	1.00	28.00	0.00	5.00	1.00	13.00	52.00	0.00
**961.00**	28.00	67.00	166.00	24.31	2.00	1.00	3.00	1.00	0.00	0.00	0.00	2.00	8.00	37.86	1.00	26.00	0.00	4.00	1.00	14.00	56.00	0.00
**962.00**	30.00	56.00	160.00	21.88	2.00	1.00	3.00	1.00	0.00	0.00	0.00	3.00	5.00	25.71	1.00	36.00	1.00	6.00	0.00	15.00	60.00	0.00
**963.00**	25.00	40.00	150.00	17.78	1.00	1.00	1.00	1.00	0.00	0.00	0.00	3.00	11.00	36.43	1.00	22.00	0.00	3.00	1.00	15.00	60.00	0.00
**964.00**	24.00	53.00	170.00	18.34	1.00	1.00	1.00	1.00	1.00	1.00	0.00	3.00	8.00	42.86	1.00	24.00	0.00	12.00	0.00	10.00	40.00	1.00
**965.00**	25.00	50.00	168.00	17.72	1.00	1.00	1.00	1.00	0.00	1.00	0.00	3.00	6.00	0.00	1.00	25.00	0.00	7.00	0.00	15.00	60.00	0.00
**966.00**	24.00	45.00	163.00	16.94	1.00	1.00	1.00	1.00	0.00	1.00	0.00	3.00	11.00	18.57	1.00	38.00	1.00	3.00	1.00	17.00	68.00	0.00
**967.00**	30.00	46.00	159.00	18.20	1.00	1.00	3.00	1.00	0.00	0.00	0.00	3.00	6.50	35.00	1.00	31.00	1.00	14.00	0.00	12.00	48.00	1.00
**968.00**	28.00	90.00	160.00	35.16	4.00	1.00	2.00	1.00	0.00	0.00	0.00	1.00	6.50	12.86	1.00	20.00	0.00	6.00	0.00	11.00	44.00	1.00
**969.00**	31.00	115.00	190.00	31.86	4.00	1.00	2.00	2.00	0.00	0.00	1.00	2.00	8.00	51.43	1.00	28.00	0.00	14.00	0.00	6.00	24.00	1.00
**970.00**	32.00	92.00	173.00	30.74	4.00	0.00	2.00	2.00	0.00	0.00	1.00	2.00	9.00	0.00	1.00	16.00	0.00	6.00	0.00	12.00	48.00	1.00
**971.00**	29.00	105.00	187.00	30.03	4.00	1.00	2.00	1.00	0.00	0.00	1.00	2.00	6.00	5.71	1.00	24.00	0.00	2.00	1.00	13.00	52.00	0.00
**972.00**	32.00	89.00	165.00	32.69	4.00	1.00	2.00	2.00	0.00	0.00	1.00	2.00	11.00	0.00	1.00	29.00	0.00	3.00	1.00	13.00	52.00	0.00
**973.00**	29.00	105.00	187.00	30.03	4.00	1.00	2.00	2.00	1.00	0.00	1.00	3.00	11.00	0.00	1.00	23.00	0.00	4.00	1.00	16.00	64.00	0.00
**974.00**	32.00	97.00	178.00	30.61	4.00	1.00	2.00	2.00	0.00	0.00	1.00	3.00	9.00	0.00	1.00	24.00	0.00	2.00	1.00	19.00	76.00	0.00
**975.00**	29.00	92.00	174.00	30.39	4.00	1.00	2.00	2.00	0.00	1.00	0.00	2.00	11.00	7.14	1.00	22.00	0.00	7.00	0.00	11.00	44.00	1.00
**976.00**	29.00	86.00	169.00	30.11	4.00	1.00	3.00	1.00	0.00	0.00	1.00	3.00	11.00	17.14	1.00	27.00	0.00	86.21	0.00	19.00	76.00	0.00
**977.00**	26.00	78.00	172.00	26.37	3.00	1.00	2.00	1.00	0.00	0.00	0.00	1.00	4.00	17.14	1.00	31.00	1.00	3.00	1.00	12.00	48.00	1.00
**978.00**	25.00	79.00	166.00	28.67	3.00	0.00	2.00	1.00	0.00	0.00	1.00	2.00	10.00	26.43	1.00	21.00	0.00	10.00	0.00	15.00	60.00	0.00
**979.00**	28.00	82.00	168.00	29.05	3.00	1.00	2.00	1.00	0.00	0.00	0.00	2.00	1.50	18.57	1.00	35.00	1.00	2.00	1.00	14.00	56.00	0.00
**980.00**	31.00	95.00	179.00	29.65	3.00	1.00	2.00	2.00	1.00	0.00	1.00	3.00	4.00	32.14	1.00	27.00	0.00	7.00	0.00	5.00	20.00	1.00
**981.00**	29.00	89.00	181.00	27.17	3.00	1.00	2.00	2.00	1.00	0.00	1.00	3.00	11.00	8.57	1.00	30.00	1.00	7.00	0.00	8.00	32.00	1.00
**982.00**	32.00	76.00	173.00	25.39	3.00	1.00	2.00	2.00	0.00	0.00	1.00	3.00	2.00	0.00	1.00	26.00	0.00	8.00	0.00	14.00	56.00	0.00
**983.00**	31.00	72.00	162.00	27.43	3.00	1.00	2.00	1.00	0.00	0.00	0.00	3.00	5.00	0.00	1.00	23.00	0.00	6.00	0.00	9.00	36.00	1.00
**984.00**	33.00	64.00	155.00	26.64	3.00	0.00	2.00	1.00	0.00	0.00	0.00	3.00	11.00	0.00	1.00	32.00	1.00	7.00	0.00	14.00	56.00	0.00
**985.00**	25.00	70.00	164.00	26.03	3.00	1.00	2.00	2.00	0.00	0.00	0.00	3.00	6.00	35.00	1.00	28.00	0.00	5.00	1.00	10.00	40.00	1.00
**986.00**	26.00	72.00	156.00	29.59	3.00	1.00	2.00	2.00	0.00	0.00	0.00	3.00	4.00	25.71	1.00	28.00	0.00	5.00	1.00	10.00	40.00	1.00
**987.00**	30.00	69.00	165.00	25.34	3.00	1.00	2.00	2.00	0.00	0.00	0.00	3.00	9.00	14.29	1.00	29.00	0.00	1.00	1.00	16.00	64.00	0.00
**988.00**	31.00	78.00	170.00	26.99	3.00	0.00	2.00	1.00	0.00	0.00	1.00	3.00	4.00	62.14	1.00	34.00	1.00	5.00	1.00	17.00	68.00	0.00
**989.00**	24.00	67.00	162.00	25.53	3.00	1.00	2.00	1.00	0.00	0.00	0.00	3.00	5.50	0.00	1.00	26.00	0.00	3.00	1.00	16.00	64.00	0.00
**990.00**	33.00	83.00	178.00	26.20	3.00	1.00	2.00	2.00	1.00	1.00	1.00	2.00	7.00	12.86	1.00	28.00	0.00	5.00	1.00	17.00	68.00	0.00

Subject	Age	Weight	Height	BMI	BMI_C	Residence	Education	Marital_Status	Smoking	Chronic_Dise	Gender	Health	Sitting_Time	PA_Min_Day	IPAQ_C	Dietary_Score	Dietary_C	PSQI_Total	Sleep_C	WHO5_Total	WHO5_Total100	WHO5_Class
991.00	30.00	74.00	163.00	27.85	3.00	1.00	3.00	2.00	0.00	0.00	0.00	2.00	8.00	25.71	1.00	34.00	1.00	9.00	0.00	12.00	48.00	1.00
992.00	31.00	72.00	168.00	25.51	3.00	1.00	3.00	2.00	0.00	0.00	1.00	3.00	4.00	17.14	1.00	25.00	0.00	6.00	0.00	17.00	68.00	0.00
993.00	25.00	67.00	185.00	19.58	2.00	1.00	1.00	1.00	0.00	0.00	1.00	3.00	10.00	55.71	1.00	29.00	0.00	4.00	1.00	13.00	52.00	0.00
994.00	32.00	60.00	169.00	21.01	2.00	0.00	2.00	1.00	1.00	0.00	1.00	3.00	4.00	60.00	1.00	20.00	0.00	3.00	1.00	15.00	60.00	0.00
995.00	33.00	58.00	159.00	22.94	2.00	1.00	2.00	2.00	0.00	0.00	0.00	1.00	7.00	66.43	1.00	32.00	1.00	5.00	1.00	9.00	36.00	1.00
996.00	26.00	64.00	168.00	22.68	2.00	1.00	2.00	1.00	0.00	0.00	0.00	2.00	7.00	8.57	1.00	24.00	0.00	6.00	0.00	17.00	68.00	0.00
997.00	28.00	50.00	158.00	20.03	2.00	1.00	2.00	2.00	0.00	0.00	0.00	2.00	5.00	2.86	1.00	32.00	1.00	2.00	1.00	14.00	56.00	0.00
998.00	29.00	78.00	178.00	24.62	2.00	1.00	2.00	2.00	1.00	0.00	1.00	2.00	4.00	17.14	1.00	23.00	0.00	4.00	1.00	13.00	52.00	0.00
999.00	30.00	64.00	179.00	19.97	2.00	0.00	2.00	1.00	0.00	0.00	1.00	3.00	8.00	0.00	1.00	27.00	0.00	8.00	0.00	3.00	12.00	1.00
1000.00	28.00	80.00	179.00	24.97	2.00	1.00	2.00	1.00	0.00	0.00	1.00	3.00	10.00	8.57	1.00	21.00	0.00	9.00	0.00	8.00	32.00	1.00
1001.00	30.00	50.00	160.00	19.53	2.00	1.00	2.00	1.00	0.00	0.00	0.00	3.00	8.00	12.86	1.00	34.00	1.00	8.00	0.00	15.00	60.00	0.00
1002.00	25.00	52.00	160.00	20.31	2.00	1.00	2.00	2.00	1.00	0.00	0.00	3.00	2.00	8.57	1.00	23.00	0.00	6.00	0.00	15.00	60.00	0.00
1003.00	24.00	65.00	174.00	21.47	2.00	1.00	2.00	1.00	0.00	0.00	0.00	3.00	2.50	12.86	1.00	27.00	0.00	3.00	1.00	10.00	40.00	1.00
1004.00	28.00	55.00	162.00	20.96	2.00	0.00	2.00	2.00	0.00	0.00	0.00	3.00	7.00	27.14	1.00	33.00	1.00	5.00	1.00	14.00	56.00	0.00
1005.00	24.00	55.00	167.00	19.72	2.00	0.00	2.00	2.00	0.00	0.00	0.00	3.00	2.50	8.57	1.00	30.00	1.00	1.00	1.00	13.00	52.00	0.00
1006.00	25.00	66.00	183.00	19.71	2.00	1.00	2.00	1.00	1.00	0.00	1.00	3.00	6.00	4.29	1.00	34.00	1.00	4.00	1.00	15.00	60.00	0.00
1007.00	29.00	65.00	166.00	23.59	2.00	1.00	2.00	2.00	0.00	0.00	1.00	3.00	9.00	37.14	1.00	28.00	0.00	2.00	1.00	18.00	72.00	0.00
1008.00	30.00	75.00	176.00	24.21	2.00	1.00	2.00	2.00	0.00	0.00	1.00	3.00	4.00	4.29	1.00	27.00	0.00	2.00	1.00	18.00	72.00	0.00
1009.00	25.00	59.00	160.00	23.05	2.00	0.00	2.00	1.00	0.00	0.00	0.00	3.00	8.00	25.71	1.00	30.00	1.00	7.00	0.00	16.00	64.00	0.00
1010.00	32.00	65.00	178.00	20.52	2.00	1.00	2.00	2.00	1.00	0.00	1.00	3.00	10.00	8.57	1.00	25.00	0.00	11.00	0.00	13.00	52.00	0.00
1011.00	30.00	59.00	160.00	23.05	2.00	0.00	2.00	1.00	0.00	0.00	0.00	3.00	6.00	64.29	1.00	36.00	1.00	4.00	1.00	16.00	64.00	0.00
1012.00	31.00	64.00	165.00	23.51	2.00	0.00	2.00	2.00	0.00	0.00	1.00	3.00	1.50	21.43	1.00	35.00	1.00	0.00	1.00	22.00	88.00	0.00
1013.00	24.00	75.00	177.00	23.94	2.00	0.00	2.00	1.00	0.00	0.00	0.00	3.00	5.50	10.71	1.00	22.00	0.00	4.00	1.00	14.00	56.00	0.00
1014.00	25.00	66.00	183.00	19.71	2.00	1.00	2.00	1.00	0.00	1.00	1.00	2.00	9.00	29.29	1.00	29.00	0.00	8.00	0.00	2.00	8.00	1.00
1015.00	32.00	78.00	178.00	24.62	2.00	1.00	2.00	2.00	1.00	1.00	1.00	2.00	3.00	27.86	1.00	23.00	0.00	1.00	1.00	17.00	68.00	0.00
1016.00	33.00	60.00	158.00	24.03	2.00	1.00	3.00	2.00	0.00	0.00	0.00	2.00	5.00	0.00	1.00	24.00	0.00	5.00	1.00	11.00	44.00	1.00
1017.00	33.00	54.00	151.00	23.68	2.00	1.00	3.00	1.00	0.00	0.00	0.00	3.00	3.50	17.14	1.00	23.00	0.00	2.00	1.00	13.00	52.00	0.00
1018.00	28.00	53.00	156.00	21.78	2.00	0.00	3.00	2.00	0.00	0.00	0.00	3.00	9.00	8.57	1.00	27.00	0.00	4.00	1.00	15.00	60.00	0.00
1019.00	28.00	56.00	168.00	19.84	2.00	1.00	3.00	1.00	0.00	0.00	1.00	3.00	4.50	1.43	1.00	23.00	0.00	3.00	1.00	16.00	64.00	0.00
1020.00	27.00	62.00	173.00	20.72	2.00	1.00	3.00	1.00	0.00	0.00	1.00	3.00	8.00	22.86	1.00	21.00	0.00	3.00	1.00	15.00	60.00	0.00
1021.00	30.00	70.00	172.00	23.66	2.00	1.00	3.00	2.00	0.00	0.00	1.00	3.00	7.00	8.57	1.00	23.00	0.00	5.00	1.00	19.00	76.00	0.00
1022.00	33.00	70.00	169.00	24.51	2.00	1.00	3.00	2.00	0.00	1.00	1.00	2.00	9.00	19.29	1.00	20.00	0.00	4.00	1.00	16.00	64.00	0.00
1023.00	27.00	47.00	165.00	17.26	1.00	1.00	3.00	1.00	0.00	0.00	0.00	1.00	11.00	2.14	1.00	18.00	0.00	11.00	0.00	6.00	24.00	1.00
1024.00	30.00	80.00	160.00	31.25	4.00	1.00	2.00	2.00	0.00	0.00	0.00	2.00	4.00	0.00	1.00	20.00	0.00	5.00	1.00	12.00	48.00	1.00
1025.00	25.00	85.00	173.00	28.40	3.00	0.00	1.00	1.00	0.00	0.00	1.00	3.00	8.00	0.00	1.00	20.00	0.00	7.00	0.00	10.00	40.00	1.00
1026.00	31.00	69.00	163.00	25.97	3.00	1.00	2.00	2.00	0.00	0.00	0.00	2.00	9.00	2.86	1.00	23.00	0.00	5.00	1.00	11.00	44.00	1.00
1027.00	28.00	75.00	160.00	29.30	3.00	1.00	2.00	1.00	0.00	0.00	0.00	3.00	6.00	2.14	1.00	36.00	1.00	2.00	1.00	17.00	68.00	0.00
1028.00	26.00	62.00	169.00	21.71	2.00	1.00	1.00	1.00	0.00	0.00	0.00	2.00	6.00	0.00	1.00	22.00	0.00	7.00	0.00	8.00	32.00	1.00
1029.00	28.00	72.00	175.00	23.51	2.00	1.00	1.00	1.00	0.00	0.00	1.00	3.00	3.00	6.43	1.00	26.00	0.00	7.00	0.00	16.00	64.00	0.00
1030.00	29.00	57.00	162.00	21.72	2.00	0.00	2.00	1.00	0.00	0.00	0.00	3.00	8.00	1.43	1.00	17.00	0.00	6.00	0.00	16.00	64.00	0.00
1031.00	26.00	63.00	165.00	23.14	2.00	1.00	2.00	1.00	0.00	0.00	1.00	3.00	5.00	45.00	1.00	34.00	1.00	1.00	1.00	12.00	48.00	1.00
1032.00	29.00	60.00	159.00	23.73	2.00	1.00	2.00	2.00	1.00	1.00	0.00	3.00	2.50	30.00	1.00	26.00	0.00	9.00	0.00	14.00	56.00	0.00
1033.00	24.00	66.00	177.00	21.07	2.00	1.00	2.00	1.00	1.00	1.00	1.00	3.00	5.00	20.00	1.00	26.00	0.00	4.00	1.00	17.00	68.00	0.00
1034.00	25.00	58.00	174.00	19.16	2.00	1.00	3.00	1.00	0.00	0.00	0.00	1.00	11.00	0.00	1.00	20.00	0.00	3.00	1.00	14.00	56.00	0.00
1035.00	25.00	55.00	175.00	17.96	1.00	1.00	3.00	1.00	0.00	0.00	1.00	1.00	6.00	14.29	1.00	24.00	0.00	3.00	1.00	9.00	36.00	1.00
1036.00	39.00	85.00	180.00	26.23	3.00	0.00	1.00	2.00	0.00	0.00	1.00	3.00	7.00	31.86	1.00	31.00	1.00	4.00	1.00	10.00	40.00	1.00
1037.00	36.00	100.00	182.00	30.19	4.00	1.00	1.00	2.00	0.00	0.00	1.00	2.00	4.50	32.14	1.00	24.00	0.00	4.00	1.00	12.00	48.00	1.00
1038.00	39.00	125.00	190.00	34.63	4.00	1.00	2.00	1.00	1.00	0.00	1.00	3.00	3.50	36.43	1.00	32.00	1.00	1.00	1.00	17.00	68.00	0.00
1039.00	39.00	123.00	190.00	34.07	4.00	1.00	2.00	1.00	1.00	0.00	1.00	3.00	3.50	38.57	1.00	31.00	1.00	3.00	1.00	17.00	68.00	0.00
1040.00	43.00	105.00	187.00	30.03	4.00	1.00	3.00	2.00	0.00	0.00	1.00	2.00	5.00	5.71	1.00	26.00	0.00	2.00	1.00	15.00	60.00	0.00
1041.00	36.00	85.00	183.00	25.38	3.00	1.00	2.00	2.00	0.00	0.00	1.00	3.00	6.00	12.86	1.00	25.00	0.00	1.00	1.00	17.00	68.00	0.00
1042.00	40.00	84.00	173.00	28.07	3.00	1.00	2.00	2.00	1.00	1.00	1.00	1.00	2.00	51.43	1.00	35.00	1.00	0.00	1.00	14.00	56.00	0.00
1043.00	34.00	71.00	162.00	27.05	3.00	1.00	2.00	2.00	0.00	0.00	0.00	3.00	7.00	12.86	1.00	34.00	1.00	3.00	1.00	16.00	64.00	0.00
1044.00	40.00	80.00	178.00	25.25	3.00	1.00	2.00	2.00	1.00	0.00	1.00	3.00	4.00	34.29	1.00	31.00	1.00	4.00	1.00	12.00	48.00	1.00
1045.00	43.00	78.00	172.00	26.37	3.00	1.00	2.00	2.00	0.00	0.00	1.00	3.00	1.50	8.57	1.00	34.00	1.00	3.00	1.00	17.00	68.00	0.00
1046.00	40.00	100.00	191.00	27.41	3.00	1.00	2.00	2.00	1.00	1.00	1.00	1.00	5.50	50.71	1.00	33.00	1.00	9.00	0.00	6.00	24.00	1.00
1047.00	43.00	110.00	192.00	29.84	3.00	1.00	3.00	2.00	1.00	0.00	1.00	2.00	9.00	6.43	1.00	20.00	0.00	7.00	0.00	7.00	28.00	1.00
1048.00	42.00	55.00	168.00	19.49	2.00	1.00	1.00	2.00	0.00	0.00	0.00	3.00	5.00	12.86	1.00	37.00	1.00	0.00	1.00	16.00	64.00	0.00
1049.00	38.00	65.00	163.00	24.46	2.00	1.00	2.00	2.00	1.00	1.00	1.00	2.00	6.00	27.14	1.00	31.00	1.00	1.00	1.00	21.00	84.00	0.00
1050.00	40.00	75.00	175.00	24.49	2.00	1.00	2.00	2.00	0.00	0.00	0.00	3.00	11.00	60.00	1.00	34.00	1.00	11.00	0.00	12.00	48.00	1.00
1051.00	43.00	82.00	185.00	23.96	2.00	0.00	2.00	2.00	0.00	0.00	1.00	3.00	3.00	25.71	1.00	27.00	0.00	5.00	1.00	12.00	48.00	1.00
1052.00	35.00	76.00	175.00	24.82	2.00	1.00	2.00	3.00	0.00	0.00	1.00	3.00	4.00	22.86	1.00	39.00	1.00	5.00	1.00	12.00	48.00	1.00
1053.00	35.00	65.00	170.00	22.49	2.00	1.00	2.00	2.00	0.00	0.00	0.00	3.00	4.00	35.71	1.00	33.00	1.00	1.00	1.00	14.00	56.00	0.00
1054.00	39.00	72.00	170.00	24.91	2.00	1.00	3.00	2.00	0.00	0.00	1.00	3.00	6.00	12.86	1.00	25.00	0.00	0.00	1.00	13.00	52.00	0.00
1055.00	38.00	64.00	170.00	22.15	2.00	1.00	3.00	1.00	0.00	0.00	0.00	3.00	4.50	55.71	1.00	33.00	1.00	2.00	1.00	14.00	56.00	0.00
1056.00	38.00	118.00	191.00	32.35	4.00	1.00	1.00	2.00	1.00	0.00	1.00	3.00	7.00	60.00	1.00	26.00	0.00	6.00	0.00	14.00	56.00	0.00

Subject	Age	Weight	Height	BMI	BMI_C	Residence	Education	Marital_Status	Smoking	Chronic_Dise	Gender	Health	Sitting_Time	PA_Min_Day	IPAQ_C	Dietary_Score	Dietary_C	PSQI_Total	Sleep_C	WHO5_Total	WHO5_Total100	WHO5_Class
1057.00	37.00	104.00	183.00	31.05	4.00	0.00	2.00	2.00	0.00	0.00	1.00	3.00	8.00	32.14	1.00	28.00	0.00	6.00	0.00	13.00	52.00	0.00
1058.00	34.00	105.00	170.00	36.33	4.00	1.00	2.00	2.00	0.00	0.00	1.00	2.00	9.00	0.00	1.00	24.00	0.00	3.00	1.00	13.00	52.00	0.00
1059.00	40.00	120.00	185.00	35.06	4.00	1.00	2.00	2.00	1.00	0.00	1.00	3.00	9.00	0.00	1.00	24.00	0.00	8.00	0.00	11.00	44.00	1.00
1060.00	35.00	100.00	177.00	31.92	4.00	1.00	2.00	2.00	1.00	0.00	1.00	3.00	2.50	57.86	1.00	27.00	0.00	10.00	0.00	11.00	44.00	1.00
1061.00	38.00	120.00	167.00	43.03	4.00	1.00	2.00	2.00	0.00	0.00	1.00	3.00	4.00	8.57	1.00	26.00	0.00	9.00	0.00	11.00	44.00	1.00
1062.00	36.00	95.00	175.00	31.02	4.00	1.00	2.00	2.00	0.00	0.00	1.00	3.00	4.00	17.14	1.00	27.00	0.00	7.00	0.00	17.00	68.00	0.00
1063.00	42.00	105.00	173.00	35.08	4.00	1.00	2.00	2.00	0.00	1.00	1.00	2.00	9.00	7.14	1.00	33.00	1.00	6.00	0.00	12.00	48.00	1.00
1064.00	36.00	90.00	169.00	31.51	4.00	1.00	2.00	2.00	1.00	1.00	1.00	2.00	10.00	4.29	1.00	40.00	1.00	3.00	1.00	13.00	52.00	0.00
1065.00	38.00	100.00	178.00	31.56	4.00	1.00	2.00	3.00	1.00	1.00	1.00	2.00	7.00	8.57	1.00	32.00	1.00	3.00	1.00	17.00	68.00	0.00
1066.00	36.00	90.00	171.00	30.78	4.00	1.00	2.00	2.00	0.00	1.00	1.00	3.00	4.00	41.71	1.00	34.00	1.00	6.00	0.00	19.00	76.00	0.00
1067.00	44.00	88.00	170.00	30.45	4.00	1.00	3.00	2.00	0.00	0.00	1.00	2.00	5.00	45.00	1.00	37.00	1.00	6.00	0.00	20.00	80.00	0.00
1068.00	42.00	120.00	183.00	35.83	4.00	1.00	3.00	2.00	1.00	0.00	1.00	2.00	11.00	0.00	1.00	31.00	1.00	4.00	1.00	12.00	48.00	1.00
1069.00	35.00	70.00	162.00	26.67	3.00	1.00	2.00	1.00	0.00	1.00	0.00	2.00	4.50	0.00	1.00	29.00	0.00	10.00	0.00	4.00	16.00	1.00
1070.00	35.00	90.00	178.00	28.41	3.00	1.00	2.00	2.00	1.00	0.00	1.00	1.00	5.50	0.00	1.00	22.00	0.00	8.00	0.00	3.00	12.00	1.00
1071.00	40.00	84.00	175.00	27.43	3.00	1.00	2.00	2.00	1.00	0.00	1.00	2.00	4.00	2.14	1.00	22.00	0.00	9.00	0.00	12.00	48.00	1.00
1072.00	40.00	78.00	176.00	25.18	3.00	1.00	2.00	2.00	1.00	0.00	1.00	3.00	9.00	54.29	1.00	22.00	0.00	4.00	1.00	15.00	60.00	0.00
1073.00	35.00	65.00	160.00	25.39	3.00	1.00	2.00	2.00	0.00	0.00	0.00	3.00	4.00	6.43	1.00	26.00	0.00	2.00	1.00	16.00	64.00	0.00
1074.00	34.00	80.00	173.00	26.73	3.00	1.00	2.00	2.00	1.00	0.00	1.00	3.00	7.00	8.57	1.00	33.00	1.00	6.00	0.00	18.00	72.00	0.00
1075.00	35.00	80.00	172.00	27.04	3.00	1.00	2.00	2.00	0.00	0.00	1.00	3.00	7.00	34.29	1.00	28.00	0.00	1.00	1.00	16.00	64.00	0.00
1076.00	38.00	96.00	184.00	28.36	3.00	1.00	2.00	2.00	0.00	0.00	1.00	3.00	4.00	36.43	1.00	30.00	1.00	5.00	1.00	15.00	60.00	0.00
1077.00	35.00	82.00	170.00	28.37	3.00	1.00	2.00	2.00	0.00	1.00	1.00	2.00	6.00	2.86	1.00	23.00	0.00	1.00	1.00	13.00	52.00	0.00
1078.00	35.00	65.00	161.00	25.08	3.00	1.00	2.00	2.00	1.00	1.00	0.00	3.00	6.00	7.14	1.00	26.00	0.00	9.00	0.00	15.00	60.00	0.00
1079.00	42.00	70.00	162.00	26.67	3.00	1.00	3.00	1.00	0.00	0.00	0.00	2.00	7.00	0.00	1.00	30.00	1.00	1.00	1.00	13.00	52.00	0.00
1080.00	44.00	100.00	190.00	27.70	3.00	1.00	3.00	2.00	1.00	0.00	1.00	3.00	6.50	12.86	1.00	25.00	0.00	6.00	0.00	16.00	64.00	0.00
1081.00	41.00	102.00	193.00	27.38	3.00	1.00	3.00	2.00	1.00	1.00	1.00	2.00	10.00	8.57	1.00	28.00	0.00	7.00	0.00	12.00	48.00	1.00
1082.00	42.00	74.00	174.00	24.44	2.00	1.00	1.00	2.00	1.00	1.00	1.00	1.00	8.00	0.00	1.00	32.00	1.00	5.00	1.00	14.00	56.00	0.00
1083.00	37.00	54.00	154.00	22.77	2.00	1.00	2.00	2.00	1.00	0.00	0.00	3.00	11.00	0.00	1.00	23.00	0.00	7.00	0.00	10.00	40.00	1.00
1084.00	40.00	58.00	163.00	21.83	2.00	1.00	2.00	2.00	1.00	1.00	1.00	1.00	6.00	1.43	1.00	26.00	0.00	7.00	0.00	8.00	32.00	1.00
1085.00	34.00	54.00	161.00	20.83	2.00	1.00	2.00	2.00	0.00	0.00	0.00	2.00	9.00	0.00	1.00	26.00	0.00	1.00	1.00	13.00	52.00	0.00
1086.00	40.00	69.00	167.00	24.74	2.00	1.00	2.00	2.00	0.00	0.00	0.00	3.00	6.00	30.00	1.00	19.00	0.00	4.00	1.00	15.00	60.00	0.00
1087.00	41.00	76.00	176.00	24.54	2.00	1.00	2.00	2.00	0.00	0.00	0.00	3.00	3.00	4.29	1.00	30.00	1.00	5.00	1.00	10.00	40.00	1.00
1088.00	38.00	70.00	171.00	23.94	2.00	1.00	2.00	2.00	1.00	0.00	1.00	3.00	7.00	1.43	1.00	23.00	0.00	5.00	1.00	16.00	64.00	0.00
1089.00	40.00	56.00	168.00	19.84	2.00	1.00	2.00	2.00	0.00	0.00	0.00	3.00	5.00	38.57	1.00	30.00	1.00	5.00	1.00	15.00	60.00	0.00
1090.00	38.00	60.00	157.00	24.34	2.00	0.00	2.00	2.00	0.00	1.00	0.00	1.00	6.50	0.71	1.00	35.00	1.00	10.00	0.00	12.00	48.00	1.00
1091.00	36.00	72.00	175.00	23.51	2.00	1.00	2.00	2.00	1.00	1.00	1.00	2.00	6.00	28.57	1.00	27.00	0.00	3.00	1.00	15.00	60.00	0.00
1092.00	36.00	80.00	180.00	24.69	2.00	1.00	2.00	2.00	1.00	1.00	1.00	2.00	8.00	10.00	1.00	19.00	0.00	7.00	0.00	17.00	68.00	0.00
1093.00	37.00	45.00	150.00	20.00	2.00	1.00	3.00	2.00	0.00	0.00	0.00	2.00	6.50	34.29	1.00	31.00	1.00	4.00	1.00	17.00	68.00	0.00
1094.00	39.00	82.00	175.00	26.78	3.00	0.00	2.00	2.00	1.00	0.00	1.00	2.00	9.00	0.00	1.00	23.00	0.00	11.00	0.00	14.00	56.00	0.00
1095.00	37.00	82.00	172.00	27.72	3.00	1.00	2.00	2.00	0.00	0.00	1.00	3.00	1.50	0.00	1.00	28.00	0.00	6.00	0.00	13.00	52.00	0.00
1096.00	37.00	81.00	178.00	25.56	3.00	1.00	2.00	2.00	0.00	1.00	1.00	3.00	6.50	60.00	1.00	29.00	0.00	4.00	1.00	15.00	60.00	0.00
1097.00	35.00	85.00	180.00	26.23	3.00	1.00	2.00	2.00	0.00	1.00	1.00	3.00	5.00	34.29	1.00	20.00	0.00	7.00	0.00	21.00	84.00	0.00
1098.00	40.00	88.00	178.00	27.77	3.00	1.00	3.00	2.00	0.00	0.00	1.00	3.00	7.00	8.57	1.00	29.00	0.00	5.00	1.00	12.00	48.00	1.00
1099.00	36.00	58.00	152.00	25.10	3.00	1.00	3.00	1.00	0.00	0.00	0.00	3.00	10.00	25.71	1.00	36.00	1.00	0.00	1.00	14.00	56.00	0.00
1100.00	44.00	88.00	185.00	25.71	3.00	1.00	3.00	2.00	1.00	0.00	1.00	3.00	7.00	4.29	1.00	23.00	0.00	4.00	1.00	12.00	48.00	1.00
1101.00	37.00	60.00	159.00	23.73	2.00	1.00	2.00	2.00	0.00	0.00	0.00	3.00	6.00	0.00	1.00	32.00	1.00	5.00	1.00	14.00	56.00	0.00
1102.00	40.00	73.00	171.00	24.96	2.00	1.00	2.00	2.00	1.00	1.00	1.00	3.00	6.50	6.43	1.00	34.00	1.00	2.00	1.00	12.00	48.00	1.00
1103.00	44.00	90.00	168.00	31.89	4.00	0.00	1.00	3.00	0.00	0.00	0.00	3.00	4.00	17.14	1.00	37.00	1.00	4.00	1.00	12.00	48.00	1.00
1104.00	36.00	100.00	178.00	31.56	4.00	1.00	2.00	1.00	1.00	0.00	1.00	3.00	7.00	8.57	1.00	29.00	0.00	3.00	1.00	17.00	68.00	0.00
1105.00	41.00	75.00	170.00	25.95	3.00	1.00	1.00	2.00	1.00	1.00	1.00	1.00	3.00	8.57	1.00	24.00	0.00	5.00	1.00	13.00	52.00	0.00
1106.00	34.00	70.00	163.00	26.35	3.00	1.00	2.00	2.00	0.00	1.00	0.00	1.00	2.50	8.57	1.00	32.00	1.00	6.00	0.00	10.00	40.00	1.00
1107.00	37.00	56.00	170.00	19.38	2.00	1.00	1.00	2.00	0.00	0.00	0.00	3.00	4.00	2.86	1.00	33.00	1.00	4.00	1.00	16.00	64.00	0.00
1108.00	44.00	75.00	182.00	22.64	2.00	1.00	1.00	3.00	1.00	0.00	1.00	3.00	4.00	55.71	1.00	28.00	0.00	4.00	1.00	10.00	40.00	1.00
1109.00	35.00	76.00	183.00	22.69	2.00	0.00	1.00	2.00	1.00	1.00	1.00	2.00	9.00	60.00	1.00	24.00	0.00	6.00	0.00	6.00	24.00	1.00
1110.00	38.00	60.00	167.00	21.51	2.00	1.00	2.00	2.00	0.00	0.00	0.00	2.00	1.00	51.43	1.00	33.00	1.00	2.00	1.00	12.00	48.00	1.00
1111.00	43.00	60.00	182.00	18.11	1.00	0.00	1.00	2.00	0.00	0.00	1.00	3.00	3.50	5.71	1.00	35.00	1.00	4.00	1.00	13.00	52.00	0.00
1112.00	38.00	90.00	178.00	28.41	3.00	1.00	2.00	2.00	0.00	0.00	1.00	3.00	5.50	47.14	1.00	29.00	0.00	3.00	1.00	16.00	64.00	0.00
1113.00	35.00	87.00	178.00	27.46	3.00	1.00	2.00	1.00	1.00	0.00	1.00	3.00	2.00	42.86	1.00	33.00	1.00	5.00	1.00	19.00	76.00	0.00
1114.00	39.00	77.00	175.00	25.14	3.00	1.00	2.00	2.00	0.00	0.00	1.00	3.00	6.00	4.00	1.00	27.00	0.00	8.00	0.00	13.00	52.00	0.00
1115.00	35.00	62.00	168.00	21.97	2.00	1.00	1.00	1.00	1.00	1.00	0.00	3.00	8.00	39.29	1.00	26.00	0.00	5.00	1.00	9.00	36.00	1.00
1116.00	44.00	75.00	158.00	30.04	4.00	1.00	2.00	2.00	0.00	0.00	0.00	2.00	4.00	4.29	1.00	26.00	0.00	1.00	1.00	13.00	52.00	0.00
1117.00	40.00	104.00	167.00	37.29	4.00	1.00	2.00	2.00	0.00	1.00	0.00	3.00	5.00	25.71	1.00	31.00	1.00	3.00	1.00	19.00	76.00	0.00
1118.00	39.00	88.00	168.00	31.18	4.00	1.00	2.00	2.00	0.00	1.00	0.00	3.00	2.50	30.00	1.00	31.00	1.00	5.00	1.00	16.00	64.00	0.00
1119.00	42.00	95.00	165.00	34.89	4.00	1.00	2.00	2.00	0.00	0.00	0.00	2.00	2.00	25.71	1.00	37.00	1.00	9.00	0.00	14.00	56.00	0.00
1120.00	37.00	78.00	159.00	30.85	4.00	1.00	2.00	2.00	0.00	0.00	0.00	3.00	7.00	8.57	1.00	32.00	1.00	8.00	0.00	12.00	48.00	1.00
1121.00	41.00	88.00	168.00	31.18	4.00	1.00	2.00	2.00	1.00	0.00	0.00	3.00	7.00	0.00	1.00	35.00	1.00	8.00	0.00	14.00	56.00	0.00
1122.00	40.00	92.00	170.00	31.83	4.00	1.00	2.00	2.00	0.00	1.00	0.00	2.00	3.00	0.00	1.00	23.00	0.00	8.00	0.00	9.00	36.00	1.00

Subject	Age	Weight	Height	BMI	BMI_C	Residence	Education	Marital_Status	Smoking	Chronic_Dise	Gender	Health	Sitting_Time	PA_Min_Day	IPAQ_C	Dietary_Score	Dietary_C	PSQI_Total	Sleep_C	WHO5_Total	WHO5_Total100	WHO5_Class
1123.00	44.00	90.00	170.00	31.14	4.00	1.00	2.00	2.00	0.00	1.00	0.00	3.00	6.50	60.00	1.00	30.00	1.00	2.00	1.00	15.00	60.00	0.00
1124.00	34.00	95.00	168.00	33.66	4.00	1.00	3.00	2.00	1.00	0.00	0.00	2.00	6.00	25.71	1.00	28.00	0.00	7.00	0.00	15.00	60.00	0.00
1125.00	39.00	72.00	164.00	26.77	3.00	1.00	1.00	2.00	0.00	0.00	0.00	2.00	4.00	0.00	1.00	23.00	0.00	6.00	0.00	9.00	36.00	1.00
1126.00	42.00	70.00	164.00	26.03	3.00	1.00	1.00	2.00	0.00	1.00	0.00	3.00	4.00	17.14	1.00	22.00	0.00	5.00	1.00	9.00	36.00	1.00
1127.00	40.00	70.00	164.00	26.03	3.00	1.00	1.00	2.00	0.00	0.00	0.00	3.00	10.00	4.29	1.00	28.00	0.00	5.00	1.00	14.00	56.00	0.00
1128.00	42.00	73.00	165.00	26.81	3.00	1.00	1.00	2.00	0.00	0.00	0.00	3.00	5.00	15.71	1.00	20.00	0.00	3.00	1.00	16.00	64.00	0.00
1129.00	37.00	80.00	167.00	28.69	3.00	1.00	1.00	2.00	1.00	1.00	0.00	2.00	6.00	25.71	1.00	19.00	0.00	10.00	0.00	6.00	24.00	1.00
1130.00	40.00	64.00	159.00	25.32	3.00	1.00	1.00	2.00	0.00	1.00	0.00	3.00	6.00	34.29	1.00	28.00	0.00	0.00	1.00	19.00	76.00	0.00
1131.00	39.00	70.00	160.00	27.34	3.00	0.00	1.00	2.00	0.00	1.00	0.00	3.00	2.00	38.71	1.00	40.00	1.00	1.00	1.00	13.00	52.00	0.00
1132.00	36.00	70.00	164.00	26.03	3.00	1.00	2.00	2.00	0.00	1.00	0.00	2.00	11.00	0.00	1.00	31.00	1.00	8.00	0.00	12.00	48.00	1.00
1133.00	42.00	80.00	164.00	29.74	3.00	1.00	2.00	2.00	0.00	1.00	0.00	2.00	4.00	4.29	1.00	38.00	1.00	2.00	1.00	12.00	48.00	1.00
1134.00	35.00	70.00	163.00	26.35	3.00	1.00	2.00	2.00	0.00	1.00	0.00	3.00	3.50	0.00	1.00	31.00	1.00	4.00	1.00	11.00	44.00	1.00
1135.00	44.00	74.00	161.00	28.55	3.00	1.00	2.00	2.00	0.00	0.00	0.00	3.00	4.00	19.29	1.00	32.00	1.00	8.00	0.00	15.00	60.00	0.00
1136.00	34.00	76.00	175.00	24.82	2.00	1.00	1.00	2.00	0.00	1.00	0.00	2.00	5.00	14.29	1.00	31.00	1.00	5.00	1.00	13.00	52.00	0.00
1137.00	34.00	70.00	172.00	23.66	2.00	1.00	1.00	1.00	0.00	0.00	0.00	3.00	4.00	20.14	1.00	27.00	0.00	6.00	0.00	14.00	56.00	0.00
1138.00	41.00	64.00	163.00	24.09	2.00	1.00	1.00	2.00	1.00	1.00	0.00	2.00	1.50	4.29	1.00	31.00	1.00	8.00	0.00	9.00	36.00	1.00
1139.00	41.00	64.00	164.00	23.80	2.00	1.00	2.00	2.00	0.00	0.00	0.00	3.00	6.50	38.57	1.00	21.00	0.00	4.00	1.00	8.00	32.00	1.00
1140.00	44.00	65.00	165.00	23.88	2.00	1.00	2.00	2.00	0.00	0.00	0.00	3.00	1.50	28.57	1.00	32.00	1.00	4.00	1.00	12.00	48.00	1.00
1141.00	34.00	56.00	158.00	22.43	2.00	1.00	2.00	2.00	0.00	0.00	0.00	2.00	5.00	0.00	1.00	27.00	0.00	11.00	0.00	11.00	44.00	1.00
1142.00	35.00	67.00	165.00	24.61	2.00	1.00	2.00	2.00	0.00	0.00	0.00	3.00	7.00	25.71	1.00	29.00	0.00	2.00	1.00	14.00	56.00	0.00
1143.00	36.00	70.00	173.00	23.39	2.00	1.00	2.00	2.00	1.00	0.00	0.00	3.00	4.00	0.00	1.00	27.00	0.00	8.00	0.00	13.00	52.00	0.00
1144.00	35.00	58.00	176.00	18.72	2.00	1.00	2.00	2.00	0.00	0.00	0.00	3.00	4.00	4.29	1.00	27.00	0.00	3.00	1.00	20.00	80.00	0.00
1145.00	38.00	49.00	158.00	19.63	2.00	1.00	2.00	2.00	1.00	0.00	0.00	3.00	4.50	0.00	1.00	28.00	0.00	6.00	0.00	15.00	60.00	0.00
1146.00	35.00	61.00	163.00	22.96	2.00	1.00	2.00	2.00	1.00	1.00	0.00	2.00	9.00	0.00	1.00	30.00	1.00	5.00	1.00	11.00	44.00	1.00
1147.00	41.00	67.00	167.00	24.02	2.00	1.00	2.00	2.00	0.00	1.00	0.00	2.00	6.00	8.57	1.00	28.00	0.00	5.00	1.00	16.00	64.00	0.00
1148.00	40.00	63.00	167.00	22.59	2.00	1.00	2.00	2.00	0.00	1.00	0.00	3.00	9.00	25.71	1.00	28.00	0.00	6.00	0.00	11.00	44.00	1.00
1149.00	39.00	63.00	162.00	24.01	2.00	1.00	2.00	2.00	1.00	1.00	0.00	3.00	5.00	0.00	1.00	28.00	0.00	4.00	1.00	12.00	48.00	1.00
1150.00	40.00	65.00	168.00	23.03	2.00	1.00	2.00	2.00	0.00	1.00	0.00	3.00	4.50	0.00	1.00	31.00	1.00	3.00	1.00	15.00	60.00	0.00
1151.00	44.00	95.00	192.00	25.77	3.00	1.00	2.00	2.00	1.00	1.00	1.00	3.00	10.00	22.86	1.00	28.00	0.00	6.00	0.00	10.00	40.00	1.00
1152.00	44.00	95.00	192.00	25.77	3.00	1.00	2.00	2.00	1.00	1.00	1.00	3.00	11.00	60.00	1.00	27.00	0.00	2.00	1.00	18.00	72.00	0.00
1153.00	37.00	90.00	178.00	28.41	3.00	1.00	3.00	1.00	1.00	0.00	1.00	3.00	9.00	2.14	1.00	31.00	1.00	6.00	0.00	9.00	36.00	1.00
1154.00	42.00	83.00	176.00	26.79	3.00	1.00	3.00	2.00	0.00	1.00	1.00	2.00	8.00	8.57	1.00	23.00	0.00	9.00	0.00	6.00	24.00	1.00
1155.00	38.00	77.00	176.00	24.86	2.00	1.00	3.00	2.00	0.00	0.00	1.00	2.00	7.00	4.29	1.00	29.00	0.00	2.00	1.00	12.00	48.00	1.00
1156.00	40.00	95.00	165.00	34.89	4.00	1.00	2.00	2.00	1.00	0.00	0.00	3.00	8.00	4.29	1.00	24.00	0.00	7.00	0.00	10.00	40.00	1.00
1157.00	41.00	91.00	187.00	26.02	3.00	0.00	2.00	2.00	0.00	0.00	1.00	3.00	4.00	27.86	1.00	21.00	0.00	4.00	1.00	15.00	60.00	0.00
1158.00	41.00	85.00	178.00	26.83	3.00	1.00	2.00	2.00	1.00	1.00	1.00	2.00	4.00	27.86	1.00	21.00	0.00	3.00	1.00	11.00	44.00	1.00
1159.00	41.00	87.00	179.00	27.15	3.00	0.00	2.00	1.00	1.00	1.00	1.00	2.00	3.00	32.14	1.00	23.00	0.00	3.00	1.00	14.00	56.00	0.00
1160.00	37.00	86.00	165.00	31.59	4.00	1.00	2.00	2.00	0.00	0.00	0.00	2.00	6.00	12.86	1.00	29.00	0.00	6.00	0.00	11.00	44.00	1.00
1161.00	40.00	112.00	183.00	33.44	4.00	1.00	3.00	2.00	1.00	0.00	1.00	3.00	10.00	2.14	1.00	29.00	0.00	5.00	1.00	15.00	60.00	0.00
1162.00	37.00	99.00	181.00	30.22	4.00	1.00	3.00	2.00	0.00	1.00	1.00	3.00	5.00	10.71	1.00	30.00	1.00	4.00	1.00	14.00	56.00	0.00
1163.00	39.00	90.00	172.00	30.42	4.00	1.00	3.00	2.00	1.00	1.00	1.00	3.00	10.00	34.29	1.00	31.00	1.00	2.00	1.00	13.00	52.00	0.00
1164.00	34.00	69.00	160.00	26.95	3.00	1.00	2.00	3.00	0.00	0.00	0.00	3.00	6.00	0.00	1.00	30.00	1.00	3.00	1.00	13.00	52.00	0.00
1165.00	39.00	82.00	177.00	26.17	3.00	1.00	3.00	2.00	0.00	1.00	0.00	3.00	2.50	8.57	1.00	29.00	0.00	8.00	0.00	16.00	64.00	0.00
1166.00	43.00	74.00	170.00	25.61	3.00	1.00	3.00	2.00	1.00	1.00	1.00	3.00	6.00	21.43	1.00	34.00	1.00	1.00	1.00	17.00	68.00	0.00
1167.00	38.00	55.00	162.00	20.96	2.00	1.00	1.00	1.00	0.00	0.00	0.00	1.00	7.00	0.00	1.00	17.00	0.00	9.00	0.00	11.00	44.00	1.00
1168.00	44.00	58.00	161.00	22.38	2.00	1.00	2.00	2.00	0.00	0.00	0.00	3.00	7.00	25.71	1.00	32.00	1.00	6.00	0.00	13.00	52.00	0.00
1169.00	40.00	54.00	157.00	21.91	2.00	1.00	3.00	2.00	0.00	0.00	0.00	3.00	2.00	8.57	1.00	33.00	1.00	4.00	1.00	10.00	40.00	1.00
1170.00	43.00	77.50	169.00	27.13	3.00	1.00	2.00	2.00	1.00	1.00	1.00	2.00	4.50	55.71	1.00	23.00	0.00	7.00	0.00	11.00	44.00	1.00
1171.00	42.00	75.00	165.00	27.55	3.00	1.00	2.00	3.00	1.00	0.00	0.00	1.00	6.00	0.00	1.00	24.00	0.00	4.00	1.00	12.00	48.00	1.00
1172.00	40.00	65.00	150.00	28.89	3.00	0.00	2.00	2.00	0.00	0.00	0.00	3.00	6.00	0.00	1.00	23.00	0.00	14.00	0.00	7.00	28.00	1.00
1173.00	35.00	90.00	170.00	31.14	4.00	1.00	3.00	2.00	0.00	0.00	1.00	1.00	9.00	0.00	1.00	25.00	0.00	2.00	1.00	9.00	36.00	1.00
1174.00	43.00	117.00	165.00	42.98	4.00	1.00	2.00	2.00	0.00	0.00	0.00	1.00	11.00	6.43	1.00	27.00	0.00	12.00	0.00	9.00	36.00	1.00
1175.00	39.00	85.00	164.00	31.60	4.00	1.00	2.00	2.00	1.00	0.00	0.00	2.00	10.00	51.43	1.00	28.00	0.00	9.00	0.00	2.00	8.00	1.00
1176.00	43.00	76.00	158.00	30.44	4.00	1.00	2.00	3.00	0.00	0.00	0.00	2.00	9.00	0.00	1.00	19.00	0.00	4.00	1.00	18.00	72.00	0.00
1177.00	43.00	76.00	158.00	30.44	4.00	1.00	2.00	1.00	0.00	0.00	0.00	2.00	5.50	17.14	1.00	20.00	0.00	2.00	1.00	17.00	68.00	0.00
1178.00	34.00	87.00	167.00	31.20	4.00	1.00	2.00	2.00	0.00	0.00	0.00	3.00	4.00	19.29	1.00	32.00	1.00	6.00	0.00	13.00	52.00	0.00
1179.00	40.00	98.00	157.00	39.76	4.00	1.00	2.00	2.00	0.00	0.00	0.00	3.00	7.00	10.00	1.00	32.00	1.00	7.00	0.00	15.00	60.00	0.00
1180.00	38.00	102.00	174.00	33.69	4.00	0.00	2.00	2.00	0.00	0.00	1.00	3.00	11.00	8.57	1.00	21.00	0.00	5.00	1.00	18.00	72.00	0.00
1181.00	37.00	100.00	172.00	33.80	4.00	1.00	2.00	2.00	0.00	0.00	1.00	3.00	2.00	25.71	1.00	28.00	0.00	3.00	1.00	13.00	52.00	0.00
1182.00	43.00	130.00	184.00	38.40	4.00	1.00	2.00	2.00	0.00	0.00	1.00	3.00	7.00	8.57	1.00	36.00	1.00	5.00	1.00	17.00	68.00	0.00
1183.00	37.00	105.00	182.00	31.70	4.00	1.00	2.00	3.00	1.00	1.00	1.00	2.00	5.50	11.43	1.00	27.00	0.00	4.00	1.00	14.00	56.00	0.00
1184.00	41.00	92.00	172.00	31.10	4.00	0.00	2.00	2.00	1.00	1.00	1.00	3.00	9.00	7.14	1.00	29.00	0.00	7.00	0.00	18.00	72.00	0.00
1185.00	42.00	80.00	163.00	30.11	4.00	1.00	3.00	1.00	0.00	0.00	0.00	2.00	7.00	19.29	1.00	28.00	0.00	5.00	1.00	13.00	52.00	0.00
1186.00	35.00	96.00	150.00	42.67	4.00	1.00	3.00	2.00	1.00	1.00	0.00	1.00	7.00	5.71	1.00	25.00	0.00	7.00	0.00	6.00	24.00	1.00
1187.00	38.00	76.00	168.00	26.93	3.00	0.00	1.00	2.00	1.00	0.00	1.00	3.00	6.00	40.00	1.00	29.00	0.00	8.00	0.00	13.00	52.00	0.00
1188.00	42.00	78.00	165.00	28.65	3.00	1.00	2.00	2.00	0.00	0.00	1.00	3.00	4.00	0.00	1.00	36.00	1.00	3.00	1.00	12.00	48.00	1.00

Subject	Age	Weight	Height	BMI	BMI_C	Residence	Education	Marital_Status	Smoking	Chronic_Dise	Gender	Health	Sitting_Time	PA_Min_Day	IPAQ_C	Dietary_Score	Dietary_C	PSQI_Total	Sleep_C	WHO5_Total	WHO5_Total100	WHO5_Class
1189.00	42.00	69.00	156.00	28.35	3.00	1.00	2.00	2.00	0.00	1.00	0.00	3.00	6.00	46.43	1.00	29.00	0.00	3.00	1.00	14.00	56.00	0.00
1190.00	40.00	70.00	160.00	27.34	3.00	1.00	2.00	2.00	0.00	0.00	0.00	2.00	4.00	25.71	1.00	27.00	0.00	7.00	0.00	12.00	48.00	1.00
1191.00	43.00	76.00	173.00	25.39	3.00	1.00	2.00	2.00	0.00	0.00	1.00	3.00	8.00	48.57	1.00	25.00	0.00	8.00	0.00	13.00	52.00	0.00
1192.00	35.00	87.00	183.00	25.98	3.00	1.00	2.00	2.00	1.00	0.00	1.00	3.00	6.00	34.29	1.00	38.00	1.00	2.00	1.00	15.00	60.00	0.00
1193.00	40.00	80.00	175.00	26.12	3.00	1.00	2.00	2.00	0.00	0.00	1.00	3.00	5.00	24.29	1.00	22.00	0.00	7.00	0.00	15.00	60.00	0.00
1194.00	37.00	76.00	166.00	27.58	3.00	1.00	2.00	2.00	1.00	1.00	1.00	1.00	10.00	0.00	1.00	16.00	0.00	7.00	0.00	15.00	60.00	0.00
1195.00	40.00	98.00	188.00	27.73	3.00	1.00	2.00	2.00	1.00	1.00	1.00	2.00	10.00	6.43	1.00	29.00	0.00	5.00	1.00	11.00	44.00	1.00
1196.00	38.00	70.00	154.00	29.52	3.00	1.00	2.00	2.00	0.00	1.00	0.00	3.00	7.00	2.14	1.00	26.00	0.00	12.00	0.00	14.00	56.00	0.00
1197.00	39.00	80.00	175.00	26.12	3.00	1.00	3.00	2.00	0.00	0.00	1.00	2.00	6.50	1.43	1.00	30.00	1.00	11.00	0.00	9.00	36.00	1.00
1198.00	43.00	75.00	167.00	26.89	3.00	1.00	3.00	2.00	1.00	0.00	0.00	3.00	9.00	4.29	1.00	35.00	1.00	5.00	1.00	15.00	60.00	0.00
1199.00	34.00	64.00	172.00	21.63	2.00	1.00	1.00	3.00	1.00	0.00	0.00	3.00	2.00	0.00	1.00	33.00	1.00	4.00	1.00	13.00	52.00	0.00
1200.00	39.00	75.00	189.00	21.00	2.00	1.00	2.00	2.00	0.00	0.00	0.00	3.00	4.00	17.14	1.00	23.00	0.00	5.00	1.00	10.00	40.00	1.00
1201.00	39.00	58.00	165.00	21.30	2.00	0.00	2.00	2.00	1.00	0.00	0.00	3.00	8.00	15.71	1.00	34.00	1.00	10.00	0.00	12.00	48.00	1.00
1202.00	42.00	60.00	156.00	24.65	2.00	0.00	2.00	2.00	0.00	0.00	0.00	3.00	6.00	12.86	1.00	28.00	0.00	2.00	1.00	14.00	56.00	0.00
1203.00	42.00	70.00	172.00	23.66	2.00	1.00	2.00	2.00	0.00	0.00	0.00	3.00	4.50	30.00	1.00	36.00	1.00	87.21	0.00	16.00	64.00	0.00
1204.00	37.00	69.00	174.00	22.79	2.00	1.00	2.00	2.00	0.00	1.00	1.00	2.00	8.00	68.57	1.00	30.00	1.00	0.00	1.00	14.00	56.00	0.00
1205.00	37.00	70.00	173.00	23.39	2.00	1.00	2.00	2.00	1.00	0.00	0.00	2.00	3.00	0.00	1.00	30.00	1.00	4.00	1.00	11.00	44.00	1.00
1206.00	37.00	56.00	165.00	20.57	2.00	1.00	2.00	2.00	1.00	0.00	0.00	2.00	5.50	12.86	1.00	24.00	0.00	10.00	0.00	7.00	28.00	1.00
1207.00	38.00	55.00	165.00	20.20	2.00	1.00	2.00	2.00	1.00	0.00	0.00	2.00	7.00	53.57	1.00	33.00	1.00	1.00	1.00	12.00	48.00	1.00
1208.00	43.00	60.00	156.00	24.65	2.00	0.00	2.00	2.00	0.00	0.00	0.00	3.00	7.00	25.71	1.00	36.00	1.00	7.00	0.00	9.00	36.00	1.00
1209.00	42.00	61.00	159.00	24.13	2.00	1.00	2.00	2.00	0.00	0.00	0.00	3.00	6.00	0.00	1.00	18.00	0.00	7.00	0.00	10.00	40.00	1.00
1210.00	39.00	78.00	183.00	23.29	2.00	1.00	2.00	2.00	0.00	0.00	1.00	3.00	5.00	39.29	1.00	32.00	1.00	8.00	0.00	10.00	40.00	1.00
1211.00	39.00	72.00	176.00	23.24	2.00	0.00	2.00	2.00	0.00	0.00	1.00	3.00	4.00	36.00	1.00	30.00	1.00	11.00	0.00	16.00	64.00	0.00
1212.00	40.00	75.00	178.00	23.67	2.00	1.00	2.00	2.00	1.00	1.00	1.00	2.00	4.00	12.86	1.00	29.00	0.00	6.00	0.00	12.00	48.00	1.00
1213.00	39.00	74.00	175.00	24.16	2.00	1.00	2.00	2.00	1.00	1.00	1.00	2.00	5.00	5.71	1.00	29.00	0.00	6.00	0.00	11.00	44.00	1.00
1214.00	40.00	70.00	168.00	24.80	2.00	1.00	3.00	2.00	0.00	0.00	0.00	2.00	6.50	12.86	1.00	32.00	1.00	3.00	1.00	16.00	64.00	0.00
1215.00	35.00	70.00	168.00	24.80	2.00	1.00	3.00	2.00	1.00	0.00	1.00	3.00	5.00	18.57	1.00	28.00	0.00	8.00	0.00	9.00	36.00	1.00
1216.00	38.00	77.00	177.00	24.58	2.00	1.00	3.00	2.00	0.00	0.00	1.00	3.00	6.00	38.57	1.00	32.00	1.00	9.00	0.00	12.00	48.00	1.00
1217.00	35.00	74.00	173.00	24.73	2.00	1.00	3.00	2.00	0.00	0.00	1.00	3.00	11.00	2.86	1.00	24.00	0.00	4.00	1.00	12.00	48.00	1.00
1218.00	39.00	60.00	160.00	23.44	2.00	1.00	3.00	3.00	0.00	0.00	0.00	3.00	3.50	21.43	1.00	38.00	1.00	3.00	1.00	19.00	76.00	0.00
1219.00	39.00	55.00	158.00	22.03	2.00	1.00	3.00	1.00	1.00	0.00	0.00	3.00	9.00	0.00	1.00	22.00	0.00	4.00	1.00	18.00	72.00	0.00
1220.00	38.00	88.00	188.00	24.90	2.00	1.00	3.00	2.00	0.00	1.00	1.00	1.00	2.50	8.57	1.00	30.00	1.00	5.00	1.00	12.00	48.00	1.00
1221.00	39.00	54.00	173.00	18.04	1.00	1.00	2.00	2.00	1.00	1.00	1.00	3.00	9.00	1.43	1.00	30.00	1.00	3.00	1.00	13.00	52.00	0.00
1222.00	42.00	85.00	155.00	35.38	4.00	1.00	2.00	2.00	0.00	0.00	0.00	3.00	1.50	0.00	1.00	24.00	0.00	3.00	1.00	13.00	52.00	0.00
1223.00	36.00	58.00	156.00	23.83	2.00	1.00	2.00	2.00	0.00	0.00	0.00	2.00	5.00	0.00	1.00	30.00	1.00	7.00	0.00	11.00	44.00	1.00
1224.00	40.00	70.00	170.00	24.22	2.00	1.00	2.00	2.00	0.00	0.00	0.00	2.00	2.50	0.00	1.00	28.00	0.00	6.00	0.00	16.00	64.00	0.00
1225.00	39.00	92.00	160.00	35.94	4.00	1.00	3.00	2.00	0.00	0.00	0.00	2.00	7.00	21.43	1.00	31.00	1.00	4.00	1.00	20.00	80.00	0.00
1226.00	41.00	110.00	177.00	35.11	4.00	1.00	3.00	2.00	0.00	0.00	1.00	3.00	7.00	17.14	1.00	22.00	0.00	5.00	1.00	10.00	40.00	1.00
1227.00	44.00	90.00	158.00	36.05	4.00	1.00	3.00	2.00	0.00	0.00	0.00	3.00	4.00	2.14	1.00	26.00	0.00	3.00	1.00	15.00	60.00	0.00
1228.00	37.00	115.00	186.00	33.24	4.00	0.00	3.00	3.00	0.00	0.00	1.00	3.00	7.00	17.14	1.00	34.00	1.00	4.00	1.00	15.00	60.00	0.00
1229.00	36.00	100.00	171.00	34.20	4.00	1.00	3.00	2.00	0.00	0.00	0.00	3.00	5.00	20.00	1.00	24.00	0.00	9.00	0.00	10.00	40.00	1.00
1230.00	40.00	80.00	163.00	30.11	4.00	1.00	3.00	2.00	0.00	0.00	0.00	3.00	5.00	0.00	1.00	27.00	0.00	4.00	1.00	16.00	64.00	0.00
1231.00	36.00	90.00	171.00	30.78	4.00	1.00	3.00	2.00	1.00	0.00	1.00	3.00	5.00	4.29	1.00	34.00	1.00	7.00	0.00	13.00	52.00	0.00
1232.00	41.00	95.00	175.00	31.02	4.00	1.00	3.00	2.00	0.00	0.00	1.00	3.00	8.00	25.71	1.00	34.00	1.00	6.00	0.00	15.00	60.00	0.00
1233.00	34.00	98.00	175.00	32.00	4.00	1.00	3.00	2.00	1.00	0.00	1.00	3.00	6.00	28.57	1.00	33.00	1.00	0.00	1.00	23.00	92.00	0.00
1234.00	35.00	98.00	169.00	34.31	4.00	1.00	3.00	2.00	0.00	0.00	1.00	3.00	9.00	19.29	1.00	31.00	1.00	5.00	1.00	15.00	60.00	0.00
1235.00	40.00	115.00	179.00	35.89	4.00	1.00	3.00	2.00	0.00	0.00	1.00	3.00	11.00	45.00	1.00	31.00	1.00	4.00	1.00	12.00	48.00	1.00
1236.00	43.00	90.00	167.00	32.27	4.00	1.00	3.00	2.00	0.00	1.00	0.00	1.00	6.00	0.71	1.00	23.00	0.00	10.00	0.00	9.00	36.00	1.00
1237.00	43.00	106.00	180.00	32.72	4.00	1.00	3.00	2.00	0.00	1.00	1.00	2.00	10.00	27.86	1.00	32.00	1.00	6.00	0.00	16.00	64.00	0.00
1238.00	38.00	115.00	174.00	37.98	4.00	0.00	3.00	2.00	0.00	1.00	1.00	2.00	6.00	51.43	1.00	32.00	1.00	5.00	1.00	12.00	48.00	1.00
1239.00	43.00	80.00	162.00	30.48	4.00	1.00	3.00	2.00	0.00	1.00	0.00	3.00	6.50	0.00	1.00	32.00	1.00	6.00	0.00	12.00	48.00	1.00
1240.00	39.00	82.00	156.00	33.69	4.00	1.00	3.00	2.00	0.00	1.00	0.00	3.00	9.00	0.00	1.00	24.00	0.00	7.00	0.00	11.00	44.00	1.00
1241.00	41.00	80.00	170.00	27.68	3.00	1.00	1.00	2.00	0.00	0.00	0.00	2.00	2.50	5.71	1.00	23.00	0.00	4.00	1.00	16.00	64.00	0.00
1242.00	40.00	123.00	204.00	29.56	3.00	0.00	2.00	2.00	0.00	0.00	1.00	3.00	9.00	21.57	1.00	27.00	0.00	4.00	1.00	9.00	36.00	1.00
1243.00	42.00	76.00	174.00	25.10	3.00	1.00	3.00	2.00	0.00	0.00	1.00	1.00	3.00	18.57	1.00	23.00	0.00	9.00	0.00	13.00	52.00	0.00
1244.00	37.00	64.00	157.00	25.96	3.00	1.00	3.00	2.00	0.00	0.00	0.00	1.00	7.00	17.86	1.00	24.00	0.00	10.00	0.00	17.00	68.00	0.00
1245.00	44.00	72.00	165.00	26.45	3.00	1.00	3.00	2.00	0.00	0.00	1.00	2.00	6.00	0.00	1.00	26.00	0.00	8.00	0.00	11.00	44.00	1.00
1246.00	44.00	73.00	169.00	25.56	3.00	1.00	3.00	2.00	0.00	0.00	0.00	2.00	9.00	17.14	1.00	29.00	0.00	3.00	1.00	9.00	36.00	1.00
1247.00	43.00	87.00	173.00	29.07	3.00	1.00	3.00	2.00	0.00	0.00	1.00	2.00	3.50	21.43	1.00	26.00	0.00	5.00	1.00	11.00	44.00	1.00
1248.00	43.00	77.00	162.00	29.34	3.00	1.00	3.00	1.00	0.00	0.00	0.00	2.00	9.00	0.00	1.00	27.00	0.00	4.00	1.00	17.00	68.00	0.00
1249.00	41.00	82.00	172.00	27.72	3.00	1.00	3.00	2.00	0.00	0.00	1.00	2.00	10.00	6.43	1.00	19.00	0.00	4.00	1.00	11.00	44.00	1.00
1250.00	44.00	73.00	161.00	28.16	3.00	1.00	3.00	2.00	0.00	0.00	0.00	3.00	7.00	8.86	1.00	23.00	0.00	7.00	0.00	10.00	40.00	1.00
1251.00	36.00	80.00	168.00	28.34	3.00	1.00	3.00	2.00	0.00	0.00	0.00	3.00	4.50	2.14	1.00	27.00	0.00	12.00	0.00	13.00	52.00	0.00
1252.00	40.00	70.00	167.00	25.10	3.00	1.00	3.00	2.00	0.00	0.00	1.00	3.00	7.00	4.29	1.00	23.00	0.00	7.00	0.00	8.00	32.00	1.00
1253.00	42.00	100.00	187.00	28.60	3.00	1.00	3.00	2.00	0.00	0.00	1.00	3.00	8.00	12.86	1.00	23.00	0.00	6.00	0.00	9.00	36.00	1.00
1254.00	42.00	59.00	152.00	25.54	3.00	1.00	3.00	2.00	0.00	0.00	0.00	3.00	7.00	2.86	1.00	27.00	0.00	5.00	1.00	14.00	56.00	0.00

Subject	Age	Weight	Height	BMI	BMI_C	Residence	Education	Marital_Status	Smoking	Chronic_Dise	Gender	Health	Sitting_Time	PA_Min_Day	IPAQ_C	Dietary_Score	Dietary_C	PSQI_Total	Sleep_C	WHO5_Total	WHO5_Total100	WHO5_Class
1255.00	35.00	95.00	189.00	26.60	3.00	1.00	3.00	2.00	0.00	0.00	1.00	3.00	5.50	53.57	1.00	28.00	0.00	3.00	1.00	13.00	52.00	0.00
1256.00	40.00	65.00	160.00	25.39	3.00	1.00	3.00	2.00	0.00	0.00	0.00	3.00	5.00	17.14	1.00	32.00	1.00	3.00	1.00	20.00	80.00	0.00
1257.00	40.00	73.00	160.00	28.52	3.00	1.00	3.00	2.00	0.00	0.00	0.00	3.00	4.00	31.43	1.00	33.00	1.00	5.00	1.00	18.00	72.00	0.00
1258.00	39.00	83.00	172.00	28.06	3.00	1.00	3.00	2.00	0.00	0.00	1.00	3.00	5.00	60.00	1.00	26.00	0.00	4.00	1.00	18.00	72.00	0.00
1259.00	43.00	85.00	172.00	28.73	3.00	1.00	3.00	2.00	0.00	0.00	1.00	3.00	3.00	33.57	1.00	37.00	1.00	1.00	1.00	15.00	60.00	0.00
1260.00	41.00	75.00	173.00	25.06	3.00	1.00	3.00	2.00	0.00	0.00	1.00	3.00	4.00	48.43	1.00	29.00	0.00	2.00	1.00	15.00	60.00	0.00
1261.00	38.00	80.00	176.00	25.83	3.00	1.00	3.00	1.00	0.00	0.00	0.00	3.00	6.50	25.71	1.00	20.00	0.00	2.00	1.00	12.00	48.00	1.00
1262.00	38.00	90.00	187.00	25.74	3.00	1.00	3.00	3.00	0.00	0.00	1.00	3.00	6.00	45.71	1.00	29.00	0.00	3.00	1.00	15.00	60.00	0.00
1263.00	38.00	78.00	170.00	26.99	3.00	1.00	3.00	2.00	0.00	0.00	0.00	3.00	4.00	34.29	1.00	31.00	1.00	0.00	1.00	14.00	56.00	0.00
1264.00	42.00	79.00	176.00	25.50	3.00	1.00	3.00	2.00	0.00	0.00	1.00	3.00	7.00	21.43	1.00	36.00	1.00	2.00	1.00	15.00	60.00	0.00
1265.00	41.00	90.00	180.00	27.78	3.00	1.00	3.00	3.00	0.00	0.00	1.00	3.00	6.00	4.29	1.00	29.00	0.00	6.00	0.00	16.00	64.00	0.00
1266.00	40.00	68.00	155.00	28.30	3.00	1.00	3.00	2.00	0.00	1.00	0.00	1.00	6.00	8.57	1.00	29.00	0.00	15.00	0.00	3.00	12.00	1.00
1267.00	43.00	90.00	174.00	29.73	3.00	1.00	3.00	2.00	0.00	1.00	1.00	1.00	5.00	5.71	1.00	28.00	0.00	2.00	1.00	10.00	40.00	1.00
1268.00	36.00	83.00	175.00	27.10	3.00	1.00	3.00	2.00	1.00	1.00	1.00	2.00	5.50	5.14	1.00	20.00	0.00	6.00	0.00	12.00	48.00	1.00
1269.00	40.00	90.00	174.00	29.73	3.00	1.00	3.00	2.00	1.00	1.00	1.00	2.00	2.50	22.86	1.00	32.00	1.00	6.00	0.00	13.00	52.00	0.00
1270.00	39.00	84.00	168.00	29.76	3.00	1.00	3.00	2.00	0.00	1.00	1.00	2.00	6.50	47.14	1.00	29.00	0.00	4.00	1.00	21.00	84.00	0.00
1271.00	44.00	86.00	181.00	26.25	3.00	1.00	3.00	3.00	1.00	1.00	1.00	2.00	3.00	47.14	1.00	33.00	1.00	3.00	1.00	23.00	92.00	0.00
1272.00	40.00	80.00	178.00	25.25	3.00	1.00	3.00	2.00	0.00	1.00	1.00	3.00	3.00	8.57	1.00	32.00	1.00	8.00	0.00	16.00	64.00	0.00
1273.00	43.00	76.00	163.00	28.60	3.00	1.00	3.00	1.00	0.00	1.00	0.00	3.00	7.00	20.00	1.00	26.00	0.00	1.00	1.00	14.00	56.00	0.00
1274.00	39.00	82.00	169.00	28.71	3.00	1.00	3.00	2.00	0.00	1.00	1.00	3.00	7.00	24.29	1.00	30.00	1.00	4.00	1.00	15.00	60.00	0.00
1275.00	39.00	94.00	182.00	28.38	3.00	1.00	3.00	2.00	0.00	1.00	1.00	3.00	7.00	17.14	1.00	29.00	0.00	5.00	1.00	17.00	68.00	0.00
1276.00	39.00	54.00	157.00	21.91	2.00	1.00	3.00	2.00	0.00	0.00	0.00	2.00	5.00	21.43	1.00	30.00	1.00	4.00	1.00	11.00	44.00	1.00
1277.00	38.00	55.00	153.00	23.50	2.00	1.00	3.00	2.00	0.00	0.00	0.00	2.00	6.50	0.71	1.00	35.00	1.00	1.00	1.00	15.00	60.00	0.00
1278.00	39.00	70.00	174.00	23.12	2.00	1.00	3.00	2.00	0.00	0.00	1.00	3.00	5.00	17.14	1.00	29.00	0.00	4.00	1.00	10.00	40.00	1.00
1279.00	38.00	67.00	178.00	21.15	2.00	1.00	3.00	2.00	0.00	0.00	1.00	3.00	8.00	51.43	1.00	23.00	0.00	7.00	0.00	8.00	32.00	1.00
1280.00	38.00	65.00	165.00	23.88	2.00	1.00	3.00	2.00	0.00	0.00	0.00	3.00	7.00	10.71	1.00	18.00	0.00	4.00	1.00	15.00	60.00	0.00
1281.00	34.00	67.00	165.00	24.61	2.00	1.00	3.00	2.00	0.00	0.00	0.00	3.00	5.00	34.29	1.00	37.00	1.00	4.00	1.00	14.00	56.00	0.00
1282.00	43.00	50.00	156.00	20.55	2.00	1.00	3.00	2.00	0.00	0.00	0.00	3.00	4.00	8.57	1.00	29.00	0.00	5.00	1.00	14.00	56.00	0.00
1283.00	39.00	70.00	169.00	24.51	2.00	1.00	3.00	2.00	0.00	0.00	0.00	3.00	7.00	34.29	1.00	28.00	0.00	5.00	1.00	15.00	60.00	0.00
1284.00	38.00	60.00	165.00	22.04	2.00	1.00	3.00	2.00	0.00	0.00	0.00	3.00	7.00	5.71	1.00	30.00	1.00	6.00	0.00	15.00	60.00	0.00
1285.00	35.00	58.00	158.00	23.23	2.00	1.00	3.00	1.00	0.00	0.00	0.00	3.00	6.00	12.86	1.00	35.00	1.00	0.00	1.00	16.00	64.00	0.00
1286.00	44.00	67.00	180.00	20.68	2.00	1.00	3.00	1.00	0.00	0.00	0.00	3.00	8.00	40.00	1.00	29.00	0.00	1.00	1.00	16.00	64.00	0.00
1287.00	36.00	62.00	167.00	22.23	2.00	1.00	3.00	2.00	0.00	0.00	1.00	3.00	8.00	50.00	1.00	34.00	1.00	4.00	1.00	13.00	52.00	0.00
1288.00	43.00	74.00	174.00	24.44	2.00	1.00	3.00	2.00	1.00	0.00	1.00	3.00	2.50	19.29	1.00	29.00	0.00	3.00	1.00	13.00	52.00	0.00
1289.00	40.00	75.00	174.00	24.77	2.00	1.00	3.00	2.00	0.00	0.00	1.00	3.00	4.00	28.57	1.00	29.00	0.00	4.00	1.00	16.00	64.00	0.00
1290.00	37.00	79.00	185.00	23.08	2.00	1.00	3.00	2.00	0.00	0.00	1.00	3.00	9.00	27.86	1.00	31.00	1.00	2.00	1.00	16.00	64.00	0.00
1291.00	40.00	90.00	190.00	24.93	2.00	0.00	3.00	1.00	0.00	0.00	1.00	3.00	4.00	21.43	1.00	32.00	1.00	5.00	1.00	16.00	64.00	0.00
1292.00	35.00	61.00	160.00	23.83	2.00	1.00	3.00	2.00	0.00	1.00	0.00	1.00	8.00	0.00	1.00	35.00	1.00	6.00	0.00	3.00	12.00	1.00
1293.00	41.00	74.00	181.00	22.59	2.00	1.00	3.00	2.00	0.00	1.00	1.00	2.00	5.00	20.00	1.00	29.00	0.00	11.00	0.00	11.00	44.00	1.00
1294.00	42.00	72.00	187.00	20.59	2.00	1.00	3.00	2.00	0.00	1.00	1.00	2.00	4.00	53.71	1.00	27.00	0.00	14.00	0.00	12.00	48.00	1.00
1295.00	40.00	78.00	178.00	24.62	2.00	0.00	3.00	2.00	1.00	1.00	1.00	2.00	8.00	30.00	1.00	31.00	1.00	5.00	1.00	19.00	76.00	0.00
1296.00	38.00	74.00	178.00	23.36	2.00	1.00	3.00	2.00	1.00	1.00	1.00	2.00	8.00	34.29	1.00	30.00	1.00	6.00	0.00	15.00	60.00	0.00
1297.00	40.00	58.00	168.00	20.55	2.00	1.00	3.00	2.00	0.00	1.00	0.00	3.00	5.00	42.86	1.00	34.00	1.00	2.00	1.00	16.00	64.00	0.00
1298.00	40.00	86.00	159.00	34.02	4.00	1.00	3.00	2.00	0.00	0.00	0.00	3.00	2.50	4.29	1.00	33.00	1.00	11.00	0.00	9.00	36.00	1.00
1299.00	37.00	85.00	175.00	27.76	3.00	1.00	3.00	2.00	0.00	0.00	1.00	2.00	6.00	17.14	1.00	31.00	1.00	1.00	1.00	13.00	52.00	0.00
1300.00	41.00	97.00	185.00	28.34	3.00	1.00	3.00	2.00	0.00	0.00	1.00	3.00	7.00	4.29	1.00	34.00	1.00	2.00	1.00	14.00	56.00	0.00
1301.00	34.00	60.00	162.00	22.86	2.00	1.00	3.00	2.00	0.00	0.00	0.00	3.00	9.00	60.00	1.00	37.00	1.00	4.00	1.00	15.00	60.00	0.00
1302.00	42.00	82.00	165.00	30.12	4.00	1.00	2.00	2.00	0.00	0.00	0.00	3.00	6.50	47.14	1.00	30.00	1.00	3.00	1.00	13.00	52.00	0.00
1303.00	35.00	93.00	170.00	32.18	4.00	0.00	2.00	2.00	0.00	0.00	0.00	3.00	3.00	15.71	1.00	34.00	1.00	5.00	1.00	19.00	76.00	0.00
1304.00	44.00	94.00	172.00	31.77	4.00	1.00	2.00	3.00	0.00	0.00	0.00	1.00	11.00	0.00	1.00	28.00	0.00	7.00	0.00	9.00	36.00	1.00
1305.00	35.00	92.00	175.00	30.04	4.00	0.00	2.00	2.00	0.00	0.00	0.00	2.00	10.00	12.86	1.00	16.00	0.00	14.00	0.00	7.00	28.00	1.00
1306.00	40.00	88.00	170.00	30.45	4.00	0.00	2.00	2.00	0.00	0.00	1.00	2.00	2.50	57.14	1.00	34.00	1.00	5.00	1.00	15.00	60.00	0.00
1307.00	42.00	75.00	156.00	30.82	4.00	1.00	2.00	2.00	0.00	0.00	0.00	3.00	6.00	11.43	1.00	33.00	1.00	8.00	0.00	6.00	24.00	1.00
1308.00	35.00	74.00	157.00	30.02	4.00	0.00	2.00	1.00	0.00	0.00	0.00	3.00	4.00	4.29	1.00	27.00	0.00	4.00	1.00	13.00	52.00	0.00
1309.00	35.00	105.00	157.00	42.60	4.00	1.00	2.00	1.00	0.00	0.00	0.00	3.00	4.00	4.29	1.00	27.00	0.00	4.00	1.00	13.00	52.00	0.00
1310.00	39.00	90.00	160.00	35.16	4.00	1.00	2.00	2.00	0.00	0.00	0.00	3.00	9.00	0.00	1.00	27.00	0.00	2.00	1.00	16.00	64.00	0.00
1311.00	40.00	90.00	165.00	33.06	4.00	0.00	2.00	2.00	0.00	0.00	0.00	3.00	2.00	0.00	1.00	31.00	1.00	2.00	1.00	18.00	72.00	0.00
1312.00	35.00	100.00	165.00	36.73	4.00	1.00	2.00	2.00	0.00	0.00	1.00	3.00	6.00	8.57	1.00	32.00	1.00	6.00	0.00	15.00	60.00	0.00
1313.00	44.00	80.00	160.00	31.25	4.00	1.00	2.00	2.00	0.00	0.00	0.00	3.00	4.00	2.86	1.00	30.00	1.00	2.00	1.00	18.00	72.00	0.00
1314.00	34.00	83.00	165.00	30.49	4.00	1.00	2.00	2.00	0.00	0.00	0.00	3.00	10.00	18.57	1.00	28.00	0.00	0.00	1.00	17.00	68.00	0.00
1315.00	43.00	80.00	160.00	31.25	4.00	0.00	2.00	2.00	0.00	0.00	0.00	3.00	5.50	0.00	1.00	12.00	0.00	1.00	1.00	13.00	52.00	0.00
1316.00	41.00	95.00	165.00	34.89	4.00	0.00	2.00	2.00	1.00	1.00	1.00	1.00	3.00	12.86	1.00	28.00	0.00	7.00	0.00	16.00	64.00	0.00
1317.00	38.00	115.00	183.00	34.34	4.00	1.00	2.00	2.00	0.00	1.00	1.00	1.00	4.50	0.00	1.00	33.00	1.00	4.00	1.00	19.00	76.00	0.00
1318.00	39.00	85.00	164.00	31.60	4.00	1.00	2.00	2.00	1.00	1.00	1.00	1.00	8.00	8.57	1.00	20.00	0.00	5.00	1.00	18.00	72.00	0.00
1319.00	40.00	99.00	173.00	33.08	4.00	1.00	2.00	2.00	1.00	1.00	1.00	2.00	6.00	5.00	1.00	28.00	0.00	12.00	0.00	12.00	48.00	1.00
1320.00	41.00	97.00	170.00	33.56	4.00	1.00	2.00	2.00	1.00	1.00	1.00	2.00	11.00	20.00	1.00	22.00	0.00	6.00	0.00	12.00	48.00	1.00

Subject	Age	Weight	Height	BMI	BMI_C	Residence	Education	Marital_Status	Smoking	Chronic_Dise	Gender	Health	Sitting_Time	PA_Min_Day	IPAQ_C	Dietary_Score	Dietary_C	PSQI_Total	Sleep_C	WHO5_Total	WHO5_Total100	WHO5_Class
**1321.00**	39.00	108.00	174.00	35.67	4.00	1.00	2.00	2.00	0.00	1.00	1.00	3.00	6.00	15.00	1.00	27.00	0.00	8.00	0.00	15.00	60.00	0.00
**1322.00**	44.00	107.00	180.00	33.02	4.00	0.00	3.00	2.00	0.00	0.00	1.00	1.00	6.00	0.00	1.00	35.00	1.00	7.00	0.00	13.00	52.00	0.00
**1323.00**	43.00	90.00	162.00	34.29	4.00	1.00	3.00	2.00	0.00	1.00	0.00	1.00	7.00	0.00	1.00	35.00	1.00	0.00	1.00	14.00	56.00	0.00
**1324.00**	42.00	100.00	175.00	32.65	4.00	1.00	3.00	2.00	1.00	1.00	1.00	2.00	8.00	17.14	1.00	25.00	0.00	7.00	0.00	12.00	48.00	1.00
**1325.00**	40.00	83.00	170.00	28.72	3.00	1.00	2.00	2.00	1.00	0.00	0.00	2.00	1.50	3.57	1.00	22.00	0.00	10.00	0.00	7.00	28.00	1.00
**1326.00**	40.00	95.00	180.00	29.32	3.00	1.00	2.00	2.00	0.00	0.00	1.00	2.00	6.00	25.71	1.00	29.00	0.00	5.00	1.00	11.00	44.00	1.00
**1327.00**	39.00	67.00	158.00	26.84	3.00	1.00	2.00	2.00	0.00	0.00	0.00	2.00	2.00	0.00	1.00	29.00	0.00	7.00	0.00	12.00	48.00	1.00
**1328.00**	37.00	62.00	150.00	27.56	3.00	1.00	2.00	2.00	1.00	0.00	0.00	2.00	6.50	0.00	1.00	20.00	0.00	5.00	1.00	13.00	52.00	0.00
**1329.00**	40.00	70.00	156.00	28.76	3.00	1.00	2.00	2.00	0.00	0.00	0.00	3.00	1.50	2.14	1.00	20.00	0.00	9.00	0.00	13.00	52.00	0.00
**1330.00**	35.00	70.00	165.00	25.71	3.00	1.00	2.00	2.00	0.00	0.00	0.00	3.00	8.00	37.14	1.00	31.00	1.00	5.00	1.00	15.00	60.00	0.00
**1331.00**	42.00	82.00	181.00	25.03	3.00	1.00	2.00	2.00	0.00	0.00	1.00	3.00	9.00	17.14	1.00	22.00	0.00	4.00	1.00	15.00	60.00	0.00
**1332.00**	37.00	80.00	165.00	29.38	3.00	1.00	2.00	2.00	0.00	0.00	0.00	3.00	9.00	12.86	1.00	23.00	0.00	6.00	0.00	9.00	36.00	1.00
**1333.00**	41.00	70.00	153.00	29.90	3.00	1.00	2.00	2.00	0.00	0.00	0.00	3.00	3.50	17.14	1.00	33.00	1.00	3.00	1.00	13.00	52.00	0.00
**1334.00**	40.00	64.00	150.00	28.44	3.00	1.00	2.00	2.00	0.00	0.00	0.00	3.00	5.50	2.14	1.00	31.00	1.00	6.00	0.00	9.00	36.00	1.00
**1335.00**	44.00	76.00	170.00	26.30	3.00	1.00	2.00	3.00	0.00	0.00	1.00	3.00	6.00	17.14	1.00	25.00	0.00	6.00	0.00	16.00	64.00	0.00
**1336.00**	35.00	79.00	173.00	26.40	3.00	1.00	2.00	2.00	0.00	0.00	1.00	3.00	7.00	18.57	1.00	32.00	1.00	4.00	1.00	13.00	52.00	0.00
**1337.00**	40.00	70.00	165.00	25.71	3.00	1.00	2.00	2.00	0.00	0.00	0.00	3.00	7.00	8.57	1.00	30.00	1.00	4.00	1.00	12.00	48.00	1.00
**1338.00**	34.00	70.00	164.00	26.03	3.00	1.00	2.00	2.00	0.00	0.00	0.00	3.00	5.50	31.43	1.00	30.00	1.00	3.00	1.00	15.00	60.00	0.00
**1339.00**	39.00	82.00	174.00	27.08	3.00	0.00	2.00	2.00	0.00	0.00	1.00	3.00	5.00	11.43	1.00	20.00	0.00	3.00	1.00	13.00	52.00	0.00
**1340.00**	42.00	90.00	180.00	27.78	3.00	1.00	2.00	2.00	1.00	1.00	1.00	1.00	4.00	34.29	1.00	27.00	0.00	7.00	0.00	8.00	32.00	1.00
**1341.00**	34.00	77.00	165.00	28.28	3.00	1.00	2.00	2.00	0.00	1.00	0.00	2.00	9.00	0.00	1.00	29.00	0.00	6.00	0.00	4.00	16.00	1.00
**1342.00**	37.00	85.00	180.00	26.23	3.00	1.00	2.00	1.00	0.00	1.00	1.00	3.00	7.00	0.00	1.00	33.00	1.00	9.00	0.00	10.00	40.00	1.00
**1343.00**	39.00	84.00	170.00	29.07	3.00	0.00	2.00	2.00	0.00	1.00	1.00	3.00	2.50	17.14	1.00	34.00	1.00	5.00	1.00	21.00	84.00	0.00
**1344.00**	39.00	80.00	167.00	28.69	3.00	0.00	3.00	3.00	1.00	0.00	1.00	1.00	9.00	36.43	1.00	35.00	1.00	8.00	0.00	9.00	36.00	1.00
**1345.00**	39.00	73.00	161.00	28.16	3.00	1.00	3.00	2.00	0.00	0.00	0.00	2.00	5.00	4.29	1.00	31.00	1.00	11.00	0.00	12.00	48.00	1.00
**1346.00**	40.00	90.00	180.00	27.78	3.00	1.00	3.00	2.00	0.00	0.00	1.00	2.00	4.00	41.43	1.00	38.00	1.00	3.00	1.00	14.00	56.00	0.00
**1347.00**	38.00	68.00	162.00	25.91	3.00	1.00	3.00	1.00	0.00	0.00	0.00	2.00	6.00	55.71	1.00	35.00	1.00	1.00	1.00	15.00	60.00	0.00
**1348.00**	35.00	70.00	165.00	25.71	3.00	1.00	3.00	1.00	0.00	0.00	0.00	3.00	5.00	17.14	1.00	37.00	1.00	4.00	1.00	7.00	28.00	1.00
**1349.00**	43.00	74.00	160.00	28.91	3.00	1.00	3.00	3.00	0.00	0.00	0.00	3.00	11.00	8.57	1.00	23.00	0.00	2.00	1.00	13.00	52.00	0.00
**1350.00**	43.00	73.00	168.00	25.86	3.00	1.00	3.00	2.00	0.00	1.00	1.00	3.00	8.00	17.14	1.00	28.00	0.00	4.00	1.00	18.00	72.00	0.00
**1351.00**	35.00	83.00	170.00	28.72	3.00	0.00	3.00	2.00	0.00	1.00	1.00	3.00	4.50	15.00	1.00	33.00	1.00	5.00	1.00	19.00	76.00	0.00
**1352.00**	40.00	51.00	158.00	20.43	2.00	0.00	1.00	2.00	1.00	0.00	0.00	2.00	3.00	24.29	1.00	27.00	0.00	0.00	1.00	17.00	68.00	0.00
**1353.00**	38.00	53.00	163.00	19.95	2.00	1.00	2.00	2.00	0.00	0.00	0.00	3.00	7.00	11.43	1.00	31.00	1.00	13.00	0.00	11.00	44.00	1.00
**1354.00**	39.00	58.00	163.00	21.83	2.00	0.00	2.00	2.00	0.00	0.00	0.00	1.00	8.00	0.00	1.00	26.00	0.00	2.00	1.00	9.00	36.00	1.00
**1355.00**	36.00	58.00	158.00	23.23	2.00	0.00	2.00	1.00	1.00	0.00	0.00	2.00	8.00	21.43	1.00	27.00	0.00	6.00	0.00	7.00	28.00	1.00
**1356.00**	40.00	70.00	171.00	23.94	2.00	1.00	2.00	2.00	0.00	0.00	0.00	2.00	8.00	57.29	1.00	25.00	0.00	9.00	0.00	12.00	48.00	1.00
**1357.00**	43.00	80.00	182.00	24.15	2.00	1.00	2.00	2.00	0.00	0.00	1.00	3.00	6.00	47.14	1.00	20.00	0.00	7.00	0.00	12.00	48.00	1.00
**1358.00**	36.00	62.00	160.00	24.22	2.00	1.00	2.00	3.00	1.00	0.00	1.00	3.00	7.00	34.29	1.00	33.00	1.00	2.00	1.00	16.00	64.00	0.00
**1359.00**	37.00	87.00	187.00	24.88	2.00	1.00	2.00	2.00	0.00	0.00	1.00	3.00	3.00	22.86	1.00	40.00	1.00	6.00	0.00	15.00	60.00	0.00
**1360.00**	38.00	68.00	175.00	22.20	2.00	1.00	2.00	2.00	0.00	0.00	0.00	3.00	1.50	17.14	1.00	40.00	1.00	3.00	1.00	15.00	60.00	0.00
**1361.00**	42.00	77.00	178.00	24.30	2.00	1.00	2.00	2.00	1.00	0.00	1.00	3.00	5.00	64.29	1.00	32.00	1.00	3.00	1.00	13.00	52.00	0.00
**1362.00**	34.00	60.00	165.00	22.04	2.00	1.00	2.00	2.00	0.00	0.00	0.00	3.00	6.00	0.00	1.00	21.00	0.00	7.00	0.00	17.00	68.00	0.00
**1363.00**	36.00	56.00	158.00	22.43	2.00	0.00	2.00	2.00	0.00	1.00	0.00	1.00	3.00	12.86	1.00	30.00	1.00	8.00	0.00	12.00	48.00	1.00
**1364.00**	40.00	55.00	160.00	21.48	2.00	1.00	2.00	2.00	0.00	1.00	0.00	1.00	4.50	8.57	1.00	22.00	0.00	4.00	1.00	9.00	36.00	1.00
**1365.00**	37.00	65.00	169.00	22.76	2.00	1.00	2.00	2.00	1.00	1.00	0.00	3.00	4.00	21.43	1.00	28.00	0.00	9.00	0.00	10.00	40.00	1.00
**1366.00**	42.00	65.00	164.00	24.17	2.00	1.00	2.00	2.00	0.00	1.00	1.00	3.00	3.50	0.00	1.00	24.00	0.00	11.00	0.00	9.00	36.00	1.00
**1367.00**	42.00	92.00	192.00	24.96	2.00	1.00	3.00	2.00	0.00	0.00	1.00	2.00	4.00	25.00	1.00	21.00	0.00	7.00	0.00	14.00	56.00	0.00
**1368.00**	39.00	58.00	160.00	22.66	2.00	1.00	3.00	2.00	0.00	0.00	0.00	3.00	6.00	15.71	1.00	24.00	0.00	3.00	1.00	13.00	52.00	0.00
**1369.00**	36.00	58.00	163.00	21.83	2.00	1.00	3.00	1.00	0.00	0.00	0.00	3.00	10.00	0.00	1.00	38.00	1.00	3.00	1.00	19.00	76.00	0.00
**1370.00**	40.00	66.00	165.00	24.24	2.00	0.00	3.00	3.00	1.00	0.00	0.00	3.00	11.00	5.71	1.00	37.00	1.00	4.00	1.00	20.00	80.00	0.00
**1371.00**	35.00	65.00	175.00	21.22	2.00	1.00	3.00	2.00	1.00	0.00	1.00	3.00	7.00	0.00	1.00	27.00	0.00	1.00	1.00	17.00	68.00	0.00
**1372.00**	39.00	62.00	163.00	23.34	2.00	0.00	3.00	2.00	0.00	0.00	0.00	3.00	3.00	53.57	1.00	28.00	0.00	3.00	1.00	15.00	60.00	0.00
**1373.00**	36.00	62.50	164.00	23.24	2.00	1.00	3.00	2.00	0.00	0.00	0.00	3.00	5.00	68.57	1.00	29.00	0.00	7.00	0.00	15.00	60.00	0.00
**1374.00**	42.00	70.00	170.00	24.22	2.00	1.00	3.00	2.00	0.00	1.00	0.00	3.00	5.00	37.14	1.00	33.00	1.00	4.00	1.00	15.00	60.00	0.00
**1375.00**	38.00	91.00	160.00	35.55	4.00	1.00	1.00	2.00	0.00	0.00	0.00	3.00	7.00	17.14	1.00	30.00	1.00	4.00	1.00	15.00	60.00	0.00
**1376.00**	41.00	63.00	155.00	26.22	3.00	1.00	1.00	2.00	0.00	0.00	0.00	2.00	4.00	20.00	1.00	33.00	1.00	5.00	1.00	11.00	44.00	1.00
**1377.00**	40.00	75.00	165.00	27.55	3.00	1.00	2.00	2.00	0.00	0.00	0.00	3.00	4.00	4.29	1.00	25.00	0.00	1.00	1.00	13.00	52.00	0.00
**1378.00**	34.00	64.00	180.00	19.75	2.00	0.00	2.00	1.00	0.00	0.00	1.00	3.00	10.00	36.43	1.00	24.00	0.00	7.00	0.00	9.00	36.00	1.00
**1379.00**	37.00	67.00	170.00	23.18	2.00	1.00	2.00	2.00	0.00	0.00	0.00	3.00	2.00	10.00	1.00	34.00	1.00	3.00	1.00	15.00	60.00	0.00
**1380.00**	55.00	106.00	178.00	33.46	4.00	1.00	2.00	2.00	0.00	0.00	1.00	3.00	10.00	27.14	1.00	23.00	0.00	12.00	0.00	12.00	48.00	1.00
**1381.00**	56.00	101.00	183.00	30.16	4.00	1.00	2.00	2.00	0.00	0.00	1.00	3.00	7.00	27.14	1.00	34.00	1.00	4.00	1.00	10.00	40.00	1.00
**1382.00**	47.00	95.00	175.00	31.02	4.00	1.00	3.00	2.00	0.00	0.00	1.00	2.00	9.00	68.57	1.00	39.00	1.00	2.00	1.00	20.00	80.00	0.00
**1383.00**	69.00	89.00	171.00	30.44	4.00	1.00	3.00	2.00	0.00	1.00	1.00	3.00	2.50	28.57	1.00	41.00	1.00	3.00	1.00	20.00	80.00	0.00
**1384.00**	47.00	87.00	176.00	28.09	3.00	1.00	2.00	2.00	0.00	1.00	1.00	3.00	4.00	30.00	1.00	23.00	0.00	8.00	0.00	10.00	40.00	1.00
**1385.00**	50.00	72.00	164.00	26.77	3.00	1.00	2.00	2.00	1.00	0.00	1.00	2.00	11.00	47.14	1.00	27.00	0.00	5.00	1.00	12.00	48.00	1.00
**1386.00**	50.00	76.00	167.00	27.25	3.00	1.00	2.00	2.00	0.00	0.00	0.00	3.00	5.00	62.86	1.00	36.00	1.00	6.00	0.00	9.00	36.00	1.00

Subject	Age	Weight	Height	BMI	BMI_C	Residence	Education	Marital_Status	Smoking	Chronic_Dise	Gender	Health	Sitting_Time	PA_Min_Day	IPAQ_C	Dietary_Score	Dietary_C	PSQI_Total	Sleep_C	WHO5_Total	WHO5_Total100	WHO5_Class
1387.00	47.00	74.00	166.00	26.85	3.00	1.00	2.00	2.00	0.00	0.00	0.00	3.00	11.00	0.00	1.00	23.00	0.00	3.00	1.00	16.00	64.00	0.00
1388.00	52.00	76.00	172.00	25.69	3.00	1.00	2.00	2.00	0.00	0.00	1.00	3.00	5.00	23.57	1.00	30.00	1.00	4.00	1.00	18.00	72.00	0.00
1389.00	58.00	86.00	180.00	26.54	3.00	1.00	2.00	2.00	0.00	0.00	1.00	3.00	5.50	18.57	1.00	28.00	0.00	3.00	1.00	19.00	76.00	0.00
1390.00	50.00	90.00	175.00	29.39	3.00	1.00	2.00	2.00	0.00	0.00	0.00	3.00	8.00	25.71	1.00	36.00	1.00	4.00	1.00	13.00	52.00	0.00
1391.00	58.00	85.00	172.00	28.73	3.00	1.00	3.00	2.00	1.00	1.00	1.00	2.00	6.00	11.43	1.00	30.00	1.00	9.00	0.00	9.00	36.00	1.00
1392.00	50.00	100.00	185.00	29.22	3.00	1.00	3.00	2.00	1.00	1.00	1.00	2.00	8.00	42.86	1.00	31.00	1.00	4.00	1.00	12.00	48.00	1.00
1393.00	48.00	69.00	171.00	23.60	2.00	1.00	3.00	3.00	1.00	0.00	1.00	3.00	10.00	28.57	1.00	18.00	0.00	7.00	0.00	15.00	60.00	0.00
1394.00	45.00	87.00	168.00	30.82	4.00	1.00	1.00	2.00	0.00	1.00	1.00	1.00	9.00	6.43	1.00	38.00	1.00	8.00	0.00	9.00	36.00	1.00
1395.00	56.00	90.00	173.00	30.07	4.00	1.00	2.00	2.00	0.00	1.00	1.00	1.00	7.00	51.43	1.00	25.00	0.00	8.00	0.00	9.00	36.00	1.00
1396.00	53.00	106.00	183.00	31.65	4.00	1.00	2.00	2.00	0.00	0.00	1.00	3.00	5.00	12.86	1.00	29.00	0.00	9.00	0.00	9.00	36.00	1.00
1397.00	45.00	120.00	183.00	35.83	4.00	1.00	2.00	2.00	0.00	0.00	1.00	3.00	9.00	0.00	1.00	21.00	0.00	5.00	1.00	10.00	40.00	1.00
1398.00	55.00	108.00	186.00	31.22	4.00	1.00	2.00	2.00	1.00	0.00	1.00	3.00	6.00	42.86	1.00	35.00	1.00	5.00	1.00	16.00	64.00	0.00
1399.00	45.00	100.00	176.00	32.28	4.00	1.00	2.00	2.00	0.00	1.00	1.00	1.00	7.00	0.00	1.00	26.00	0.00	5.00	1.00	17.00	68.00	0.00
1400.00	50.00	120.00	177.00	38.30	4.00	1.00	2.00	2.00	0.00	1.00	0.00	2.00	4.50	51.43	1.00	40.00	1.00	6.00	0.00	12.00	48.00	1.00
1401.00	48.00	117.00	180.00	36.11	4.00	1.00	3.00	2.00	0.00	0.00	1.00	3.00	9.00	0.00	1.00	32.00	1.00	4.00	1.00	12.00	48.00	1.00
1402.00	45.00	103.00	182.00	31.10	4.00	1.00	3.00	2.00	1.00	1.00	1.00	2.00	9.00	25.71	1.00	32.00	1.00	6.00	0.00	16.00	64.00	0.00
1403.00	49.00	68.00	162.00	25.91	3.00	1.00	2.00	2.00	0.00	0.00	0.00	3.00	8.00	13.43	1.00	29.00	0.00	6.00	0.00	17.00	68.00	0.00
1404.00	45.00	91.00	181.00	27.78	3.00	1.00	2.00	2.00	1.00	1.00	1.00	2.00	10.00	64.29	1.00	44.00	1.00	2.00	1.00	12.00	48.00	1.00
1405.00	56.00	66.00	159.00	26.11	3.00	1.00	2.00	3.00	1.00	0.00	0.00	3.00	11.00	0.00	1.00	19.00	0.00	9.00	0.00	8.00	32.00	1.00
1406.00	55.00	95.00	178.00	29.98	3.00	1.00	2.00	2.00	1.00	0.00	1.00	3.00	9.00	45.00	1.00	30.00	1.00	2.00	1.00	17.00	68.00	0.00
1407.00	49.00	89.00	180.00	27.47	3.00	1.00	2.00	2.00	1.00	0.00	1.00	3.00	11.00	8.57	1.00	26.00	0.00	3.00	1.00	13.00	52.00	0.00
1408.00	48.00	102.00	186.00	29.48	3.00	1.00	2.00	2.00	1.00	1.00	1.00	1.00	8.00	17.14	1.00	31.00	1.00	0.00	1.00	16.00	64.00	0.00
1409.00	45.00	105.00	192.00	28.48	3.00	1.00	2.00	2.00	1.00	1.00	1.00	2.00	7.00	17.14	1.00	29.00	0.00	8.00	0.00	9.00	36.00	1.00
1410.00	46.00	85.00	175.00	27.76	3.00	0.00	2.00	2.00	0.00	1.00	1.00	2.00	5.00	17.14	1.00	23.00	0.00	6.00	0.00	12.00	48.00	1.00
1411.00	49.00	85.00	170.00	29.41	3.00	1.00	2.00	2.00	1.00	1.00	1.00	2.00	7.00	5.00	1.00	24.00	0.00	5.00	1.00	13.00	52.00	0.00
1412.00	54.00	85.00	174.00	28.08	3.00	1.00	3.00	2.00	0.00	0.00	1.00	3.00	6.00	0.00	1.00	28.00	0.00	7.00	0.00	10.00	40.00	1.00
1413.00	45.00	77.00	167.00	27.61	3.00	1.00	3.00	2.00	0.00	0.00	1.00	3.00	3.00	17.14	1.00	32.00	1.00	0.00	1.00	17.00	68.00	0.00
1414.00	46.00	63.00	155.00	26.22	3.00	1.00	3.00	2.00	1.00	1.00	0.00	3.00	9.00	34.29	1.00	21.00	0.00	3.00	1.00	16.00	64.00	0.00
1415.00	50.00	75.00	183.00	22.40	2.00	1.00	1.00	2.00	1.00	1.00	1.00	1.00	4.00	51.43	1.00	27.00	0.00	8.00	0.00	8.00	32.00	1.00
1416.00	45.00	60.00	170.00	20.76	2.00	1.00	2.00	2.00	1.00	0.00	1.00	3.00	11.00	25.71	1.00	34.00	1.00	3.00	1.00	20.00	80.00	0.00
1417.00	62.00	52.00	158.00	20.83	2.00	1.00	2.00	2.00	0.00	0.00	0.00	2.00	9.00	17.14	1.00	32.00	1.00	9.00	0.00	12.00	48.00	1.00
1418.00	52.00	68.00	169.00	23.81	2.00	1.00	2.00	2.00	0.00	0.00	0.00	2.00	4.50	26.43	1.00	36.00	1.00	3.00	1.00	17.00	68.00	0.00
1419.00	49.00	71.00	170.00	24.57	2.00	1.00	2.00	2.00	0.00	0.00	1.00	3.00	2.00	35.71	1.00	33.00	1.00	3.00	1.00	15.00	60.00	0.00
1420.00	50.00	80.00	180.00	24.69	2.00	1.00	2.00	2.00	0.00	1.00	1.00	2.00	7.00	17.14	1.00	28.00	0.00	7.00	0.00	12.00	48.00	1.00
1421.00	57.00	67.00	169.00	23.46	2.00	1.00	2.00	2.00	0.00	1.00	1.00	2.00	4.00	51.43	1.00	35.00	1.00	9.00	0.00	15.00	60.00	0.00
1422.00	47.00	58.00	167.00	20.80	2.00	1.00	3.00	1.00	1.00	0.00	0.00	3.00	7.00	12.86	1.00	38.00	1.00	5.00	1.00	13.00	52.00	0.00
1423.00	50.00	75.00	180.00	23.15	2.00	1.00	3.00	2.00	1.00	0.00	1.00	3.00	7.00	65.71	1.00	35.00	1.00	2.00	1.00	16.00	64.00	0.00
1424.00	50.00	110.00	173.00	36.75	4.00	1.00	2.00	2.00	0.00	1.00	1.00	1.00	6.50	51.43	1.00	28.00	0.00	6.00	0.00	10.00	40.00	1.00
1425.00	45.00	70.00	152.00	30.30	4.00	1.00	2.00	2.00	0.00	1.00	0.00	2.00	5.00	19.29	1.00	32.00	1.00	6.00	0.00	11.00	44.00	1.00
1426.00	48.00	115.00	172.00	38.87	4.00	1.00	2.00	2.00	1.00	1.00	1.00	3.00	10.00	17.14	1.00	29.00	0.00	12.00	0.00	13.00	52.00	0.00
1427.00	50.00	95.00	180.00	29.32	3.00	1.00	2.00	2.00	0.00	0.00	1.00	3.00	5.00	8.57	1.00	29.00	0.00	2.00	1.00	15.00	60.00	0.00
1428.00	47.00	77.00	170.00	26.64	3.00	1.00	2.00	2.00	0.00	0.00	1.00	3.00	10.00	5.71	1.00	37.00	1.00	2.00	1.00	16.00	64.00	0.00
1429.00	72.00	75.00	172.00	25.35	3.00	1.00	2.00	2.00	0.00	1.00	1.00	2.00	4.50	19.29	1.00	30.00	1.00	7.00	0.00	12.00	48.00	1.00
1430.00	55.00	80.00	172.00	27.04	3.00	1.00	2.00	2.00	1.00	1.00	1.00	2.00	9.00	11.43	1.00	26.00	0.00	0.00	1.00	19.00	76.00	0.00
1431.00	52.00	78.00	171.00	26.67	3.00	1.00	3.00	2.00	1.00	1.00	1.00	2.00	9.00	0.00	1.00	28.00	0.00	6.00	0.00	17.00	68.00	0.00
1432.00	52.00	78.00	171.00	26.67	3.00	1.00	3.00	2.00	1.00	1.00	1.00	2.00	9.00	0.00	1.00	28.00	0.00	6.00	0.00	17.00	68.00	0.00
1433.00	47.00	67.00	173.00	22.39	2.00	1.00	2.00	2.00	0.00	0.00	0.00	3.00	4.00	27.86	1.00	38.00	1.00	4.00	1.00	18.00	72.00	0.00
1434.00	61.00	71.00	170.00	24.57	2.00	1.00	2.00	2.00	1.00	1.00	1.00	3.00	6.00	21.43	1.00	28.00	0.00	6.00	0.00	21.00	84.00	0.00
1435.00	54.00	62.00	175.00	20.24	2.00	1.00	3.00	2.00	0.00	0.00	1.00	3.00	7.00	38.57	1.00	36.00	1.00	3.00	1.00	14.00	56.00	0.00
1436.00	47.00	87.00	160.00	33.98	4.00	0.00	1.00	2.00	1.00	1.00	1.00	2.00	1.50	60.00	1.00	38.00	1.00	8.00	0.00	18.00	72.00	0.00
1437.00	50.00	100.00	181.00	30.52	4.00	1.00	2.00	1.00	1.00	0.00	1.00	3.00	7.00	60.00	1.00	35.00	1.00	3.00	1.00	13.00	52.00	0.00
1438.00	52.00	98.00	185.00	28.63	3.00	0.00	1.00	2.00	0.00	1.00	1.00	3.00	5.00	0.00	1.00	30.00	1.00	6.00	0.00	13.00	52.00	0.00
1439.00	52.00	98.00	185.00	28.63	3.00	0.00	1.00	2.00	0.00	1.00	1.00	3.00	5.00	0.00	1.00	30.00	1.00	6.00	0.00	13.00	52.00	0.00
1440.00	50.00	83.00	173.00	27.73	3.00	1.00	1.00	2.00	0.00	1.00	1.00	3.00	8.00	21.43	1.00	33.00	1.00	6.00	0.00	14.00	56.00	0.00
1441.00	60.00	85.00	170.00	29.41	3.00	1.00	1.00	2.00	0.00	0.00	1.00	3.00	6.00	21.43	1.00	36.00	1.00	1.00	1.00	17.00	68.00	0.00
1442.00	46.00	80.00	172.00	27.04	3.00	1.00	1.00	2.00	0.00	1.00	1.00	2.00	10.00	2.14	1.00	31.00	1.00	6.00	0.00	11.00	44.00	1.00
1443.00	55.00	72.00	165.00	26.45	3.00	1.00	2.00	2.00	0.00	0.00	0.00	3.00	7.00	51.43	1.00	31.00	1.00	7.00	0.00	12.00	48.00	1.00
1444.00	52.00	88.00	189.00	24.64	2.00	1.00	1.00	2.00	0.00	0.00	1.00	3.00	9.00	8.57	1.00	30.00	1.00	4.00	1.00	15.00	60.00	0.00
1445.00	47.00	81.00	167.00	29.04	3.00	1.00	2.00	2.00	0.00	1.00	1.00	2.00	1.00	53.57	1.00	33.00	1.00	8.00	0.00	14.00	56.00	0.00
1446.00	60.00	80.00	174.00	26.42	3.00	1.00	2.00	2.00	0.00	1.00	1.00	1.00	7.00	0.00	1.00	29.00	0.00	8.00	0.00	12.00	48.00	1.00
1447.00	47.00	79.00	168.00	27.99	3.00	1.00	2.00	3.00	1.00	1.00	1.00	1.00	9.00	17.86	1.00	34.00	1.00	3.00	1.00	14.00	56.00	0.00
1448.00	45.00	74.00	170.00	25.61	3.00	1.00	2.00	2.00	0.00	1.00	1.00	3.00	3.00	0.00	1.00	24.00	0.00	5.00	1.00	10.00	40.00	1.00
1449.00	47.00	86.00	173.00	28.73	3.00	1.00	3.00	2.00	0.00	1.00	1.00	3.00	2.50	11.43	1.00	33.00	1.00	8.00	0.00	15.00	60.00	0.00
1450.00	55.00	102.00	178.00	32.19	4.00	1.00	1.00	2.00	0.00	0.00	0.00	2.00	9.00	0.00	1.00	27.00	0.00	12.00	0.00	12.00	48.00	1.00
1451.00	53.00	82.00	163.00	30.86	4.00	1.00	1.00	2.00	0.00	0.00	0.00	3.00	1.00	55.00	1.00	32.00	1.00	8.00	0.00	14.00	56.00	0.00
1452.00	45.00	92.00	156.00	37.80	4.00	0.00	1.00	2.00	0.00	1.00	0.00	3.00	2.50	12.86	1.00	28.00	0.00	10.00	0.00	12.00	48.00	1.00

Subject	Age	Weight	Height	BMI	BMI_C	Residence	Education	Marital_Status	Smoking	Chronic_Dise	Gender	Health	Sitting_Time	PA_Min_Day	IPAQ_C	Dietary_Score	Dietary_C	PSQI_Total	Sleep_C	WHO5_Total	WHO5_Total100	WHO5_Class
1453.00	52.00	125.00	165.00	45.91	4.00	1.00	2.00	2.00	0.00	1.00	0.00	1.00	6.00	0.00	1.00	27.00	0.00	9.00	0.00	9.00	36.00	1.00
1454.00	55.00	102.00	178.00	32.19	4.00	1.00	2.00	2.00	1.00	1.00	0.00	2.00	6.00	0.00	1.00	25.00	0.00	13.00	0.00	9.00	36.00	1.00
1455.00	50.00	80.00	160.00	31.25	4.00	1.00	2.00	2.00	0.00	1.00	0.00	2.00	6.00	0.00	1.00	27.00	0.00	5.00	1.00	12.00	48.00	1.00
1456.00	53.00	80.00	168.00	28.34	3.00	1.00	2.00	2.00	0.00	1.00	0.00	2.00	10.00	21.43	1.00	37.00	1.00	3.00	1.00	14.00	56.00	0.00
1457.00	53.00	81.00	168.00	28.70	3.00	1.00	2.00	2.00	0.00	0.00	0.00	3.00	6.00	25.00	1.00	30.00	1.00	9.00	0.00	10.00	40.00	1.00
1458.00	48.00	65.00	158.00	26.04	3.00	1.00	2.00	2.00	0.00	0.00	0.00	3.00	2.00	4.29	1.00	29.00	0.00	1.00	1.00	17.00	68.00	0.00
1459.00	50.00	83.00	170.00	28.72	3.00	1.00	2.00	2.00	0.00	1.00	0.00	3.00	11.00	34.29	1.00	31.00	1.00	3.00	1.00	15.00	60.00	0.00
1460.00	54.00	78.00	167.00	27.97	3.00	1.00	2.00	2.00	0.00	1.00	0.00	3.00	5.00	8.57	1.00	29.00	0.00	9.00	0.00	16.00	64.00	0.00
1461.00	55.00	80.00	170.00	27.68	3.00	1.00	2.00	2.00	0.00	1.00	0.00	3.00	11.00	14.29	1.00	37.00	1.00	1.00	1.00	16.00	64.00	0.00
1462.00	63.00	63.00	163.00	23.71	2.00	1.00	1.00	2.00	0.00	1.00	0.00	1.00	2.00	0.00	1.00	28.00	0.00	8.00	0.00	10.00	40.00	1.00
1463.00	45.00	68.00	168.00	24.09	2.00	1.00	2.00	3.00	0.00	1.00	0.00	3.00	7.00	26.43	1.00	32.00	1.00	4.00	1.00	14.00	56.00	0.00
1464.00	50.00	68.00	165.00	24.98	2.00	1.00	2.00	2.00	0.00	0.00	0.00	3.00	5.00	0.00	1.00	26.00	0.00	3.00	1.00	18.00	72.00	0.00
1465.00	45.00	60.00	162.00	22.86	2.00	1.00	2.00	2.00	0.00	0.00	0.00	3.00	6.00	51.43	1.00	31.00	1.00	8.00	0.00	16.00	64.00	0.00
1466.00	50.00	54.00	153.00	23.07	2.00	1.00	2.00	2.00	0.00	0.00	0.00	3.00	1.00	34.29	1.00	38.00	1.00	3.00	1.00	18.00	72.00	0.00
1467.00	63.00	64.00	162.00	24.39	2.00	1.00	2.00	2.00	0.00	1.00	0.00	1.00	1.50	8.57	1.00	35.00	1.00	6.00	0.00	14.00	56.00	0.00
1468.00	53.00	55.00	160.00	21.48	2.00	1.00	2.00	3.00	1.00	1.00	0.00	2.00	7.00	2.86	1.00	29.00	0.00	5.00	1.00	17.00	68.00	0.00
1469.00	55.00	88.00	175.00	28.73	3.00	1.00	2.00	2.00	0.00	0.00	1.00	3.00	8.00	32.14	1.00	27.00	0.00	4.00	1.00	21.00	84.00	0.00
1470.00	46.00	77.00	176.00	24.86	2.00	1.00	2.00	2.00	1.00	1.00	1.00	2.00	5.00	42.86	1.00	25.00	0.00	6.00	0.00	15.00	60.00	0.00
1471.00	46.00	100.00	178.00	31.56	4.00	1.00	2.00	2.00	1.00	1.00	1.00	2.00	4.00	11.43	1.00	30.00	1.00	8.00	0.00	12.00	48.00	1.00
1472.00	52.00	118.00	179.00	36.83	4.00	1.00	2.00	2.00	0.00	0.00	1.00	3.00	2.00	27.14	1.00	30.00	1.00	2.00	1.00	19.00	76.00	0.00
1473.00	61.00	90.00	172.00	30.42	4.00	1.00	2.00	2.00	0.00	0.00	1.00	3.00	2.00	20.00	1.00	40.00	1.00	3.00	1.00	15.00	60.00	0.00
1474.00	46.00	110.00	173.00	36.75	4.00	1.00	3.00	2.00	0.00	1.00	1.00	1.00	6.00	34.29	1.00	31.00	1.00	2.00	1.00	17.00	68.00	0.00
1475.00	63.00	82.00	162.00	31.25	4.00	1.00	3.00	2.00	0.00	1.00	0.00	3.00	8.00	5.71	1.00	35.00	1.00	8.00	0.00	13.00	52.00	0.00
1476.00	75.00	112.00	175.00	36.57	4.00	1.00	3.00	2.00	0.00	1.00	1.00	3.00	5.00	0.00	1.00	38.00	1.00	4.00	1.00	14.00	56.00	0.00
1477.00	71.00	90.00	183.00	26.87	3.00	1.00	2.00	2.00	1.00	1.00	1.00	2.00	3.50	38.57	1.00	25.00	0.00	8.00	0.00	17.00	68.00	0.00
1478.00	70.00	75.00	173.00	25.06	3.00	1.00	2.00	2.00	1.00	1.00	1.00	2.00	4.00	10.71	1.00	37.00	1.00	5.00	1.00	16.00	64.00	0.00
1479.00	68.00	94.00	183.00	28.07	3.00	1.00	3.00	2.00	0.00	0.00	1.00	3.00	6.00	48.57	1.00	40.00	1.00	7.00	0.00	14.00	56.00	0.00
1480.00	70.00	83.00	170.00	28.72	3.00	1.00	3.00	2.00	1.00	1.00	1.00	2.00	4.00	0.00	1.00	32.00	1.00	9.00	0.00	12.00	48.00	1.00
1481.00	56.00	85.00	171.00	29.07	3.00	1.00	2.00	2.00	0.00	1.00	0.00	2.00	5.00	17.14	1.00	37.00	1.00	8.00	0.00	16.00	64.00	0.00
1482.00	56.00	118.00	178.00	37.24	4.00	1.00	1.00	2.00	1.00	0.00	1.00	2.00	3.00	0.00	1.00	24.00	0.00	6.00	0.00	15.00	60.00	0.00
1483.00	58.00	85.00	156.00	34.93	4.00	1.00	2.00	2.00	0.00	0.00	0.00	1.00	6.00	51.43	1.00	39.00	1.00	1.00	1.00	19.00	76.00	0.00
1484.00	52.00	100.00	168.00	35.43	4.00	1.00	2.00	1.00	0.00	0.00	0.00	3.00	6.50	0.00	1.00	35.00	1.00	7.00	0.00	10.00	40.00	1.00
1485.00	66.00	100.10	180.30	30.79	4.00	1.00	2.00	2.00	0.00	0.00	0.00	3.00	11.00	60.00	1.00	30.00	1.00	0.00	1.00	17.00	68.00	0.00
1486.00	56.00	95.00	174.00	31.38	4.00	1.00	2.00	2.00	0.00	0.00	1.00	3.00	6.00	13.43	1.00	26.00	0.00	10.00	0.00	17.00	68.00	0.00
1487.00	54.00	108.00	176.00	34.87	4.00	0.00	2.00	3.00	1.00	1.00	1.00	1.00	5.50	0.00	1.00	31.00	1.00	8.00	0.00	14.00	56.00	0.00
1488.00	65.00	90.00	173.00	30.07	4.00	1.00	2.00	2.00	0.00	1.00	0.00	2.00	4.00	0.00	1.00	36.00	1.00	12.00	0.00	7.00	28.00	1.00
1489.00	45.00	100.00	180.00	30.86	4.00	1.00	2.00	3.00	1.00	1.00	1.00	3.00	7.00	21.43	1.00	30.00	1.00	13.00	0.00	12.00	48.00	1.00
1490.00	45.00	113.00	186.00	32.66	4.00	1.00	2.00	2.00	1.00	1.00	1.00	3.00	10.00	0.00	1.00	32.00	1.00	8.00	0.00	5.00	20.00	1.00
1491.00	60.00	98.00	178.00	30.93	4.00	1.00	3.00	2.00	0.00	1.00	1.00	1.00	4.50	0.00	1.00	26.00	0.00	8.00	0.00	15.00	60.00	0.00
1492.00	61.00	77.00	160.00	30.08	4.00	1.00	3.00	2.00	0.00	1.00	0.00	2.00	3.50	0.00	1.00	34.00	1.00	6.00	0.00	12.00	48.00	1.00
1493.00	58.00	100.00	180.00	30.86	4.00	1.00	3.00	2.00	0.00	1.00	1.00	3.00	4.00	0.86	1.00	31.00	1.00	8.00	0.00	15.00	60.00	0.00
1494.00	47.00	80.00	172.00	27.04	3.00	1.00	1.00	2.00	1.00	0.00	1.00	2.00	8.00	38.57	1.00	26.00	0.00	5.00	1.00	13.00	52.00	0.00
1495.00	55.00	88.00	175.00	28.73	3.00	0.00	1.00	2.00	1.00	1.00	1.00	2.00	8.00	17.14	1.00	23.00	0.00	4.00	1.00	14.00	56.00	0.00
1496.00	50.00	75.00	160.00	29.30	3.00	1.00	2.00	2.00	0.00	0.00	0.00	2.00	6.50	1.00	1.00	22.00	0.00	10.00	0.00	11.00	44.00	1.00
1497.00	50.00	83.00	178.00	26.20	3.00	1.00	2.00	2.00	0.00	0.00	1.00	3.00	7.00	25.71	1.00	33.00	1.00	5.00	1.00	11.00	44.00	1.00
1498.00	57.00	95.00	181.00	29.00	3.00	1.00	2.00	2.00	1.00	0.00	1.00	1.00	7.00	2.86	1.00	24.00	0.00	7.00	0.00	9.00	36.00	1.00
1499.00	55.00	82.00	180.00	25.31	3.00	1.00	2.00	2.00	0.00	0.00	1.00	2.00	7.00	11.43	1.00	24.00	0.00	4.00	1.00	9.00	36.00	1.00
1500.00	57.00	75.00	160.00	29.30	3.00	1.00	2.00	2.00	0.00	0.00	0.00	2.00	5.00	0.00	1.00	35.00	1.00	4.00	1.00	13.00	52.00	0.00
1501.00	46.00	70.00	157.00	28.40	3.00	0.00	2.00	2.00	0.00	0.00	1.00	3.00	7.00	15.00	1.00	32.00	1.00	7.00	0.00	18.00	72.00	0.00
1502.00	55.00	87.00	180.00	26.85	3.00	1.00	2.00	2.00	0.00	1.00	1.00	1.00	8.00	17.14	1.00	39.00	1.00	9.00	0.00	2.00	8.00	1.00
1503.00	60.00	80.00	176.00	25.83	3.00	1.00	2.00	2.00	1.00	1.00	1.00	1.00	6.00	8.57	1.00	31.00	1.00	1.00	1.00	11.00	44.00	1.00
1504.00	57.00	85.00	175.00	27.76	3.00	1.00	2.00	2.00	1.00	1.00	1.00	2.00	10.00	8.57	1.00	32.00	1.00	7.00	0.00	11.00	44.00	1.00
1505.00	50.00	82.00	175.00	26.78	3.00	1.00	2.00	2.00	1.00	1.00	1.00	2.00	5.00	19.29	1.00	29.00	0.00	4.00	1.00	15.00	60.00	0.00
1506.00	57.00	85.00	175.00	27.76	3.00	1.00	2.00	2.00	1.00	1.00	1.00	2.00	9.00	8.57	1.00	32.00	1.00	6.00	0.00	12.00	48.00	1.00
1507.00	46.00	80.00	176.00	25.83	3.00	1.00	3.00	2.00	0.00	0.00	1.00	3.00	6.50	30.00	1.00	31.00	1.00	6.00	0.00	12.00	48.00	1.00
1508.00	48.00	86.00	170.00	29.76	3.00	1.00	3.00	2.00	0.00	0.00	1.00	3.00	5.00	34.29	1.00	31.00	1.00	12.00	0.00	12.00	48.00	1.00
1509.00	46.00	88.00	179.00	27.46	3.00	1.00	3.00	2.00	0.00	1.00	1.00	2.00	4.50	7.86	1.00	26.00	0.00	3.00	1.00	19.00	76.00	0.00
1510.00	45.00	72.00	164.00	26.77	3.00	1.00	3.00	2.00	0.00	1.00	1.00	2.00	5.00	12.86	1.00	30.00	1.00	5.00	1.00	14.00	56.00	0.00
1511.00	50.00	83.00	168.00	29.41	3.00	0.00	3.00	2.00	0.00	1.00	0.00	3.00	1.50	12.86	1.00	35.00	1.00	2.00	1.00	16.00	64.00	0.00
1512.00	65.00	79.00	165.00	29.02	3.00	1.00	3.00	2.00	0.00	1.00	1.00	3.00	4.00	38.57	1.00	31.00	1.00	4.00	1.00	19.00	76.00	0.00
1513.00	46.00	64.00	158.00	25.64	3.00	1.00	3.00	2.00	0.00	1.00	0.00	3.00	6.00	34.29	1.00	37.00	1.00	4.00	1.00	18.00	72.00	0.00
1514.00	50.00	77.00	170.00	26.64	3.00	1.00	3.00	2.00	1.00	1.00	1.00	3.00	7.00	18.57	1.00	28.00	0.00	5.00	1.00	15.00	60.00	0.00
1515.00	49.00	63.00	168.00	22.32	2.00	0.00	1.00	2.00	0.00	1.00	1.00	3.00	2.00	25.71	1.00	31.00	1.00	3.00	1.00	12.00	48.00	1.00
1516.00	47.00	75.00	176.00	24.21	2.00	1.00	2.00	2.00	1.00	1.00	1.00	1.00	8.00	22.14	1.00	19.00	0.00	7.00	0.00	6.00	24.00	1.00
1517.00	51.00	60.00	156.00	24.65	2.00	1.00	2.00	2.00	0.00	0.00	0.00	1.00	6.50	0.00	1.00	34.00	1.00	3.00	1.00	13.00	52.00	0.00
1518.00	45.00	60.00	159.00	23.73	2.00	1.00	2.00	2.00	0.00	0.00	0.00	3.00	8.00	0.00	1.00	29.00	0.00	5.00	1.00	13.00	52.00	0.00

Subject	Age	Weight	Height	BMI	BMI_C	Residence	Education	Marital_Status	Smoking	Chronic_Dise	Gender	Health	Sitting_Time	PA_Min_Day	IPAQ_C	Dietary_Score	Dietary_C	PSQI_Total	Sleep_C	WHO5_Total	WHO5_Total100	WHO5_Class
1519.00	45.00	63.00	165.00	23.14	2.00	1.00	2.00	2.00	0.00	0.00	1.00	3.00	10.00	11.43	1.00	35.00	1.00	7.00	0.00	14.00	56.00	0.00
1520.00	54.00	86.00	186.00	24.86	2.00	1.00	2.00	2.00	0.00	0.00	1.00	3.00	8.00	20.71	1.00	35.00	1.00	3.00	1.00	13.00	52.00	0.00
1521.00	52.00	75.00	174.00	24.77	2.00	1.00	2.00	3.00	0.00	0.00	1.00	3.00	6.00	57.14	1.00	29.00	0.00	1.00	1.00	16.00	64.00	0.00
1522.00	53.00	62.00	159.00	24.52	2.00	1.00	2.00	2.00	1.00	1.00	1.00	2.00	7.00	0.00	1.00	22.00	0.00	10.00	0.00	14.00	56.00	0.00
1523.00	49.00	76.00	170.00	26.30	3.00	0.00	3.00	2.00	0.00	0.00	0.00	3.00	3.00	25.71	1.00	30.00	1.00	0.00	1.00	21.00	84.00	0.00
1524.00	53.00	80.00	175.00	26.12	3.00	1.00	3.00	2.00	0.00	1.00	0.00	3.00	4.50	10.00	1.00	38.00	1.00	4.00	1.00	17.00	68.00	0.00
1525.00	47.00	77.00	186.00	22.26	2.00	1.00	2.00	2.00	0.00	0.00	0.00	2.00	2.00	0.00	1.00	31.00	1.00	5.00	1.00	9.00	36.00	1.00
1526.00	47.00	73.00	173.00	24.39	2.00	1.00	2.00	2.00	0.00	0.00	1.00	3.00	7.00	3.57	1.00	28.00	0.00	8.00	0.00	10.00	40.00	1.00
1527.00	58.00	105.00	180.00	32.41	4.00	1.00	2.00	2.00	1.00	0.00	1.00	3.00	5.50	2.14	1.00	24.00	0.00	5.00	1.00	10.00	40.00	1.00
1528.00	47.00	100.00	170.00	34.60	4.00	1.00	3.00	2.00	0.00	0.00	1.00	1.00	7.00	10.71	1.00	23.00	0.00	3.00	1.00	9.00	36.00	1.00
1529.00	51.00	102.00	171.00	34.88	4.00	1.00	3.00	2.00	0.00	0.00	0.00	1.00	9.00	21.43	1.00	41.00	1.00	0.00	1.00	19.00	76.00	0.00
1530.00	48.00	92.00	174.00	30.39	4.00	1.00	3.00	2.00	1.00	0.00	1.00	2.00	10.00	64.29	1.00	27.00	0.00	3.00	1.00	11.00	44.00	1.00
1531.00	57.00	79.00	158.00	31.65	4.00	1.00	3.00	3.00	0.00	0.00	0.00	2.00	9.00	8.57	1.00	29.00	0.00	12.00	0.00	11.00	44.00	1.00
1532.00	52.00	90.00	160.00	35.16	4.00	1.00	3.00	2.00	0.00	0.00	0.00	2.00	4.00	2.86	1.00	35.00	1.00	7.00	0.00	11.00	44.00	1.00
1533.00	55.00	100.00	180.00	30.86	4.00	1.00	3.00	2.00	0.00	0.00	1.00	2.00	6.00	0.00	1.00	31.00	1.00	1.00	1.00	15.00	60.00	0.00
1534.00	56.00	100.00	170.00	34.60	4.00	1.00	3.00	2.00	0.00	0.00	0.00	2.00	7.00	15.71	1.00	29.00	0.00	7.00	0.00	12.00	48.00	1.00
1535.00	50.00	109.00	177.00	34.79	4.00	1.00	3.00	2.00	0.00	0.00	1.00	2.00	7.00	4.29	1.00	31.00	1.00	3.00	1.00	13.00	52.00	0.00
1536.00	52.00	80.00	160.00	31.25	4.00	1.00	3.00	2.00	0.00	0.00	0.00	2.00	4.50	38.57	1.00	30.00	1.00	4.00	1.00	15.00	60.00	0.00
1537.00	51.00	89.00	169.00	31.16	4.00	1.00	3.00	2.00	0.00	0.00	1.00	3.00	7.00	11.43	1.00	22.00	0.00	4.00	1.00	11.00	44.00	1.00
1538.00	46.00	92.00	174.00	30.39	4.00	1.00	3.00	3.00	0.00	0.00	1.00	3.00	8.00	12.86	1.00	35.00	1.00	6.00	0.00	10.00	40.00	1.00
1539.00	50.00	83.00	162.00	31.63	4.00	1.00	3.00	2.00	0.00	0.00	0.00	3.00	6.00	17.14	1.00	31.00	1.00	5.00	1.00	13.00	52.00	0.00
1540.00	53.00	95.00	173.00	31.74	4.00	0.00	3.00	2.00	0.00	0.00	1.00	3.00	5.50	0.00	1.00	29.00	0.00	5.00	1.00	9.00	36.00	1.00
1541.00	47.00	112.00	183.00	33.44	4.00	1.00	3.00	2.00	0.00	0.00	1.00	3.00	5.00	34.29	1.00	37.00	1.00	2.00	1.00	16.00	64.00	0.00
1542.00	50.00	90.00	170.00	31.14	4.00	1.00	3.00	2.00	0.00	0.00	1.00	3.00	6.00	14.29	1.00	30.00	1.00	3.00	1.00	12.00	48.00	1.00
1543.00	45.00	130.00	193.00	34.90	4.00	1.00	3.00	2.00	0.00	0.00	1.00	3.00	4.00	55.71	1.00	33.00	1.00	0.00	1.00	13.00	52.00	0.00
1544.00	46.00	70.00	147.00	32.39	4.00	1.00	3.00	2.00	0.00	0.00	0.00	3.00	7.00	1.43	1.00	31.00	1.00	4.00	1.00	16.00	64.00	0.00
1545.00	66.00	89.00	168.00	31.53	4.00	1.00	3.00	2.00	0.00	0.00	1.00	3.00	6.00	12.86	1.00	29.00	0.00	3.00	1.00	23.00	92.00	0.00
1546.00	49.00	100.00	181.00	30.52	4.00	1.00	3.00	2.00	1.00	0.00	1.00	3.00	5.00	42.86	1.00	37.00	1.00	3.00	1.00	13.00	52.00	0.00
1547.00	54.00	89.00	170.00	30.80	4.00	1.00	3.00	2.00	0.00	0.00	1.00	3.00	8.00	12.86	1.00	37.00	1.00	4.00	1.00	14.00	56.00	0.00
1548.00	57.00	88.00	165.00	32.32	4.00	0.00	3.00	2.00	0.00	0.00	1.00	3.00	4.50	12.86	1.00	38.00	1.00	5.00	1.00	16.00	64.00	0.00
1549.00	60.00	85.00	160.00	33.20	4.00	1.00	3.00	2.00	0.00	0.00	0.00	3.00	9.00	0.00	1.00	31.00	1.00	6.00	0.00	15.00	60.00	0.00
1550.00	57.00	98.00	175.00	32.00	4.00	1.00	3.00	2.00	0.00	0.00	1.00	3.00	5.50	0.00	1.00	31.00	1.00	2.00	1.00	13.00	52.00	0.00
1551.00	56.00	104.00	173.00	34.75	4.00	1.00	3.00	2.00	1.00	1.00	1.00	1.00	11.00	22.86	1.00	26.00	0.00	9.00	0.00	6.00	24.00	1.00
1552.00	56.00	100.00	182.00	30.19	4.00	1.00	3.00	2.00	1.00	1.00	1.00	1.00	4.00	20.00	1.00	34.00	1.00	4.00	1.00	13.00	52.00	0.00
1553.00	49.00	85.00	155.00	35.38	4.00	1.00	3.00	2.00	0.00	1.00	0.00	1.00	11.00	4.29	1.00	26.00	0.00	5.00	1.00	17.00	68.00	0.00
1554.00	58.00	100.00	171.00	34.20	4.00	1.00	3.00	2.00	0.00	1.00	1.00	2.00	6.00	18.57	1.00	29.00	0.00	4.00	1.00	14.00	56.00	0.00
1555.00	45.00	107.00	176.00	34.54	4.00	1.00	3.00	2.00	0.00	1.00	1.00	2.00	6.50	2.14	1.00	30.00	1.00	7.00	0.00	12.00	48.00	1.00
1556.00	53.00	71.00	152.00	30.73	4.00	0.00	3.00	2.00	0.00	1.00	0.00	2.00	3.50	24.29	1.00	21.00	0.00	7.00	0.00	12.00	48.00	1.00
1557.00	54.00	84.00	167.00	30.12	4.00	1.00	3.00	2.00	1.00	1.00	1.00	2.00	2.00	0.00	1.00	28.00	0.00	7.00	0.00	12.00	48.00	1.00
1558.00	56.00	93.00	165.00	34.16	4.00	1.00	3.00	2.00	0.00	1.00	0.00	2.00	6.00	17.14	1.00	38.00	1.00	6.00	0.00	19.00	76.00	0.00
1559.00	47.00	115.00	191.00	31.52	4.00	1.00	3.00	2.00	0.00	1.00	1.00	2.00	8.00	0.00	1.00	32.00	1.00	6.00	0.00	14.00	56.00	0.00
1560.00	55.00	110.00	180.00	33.95	4.00	1.00	3.00	2.00	0.00	1.00	1.00	2.00	4.00	42.86	1.00	32.00	1.00	3.00	1.00	12.00	48.00	1.00
1561.00	46.00	87.00	165.00	31.96	4.00	1.00	3.00	2.00	1.00	1.00	1.00	2.00	9.00	8.57	1.00	34.00	1.00	3.00	1.00	16.00	64.00	0.00
1562.00	49.00	100.00	180.00	30.86	4.00	1.00	3.00	2.00	1.00	1.00	1.00	2.00	5.00	58.57	1.00	31.00	1.00	3.00	1.00	17.00	68.00	0.00
1563.00	54.00	100.00	168.00	35.43	4.00	1.00	3.00	2.00	1.00	1.00	1.00	3.00	11.00	0.00	1.00	32.00	1.00	6.00	0.00	12.00	48.00	1.00
1564.00	46.00	80.00	155.00	33.30	4.00	1.00	3.00	2.00	0.00	1.00	0.00	3.00	5.00	2.86	1.00	24.00	0.00	1.00	1.00	14.00	56.00	0.00
1565.00	45.00	80.00	160.00	31.25	4.00	1.00	3.00	2.00	1.00	1.00	0.00	3.00	9.00	25.71	1.00	33.00	1.00	4.00	1.00	16.00	64.00	0.00
1566.00	48.00	95.00	176.00	30.67	4.00	0.00	3.00	2.00	0.00	1.00	1.00	3.00	5.00	37.14	1.00	33.00	1.00	0.00	1.00	14.00	56.00	0.00
1567.00	56.00	95.00	178.00	29.98	3.00	1.00	2.00	2.00	0.00	0.00	1.00	3.00	9.00	5.71	1.00	30.00	1.00	5.00	1.00	13.00	52.00	0.00
1568.00	50.00	80.00	170.00	27.68	3.00	1.00	2.00	3.00	1.00	1.00	1.00	2.00	5.00	64.29	1.00	34.00	1.00	5.00	1.00	11.00	44.00	1.00
1569.00	47.00	68.00	158.00	27.24	3.00	1.00	3.00	2.00	0.00	0.00	0.00	1.00	5.00	1.43	1.00	23.00	0.00	2.00	1.00	14.00	56.00	0.00
1570.00	50.00	67.00	156.00	27.53	3.00	1.00	3.00	2.00	0.00	0.00	0.00	1.00	6.00	12.86	1.00	32.00	1.00	2.00	1.00	14.00	56.00	0.00
1571.00	45.00	67.00	162.00	25.53	3.00	1.00	3.00	2.00	0.00	0.00	0.00	2.00	4.00	34.29	1.00	21.00	0.00	3.00	1.00	12.00	48.00	1.00
1572.00	66.00	79.00	170.00	27.34	3.00	1.00	3.00	2.00	0.00	0.00	1.00	2.00	8.00	40.00	1.00	33.00	1.00	7.00	0.00	12.00	48.00	1.00
1573.00	60.00	83.00	172.00	28.06	3.00	1.00	3.00	2.00	0.00	0.00	1.00	2.00	5.00	4.29	1.00	26.00	0.00	4.00	1.00	19.00	76.00	0.00
1574.00	52.00	84.00	168.00	29.76	3.00	1.00	3.00	2.00	0.00	0.00	1.00	2.00	7.00	12.86	1.00	29.00	0.00	3.00	1.00	15.00	60.00	0.00
1575.00	48.00	64.00	150.00	28.44	3.00	1.00	3.00	2.00	0.00	0.00	0.00	2.00	1.50	0.00	1.00	35.00	1.00	4.00	1.00	16.00	64.00	0.00
1576.00	46.00	63.00	155.00	26.22	3.00	1.00	3.00	2.00	0.00	0.00	0.00	2.00	4.50	8.57	1.00	26.00	0.00	5.00	1.00	16.00	64.00	0.00
1577.00	50.00	65.00	160.00	25.39	3.00	1.00	3.00	2.00	0.00	0.00	0.00	2.00	5.50	0.00	1.00	37.00	1.00	2.00	1.00	15.00	60.00	0.00
1578.00	59.00	91.00	183.00	27.17	3.00	1.00	3.00	2.00	0.00	0.00	1.00	3.00	5.00	40.00	1.00	30.00	1.00	10.00	0.00	8.00	32.00	1.00
1579.00	46.00	80.00	165.00	29.38	3.00	1.00	3.00	2.00	0.00	0.00	0.00	3.00	7.00	0.00	1.00	25.00	0.00	10.00	0.00	10.00	40.00	1.00
1580.00	63.00	70.00	162.00	26.67	3.00	1.00	3.00	2.00	0.00	0.00	0.00	3.00	7.00	7.14	1.00	30.00	1.00	10.00	0.00	15.00	60.00	0.00
1581.00	50.00	87.00	180.00	26.85	3.00	0.00	3.00	2.00	0.00	0.00	1.00	3.00	10.00	21.43	1.00	27.00	0.00	6.00	0.00	5.00	20.00	1.00
1582.00	48.00	90.00	179.00	28.09	3.00	0.00	3.00	3.00	1.00	0.00	1.00	3.00	6.50	53.57	1.00	29.00	0.00	7.00	0.00	7.00	28.00	1.00
1583.00	56.00	84.00	173.00	28.07	3.00	1.00	3.00	2.00	0.00	0.00	1.00	3.00	10.00	13.57	1.00	27.00	0.00	7.00	0.00	13.00	52.00	0.00
1584.00	45.00	83.00	170.00	28.72	3.00	1.00	3.00	2.00	0.00	0.00	1.00	3.00	7.00	32.86	1.00	25.00	0.00	2.00	1.00	9.00	36.00	1.00

Subject	Age	Weight	Height	BMI	BMI_C	Residence	Education	Marital_Status	Smoking	Chronic_Dise	Gender	Health	Sitting_Time	PA_Min_Day	IPAQ_C	Dietary_Score	Dietary_C	PSQI_Total	Sleep_C	WHO5_Total	WHO5_Total100	WHO5_Class
1585.00	49.00	80.00	171.00	27.36	3.00	1.00	3.00	2.00	0.00	0.00	0.00	3.00	6.00	38.57	1.00	31.00	1.00	8.00	0.00	13.00	52.00	0.00
1586.00	53.00	77.00	167.00	27.61	3.00	1.00	3.00	2.00	0.00	0.00	0.00	3.00	5.00	47.86	1.00	30.00	1.00	5.00	1.00	9.00	36.00	1.00
1587.00	61.00	108.00	195.00	28.40	3.00	1.00	3.00	3.00	0.00	0.00	1.00	3.00	1.50	21.43	1.00	29.00	0.00	8.00	0.00	10.00	40.00	1.00
1588.00	50.00	69.00	162.00	26.29	3.00	1.00	3.00	3.00	0.00	0.00	0.00	3.00	6.00	12.86	1.00	28.00	0.00	10.00	0.00	10.00	40.00	1.00
1589.00	60.00	85.00	169.00	29.76	3.00	1.00	3.00	2.00	0.00	0.00	1.00	3.00	10.00	17.14	1.00	35.00	1.00	0.00	1.00	17.00	68.00	0.00
1590.00	65.00	86.00	179.00	26.84	3.00	1.00	3.00	2.00	0.00	0.00	1.00	3.00	3.00	22.86	1.00	23.00	0.00	2.00	1.00	18.00	72.00	0.00
1591.00	53.00	75.00	160.00	29.30	3.00	1.00	3.00	3.00	0.00	0.00	0.00	3.00	2.50	11.43	1.00	30.00	1.00	2.00	1.00	15.00	60.00	0.00
1592.00	49.00	100.00	188.00	28.29	3.00	1.00	3.00	2.00	0.00	0.00	1.00	3.00	7.00	15.00	1.00	30.00	1.00	6.00	0.00	15.00	60.00	0.00
1593.00	47.00	75.00	160.00	29.30	3.00	1.00	3.00	3.00	0.00	0.00	0.00	3.00	4.00	0.00	1.00	25.00	0.00	2.00	1.00	15.00	60.00	0.00
1594.00	55.00	86.00	174.00	28.41	3.00	1.00	3.00	2.00	0.00	0.00	1.00	3.00	7.00	8.57	1.00	29.00	0.00	4.00	1.00	17.00	68.00	0.00
1595.00	70.00	70.00	155.00	29.14	3.00	1.00	3.00	2.00	0.00	0.00	0.00	3.00	5.00	17.14	1.00	36.00	1.00	3.00	1.00	13.00	52.00	0.00
1596.00	52.00	75.00	172.00	25.35	3.00	1.00	3.00	2.00	0.00	0.00	0.00	3.00	10.00	0.00	1.00	30.00	1.00	2.00	1.00	13.00	52.00	0.00
1597.00	54.00	81.00	178.00	25.56	3.00	1.00	3.00	2.00	0.00	0.00	1.00	3.00	5.50	21.43	1.00	26.00	0.00	0.00	1.00	17.00	68.00	0.00
1598.00	55.00	80.00	173.00	26.73	3.00	1.00	3.00	2.00	0.00	0.00	1.00	3.00	6.00	55.71	1.00	33.00	1.00	6.00	0.00	12.00	48.00	1.00
1599.00	69.00	90.00	180.00	27.78	3.00	1.00	3.00	2.00	0.00	0.00	1.00	3.00	7.00	30.00	1.00	35.00	1.00	3.00	1.00	17.00	68.00	0.00
1600.00	55.00	83.00	180.00	25.62	3.00	1.00	3.00	2.00	0.00	0.00	1.00	3.00	5.00	55.71	1.00	38.00	1.00	1.00	1.00	19.00	76.00	0.00
1601.00	65.00	73.00	165.00	26.81	3.00	1.00	3.00	2.00	0.00	0.00	1.00	3.00	6.00	51.43	1.00	34.00	1.00	5.00	1.00	15.00	60.00	0.00
1602.00	64.00	84.00	172.00	28.39	3.00	1.00	3.00	3.00	0.00	0.00	1.00	3.00	5.00	31.43	1.00	34.00	1.00	4.00	1.00	18.00	72.00	0.00
1603.00	50.00	94.00	185.00	27.47	3.00	1.00	3.00	2.00	0.00	0.00	1.00	3.00	5.50	25.71	1.00	20.00	0.00	2.00	1.00	21.00	84.00	0.00
1604.00	48.00	75.00	162.00	28.58	3.00	1.00	3.00	3.00	0.00	1.00	0.00	1.00	6.00	0.00	1.00	26.00	0.00	7.00	0.00	9.00	36.00	1.00
1605.00	59.00	74.00	165.00	27.18	3.00	1.00	3.00	1.00	0.00	1.00	0.00	1.00	4.50	25.00	1.00	30.00	1.00	9.00	0.00	15.00	60.00	0.00
1606.00	46.00	85.00	178.00	26.83	3.00	1.00	3.00	2.00	1.00	1.00	1.00	1.00	3.00	51.43	1.00	34.00	1.00	6.00	0.00	13.00	52.00	0.00
1607.00	59.00	89.00	175.00	29.06	3.00	1.00	3.00	3.00	1.00	1.00	1.00	1.00	7.00	43.57	1.00	22.00	0.00	11.00	0.00	15.00	60.00	0.00
1608.00	45.00	85.00	174.00	28.08	3.00	1.00	3.00	1.00	1.00	1.00	1.00	1.00	6.00	37.14	1.00	25.00	0.00	4.00	1.00	15.00	60.00	0.00
1609.00	53.00	85.00	178.00	26.83	3.00	1.00	3.00	2.00	1.00	1.00	1.00	1.00	9.00	12.86	1.00	29.00	0.00	4.00	1.00	15.00	60.00	0.00
1610.00	56.00	88.00	178.00	27.77	3.00	1.00	3.00	2.00	0.00	1.00	1.00	2.00	5.00	20.00	1.00	32.00	1.00	3.00	1.00	10.00	40.00	1.00
1611.00	54.00	92.00	183.00	27.47	3.00	1.00	3.00	2.00	0.00	1.00	1.00	2.00	4.50	0.00	1.00	34.00	1.00	7.00	0.00	12.00	48.00	1.00
1612.00	54.00	76.00	160.00	29.69	3.00	1.00	3.00	2.00	0.00	1.00	0.00	2.00	7.00	0.00	1.00	35.00	1.00	10.00	0.00	9.00	36.00	1.00
1613.00	50.00	100.00	195.00	26.30	3.00	1.00	3.00	2.00	1.00	1.00	1.00	2.00	8.00	0.00	1.00	32.00	1.00	7.00	0.00	9.00	36.00	1.00
1614.00	54.00	80.00	165.00	29.38	3.00	0.00	3.00	2.00	1.00	1.00	1.00	2.00	6.00	30.00	1.00	31.00	1.00	7.00	0.00	12.00	48.00	1.00
1615.00	50.00	95.00	178.00	29.98	3.00	0.00	3.00	2.00	1.00	1.00	1.00	2.00	5.00	4.29	1.00	33.00	1.00	9.00	0.00	15.00	60.00	0.00
1616.00	65.00	73.00	165.00	26.81	3.00	1.00	3.00	2.00	1.00	1.00	1.00	2.00	6.00	10.71	1.00	32.00	1.00	6.00	0.00	12.00	48.00	1.00
1617.00	45.00	75.00	163.00	28.23	3.00	1.00	3.00	2.00	0.00	1.00	0.00	3.00	10.00	2.86	1.00	26.00	0.00	13.00	0.00	10.00	40.00	1.00
1618.00	56.00	95.00	182.00	28.68	3.00	1.00	3.00	2.00	0.00	1.00	1.00	3.00	8.00	12.86	1.00	29.00	0.00	3.00	1.00	10.00	40.00	1.00
1619.00	60.00	90.00	178.00	28.41	3.00	1.00	3.00	2.00	0.00	1.00	1.00	3.00	5.00	8.57	1.00	32.00	1.00	5.00	1.00	18.00	72.00	0.00
1620.00	62.00	85.00	172.00	28.73	3.00	1.00	3.00	2.00	1.00	1.00	1.00	3.00	8.00	34.29	1.00	33.00	1.00	4.00	1.00	18.00	72.00	0.00
1621.00	52.00	88.00	172.00	29.75	3.00	1.00	3.00	2.00	0.00	1.00	1.00	3.00	5.00	42.86	1.00	34.00	1.00	4.00	1.00	14.00	56.00	0.00
1622.00	65.00	90.00	181.00	27.47	3.00	1.00	3.00	2.00	0.00	1.00	1.00	3.00	6.00	10.71	1.00	31.00	1.00	3.00	1.00	12.00	48.00	1.00
1623.00	64.00	84.00	182.00	25.36	3.00	1.00	3.00	2.00	0.00	1.00	1.00	3.00	4.00	31.43	1.00	38.00	1.00	4.00	1.00	14.00	56.00	0.00
1624.00	45.00	93.00	186.00	26.88	3.00	1.00	3.00	2.00	0.00	1.00	1.00	3.00	5.00	60.00	1.00	27.00	0.00	2.00	1.00	21.00	84.00	0.00
1625.00	45.00	67.00	169.00	23.46	2.00	1.00	3.00	2.00	1.00	0.00	0.00	2.00	8.00	32.14	1.00	32.00	1.00	4.00	1.00	9.00	36.00	1.00
1626.00	50.00	57.00	158.00	22.83	2.00	1.00	3.00	2.00	0.00	0.00	0.00	2.00	9.00	34.29	1.00	33.00	1.00	4.00	1.00	11.00	44.00	1.00
1627.00	46.00	70.00	178.00	22.09	2.00	1.00	3.00	2.00	1.00	0.00	1.00	2.00	8.00	2.14	1.00	21.00	0.00	6.00	0.00	12.00	48.00	1.00
1628.00	50.00	68.00	180.00	20.99	2.00	1.00	3.00	2.00	1.00	0.00	1.00	2.00	4.00	18.57	1.00	24.00	0.00	8.00	0.00	14.00	56.00	0.00
1629.00	47.00	63.00	165.00	23.14	2.00	0.00	3.00	2.00	1.00	0.00	0.00	2.00	10.00	0.00	1.00	32.00	1.00	8.00	0.00	12.00	48.00	1.00
1630.00	46.00	70.00	170.00	24.22	2.00	1.00	3.00	1.00	0.00	0.00	0.00	2.00	8.00	6.43	1.00	36.00	1.00	3.00	1.00	21.00	84.00	0.00
1631.00	51.00	68.00	173.00	22.72	2.00	1.00	3.00	2.00	0.00	0.00	0.00	3.00	4.00	0.00	1.00	34.00	1.00	4.00	1.00	16.00	64.00	0.00
1632.00	48.00	76.00	175.00	24.82	2.00	1.00	3.00	2.00	1.00	0.00	1.00	3.00	7.00	0.00	1.00	25.00	0.00	12.00	0.00	9.00	36.00	1.00
1633.00	50.00	55.00	160.00	21.48	2.00	1.00	3.00	2.00	0.00	0.00	0.00	3.00	6.00	0.00	1.00	29.00	0.00	3.00	1.00	13.00	52.00	0.00
1634.00	56.00	60.00	165.00	22.04	2.00	1.00	3.00	2.00	0.00	0.00	0.00	3.00	6.00	38.57	1.00	40.00	1.00	7.00	0.00	15.00	60.00	0.00
1635.00	60.00	66.00	165.00	24.24	2.00	1.00	3.00	2.00	0.00	0.00	1.00	3.00	5.00	12.86	1.00	32.00	1.00	3.00	1.00	15.00	60.00	0.00
1636.00	46.00	65.00	164.00	24.17	2.00	1.00	3.00	2.00	0.00	0.00	0.00	3.00	6.50	17.14	1.00	31.00	1.00	4.00	1.00	16.00	64.00	0.00
1637.00	46.00	83.00	185.00	24.25	2.00	1.00	3.00	2.00	1.00	1.00	1.00	1.00	11.00	4.29	1.00	32.00	1.00	10.00	0.00	16.00	64.00	0.00
1638.00	55.00	80.00	180.00	24.69	2.00	1.00	3.00	3.00	0.00	1.00	1.00	1.00	6.00	27.86	1.00	34.00	1.00	3.00	1.00	16.00	64.00	0.00
1639.00	52.00	75.00	174.00	24.77	2.00	1.00	3.00	2.00	0.00	1.00	1.00	2.00	6.00	51.43	1.00	30.00	1.00	9.00	0.00	15.00	60.00	0.00
1640.00	49.00	54.00	163.00	20.32	2.00	1.00	3.00	1.00	0.00	1.00	0.00	3.00	6.50	0.00	1.00	27.00	0.00	5.00	1.00	17.00	68.00	0.00
1641.00	51.00	63.00	162.00	24.01	2.00	1.00	3.00	2.00	0.00	1.00	0.00	3.00	9.00	14.29	1.00	34.00	1.00	2.00	1.00	20.00	80.00	0.00
1642.00	53.00	90.00	169.00	31.51	4.00	1.00	2.00	2.00	1.00	0.00	1.00	3.00	2.00	45.71	1.00	27.00	0.00	6.00	0.00	12.00	48.00	1.00
1643.00	49.00	73.00	153.00	31.18	4.00	1.00	2.00	2.00	0.00	1.00	0.00	2.00	5.00	62.86	1.00	33.00	1.00	8.00	0.00	21.00	84.00	0.00
1644.00	54.00	110.00	167.00	39.44	4.00	0.00	2.00	2.00	0.00	0.00	0.00	2.00	11.00	0.00	1.00	41.00	1.00	6.00	0.00	14.00	56.00	0.00
1645.00	46.00	115.00	180.00	35.49	4.00	1.00	2.00	2.00	0.00	0.00	1.00	3.00	7.00	34.29	1.00	35.00	1.00	6.00	0.00	11.00	44.00	1.00
1646.00	53.00	86.00	157.00	34.89	4.00	1.00	2.00	2.00	0.00	0.00	0.00	3.00	6.00	3.57	1.00	29.00	0.00	11.00	0.00	11.00	44.00	1.00
1647.00	52.00	100.00	180.00	30.86	4.00	1.00	2.00	2.00	0.00	0.00	1.00	3.00	5.00	25.71	1.00	35.00	1.00	6.00	0.00	21.00	84.00	0.00
1648.00	50.00	77.00	158.00	30.84	4.00	1.00	2.00	2.00	1.00	1.00	1.00	1.00	11.00	20.71	1.00	24.00	0.00	8.00	0.00	10.00	40.00	1.00
1649.00	57.00	92.00	159.00	36.39	4.00	1.00	2.00	2.00	1.00	1.00	0.00	1.00	8.00	34.29	1.00	38.00	1.00	8.00	0.00	13.00	52.00	0.00
1650.00	56.00	100.00	180.00	30.86	4.00	1.00	2.00	2.00	0.00	1.00	1.00	1.00	3.50	25.71	1.00	26.00	0.00	3.00	1.00	9.00	36.00	1.00

Subject	Age	Weight	Height	BMI	BMI_C	Residence	Education	Marital_Status	Smoking	Chronic_Dise	Gender	Health	Sitting_Time	PA_Min_Day	IPAQ_C	Dietary_Score	Dietary_C	PSQI_Total	Sleep_C	WHO5_Total	WHO5_Total100	WHO5_Class
**1651.00**	59.00	90.00	158.00	36.05	4.00	1.00	2.00	2.00	0.00	1.00	0.00	2.00	3.00	0.00	1.00	35.00	1.00	8.00	0.00	10.00	40.00	1.00
**1652.00**	52.00	99.00	180.00	30.56	4.00	1.00	2.00	2.00	1.00	1.00	1.00	2.00	3.50	55.71	1.00	36.00	1.00	8.00	0.00	8.00	32.00	1.00
**1653.00**	52.00	102.00	173.00	34.08	4.00	1.00	2.00	2.00	1.00	1.00	1.00	2.00	5.00	27.86	1.00	33.00	1.00	9.00	0.00	12.00	48.00	1.00
**1654.00**	60.00	94.00	177.00	30.00	4.00	1.00	2.00	2.00	1.00	1.00	1.00	2.00	6.00	4.29	1.00	29.00	0.00	3.00	1.00	12.00	48.00	1.00
**1655.00**	47.00	162.00	184.00	47.85	4.00	1.00	2.00	2.00	1.00	1.00	1.00	2.00	8.00	8.57	1.00	30.00	1.00	11.00	0.00	12.00	48.00	1.00
**1656.00**	45.00	100.00	178.00	31.56	4.00	1.00	2.00	2.00	0.00	1.00	1.00	3.00	6.00	30.00	1.00	34.00	1.00	4.00	1.00	12.00	48.00	1.00
**1657.00**	51.00	100.00	159.00	39.56	4.00	1.00	3.00	1.00	0.00	0.00	0.00	3.00	8.00	17.14	1.00	22.00	0.00	2.00	1.00	19.00	76.00	0.00
**1658.00**	48.00	98.00	170.00	33.91	4.00	1.00	3.00	2.00	0.00	1.00	1.00	1.00	7.00	12.86	1.00	32.00	1.00	7.00	0.00	10.00	40.00	1.00
**1659.00**	46.00	100.00	181.00	30.52	4.00	1.00	3.00	2.00	0.00	1.00	1.00	2.00	6.00	10.71	1.00	27.00	0.00	8.00	0.00	13.00	52.00	0.00
**1660.00**	45.00	78.00	160.00	30.47	4.00	1.00	3.00	2.00	0.00	1.00	0.00	3.00	8.00	14.29	1.00	28.00	0.00	5.00	1.00	13.00	52.00	0.00
**1661.00**	50.00	105.00	182.00	31.70	4.00	1.00	3.00	2.00	0.00	1.00	1.00	3.00	11.00	0.00	1.00	25.00	0.00	4.00	1.00	12.00	48.00	1.00
**1662.00**	51.00	91.00	158.00	36.45	4.00	1.00	3.00	1.00	0.00	1.00	0.00	3.00	6.50	4.29	1.00	37.00	1.00	3.00	1.00	18.00	72.00	0.00
**1663.00**	49.00	76.00	173.00	25.39	3.00	0.00	2.00	2.00	0.00	0.00	1.00	3.00	8.00	6.43	1.00	23.00	0.00	6.00	0.00	17.00	68.00	0.00
**1664.00**	49.00	80.00	169.00	28.01	3.00	1.00	2.00	3.00	1.00	1.00	0.00	1.00	11.00	25.71	1.00	41.00	1.00	9.00	0.00	12.00	48.00	1.00
**1665.00**	50.00	71.00	156.00	29.17	3.00	1.00	2.00	2.00	0.00	1.00	0.00	3.00	3.50	4.29	1.00	32.00	1.00	9.00	0.00	10.00	40.00	1.00
**1666.00**	45.00	90.00	185.00	26.30	3.00	1.00	2.00	2.00	0.00	0.00	1.00	2.00	8.00	12.86	1.00	31.00	1.00	3.00	1.00	13.00	52.00	0.00
**1667.00**	46.00	78.00	164.00	29.00	3.00	1.00	2.00	2.00	0.00	0.00	0.00	3.00	2.50	0.00	1.00	29.00	0.00	7.00	0.00	7.00	28.00	1.00
**1668.00**	53.00	80.00	170.00	27.68	3.00	1.00	2.00	2.00	0.00	0.00	1.00	3.00	5.00	22.14	1.00	27.00	0.00	7.00	0.00	13.00	52.00	0.00
**1669.00**	48.00	88.00	177.00	28.09	3.00	1.00	2.00	2.00	0.00	0.00	1.00	3.00	6.00	17.14	1.00	28.00	0.00	7.00	0.00	10.00	40.00	1.00
**1670.00**	48.00	80.00	167.00	28.69	3.00	0.00	2.00	2.00	0.00	0.00	0.00	3.00	2.50	19.29	1.00	34.00	1.00	4.00	1.00	13.00	52.00	0.00
**1671.00**	61.00	75.00	173.00	25.06	3.00	1.00	2.00	2.00	1.00	1.00	1.00	1.00	7.00	4.29	1.00	34.00	1.00	5.00	1.00	13.00	52.00	0.00
**1672.00**	61.00	75.00	173.00	25.06	3.00	1.00	2.00	2.00	1.00	1.00	1.00	1.00	8.00	6.43	1.00	35.00	1.00	5.00	1.00	13.00	52.00	0.00
**1673.00**	51.00	85.00	175.00	27.76	3.00	1.00	2.00	2.00	0.00	1.00	1.00	2.00	3.00	0.00	1.00	19.00	0.00	7.00	0.00	4.00	16.00	1.00
**1674.00**	53.00	80.00	165.00	29.38	3.00	1.00	2.00	2.00	0.00	1.00	1.00	2.00	6.00	9.43	1.00	30.00	1.00	13.00	0.00	2.00	8.00	1.00
**1675.00**	60.00	75.00	168.00	26.57	3.00	1.00	2.00	3.00	0.00	1.00	0.00	2.00	11.00	11.43	1.00	40.00	1.00	7.00	0.00	15.00	60.00	0.00
**1676.00**	54.00	100.00	184.00	29.54	3.00	1.00	2.00	2.00	0.00	1.00	1.00	2.00	11.00	0.00	1.00	33.00	1.00	8.00	0.00	13.00	52.00	0.00
**1677.00**	45.00	90.00	184.00	26.58	3.00	1.00	2.00	2.00	1.00	1.00	1.00	2.00	10.00	0.00	1.00	20.00	0.00	8.00	0.00	11.00	44.00	1.00
**1678.00**	55.00	80.00	177.00	25.54	3.00	1.00	2.00	2.00	1.00	1.00	1.00	2.00	4.00	53.57	1.00	41.00	1.00	1.00	1.00	18.00	72.00	0.00
**1679.00**	52.00	78.00	174.00	25.76	3.00	1.00	2.00	2.00	0.00	1.00	1.00	3.00	4.00	34.29	1.00	30.00	1.00	7.00	0.00	10.00	40.00	1.00
**1680.00**	50.00	88.00	179.00	27.46	3.00	1.00	2.00	2.00	0.00	1.00	1.00	3.00	9.00	0.00	1.00	37.00	1.00	13.00	0.00	15.00	60.00	0.00
**1681.00**	49.00	74.00	170.00	25.61	3.00	1.00	3.00	2.00	0.00	0.00	0.00	3.00	4.00	4.29	1.00	31.00	1.00	7.00	0.00	16.00	64.00	0.00
**1682.00**	47.00	76.00	170.00	26.30	3.00	1.00	3.00	2.00	0.00	0.00	1.00	3.00	4.00	17.14	1.00	28.00	0.00	5.00	1.00	12.00	48.00	1.00
**1683.00**	68.00	74.00	165.00	27.18	3.00	1.00	3.00	2.00	1.00	1.00	1.00	1.00	7.00	17.14	1.00	35.00	1.00	8.00	0.00	12.00	48.00	1.00
**1684.00**	65.00	80.00	173.00	26.73	3.00	1.00	3.00	2.00	0.00	1.00	1.00	2.00	7.00	8.57	1.00	36.00	1.00	6.00	0.00	12.00	48.00	1.00
**1685.00**	45.00	64.00	169.00	22.41	2.00	1.00	2.00	2.00	0.00	0.00	1.00	2.00	4.00	22.86	1.00	32.00	1.00	4.00	1.00	13.00	52.00	0.00
**1686.00**	48.00	52.00	154.00	21.93	2.00	1.00	2.00	2.00	0.00	0.00	0.00	3.00	5.00	0.00	1.00	23.00	0.00	8.00	0.00	6.00	24.00	1.00
**1687.00**	48.00	78.00	186.00	22.55	2.00	1.00	2.00	2.00	0.00	0.00	1.00	3.00	6.00	19.29	1.00	24.00	0.00	6.00	0.00	10.00	40.00	1.00
**1688.00**	59.00	58.00	167.00	20.80	2.00	1.00	2.00	2.00	0.00	0.00	1.00	3.00	5.50	41.43	1.00	32.00	1.00	6.00	0.00	12.00	48.00	1.00
**1689.00**	45.00	87.00	190.00	24.10	2.00	1.00	2.00	2.00	1.00	1.00	1.00	2.00	5.00	42.86	1.00	26.00	0.00	2.00	1.00	15.00	60.00	0.00
**1690.00**	50.00	52.00	167.00	18.65	2.00	1.00	3.00	2.00	1.00	0.00	1.00	2.00	5.00	30.00	1.00	34.00	1.00	5.00	1.00	20.00	80.00	0.00
**1691.00**	50.00	75.00	178.00	23.67	2.00	1.00	3.00	2.00	0.00	0.00	1.00	3.00	4.00	55.71	1.00	27.00	0.00	8.00	0.00	16.00	64.00	0.00
**1692.00**	48.00	70.00	168.00	24.80	2.00	1.00	3.00	2.00	1.00	0.00	1.00	3.00	7.00	60.00	1.00	38.00	1.00	4.00	1.00	15.00	60.00	0.00
**1693.00**	67.00	100.00	171.00	34.20	4.00	1.00	1.00	2.00	1.00	1.00	1.00	1.00	11.00	0.00	1.00	21.00	0.00	9.00	0.00	9.00	36.00	1.00
**1694.00**	54.00	110.00	175.00	35.92	4.00	1.00	2.00	2.00	0.00	0.00	1.00	3.00	2.50	45.00	1.00	25.00	0.00	2.00	1.00	18.00	72.00	0.00
**1695.00**	62.00	105.00	182.00	31.70	4.00	1.00	2.00	3.00	0.00	0.00	1.00	3.00	4.00	12.86	1.00	38.00	1.00	3.00	1.00	15.00	60.00	0.00
**1696.00**	48.00	71.00	150.00	31.56	4.00	1.00	2.00	2.00	0.00	0.00	0.00	3.00	2.00	25.00	1.00	31.00	1.00	3.00	1.00	13.00	52.00	0.00
**1697.00**	60.00	100.00	174.00	33.03	4.00	0.00	2.00	2.00	1.00	1.00	1.00	1.00	7.00	8.57	1.00	24.00	0.00	9.00	0.00	13.00	52.00	0.00
**1698.00**	60.00	86.00	168.00	30.47	4.00	1.00	2.00	2.00	0.00	1.00	1.00	2.00	6.00	10.71	1.00	27.00	0.00	10.00	0.00	15.00	60.00	0.00
**1699.00**	58.00	96.00	176.00	30.99	4.00	1.00	2.00	2.00	0.00	1.00	1.00	3.00	4.00	17.14	1.00	30.00	1.00	4.00	1.00	16.00	64.00	0.00
**1700.00**	48.00	94.00	167.00	33.71	4.00	1.00	3.00	2.00	0.00	0.00	1.00	2.00	9.00	0.00	1.00	31.00	1.00	2.00	1.00	15.00	60.00	0.00
**1701.00**	48.00	103.00	160.00	40.23	4.00	1.00	3.00	1.00	0.00	0.00	0.00	3.00	4.00	0.00	1.00	27.00	0.00	4.00	1.00	14.00	56.00	0.00
**1702.00**	66.00	78.00	172.00	26.37	3.00	1.00	1.00	2.00	1.00	1.00	1.00	2.00	7.00	25.71	1.00	35.00	1.00	10.00	0.00	4.00	16.00	1.00
**1703.00**	70.00	85.00	175.00	27.76	3.00	1.00	1.00	2.00	1.00	1.00	1.00	2.00	6.50	2.86	1.00	31.00	1.00	6.00	0.00	15.00	60.00	0.00
**1704.00**	50.00	75.00	173.00	25.06	3.00	1.00	2.00	2.00	0.00	0.00	0.00	3.00	5.00	0.00	1.00	27.00	0.00	3.00	1.00	14.00	56.00	0.00
**1705.00**	78.00	75.00	160.00	29.30	3.00	1.00	2.00	2.00	0.00	1.00	1.00	1.00	10.00	8.57	1.00	27.00	0.00	10.00	0.00	14.00	56.00	0.00
**1706.00**	55.00	89.00	176.00	28.73	3.00	1.00	2.00	3.00	0.00	1.00	1.00	2.00	10.00	12.86	1.00	27.00	0.00	9.00	0.00	6.00	24.00	1.00
**1707.00**	57.00	90.00	177.00	28.73	3.00	1.00	2.00	2.00	1.00	1.00	1.00	2.00	8.00	64.29	1.00	36.00	1.00	6.00	0.00	13.00	52.00	0.00
**1708.00**	49.00	92.00	179.00	28.71	3.00	1.00	2.00	2.00	0.00	1.00	1.00	2.00	1.00	30.00	1.00	35.00	1.00	1.00	1.00	19.00	76.00	0.00
**1709.00**	50.00	69.00	158.00	27.64	3.00	1.00	2.00	3.00	1.00	1.00	0.00	3.00	9.00	0.00	1.00	22.00	0.00	5.00	1.00	17.00	68.00	0.00
**1710.00**	58.00	80.00	171.00	27.36	3.00	0.00	3.00	2.00	1.00	1.00	1.00	1.00	4.00	4.29	1.00	23.00	0.00	12.00	0.00	1.00	4.00	1.00
**1711.00**	45.00	79.00	172.00	26.70	3.00	1.00	3.00	2.00	0.00	1.00	0.00	2.00	7.00	51.43	1.00	33.00	1.00	5.00	1.00	14.00	56.00	0.00
**1712.00**	55.00	80.00	170.00	27.68	3.00	1.00	3.00	2.00	0.00	1.00	1.00	2.00	8.00	0.86	1.00	30.00	1.00	3.00	1.00	13.00	52.00	0.00
**1713.00**	66.00	66.00	166.00	23.95	2.00	1.00	1.00	2.00	0.00	0.00	0.00	2.00	12.00	15.71	1.00	20.00	0.00	5.00	1.00	7.00	28.00	1.00
**1714.00**	51.00	55.00	165.00	20.20	2.00	1.00	1.00	2.00	0.00	0.00	1.00	3.00	3.00	72.86	1.00	22.00	0.00	9.00	0.00	19.00	76.00	0.00
**1715.00**	76.00	67.00	167.00	24.02	2.00	1.00	1.00	2.00	1.00	1.00	1.00	2.00	1.00	0.00	1.00	20.00	0.00		0.00	8.00	32.00	1.00
**1716.00**	62.00	70.00	176.00	22.60	2.00	1.00	1.00	2.00	1.00	1.00	1.00	3.00	9.00	55.71	1.00	34.00	1.00	5.00	1.00	12.00	48.00	1.00

Subject	Age	Weight	Height	BMI	BMI_C	Residence	Education	Marital_Status	Smoking	Chronic_Dise	Gender	Health	Sitting_Time	PA_Min_Day	IPAQ_C	Dietary_Score	Dietary_C	PSQI_Total	Sleep_C	WHO5_Total	WHO5_Total100	WHO5_Class
1717.00	66.00	68.00	168.00	24.09	2.00	1.00	2.00	2.00	0.00	0.00	1.00	3.00	10.00	36.43	1.00	36.00	1.00	3.00	1.00	15.00	60.00	0.00
1718.00	49.00	60.00	163.00	22.58	2.00	1.00	2.00	2.00	1.00	0.00	0.00	3.00	6.00	38.57	1.00	32.00	1.00	6.00	0.00	16.00	64.00	0.00
1719.00	46.00	78.00	178.00	24.62	2.00	1.00	2.00	2.00	1.00	0.00	1.00	3.00	6.00	30.00	1.00	29.00	0.00	4.00	1.00	11.00	44.00	1.00
1720.00	65.00	76.00	182.00	22.94	2.00	1.00	2.00	2.00	0.00	1.00	1.00	2.00	6.00	21.43	1.00	39.00	1.00	12.00	0.00	12.00	48.00	1.00
1721.00	53.00	57.00	165.00	20.94	2.00	1.00	2.00	3.00	0.00	1.00	0.00	2.00	5.00	141.43	1.00	33.00	1.00	12.00	0.00	15.00	60.00	0.00
1722.00	60.00	70.00	170.00	24.22	2.00	1.00	3.00	2.00	0.00	1.00	1.00	2.00	6.00	1.43	1.00	29.00	0.00	3.00	1.00	12.00	48.00	1.00
1723.00	73.00	70.00	170.00	24.22	2.00	1.00	3.00	2.00	1.00	1.00	1.00	3.00	4.00	17.14	1.00	33.00	1.00	7.00	0.00	15.00	60.00	0.00
